# Large Language Models in Shoulder Dislocation: Performance Across Medical and Rehabilitation Domains

**DOI:** 10.3390/healthcare14213726

**Published:** 2026-11-03

**Authors:** İnci Hazal Ayas, Uğur Sözlü, Bülent İnce, Ulunay Kanatlı

**Affiliations:** 1 Department of Physiotherapy and Rehabilitation, Kulu Faculty of Health Sciences, Selçuk University, 42770 Konya, Türkiye; 2 Department of Physiotherapy and Rehabilitation, Faculty of Health Sciences, Tokat Gaziosmanpaşa University, 60250 Tokat, Türkiye; 3 Department of Orthopaedics and Traumatology, Demirtepe Life Hospital, 06570 Ankara, Türkiye; 4 Department of Orthopaedics and Traumatology, Faculty of Medicine, Gazi University, 06500 Ankara, Türkiye

**Keywords:** artificial intelligence, large language models, shoulder dislocation, rehabilitation, sports medicine, readability

## Abstract

**Highlights:**

**What are the main findings?**

• Large language models provided generally accurate and high-quality information about first-time traumatic anterior shoulder dislocation.
• Significant differences among models were observed in medical and safety questions, whereas no significant differences in accuracy, quality, or information reliability were detected in rehabilitation-related questions.

**What are the implications of the main findings?**

• LLM performance should be evaluated across different types of clinical questions, while recognizing the limitations of direct comparisons between clinical domains.
• Greater attention is warranted when LLMs are used for medical and safety questions involving acute management, recurrence, imaging, and treatment decisions.

**Abstract:**

Background/Objectives: Large language models (LLMs) are increasingly used to obtain health information about musculoskeletal conditions. However, their performance across different types of clinical questions remains insufficiently understood. This study evaluated the accuracy, quality, information reliability, and readability of responses generated by three widely used LLMs (ChatGPT 5.2, Gemini 3, and DeepSeek V3.2) to questions about first-time traumatic anterior shoulder dislocation, with separate analyses of medical and rehabilitation questions. Methods: Sixty-five clinically relevant questions were categorized into Medical and Safety (*n* = 28) and Rehabilitation (*n* = 37) domains. Responses were independently evaluated by domain experts using a 5-point accuracy scale, the Global Quality Scale (GQS), modified DISCERN (mDISCERN), and Flesch Reading Ease (FRE). Model differences were analyzed using Friedman tests with post hoc pairwise comparisons, and inter-rater agreement was assessed using intraclass correlation coefficients. Results: In the Medical and Safety Domain, the models differed significantly in accuracy (*p* < 0.001), quality (*p* < 0.001), information reliability (*p* = 0.004), and readability (*p* = 0.011). ChatGPT 5.2 and Gemini 3 had higher accuracy and GQS scores than DeepSeek V3.2, while DeepSeek V3.2 showed significantly higher mDISCERN scores than ChatGPT 5.2. Gemini 3 demonstrated significantly higher FRE scores than DeepSeek V3.2. In the Rehabilitation Domain, no significant differences were found in accuracy, quality, or information reliability (all *p* > 0.05), whereas readability differed significantly (*p* < 0.001), with ChatGPT 5.2 and Gemini 3 showing higher FRE scores than DeepSeek V3.2. Median accuracy and GQS scores were 5 for all three models in the Rehabilitation Domain, suggesting a potential ceiling effect. Readability was relatively difficult across the models, with no statistically significant correlations between FRE scores and accuracy, GQS, or mDISCERN scores. Inter-rater agreement ranged from moderate to excellent. Conclusions: LLMs provided generally accurate and high-quality information about first-time traumatic anterior shoulder dislocation. In separate domain-specific analyses, significant differences among models were observed in accuracy, quality, information reliability, and readability for medical and safety questions. For rehabilitation questions, statistically significant differences were detected only in readability. These findings do not establish a statistically significant difference in model performance between the two clinical domains. LLM-generated information should be interpreted cautiously, particularly when addressing medical decisions and patient safety.

## 1. Introduction

Traumatic anterior shoulder dislocation is a common musculoskeletal injury, particularly among young and physically active individuals. Following a first-time dislocation, recurrent instability is an important concern, especially in younger and more active patients [1]. Management extends beyond the initial reduction and may include activity modification, clinical follow-up, rehabilitation, and decisions regarding return to sport or surgical treatment [1]. Rehabilitation plays an important role in restoring shoulder function and facilitating a safe return to sport following anterior shoulder instability [2]. Consequently, patients may seek information not only about the acute injury and risk of recurrence but also about recovery, exercise, daily activities, rehabilitation, and return to sport. Shoulder dislocation is a common musculoskeletal injury, with a reported incidence of 23.9 per 100,000 person-years in the United States. Nearly half of these injuries occur in individuals aged 15–29 years, highlighting their relevance to young and physically active populations [3].

Patients increasingly use online resources to obtain information about musculoskeletal conditions and treatment options [4]. However, the quality, information reliability, and readability of online health information can vary considerably [5]. This is particularly relevant after shoulder dislocation because patients may encounter questions concerning immobilization, recurrence risk, imaging, rehabilitation, exercise progression, and return to activity throughout different stages of recovery. Information that is inaccurate, unreliable, or difficult to understand may limit its usefulness for patients. Therefore, patient-oriented health information should be both clinically appropriate and comprehensible.

Large language models (LLMs), such as ChatGPT, Gemini, and DeepSeek, have become readily accessible sources of health information [6]. Unlike traditional search engines, LLMs can generate direct, conversational responses to individual questions [7], potentially making health information easier to access for patients seeking guidance about their condition, treatment, and recovery. Previous studies have shown that LLMs can provide generally accurate and clinically useful responses to questions concerning orthopaedic conditions [8]. Nevertheless, performance may vary according to the model and the type of clinical question being asked [7]. Moreover, clinically accurate information is not necessarily easy for patients to understand. Readability therefore represents an additional and distinct consideration when evaluating AI-generated health information [9].

Although the use of LLMs in healthcare continues to increase [10], their performance across different types of questions related to shoulder dislocation has not been clearly established. Patients with a first-time traumatic anterior shoulder dislocation may ask questions ranging from acute management, recurrence, imaging, and treatment decisions to daily activities, exercise, rehabilitation progression, and return to sport. Medical and safety questions and rehabilitation-related questions differ in their clinical purpose and may therefore present different challenges for LLMs. Evaluating these domains separately may reveal differences in model performance that could be obscured when responses are assessed only as an overall group.

Therefore, this study aimed to evaluate the accuracy, quality, information reliability, and readability of information generated by three widely used LLMs (ChatGPT 5.2, Gemini 3, and DeepSeek V3.2) in response to standardized questions about first-time traumatic anterior shoulder dislocation. Responses were evaluated separately in the Medical and Safety Domain and the Rehabilitation Domain to examine differences among models within each clinical domain.

## 2. Materials and Methods

### 2.1. Study Design

This cross-sectional comparative evaluation assessed the quality, information reliability, accuracy, and readability of responses generated by three large language models to common patient-oriented questions about the management and rehabilitation of first-time traumatic anterior shoulder dislocation.

### 2.2. Question Development and LLM Response Generation

A total of 65 clinically relevant questions were developed to reflect common concerns following a first-time shoulder dislocation. A multidisciplinary team consisting of two orthopedic surgeons and two physiotherapists with experience in shoulder rehabilitation developed the questions based on routine clinical practice, commonly encountered patient questions, and current evidence on shoulder instability [1,2]. An initial set of 60 questions was developed and subsequently expanded to 65 to broaden the coverage of clinically relevant topics. All four experts reviewed the complete question set and jointly agreed on the final selection and classification of the questions. The questions were organized into two broad clinical domains reflecting the expertise of the multidisciplinary team, while avoiding excessive subdivision into small groups. All decisions were reached by consensus, without disagreements requiring resolution. No formal pilot testing or independent content-validation procedure was conducted. The complete list of questions is provided in the Supplementary Table S1. The questions were grouped into two clinical domains:

Medical and Safety Domain (MD, *n* = 28): Questions related to acute management, post-reduction care, safety, recurrence risk, and follow-up.

Rehabilitation Domain (RD, *n* = 37): Questions related to daily activities, exercise, rehabilitation planning, muscle strength, and return to sport.

The questions were also grouped by their main clinical topics. The Medical and Safety Domain included acute management and safety (*n* = 8), immobilization and post-reduction symptoms (*n* = 7), recurrence and instability (*n* = 6), follow-up and surgical management (*n* = 4), pain medication (*n* = 2), and imaging (*n* = 1). The Rehabilitation Domain included daily activities (*n* = 11), exercise initiation and progression (*n* = 9), strengthening and shoulder stability (*n* = 8), and return to sport (*n* = 9). This grouping was used only to describe the questions. The clinical topic of each question is shown in Supplementary Table S1. The Medical and Safety Domain was defined according to question content and included medical management topics with potential safety implications, such as acute management, post-reduction care, recurrence risk, imaging, and treatment decisions. This domain designation was distinct from the retrospective clinical safety assessment, which evaluated potentially consequential errors and recommendations in responses from both clinical domains. Three large language models (ChatGPT 5.2, Gemini 3, and DeepSeek V3.2) were evaluated. Responses were generated between 1 and 14 May 2026 using the free web-based versions of ChatGPT 5.2 (OpenAI, San Francisco, CA, USA), Gemini 3 (Google LLC, Mountain View, CA, USA), and DeepSeek V3.2 (DeepSeek AI, Hangzhou, China). All models were accessed using their default settings. No specific reasoning mode was manually selected, and no additional search settings were applied. Web browsing capabilities were available during response generation. No model parameters were manually modified, and no additional documents were uploaded. All questions were submitted in English, and all responses were generated in English. To ensure consistency, the same standardized prompt was used for all models:

“Please answer the following question for a young patient with a shoulder dislocation. Assume a first-time traumatic anterior shoulder dislocation following successful reduction, with no associated fracture or neurovascular injury, unless otherwise specified in the question.”

The phrase “unless otherwise specified in the question” was included to account for question-specific clinical circumstances, particularly those involving acute symptoms, emergency management, or recurrent dislocation. The same standardized prompt was applied to all 65 questions across the three LLMs.

Each question was asked using the same prompt structure in a new chat session to prevent previous conversations from influencing the responses. No follow-up prompts, response regeneration, or prompt modifications were used. The complete set of 195 generated responses is provided in Supplementary File S1, while the final consensus evaluation scores are available in Supplementary File S2.

### 2.3. Outcome Measures

Responses were evaluated by domain experts. The responses were anonymized to conceal the identity of the LLMs and presented to the evaluators in a randomized order. Before scoring, the evaluators discussed the assessment scales and scoring criteria to ensure a consistent understanding of the evaluation process. Questions in the Medical and Safety Domain were assessed by two orthopedic surgeons specializing in shoulder surgery and arthroscopy, whereas questions in the Rehabilitation Domain were assessed by two physiotherapists with PhD degrees and expertise in shoulder rehabilitation. The same four experts were involved in both question development and response evaluation. The two evaluators independently scored each response before discussing any disagreements. When scores differed, the evaluators reviewed the response together and agreed on a final consensus score. ICC values were calculated using the initial independent ratings, whereas the consensus scores were used for the main statistical analyses. Supplementary File S2 contains the initial independent ratings used for the ICC analyses and the final consensus scores used for the main statistical analyses.

Information reliability was assessed using the modified DISCERN (mDISCERN) scale. The scale includes five items that evaluate clarity, source reliability, balance, referencing, and uncertainty. Each item was scored as 1 (yes) or 0 (no), giving a total score between 0 and 5 [11,12]. Higher scores indicate greater information reliability.

Accuracy was evaluated using a 5-point Likert scale based on current clinical evidence and accepted medical practice [8]. A score of 1 indicated incorrect information with no relevant information, while a score of 2 indicated a mixture of correct and incorrect information. A score of 3 represented correct information with substantial missing, unnecessary, or confusing content. A score of 4 indicated accurate information with only minimal missing or unnecessary content, whereas a score of 5 represented complete and accurate information addressing the question.

Overall quality was evaluated using the Global Quality Scale (GQS) [13]. Scores range from 1 (poor quality) to 5 (excellent quality).

These measures were selected to assess different but related aspects of response performance. Accuracy evaluated the correctness and completeness of clinical information, GQS assessed overall information quality, and mDISCERN assessed indicators of health information reliability, including source transparency and uncertainty. Although these measures may overlap, none directly measures clinical safety.

Readability was assessed using the standard Flesch Reading Ease (FRE) formula [14,15]. FRE scores were calculated using a custom calculator developed in Microsoft Excel 2019 (Microsoft Corporation, Redmond, WA, USA) with Visual Basic for Applications (VBA). The formula was FRE = 206.835 − (1.015 × ASL) − (84.6 × ASW), where ASL represents the average number of words per sentence and ASW represents the average number of syllables per word [14,15]. The calculator estimated syllable counts using vowel groups and predefined rules for English word endings, with a minimum of one syllable per word. Sentence boundaries were identified using periods, question marks, and exclamation marks, with rule-based checks for common abbreviations to reduce incorrect sentence splitting. Bullet symbols and line breaks were replaced with spaces instead of being treated as sentence boundaries. Higher FRE scores indicate easier-to-read text. Scores of 90–100 indicate very easy readability, 60–70 indicate standard readability, and scores below 30 indicate very difficult text.

In addition to the original outcome measures, a focused retrospective clinical safety assessment was conducted to identify potentially consequential errors in the generated responses. All 195 responses were retrospectively screened for potential clinical safety concerns. The screening was conducted jointly by two orthopedic surgeons for the Medical and Safety Domain and two physiotherapists for the Rehabilitation Domain. Potential safety concerns were identified and discussed by the two evaluators in each domain.

Following the initial screening, 18 responses with potential clinical safety concerns were flagged for further review. These responses were jointly assessed by the same evaluators using two binary criteria: (1) clinically important errors or omissions that could negatively affect patient safety or clinical decision-making, and (2) unsupported or overly definitive clinical recommendations made without sufficient clinical justification or consideration of individual patient factors. Each criterion was coded as 0 (absent) or 1 (present). The two criteria assessed different aspects of clinical safety and were not mutually exclusive. A response could contain an unsupported or overly definitive recommendation that was also considered clinically important because of its potential effect on patient safety or clinical decision-making. The findings were summarized descriptively by model and clinical domain.

### 2.4. Statistical Analysis

Statistical analyses were performed using IBM SPSS Statistics version 27 (IBM Corp., Armonk, NY, USA).

Data distribution was evaluated using histograms, Q-Q plots, and the Kolmogorov–Smirnov and Shapiro–Wilk tests. Because the data were not normally distributed, results are presented as median and interquartile range (IQR).

Consensus scores were used for comparisons between the three LLMs and for correlation analyses. Differences between the three LLMs were analyzed using the Friedman test. When a significant difference was found, post hoc pairwise comparisons were performed using the Wilcoxon signed-rank test with Bonferroni correction. A *p*-value < 0.05 was considered statistically significant.

Inter-rater reliability was evaluated using intraclass correlation coefficients (ICC) based on a two-way random-effects model with absolute agreement (ICC [2, 1]). ICC values were interpreted according to Koo and Li as poor (<0.50), moderate (0.50–0.75), good (0.75–0.90), or excellent (>0.90) [16].

Spearman’s rank correlation coefficient (ρ) was used to examine the relationships between readability (FRE), accuracy, mDISCERN, and GQS scores. Correlation strength was interpreted as negligible (<0.20), weak (0.20–0.39), moderate (0.40–0.59), strong (0.60–0.79), or very strong (≥0.80) [17]. These analyses were exploratory and aimed to examine associations among response performance measures, particularly whether readability was associated with accuracy, overall quality, and information reliability.

Model comparisons were performed separately for each clinical domain. Correlation analyses were performed using all 65 questions for each LLM.

## 3. Results

A total of 65 questions were submitted to ChatGPT 5.2, Gemini 3, and DeepSeek V3.2. Model performance was evaluated separately in the Medical and Safety Domain (*n* = 28) and the Rehabilitation Domain (*n* = 37).

### 3.1. Domain-Based Comparisons

In the Medical and Safety Domain, significant differences were found among the models for accuracy (*p* < 0.001), quality (GQS; *p* < 0.001), information reliability (mDISCERN; *p* = 0.004), and readability (FRE; *p* = 0.011). For accuracy and GQS, both ChatGPT 5.2 and Gemini 3 had significantly higher scores than DeepSeek V3.2, with no significant difference between ChatGPT 5.2 and Gemini 3. For mDISCERN, DeepSeek V3.2 had significantly higher scores than ChatGPT 5.2 (Bonferroni-adjusted *p* = 0.024), whereas Gemini 3 did not differ significantly from either model. Despite identical median scores, DeepSeek V3.2 scored higher on seven paired responses, while the remaining 21 received identical scores. Regarding readability, Gemini 3 demonstrated significantly higher FRE scores than DeepSeek V3.2 (Bonferroni-adjusted *p* = 0.006), whereas ChatGPT 5.2 did not differ significantly from either model (Table 1).

In the Rehabilitation Domain, no significant differences were found among the models for accuracy, quality (GQS), or information reliability (mDISCERN) (all *p* > 0.05). Readability differed significantly among the models (FRE; *p* < 0.001), with significantly higher FRE scores for ChatGPT 5.2 and Gemini 3 than for DeepSeek V3.2 (both Bonferroni-adjusted *p* < 0.003). No significant difference in readability was observed between ChatGPT 5.2 and Gemini 3 (Table 1).

### 3.2. Inter-Rater Reliability

Inter-rater agreement ranged from moderate to excellent across the three models and both clinical domains (Table 2). In the Medical and Safety Domain, ICC values ranged from 0.530 to 0.851. In the Rehabilitation Domain, ICC values ranged from 0.678 to 0.901. Disagreements between the two independent raters occurred in 89 of 585 paired rating comparisons (15.2%) across the three models and scoring instruments.

### 3.3. Correlation Analysis

Spearman correlation analysis showed varying associations among the evaluation measures. For ChatGPT 5.2, significant positive correlations were found between accuracy and GQS (ρ = 0.446, *p* < 0.001), accuracy and mDISCERN (ρ = 0.342, *p* = 0.005), and GQS and mDISCERN (ρ = 0.361, *p* = 0.003). For Gemini 3, significant positive correlations were found between accuracy and GQS (ρ = 0.422, *p* < 0.001), accuracy and mDISCERN (ρ = 0.431, *p* < 0.001), and GQS and mDISCERN (ρ = 0.457, *p* < 0.001). For DeepSeek V3.2, a significant positive correlation was found between accuracy and GQS (ρ = 0.578, *p* < 0.001), whereas correlations between accuracy and mDISCERN (ρ = −0.048, *p* = 0.702) and between GQS and mDISCERN (ρ = −0.224, *p* = 0.073) were not statistically significant.

Correlations involving FRE were weak or negligible and were not statistically significant for any of the three models. For ChatGPT 5.2, FRE was correlated with accuracy (ρ = 0.061, *p* = 0.627), GQS (ρ = −0.118, *p* = 0.351), and mDISCERN (ρ = 0.177, *p* = 0.157). For Gemini 3, the corresponding correlations were ρ = 0.134 (*p* = 0.286), ρ = 0.042 (*p* = 0.741), and ρ = 0.200 (*p* = 0.111). For DeepSeek V3.2, they were ρ = −0.056 (*p* = 0.658), ρ = −0.117 (*p* = 0.353), and ρ = 0.182 (*p* = 0.147), respectively.

### 3.4. Retrospective Clinical Safety Assessment

The initial screening of 195 responses identified 18 responses with potential clinical safety concerns. Following expert review of these flagged responses, 10 responses (5.1%) were classified as containing clinically important errors or omissions, and 7 responses (3.6%) contained unsupported or overly definitive clinical recommendations. All seven responses in the second category were also classified as containing clinically important errors or omissions.

In the Medical and Safety Domain, clinically important errors or omissions were identified in three responses, compared with seven responses in the Rehabilitation Domain. DeepSeek V3.2 accounted for seven of the ten responses containing clinically important errors or omissions, including six in the Rehabilitation Domain (Table 3).

Examples of clinically important errors and unsupported recommendations were identified in both domains. In the Medical and Safety Domain, Gemini 3 (Question 27) provided a detailed pain medication schedule, including high-dose ibuprofen information and alternating analgesic use, without adequately addressing individual contraindications and medication-related risks. In the Rehabilitation Domain, DeepSeek V3.2 (Question 61) made an unsupported claim that 90% of recurrent dislocations occur when patients ignore movement-related fear. It also recommended specific weight-bearing exercises without adequately considering the patient’s stage of recovery. These examples illustrate how responses may contain useful information while also including potentially misleading or insufficiently individualized recommendations.

## 4. Discussion

The present study evaluated the accuracy, quality, information reliability, and readability of responses generated by three widely used LLMs to questions about first-time traumatic anterior shoulder dislocation. Overall, the models provided generally accurate and high-quality information. In the separate domain-specific analyses, statistically significant differences among models were observed for several outcomes in the Medical and Safety Domain, whereas no statistically significant differences in accuracy, quality, or information reliability were detected in the Rehabilitation Domain. However, these findings do not establish a statistically significant difference in model performance between the two clinical domains. These findings highlight the importance of evaluating LLM performance across different types of clinical questions while avoiding direct conclusions about domain-dependent differences.

One of the main findings was the difference in model performance for medical and safety questions. These questions included acute management, sling use, recurrence risk, imaging, follow-up, and surgical treatment. ChatGPT 5.2 and Gemini 3 had significantly higher accuracy and GQS scores than DeepSeek V3.2, with no significant differences between ChatGPT 5.2 and Gemini 3. For mDISCERN, DeepSeek V3.2 had significantly higher scores than ChatGPT 5.2, whereas Gemini 3 did not differ significantly from either model. The questions covered different clinical themes, and variation in their content, potential complexity, and clinical risk may have influenced the observed patterns of model performance. Medical management questions may require more nuanced clinical decision-making, particularly when recommendations depend on individual patient characteristics, treatment indications, or potential risks. Differences in how LLMs communicate clinical uncertainty, address contraindications, and formulate condition-specific recommendations may have contributed to the observed between-model variability. However, question difficulty was not independently assessed, and these factors cannot be identified as explanations for the observed results. In addition, differences in evaluator backgrounds and scoring tendencies may have contributed to the observed patterns, further limiting direct comparisons between the two domains. Previous research in orthopaedics has also shown differences between LLMs when answering clinical questions [8]. More broadly, LLM performance may vary depending on the clinical topic and the type of information requested [7]. A recent study in sports injury care similarly found differences between GPT-4o and GPT-5 in expert-rated clinical performance, although both models generally showed high clinical adequacy [18]. Differences in LLM performance across clinical scenarios have also been reported in patient-oriented evaluations. Bilgiç and Karacan found that differences among ChatGPT, Gemini, and DeepSeek became more pronounced in clinically significant scenarios, despite generally high overall accuracy [19]. Our findings further support evaluating LLM performance across specific clinical domains rather than assuming similar performance across different types of questions. On the five-point accuracy scale, a score of 5 indicates complete and accurate information, whereas a score of 4 reflects accurate information with minor omissions or unnecessary content. However, even a minor omission may be clinically important if it concerns patient safety or treatment decisions. The observed differences in accuracy may be clinically relevant, particularly when responses concern recurrence risk, imaging, surgery, or urgent assessment. However, the clinical significance of these findings depends on the nature of the information provided and the potential consequences of inaccurate or incomplete recommendations.

An important consideration when interpreting the Medical and Safety Domain findings is the clinical context provided by the standardized prompt. Although the prompt included the phrase “unless otherwise specified in the question,” some questions addressing acute symptoms or emergency management may have been interpreted within the assumed post-reduction scenario. This should be considered when interpreting responses to questions concerning immediate emergency assessment and home monitoring.

In the Rehabilitation Domain, no statistically significant differences were detected among the models for accuracy, quality, or reliability. These questions included daily activities, exercise, rehabilitation progression, strengthening, and return to sport. Notably, the median accuracy and GQS scores were 5 for all three models, suggesting a potential ceiling effect that may have limited the ability of these measures to distinguish between models. Therefore, the absence of statistically significant differences should not be interpreted as evidence of equivalent performance. The non-significant mDISCERN result (*p* = 0.067) should also be interpreted cautiously, as the absence of statistical significance does not demonstrate equivalence among the models. One possible explanation is that many rehabilitation questions involve general guidance on exercise progression and daily activities rather than acute medical decision-making. Rehabilitation and return to sport are important components of recovery after anterior shoulder instability [2]. A recent study showed that LLMs can generate structured exercise prescriptions for rheumatological conditions, although performance differed between models [20]. Similarly, Fu et al. reported moderate-to-high quality in LLM-generated exercise rehabilitation programs across several musculoskeletal disorders [21]. This difference may reflect the different models and tasks evaluated, as our study focused on common patient questions rather than structured exercise prescription.

Inter-rater agreement was generally moderate to excellent across both domains. However, some ICC estimates had relatively wide 95% confidence intervals, indicating uncertainty in the estimated agreement. Therefore, these findings should be interpreted with caution.

The retrospective clinical safety assessment provided additional information beyond the overall performance scores. Although no significant differences in accuracy, GQS, or mDISCERN were found among the models in the Rehabilitation Domain, the safety assessment identified clinically important concerns in some responses. These included potentially unsafe activity recommendations and provocative shoulder testing. Concerns related to neurological symptoms, medication advice, and overly definitive clinical recommendations were also identified. These findings suggest that high overall performance scores do not necessarily indicate the absence of clinically important errors. Therefore, accuracy, GQS, and mDISCERN should not be considered direct measures of clinical safety.

Readability was another important finding. Significant differences in FRE scores were observed among the models in both clinical domains. In the Medical and Safety Domain, Gemini 3 demonstrated significantly higher readability scores than DeepSeek V3.2, whereas ChatGPT 5.2 did not differ significantly from either model. In the Rehabilitation Domain, both ChatGPT 5.2 and Gemini 3 had significantly higher readability scores than DeepSeek V3.2. Nevertheless, median FRE scores remained within the fairly difficult reading range across all three models. Differences in sentence structure, technical terminology, and the amount of explanatory detail may have contributed to the observed variation in FRE scores. Furthermore, the use of clinically necessary medical terminology may lower FRE scores even when the information is accurate and relevant to the patient’s condition. This suggests that clinically useful information is not necessarily easy to read. Previous research has also identified readability as an important limitation of AI-generated health information [9]. These observations are consistent with findings from recent musculoskeletal studies. Fu et al. found that LLM-generated rehabilitation programs exceeded recommended patient reading levels across multiple readability indices [21], while Polat et al. reported that responses to patellofemoral pain questions also exceeded commonly recommended reading levels for patient education [22]. In our study, correlations between readability and accuracy, quality, and information reliability were weak and not statistically significant across all three models. Thus, responses with higher FRE scores were not necessarily more accurate or reliable. Clinical performance and readability should therefore be considered as separate but complementary aspects when evaluating LLM-generated health information. Correlations among accuracy, GQS, and mDISCERN should be interpreted cautiously because these measures assess partially overlapping constructs. Furthermore, the correlation analyses combined questions from two clinical domains evaluated by different expert groups, which may have influenced the observed associations. Importantly, FRE assesses textual readability rather than actual patient comprehension, which may not be adequately captured by readability formulas.

These findings have practical implications for the use of LLMs in patient health information. Patients with a first-time shoulder dislocation may seek information about recurrence, imaging, treatment, exercise, and return to sport. LLMs can provide rapid and accessible answers to many of these questions. However, the differences observed in the Medical and Safety Domain suggest that greater attention is warranted when questions involve acute management, recurrence, imaging, treatment decisions, or other safety-related issues. Current reviews of LLMs in healthcare similarly emphasize their potential to improve access to health information while noting limitations related to accuracy, reliability, and clinical interpretation [10]. In a recent musculoskeletal patient-education study, Doran found that ChatGPT generated generally acceptable and understandable responses to scoliosis-related questions, while emphasizing that AI-generated information should remain supportive and subject to clinical oversight [23]. LLM-generated information may therefore support patient education, but questions involving medical decisions and safety still require appropriate clinical assessment.

This study has several strengths. First, three widely used LLMs were evaluated using the same standardized prompt and the same set of questions. Second, the questions covered both medical and rehabilitation topics, allowing model performance to be evaluated separately within two clinical domains. Third, responses were independently evaluated by orthopedic surgeons and physiotherapists with relevant clinical expertise. Finally, multiple measures were used to assess accuracy, reliability, quality, and readability, providing a broader evaluation of model performance than a single outcome measure. The additional retrospective clinical safety assessment also provided a more clinically focused evaluation of potentially consequential errors in the generated responses.

Several limitations should be considered. First, LLMs are continuously updated; therefore, the findings reflect model performance during the study period. Second, expert evaluations involve subjective judgment, and the participation of the same experts in question development and response evaluation may have introduced expectation bias. To mitigate this risk, model identities were anonymized, responses were randomized, and evaluations were conducted independently before consensus scoring. Moreover, the two clinical domains involved different question sets and evaluator groups, and question difficulty was not independently assessed. These factors limit direct comparisons between domains, and the findings should not be interpreted as evidence of a statistically established domain effect. Third, the study used a single standardized prompt and one response per model for each question, without assessing variability across repeated generations. Although the prompt specified “unless otherwise specified in the question,” some acute management questions may not have explicitly distinguished between pre-reduction and post-reduction scenarios, potentially influencing model responses. Fourth, FRE assessed textual readability rather than actual patient comprehension. Finally, the findings may not be generalizable beyond first-time traumatic anterior shoulder dislocation. Future studies should investigate alternative prompting strategies, repeated response generation, and additional clinical conditions.

## 5. Conclusions

Large language models provided generally accurate and high-quality information about first-time traumatic anterior shoulder dislocation. In the Medical and Safety Domain, significant differences among models were observed in accuracy, quality, reliability, and readability. In contrast, no statistically significant differences in accuracy, quality, or information reliability were detected in the Rehabilitation Domain, although a potential ceiling effect may have limited the ability of these measures to distinguish between models. However, significant differences in readability were observed in both domains. These findings represent domain-specific observations rather than evidence of a statistically established difference between clinical domains. Readability remained relatively difficult across the models and showed limited associations with clinical performance. LLM-generated information may support patient education, but appropriate clinical oversight remains important, particularly for questions involving medical decisions and safety.

## Figures and Tables

**Table 1:** Comparison of large language model performance in the Medical and Safety and Rehabilitation domains.

Domain/Score	ChatGPT 5.2 Median (IQR)	Gemini 3 Median (IQR)	DeepSeek V3.2 Median (IQR)	χ^2^	Kendall’s W	*p*
Medical and Safety Domain						
Accuracy score	5.0 (5.0–5.0) ^a^	5.0 (5.0–5.0) ^a^	5.0 (4.0–5.0) ^b^	19.500	0.348	<0.001
GQS score	5.0 (5.0–5.0) ^a^	5.0 (5.0–5.0) ^a^	4.0 (4.0–5.0) ^b^	30.471	0.544	<0.001
mDISCERN scale	4.0 (4.0–4.0) ^a^	4.0 (4.0–4.0) ^ab^	4.0 (4.0–5.0) ^b^	11.143	0.199	0.004
FRE score	53.5 (43.0–58.0) ^ab^	56.0 (48.0–59.0) ^a^	48.0 (40.8–51.0) ^b^	9.018	0.161	0.011
Rehabilitation Domain						
Accuracy score	5.0 (4.0–5.0) ^a^	5.0 (4.0–5.0) ^a^	5.0 (4.0–5.0) ^a^	1.368	0.018	0.504
GQS score	5.0 (5.0–5.0) ^a^	5.0 (4.0–5.0) ^a^	5.0 (5.0–5.0) ^a^	2.333	0.032	0.311
mDISCERN scale	4.0 (4.0–4.0) ^a^	4.0 (3.0–4.0) ^a^	4.0 (3.0–4.0) ^a^	5.405	0.073	0.067
FRE score	53.3 (50.2–60.0) ^a^	55.0 (48.0–58.0) ^a^	48.0 (41.0–51.0) ^b^	21.075	0.285	<0.001

GQS: Global Quality Scale; mDISCERN: modified DISCERN; FRE: Flesch Reading Ease; IQR: interquartile range. Data are presented as median (IQR). Models that do not share a superscript letter differ significantly in pairwise comparisons (Bonferroni-adjusted *p* < 0.05). Pairwise comparisons were performed using the Wilcoxon signed-rank test with Bonferroni correction. Adjusted *p*-values are reported in the following order: ChatGPT 5.2 vs. Gemini 3; ChatGPT 5.2 vs. DeepSeek V3.2; Gemini 3 vs. DeepSeek V3.2. Medical and Safety Domain: Accuracy (0.249, 0.003, 0.009); GQS (0.471, <0.003, <0.003); mDISCERN (0.471, 0.024, 0.075); FRE (0.525, 0.321, 0.006). Rehabilitation Domain: Accuracy (0.855, 1.000, 1.000); GQS (0.855, 1.000, 0.288); mDISCERN (0.105, 0.771, 0.618); FRE (1.000, <0.003, <0.003).

**Table 2:** Inter-rater reliability of evaluation scores.

Domain/Score	ChatGPT 5.2, ICC (95% CI)	Gemini 3, ICC (95% CI)	DeepSeek V3.2, ICC (95% CI)
Rehabilitation Domain (*n* = 37)			
Accuracy score	0.778 (0.608–0.879)	0.901 (0.816–0.948)	0.898 (0.581–0.946)
mDISCERN score	0.751 (0.552–0.862)	0.809 (0.661–0.896)	0.807 (0.658–0.896)
GQS score	0.834 (0.424–0.904)	0.799 (0.646–0.891)	0.678 (0.460–0.819)
Medical and Safety Domain (*n* = 28)			
Accuracy score	0.765 (0.557–0.884)	0.664 (0.394–0.829)	0.679 (0.419–0.837)
mDISCERN score	0.791 (0.60–0.897)	0.847 (0.699–0.926)	0.765 (0.554–0.884)
GQS score	0.530 (0.201–0.751)	0.680 (0.414–0.839)	0.851 (0.706–0.928)

GQS: Global Quality Scale; mDISCERN: modified DISCERN; ICC: intraclass correlation coefficient; CI: confidence interval. ICC values were calculated using a two-way random-effects model with absolute agreement (ICC [2, 1]).

**Table 3:** Results of the retrospective clinical safety assessment.

Domain	Model	*n*	Clinical Errors, *n* (%)	Unsupported/Overconfident Claims, *n* (%)
Medical and Safety	ChatGPT 5.2	28	0 (0.0)	0 (0.0)
	Gemini 3	28	2 (7.1)	1 (3.6)
	DeepSeek V3.2	28	1 (3.6)	1 (3.6)
Rehabilitation	ChatGPT 5.2	37	0 (0.0)	0 (0.0)
	Gemini 3	37	1 (2.7)	1 (2.7)
	DeepSeek V3.2	37	6 (16.2)	4 (10.8)
Total	All models	195	10 (5.1)	7 (3.6)

Note: All 195 responses underwent initial screening, and 18 flagged responses underwent further expert review. Clinical errors included clinically important errors or omissions. Unsupported/overconfident claims included unsupported or overly definitive clinical recommendations. Percentages were calculated using the total number of responses for each model and domain.

## Data Availability

The data supporting the findings of this study are available from the corresponding author upon reasonable request.

## Supplementary Materials

The following supporting information can be downloaded at https://www.mdpi.com/article/10.3390/healthcare14213726/s1. Table S1: Questions used to evaluate large language model responses, including clinical domain and thematic classifications; Supplementary File S1: Full responses generated by ChatGPT 5.2, Gemini 3, and DeepSeek V3.2 for all 65 questions; Supplementary File S2: Question-level evaluation scores, including individual ratings provided by two independent evaluators for accuracy, Global Quality Scale (GQS), and modified DISCERN (mDISCERN), and consensus scores used in the main statistical analyses.

## Abbreviations

The following abbreviations are used in this manuscript:

LLM	Large language model
GQS	Global Quality Scale
mDISCERN	modified DISCERN
FRE	Flesch Reading Ease
ICC	Intraclass correlation coefficient
IQR	Interquartile range
MD	Medical and Safety Domain
RD	Rehabilitation Domain
