# Supplementary material for: Large Language Models in Shoulder Dislocation: Performance Across Medical and Rehabilitation Domains
Source: Healthcare (Basel). 2026 Nov 3;14(21):3726. doi: 10.3390/healthcare14213726 (PMC13763467; doi:10.3390/healthcare14213726)
Supplement: Supplementary file 1 [file healthcare-14-03726-s001.zip › File S1.pdf]

	ChatGPT-5.2's Answers	Gemini 3 Answers	DeepSeek's Answers
1	If your shoulder has already been put back in place and you've been checked in the emergency department, then you've already done the right thing.<br><br>For future reference, if your shoulder "comes out" again (a dislocation), **you should go to the emergency room as soon as possible**. Here's why:<br><br>* A dislocated shoulder is very painful and usually won't go back in properly on its own.<br>* Doctors can safely put it back in place (this is called a reduction) using the right technique and pain control.<br>* There's always a risk of associated injuries, such as damage to nerves, blood vessels, or small fractures, which need to be checked with an exam and often an X-ray.<br>* Trying to force it back in yourself can make things worse.<br><br>**There are a few rare exceptions**, like if someone has had multiple dislocations and has been specifically taught by a doctor how to reduce it safely—but even then, medical follow-up is still important.<br><br>**After a first-time dislocation (like yours):**<br><br>* It's normal to have pain and limited movement for a while.<br>* You should follow the instructions given (sling use, rest, and gradual rehab).<br>* A follow-up with an orthopedic specialist or physiotherapist is important to reduce the risk of it happening again.<br><br>**Go back to the emergency room urgently if:**<br><br>* The shoulder looks out of place again<br>* You have severe pain that's not improving<br>* You notice numbness, tingling, or weakness in your arm or hand<br>* Your arm looks pale or feels cold<br><br>If you want, I can also explain how long recovery usually takes and what you can do to prevent another dislocation.	Since your shoulder is currently back in its socket and you have already been cleared by the emergency department, you do not need to go back right now. However, it is very important to understand why people are told to go to the ER **at the moment the dislocation happens.**<br><br>### Why a Dislocation is an Emergency<br>A shoulder dislocation isn't just "out of place"; it is a traumatic event where the ball of the upper arm bone (humerus) is forced out of the socket (glenoid). You should always go to the emergency room immediately if it happens again for several critical reasons:<br><br>* **Muscle Spasms:** The longer the shoulder stays out, the more the surrounding muscles tighten and spasm. This makes it much harder and more painful for a doctor to put it back in later.<br>* **Nerve and Vessel Safety:** While you didn't have nerve damage this time, a bone sitting in the wrong place can put pressure on major nerves or blood vessels. Getting it back in quickly minimizes the risk of permanent damage.<br>* **Professional Reduction:** You should never try to "pop" it back in yourself or have a friend do it. Forceful, untrained maneuvers can cause fractures or tear the labrum (the cartilage ring) even further.<br><br>### When to Go Back (Red Flags)<br>Even though you are home now, you should return to the ER immediately if you notice any of the following "red flag" symptoms during your recovery:<br><br>1. **Numbness or Tingling:** If you feel "pins and needles" or a loss of sensation in your arm or hand.<br>2. **Color Changes:** If your hand or fingers look blue, pale, or feel unusually cold.<br>3. **Severe Swelling:** A sudden, massive increase in swelling or a pulse-like throbbing in the joint.<br>4. **Inability to Move Fingers:** If you lose the ability to wiggle your fingers or grip objects.<br><br>### Your Next Steps<br>Since you are 20 years old, your risk of a repeat dislocation is statistically higher because your tissues are more flexible and you likely lead an active lifestyle.<br><br>* **Follow-up:** Schedule an appointment with an **orthopedic sports medicine specialist**. They will likely order an MRI to check for a "Bankart lesion" (a tear in the labrum) which is very common in first-time dislocations at your age.	Based on your description of a first-time anterior shoulder dislocation that was reduced without complications, you do **not** need to go to the emergency room right away simply because it happened in the past. The emergency room's job was to confirm the joint was back in place and rule out fractures or nerve damage, which they did.<br><br>However, you **do** need urgent follow-up care with an orthopedic specialist. Here is the clear distinction you need to understand right now:<br><br>You should only return to the emergency room **if** you have specific new or worsening problems. Otherwise, this is now managed in a clinic, not the ER.<br><br>### When to Go Back to the ER Immediately<br>Go to the emergency room if you experience any of these three specific changes in the coming days:<br>1. **New numbness or tingling** that spreads down your arm or into your hand/fingers that was not there when you left the hospital.<br>2. **A change in color** where your hand or fingers turn pale, blue, or feel ice cold compared to the other hand.<br>3. **Severe, unrelenting pain** that is not relieved by the sling, ice, or over-the-counter medication prescribed.<br><br>### Why You Don't Need the ER for a Stable Shoulder<br>Since the shoulder is back in the socket and the X-rays were clean, the emergency is over. The treatment from this point forward is **non-emergent** and focused on preventing it from happening again. Going to the ER will result in a long wait only for them to tell you to see a specialist, because ER doctors do not manage long-term recovery or surgical planning.<br><br>### What You *Should* Do Instead (Next Steps)<br>Since you are 20 years old with a first-time dislocation, you are in a critical window for deciding the future health of this shoulder. Here is what you need to do next:<br><br>- **See an Orthopedic Surgeon (Sports Medicine Specialist) within 1-2 weeks.** This is non-negotiable. You need a specialist to examine your stability. They will likely order an **MRI** (not an X-ray). The MRI looks for a tear in the labrum called a **Bankart lesion**. In a 20-year-old athlete, this tear is present over 80-90% of the time, and it makes the shoulder prone to popping out again.<br>- **Keep the arm in the sling.** Follow the discharge instructions regarding sling use. Usually, it's worn for comfort

		* **Immobilization:** Keep your arm in the sling as directed by the ER. Moving it too early—especially reaching overhead or out to the side—can prevent the capsule from healing.<br>* **Physical Therapy:** This is your best defense. Strengthening the rotator cuff muscles will help act as "dynamic ligaments" to hold the joint together since the actual ligaments may now be stretched.	and protection for 1-3 weeks, except when showering or doing elbow exercises.<br>- **Do NOT test it.** Do not try to see "how far you can lift it" or "if it's still loose." You can disrupt the healing of the soft tissues.<br><br>**Summary:** No ER trip needed unless you have nerve/circulation problems. Yes, you need an appointment with an orthopedic surgeon as soon as possible to prevent this from becoming a recurrent problem that requires a bigger surgery later
2	Short answer: **No, you shouldn't try to put your shoulder back in yourself.**<br><br>Even though it might be tempting (especially because it's very painful and feels "out of place"), there are important reasons to avoid doing it:<br><br>* **You could cause more damage.** Without proper technique, you might worsen soft tissue injuries (ligaments, labrum) or even cause a fracture.<br>* **There's a risk to nerves and blood vessels.** These structures can be stretched or compressed during a dislocation, and improper reduction can injure them further.<br>* **It's very painful without proper support.** In the emergency department, doctors often use pain control or muscle relaxation to make the process safer and less traumatic.<br>* **You need imaging afterward.** Even if it "goes back in," you still need an X-ray to confirm proper position and rule out hidden injuries.<br><br>The **safe approach** if it happens again is:<br><br>* Keep your arm still (ideally supported in a sling position)<br>* Avoid forcing any movements<br>* Go to the emergency department as soon as possible<br><br>**Exception:** Some people who have had *repeated dislocations* are taught specific techniques by a doctor to reduce it themselves—but this only applies after medical guidance and training. After a **first-time dislocation like yours**, that doesn't apply.<br><br>If this is something you're worried might happen again (which is more common at your age), I can walk you through prevention strategies and what to expect going forward.	It is very tempting to want to fix the problem yourself—especially if the pain is intense—but the short answer is **no, you should never try to put your shoulder back in place by yourself.**<br><br>While movies often show characters slamming their shoulders against walls or trees, in reality, this is a dangerous move that can turn a straightforward dislocation into a much more serious injury.<br><br>### The Risks of "Self-Reduction"<br><br>* **Fractures:** The shoulder socket is relatively fragile. If you try to force the ball back in at the wrong angle, you can easily chip or break the bone (this is called a **Hill-Sachs** or **Bankart** fracture).<br>* **Tissue Entrapment:** Sometimes, when a shoulder is forced back in incorrectly, soft tissue like tendons or the labrum can get caught inside the joint. This can lock the shoulder and require surgery to fix.<br>* **Nerve and Vessel Damage:** The "brachial plexus" (a major bundle of nerves) and large arteries run right past your shoulder joint. An unguided, forceful pop can pinch or tear these structures, leading to permanent numbness or loss of function in your arm.<br>* **Increased Tearing:** A controlled "reduction" by a doctor uses specific angles to glide the bone back. Doing it yourself usually involves pulling or shoving, which can significantly increase the size of the tear in your labrum or capsule.<br><br>### Why the Hospital is Different<br>When you go to the ER, doctors often use mild sedatives or muscle relaxants. This is key because your muscles naturally seize up to "protect" the injury. By relaxing those muscles, the bone can often slide back in with very little force. They also take X-rays before and after to ensure no bones were broken during the injury or the fix.<br><br>### What to do instead<br>If it happens again:<br>1. **Immobilize:** Use a sling, a folded shirt, or hold your arm against your belly to keep it from moving.	No, you should **never** attempt to put your own shoulder back in place.<br><br>Even though you have experienced a dislocation before and know what it feels like, trying to reduce the joint yourself is extremely dangerous. Here is exactly why this rule applies specifically to you, as a 20-year-old with a previous dislocation:<br><br>### The Immediate Dangers of Self-Reduction<br>- **You Can Cause a Fracture:** The emergency doctor used a controlled, gentle technique based on X-rays that confirmed you had **no fractures**. If you try to yank or force the shoulder yourself, you can easily crack the edge of the socket (glenoid) or snap off a piece of the ball (Hill-Sachs lesion fracture). This turns a 4-week recovery into a major surgical repair with screws.<br>- **You Can Trap or Stretch Nerves:** The nerves that control your arm and hand movement wrap tightly around the shoulder joint. If the ball of the shoulder is stuck in front of the socket, a self-reduction attempt can pinch or stretch the **axillary nerve**. This can cause permanent weakness in your deltoid muscle, making it impossible to lift your arm above your head ever again—even after the shoulder is fixed.<br>- **You Will Likely Fail:** As a first-time dislocator, your muscles are strong and will go into violent spasm to protect the joint. Trying to overcome that spasm without sedation or pain relief will result in excruciating pain and muscle damage, and the shoulder will remain dislocated anyway.<br><br>### The Long-Term Consequence Specific to Your Age<br>You are 20 years old. You have a **Bankart tear** until proven otherwise by an MRI. When the shoulder is out of place, that torn piece of cartilage (labrum) is dangling inside the joint. If you manipulate the shoulder roughly by yourself, you can wedge that cartilage between the bones, creating a "locked" dislocation or tearing it so badly that it becomes irreparable. That changes your surgical outcome from a simple arthroscopic repair to a much more invasive open surgery with a longer recovery.<br><br>### What You Must Do If It Pops Out Again

		2. **Ice:** Apply ice to the area to help with the pain and control swelling.<br>3. **Get Professional Help:** Head straight to the ER or an urgent care center equipped for orthopedic injuries.<br><br>Are you currently wearing a sling, or did the ER give you specific instructions on how long to keep the arm immobilized?	If your shoulder comes out again—whether at home, at the gym, or during sports—this is your plan:<br><br>1. **Stop Moving.** Support the arm in the position of most comfort (usually slightly away from the body with the elbow bent). Do not let anyone try to straighten it or pull on it.<br>2. **Use a Pillow or Towel.** Place something soft between your elbow and your body to take the weight off the joint.<br>3. **Go to the Nearest ER or Urgent Care.** Tell them immediately: *"I have a history of anterior dislocation with a suspected labral tear. It is out. I did not try to reduce it myself."* This information helps them treat you faster and more carefully.<br><br>**Summary:** Self-reduction risks turning a simple dislocation into a broken bone or a paralyzed arm. It requires a trained professional who can assess the direction of the dislocation and apply counter-traction safely. Always seek emergency care for the re-location procedure
3	Yes—but **only gentle, limited movement**, and you should follow the instructions you were given when you left the hospital.<br><br>After a first-time anterior shoulder dislocation:<br><br>* **In the first few days**, your shoulder needs rest. You’ll usually be advised to wear a sling to protect it.<br>* It’s okay to **move your elbow, wrist, and hand** regularly to prevent stiffness.<br>* You can do **small, gentle shoulder movements within a pain-free range** if your doctor said it’s okay—but **don’t force anything**.<br><br>What to avoid:<br><br>* **No lifting, pushing, or pulling**<br>* **No overhead movements**<br>* **No sudden or large shoulder motions**, especially movements that rotate your arm outward (these can stress the healing tissues)<br><br>A helpful rule:<br>If a movement causes **sharp pain or a feeling like the shoulder might slip again**, stop immediately.<br><br>As you recover:<br><br>* Movement is gradually increased over a few weeks<br>* A physiotherapy program is usually recommended to restore strength and stability and reduce the risk of another dislocation	While it might feel okay to move your arm slightly, you need to be extremely cautious. For a first-time dislocation at age 20, the goal is to allow the stretched and torn tissues (the capsule and labrum) to knit back together in the correct position.<br><br>The short answer is: **Only move your arm within the very limited "safe zone" prescribed by your doctor.**<br><br>### The "Danger Zone"<br>The most important rule is to avoid the **"Stop Sign" position**. This is when your arm is out to the side and your hand is raised (abduction and external rotation). This specific movement puts maximum stress on the front of the shoulder—the exact spot where your dislocation occurred—and can easily cause it to pop out again before it has healed.<br><br>### What is Usually Allowed<br>In the first few days to weeks, movement is generally restricted to:<br>* **The Hand, Wrist, and Elbow:** It is actually encouraged to take your arm out of the sling a few times a day to wiggle your fingers, rotate your wrist, and gently straighten your elbow. This prevents those joints from getting stiff and helps with blood flow.<br>* **Pendulum Exercises:** If your doctor cleared them, these involve leaning over a table and letting your arm hang straight down, making tiny, gravity-led circles. This maintains some joint mobility without using the shoulder muscles.<br><br>### The Risks of Moving Too Soon<br>* **Chronic Instability:** If you move your arm too much or too early, the ligaments can heal "long" or loose. This makes your shoulder "sloppy," significantly increasing the chance that it will	Yes, it is okay to move **specific parts** of your arm, but you must be very careful about **which** movements you do. Moving the wrong way too soon is the primary reason a first-time dislocation becomes a recurring problem in a 20-year-old.<br><br>Here is the clear, practical guide for what movement is allowed and what is strictly forbidden right now.<br><br>### What is ALLOWED and Encouraged (Distal Movement)<br>You should move your **elbow, wrist, and fingers** several times a day. This is not only safe, it is necessary to prevent stiffness and swelling.<br><br>- **Pendulum Exercises:** You can lean forward slightly, let the arm hang dead weight like a rope, and use your body momentum to swing the arm gently in small circles. This keeps the shoulder joint fluid moving without using any shoulder muscles.<br>- **Grip Strengthening:** Squeeze a stress ball or a rolled-up sock repeatedly. This pumps blood flow through the arm and prevents the hand from swelling.<br>- **Wrist and Elbow Bending:** You can fully bend and straighten your elbow and wrist while the sling is off, as long as the upper arm stays glued to your side.<br><br>### What is FORBIDDEN (Rotational and Overhead Movement)<br>You must avoid **External Rotation** and **Abduction**. These are the two motions that caused the dislocation in the first place and will almost certainly pop the shoulder out again right now.

	If you’re unsure about what specific exercises are safe at your stage, it’s better to be cautious—I can outline a simple week-by-week idea of what’s typically allowed if you want.	dislocate again during simple activities like reaching for a seatbelt or sleeping.<br>* **Stretching the Scar Tissue:** Your body is currently trying to form a "scar" to plug the gap where the bone ripped through the capsule. Aggressive movement can tear this new, fragile tissue.<br><br>### General Guidelines<br>* **Wear the Sling:** Usually, you’ll be in a sling for **2 to 6 weeks**, depending on your surgeon's preference. It’s your "seatbelt" for the shoulder.<br>* **Sleep in the Sling:** Many people accidentally move their arm into a dangerous position while asleep, so keeping the sling on at night is vital.<br>* **Wait for Physical Therapy:** Do not start "testing" your range of motion or lifting weights until a physical therapist guides you. They will help you regain movement in a way that doesn't risk a re-injury.<br><br>Have they already set you up with a physical therapist, or are you still waiting for a follow-up appointment with an orthopedist?	 - **NO External Rotation:** Do **not** turn your palm forward or reach your hand out to the side as if you were holding a tray or shaking hands with someone standing to your right. This motion (turning the forearm outward away from the body) stresses the torn front of the joint capsule.<br>- **NO Reaching Behind Your Back:** Do not try to tuck in your shirt, reach for your back pocket, or unhook a bra. This position forces the ball of the shoulder forward against the damaged area.<br>- **NO Lifting the Elbow Away from the Body:** Keep the elbow tucked at your side. Do not lift the arm to put on a jacket normally; instead, lean over and let the arm dangle into the sleeve.<br><br>### A Critical Warning for Your Age Group<br>Since you are 20 and this was a sports injury, the labrum (the cartilage bumper in the socket) is likely torn. **You will not feel pain before it pops out again.** Many young patients feel "great" after a few days, take the sling off, and casually reach for the car door or a glass on a high shelf. Because the bumper is torn, the joint slides out **silently and easily**.<br><br>**The Rule of Thumb:** If the movement feels like you are about to throw a ball, swim a stroke, or put on a seatbelt the normal way—**stop**. Use your opposite arm to help position the injured arm.<br><br>Follow this guidance until you see the orthopedic surgeon. They will physically test the shoulder to tell you exactly when it is safe to start moving beyond these limits.
4	Yes—**moving your shoulder too much or too soon can cause problems**, especially after a first-time dislocation.<br><br>When your shoulder dislocated, the stabilizing tissues (like ligaments and the labrum) were stretched or torn. These need time to heal. If you stress them too early:<br><br>* **Higher risk of another dislocation** — the shoulder is more unstable in the early phase<br>* **Delayed healing** — the injured tissues may not heal properly<br>* **Chronic instability** — the shoulder may keep “slipping” or feel unreliable over time<br>* **More pain and inflammation**<br><br>That said, **complete immobility isn’t good either**. If you don’t move at all:<br><br>* Your shoulder can become stiff	Moving your shoulder too soon—or too aggressively—is one of the biggest mistakes you can make during recovery. Because you are 20, your body heals quickly, but it also has a much higher statistical chance of "chronic instability" compared to older patients.<br><br>If you move it too soon, you risk the following specific problems:<br><br>### 1. The "Loose Rubber Band" Effect<br>When you dislocated your shoulder, you stretched and likely tore the **labrum** (the cartilage bumper) and the **capsule** (the connective tissue holding the joint together).<br>* If you move too soon, these tissues heal in a stretched-out, elongated position.<br>* It’s like a rubber band that has been pulled too far; once it’s stretched out, it no longer holds the "ball" of your arm tightly in the "socket."<br><br>### 2. Recurrent Dislocations	Yes, absolutely. Moving your shoulder too soon after a first-time dislocation is the single biggest reason young, active people end up needing surgery later. At 20 years old, the consequences of early movement are more significant than they would be for someone over 60.<br><br>Here is exactly what happens inside the joint and why "feeling okay" is not a reliable guide for moving it.<br><br>### The Specific Damage Caused by Moving Too Soon<br><br>**1. You Prevent the Labrum from Scarring Down (High Recurrence Risk)**<br>When the shoulder dislocates anteriorly (forward), it almost always tears a piece of cartilage off the front of the socket called the **labrum**. This tear is called a Bankart lesion. This piece of cartilage is like a rubber bumper or a wedge that keeps the ball from rolling off the socket.

	* You may develop reduced range of motion (sometimes called a “frozen shoulder”)<br><br>So the goal is **balance**:<br><br>* Protect the shoulder early on (use the sling as advised)<br>* Do **safe, gentle movements** (like elbow, wrist, and hand motion, and limited shoulder movement if allowed)<br>* Gradually increase activity under guidance (often with physiotherapy)<br><br>A simple way to think about it:<br><br>* **Too much too soon = instability risk**<br>* **Too little for too long = stiffness risk**<br><br>If you stick to the plan given by your doctor or physiotherapist, you’ll minimize both risks. If you’d like, I can outline what a typical safe progression looks like over the first few weeks.	This is the most common problem for young athletes. If the initial healing is interrupted by early movement, the joint becomes unstable.<br>* You may find that the shoulder begins to pop out during routine tasks, like putting on a jacket, reaching behind you in the car, or even rolling over in bed.<br>* Every subsequent dislocation causes more damage to the bone and cartilage, making the problem harder to fix later.<br><br>### 3. Increased Risk of Surgery<br>Many first-time dislocations can be managed with physical therapy **if** the tissue is allowed to heal properly at the start.<br>* If you move too soon and the joint becomes permanently loose, physical therapy is much less likely to work.<br>* At that point, surgery to physically "tighten" the joint (often called a Bankart repair) becomes the only way to get back to sports or manual labor.<br><br>### 4. Bone Loss (The Hill-Sachs Lesion)<br>Every time the shoulder slides out of place, the hard edge of the socket can dent the soft ball of the arm bone. This is called a **Hill-Sachs lesion**.<br>* If you move too soon and cause "micro-slips" or another full dislocation, that dent gets deeper.<br>* A large dent makes the shoulder "catch" and slip out even more easily, creating a cycle that can lead to early-onset arthritis.<br><br>### 5. Cartilage Damage<br>Your shoulder is lined with smooth cartilage that allows for fluid movement. Moving a recently injured, unstable joint causes the "ball" to grind against the "socket" incorrectly. This "clunking" wears down the cartilage, which does not grow back.<br><br>---<br><br>**The Bottom Line:**<br>The first **6 weeks** are the most critical for tissue healing. By keeping it immobilized as directed, you are giving the "glue" inside your shoulder time to set.<br><br>Did your doctor mention a specific timeframe for how long they want you to stay in the sling before starting your active exercises?	- **If you rest the arm in the sling:** Scar tissue forms between the torn labrum and the bone. While this scar tissue is not as strong as the original anatomy, it can sometimes create enough of a tether to prevent future dislocations.<br>- **If you move too soon (especially rotating outward):** You pull the torn labrum away from the bone before it has stuck down. If it heals in the **wrong spot** or **doesn't heal at all**, the bumper is gone. Your shoulder will then have a 70-90% chance of dislocating again this year.<br><br>**2. You Worsen the "Dent" in the Bone (Hill-Sachs Lesion)**<br>During the dislocation, the soft ball of your humerus smashed against the hard, sharp edge of your socket. This left a dent or divot in the ball of the bone. This is normal.<br>- **The Problem with Early Movement:** If the soft tissues are loose and you move the arm into a vulnerable position (reaching overhead or behind you), that **dent** will catch on the edge of the socket like a hitch in a gear.<br>- **The Consequence:** Every time that dent catches, it grinds down the edge of the socket. This is called "bone loss." Once a young person loses more than 15-20% of their socket bone, a simple arthroscopic repair (small incisions, quick recovery) is no longer possible. You would require a more complex **Latarjet surgery** (bone graft from another part of your body, longer recovery, more stiffness).<br><br>**3. You Develop a False Sense of Security**<br>The most dangerous period is **Day 5 to Day 14**. The inflammation is down, the pain is mostly gone, and you feel like you can "just do a little." But the deep, stabilizing ligaments are still stretched out like old rubber bands. They are not holding the joint tight.<br>- **What Usually Happens:** You reach for a seatbelt with the injured arm. You prop yourself up in bed with that elbow. You lift a gallon of milk out of the fridge. There is no warning pain—just a sudden, sickening **CLUNK**. And it's out again.<br><br>### What Is the Risk if I Dislocate It Again?<br>If you dislocate this shoulder a second time at your age, the medical recommendation **changes from "possibly conservative care" to "definitive surgical repair."** Almost all orthopedic surgeons will recommend surgery after a second dislocation in a 20-year-old because the odds of a third, fourth, and fifth dislocation approach 100%.<br><br>**Summary:** Moving too soon erases the chance of avoiding surgery. It turns a 4-week recovery into a 6-month surgical recovery. Be patient now so you don't have to be in a sling and rehab for half a year later.

5	After a shoulder dislocation, most pain and stiffness are normal—but there are certain **warning signs** you should take seriously.<br><br>**Go to the emergency room or seek urgent care if you notice:**<br><br>* **Your shoulder looks out of place again**<br>→ This could mean it has dislocated again.<br><br>* **Severe or worsening pain** that isn’t improving with rest and medication<br>→ Could suggest another injury or complication.<br><br>* **Numbness, tingling, or loss of feeling** in your arm, shoulder, or hand<br>→ May indicate nerve involvement.<br><br>* **Weakness**, especially if you can’t lift your wrist or fingers<br>→ Another possible sign of nerve injury.<br><br>* **Your arm or hand becomes pale, cold, or changes color**<br>→ Could mean a problem with blood flow (this is urgent).<br><br>* **Significant swelling or bruising that keeps getting worse**<br>→ Might suggest bleeding or an associated injury.<br><br>**Less urgent, but still important to follow up:**<br><br>* Ongoing feeling that the shoulder is **unstable or “slipping”**<br>* **Limited movement** that isn’t improving over time<br>* Pain that persists beyond a few weeks<br><br>In general, after a first-time dislocation, things should **gradually improve**, not get worse.<br><br>If something feels clearly off or different from what you were told to expect, it’s better to get checked.	While you are currently in the recovery phase, you should always be on the lookout for specific "warning signs." Some of these mean you need to head back to the ER immediately, while others mean you need to speed up your follow-up with a specialist.<br><br>Here is what should worry you, categorized by urgency:<br><br>### 1. Immediate Concerns (Go to the ER Now)<br>These symptoms suggest that a nerve or blood vessel may have been compromised, or that the joint is not sitting correctly:<br>* **Color or Temperature Changes:** If your hand or arm looks blue, purple, or extremely pale, or if it feels cold to the touch compared to your other hand.<br>* **Severe Numbness:** A "dead" feeling in the arm, or being unable to feel a touch on your skin.<br>* **Loss of Pulse:** If you cannot feel a pulse at your wrist.<br>* **Extreme Swelling:** Rapid, massive swelling that makes the skin feel tight or shiny.<br>* **New Weakness:** If you suddenly cannot move your fingers or wrist at all.<br><br>### 2. Signs of "Instability" (Call your Orthopedist)<br>These signs suggest the shoulder isn't healing tightly enough and may be at high risk for coming out again:<br>* **The "Apprehension" Feeling:** A sudden, sharp sense of dread or the feeling that the shoulder is "slipping" when you reach for something.<br>* **Consistent Clicking or Clunking:** While some noise is normal, a painful "clunk" every time you move can indicate a significant labrum tear that isn't sitting right.<br>* **Persistent "Dead Arm":** A sharp, electric-shock-like pain that leaves your arm feeling weak for a few minutes after a certain movement.<br><br>### 3. Signs of Poor Healing<br>* **Frozen Shoulder:** If your shoulder becomes so stiff that you cannot move it even a few inches after the initial inflammatory phase (usually after the first 2-3 weeks).<br>* **Pain that Worsens:** While soreness is expected, pain that gets significantly worse after the first week—especially pain that prevents you from sleeping—should be checked out.<br><br>### 4. Long-Term Warning Signs<br>* **Night Pain:** Pain that keeps you awake months after the injury often points to a rotator cuff issue or chronic inflammation.<br>* **Giving Way:** If the shoulder "gives out" during light activities, like carrying a grocery bag.<br><br>### Why Age Matters Here	That is an excellent question because recognizing a true warning sign early can save you from permanent nerve damage or a much more complicated surgery. After a shoulder dislocation—especially in the days and weeks following the reduction—you should stop thinking about pain and start monitoring for **function and sensation**.<br><br>Here are the specific signs that should worry you and prompt immediate action (a call to your doctor or a return to the ER), separated by urgency.<br><br>### Immediate Red Flags (Go to the ER if You Notice These)<br>These signs suggest nerve or blood vessel compression that was not present when you left the hospital.<br><br>**1. The "Dead Arm" Feeling (Neurological Deficit)**<br>You expect the shoulder to be sore and weak, but you should worry if you experience a **new** onset of:<br>- **Wrist Drop:** You cannot lift your hand up (like revving a motorcycle throttle). Your hand just hangs limp at the wrist.<br>- **Deltoid Paralysis:** You try to lift your arm sideways even one inch and **nothing happens**. You think "lift," but the muscle does not twitch. This suggests injury to the Axillary Nerve, which can happen during the dislocation or if the arm was pulled on.<br>- **Numbness on the Shoulder "Badge":** The skin on the very top outside of your shoulder (where a military patch would go) feels completely numb, like you've been shot with Novocain.<br><br>**2. Vascular Changes (Circulation)**<br>- **Color Change:** Your hand or fingers turn stark white, blotchy purple, or blue compared to the other hand.<br>- **Temperature Change:** The injured hand is ice cold to the touch while the other hand is warm.<br>- **Swelling of the Whole Arm:** Some swelling is normal. However, if the entire arm from armpit to fingers becomes **rock hard, tight, and shiny**, that is not normal.<br><br>**3. Uncontrollable Muscle Spasms**<br>- The muscles in the chest and arm lock up so tightly that you cannot voluntarily relax them, and the pain is significantly worse than when you first dislocated it. This can be a sign the joint is "subluxed" (partially out) or there is significant blood in the joint causing pressure.<br><br>### Red Flags That Require a Call to the Orthopedic Surgeon (Not the ER)

		Because you are **20 years old**, your biggest worry isn't usually "stiffness"—it's **looseness**. Younger patients have a very high rate (up to 70-90% in some studies) of the shoulder coming out a second time if the initial injury isn't managed strictly.<br><br>**Summary Tip:** If you feel like your shoulder is a "loose cannon" that you can't trust during daily movements, that is a major sign that the ligaments haven't healed properly and you need a specialist's opinion.<br><br>Are you feeling any "pins and needles" or numbness in your arm right now, or is it mostly just the dull ache from the injury?	These issues are concerning and mean you need to be seen in the clinic sooner rather than later, but they are not life or limb-threatening emergencies.<br><br>**1. The "Loose" Sensation (Apprehension)**<br>- You should worry if, when you take the arm out of the sling to shower, it feels like the arm is a separate weight hanging from your body by a string. If you feel like one wrong breath will make it pop out, your labrum tear is likely significant and unstable. This is important information for the surgeon planning your care.<br><br>**2. A Hard, Visible Lump in the Front**<br>- Feel the front of your shoulder just inside the armpit crease. If you feel a **hard, bony bump** that does not move when you push on it, and it is in a different spot than your other shoulder, that could be a piece of fractured bone or the ball sitting slightly forward (subluxation).<br><br>**3. Fever or Heat from the Skin**<br>- If the skin over the shoulder becomes red, hot to the touch, and you develop a fever (over 100.4 F / 38 C), that could indicate an infection within the joint. This is rare after a simple reduction, but possible if there was a tiny puncture wound or if the skin was scraped severely during the fall.<br><br>### A Final Important Distinction: Pain vs. Worry<br>You should expect **pain**. That is normal. You should **worry** if the pain is accompanied by a **loss of function** in the hand or fingers, or a change in color.<br><br>**Bottom line:** If your hand works and looks normal, you are likely safe to wait for your specialist appointment. If your hand feels like a heavy, numb, cold block of wood at the end of your arm, that warrants an immediate trip back to the ER.
6	**Mild, temporary numbness or tingling can happen** after a shoulder dislocation—but it's something to pay attention to.<br><br>When your shoulder dislocates, nearby nerves can get **stretched or compressed**, which can cause:<br><br>* Tingling ("pins and needles")<br>* Numbness over part of the shoulder or arm<br>* Sometimes mild weakness<br><br>This often improves after the shoulder is put back in place and settles over time.<br><br>**However, it should not persist or get worse.**	It is **very common** to feel some numbness or tingling immediately after a dislocation, but whether it is "normal" depends on how long it lasts and exactly where you feel it.<br><br>Because the shoulder joint sits right next to the **brachial plexus** (the main highway of nerves for your arm), a dislocation can stretch or "shock" these nerves. Here is a breakdown of what to expect and when to be concerned.<br><br>### Common: The "Axillary Nerve" Stretch<br>The most common nerve affected during an anterior dislocation is the **axillary nerve**.	The short answer is: **Yes, some temporary numbness is common and expected. However, there is a specific type and location of numbness that is a warning sign and needs to be checked.**<br><br>Because you are asking this question a little while after the event, we need to distinguish between "normal post-injury irritation" and "delayed nerve problem."<br><br>Here is exactly how to tell the difference and what to watch for.<br><br>### What is Considered NORMAL (Expected)<br>It is very common to have altered sensation in one specific spot for a few days to a few weeks after an anterior dislocation.

	You should seek medical care **promptly** if:<br><br>* The numbness or tingling **doesn’t go away after reduction**<br>* It lasts more than a short time or is **not improving day by day**<br>* You notice **weakness** (for example, difficulty lifting your arm or moving your hand)<br>* The sensation is **getting worse instead of better**<br><br>A commonly affected nerve is the one that supplies the outer shoulder area, so some decreased sensation there can occur—but it still needs monitoring.<br><br>**Bottom line:**<br><br>* Brief tingling or numbness can be normal<br>* **Persistent, worsening, or associated weakness is not normal and should be checked**<br><br>If you want, you can describe exactly where you feel the numbness, and I can help you understand whether it fits a typical pattern.	* **What it feels like:** Numbness or a "dead" feeling specifically on the **outside of your shoulder** (the "patch" area where a soldier might wear a shoulder rank).<br>* **Why it happens:** This nerve wraps tightly around the bone that popped out. When the bone moves, the nerve gets stretched.<br>* **The Outlook:** In most cases, this is a temporary "bruise" to the nerve (called neurapraxia). It usually resolves on its own as the swelling goes down, but it can take weeks or even months to feel 100% normal again.<br><br>### Common: "Pins and Needles" from Swelling<br>You may feel general tingling in your hand or forearm. This is often caused by:<br>* **Inflammation:** Swelling in the joint can put pressure on the surrounding nerves.<br>* **Sling Position:** Sometimes the sling itself, if it’s too tight or positioned poorly, can press on nerves at the elbow or wrist.<br><br>### When Numbness is NOT Normal (Red Flags)<br>While some tingling is common, you should contact a doctor immediately if you experience:<br>* **Progressive Worsening:** If the numbness was minor at the ER but is now getting worse or spreading.<br>* **Complete Loss of Motor Function:** If you cannot "hitchhike" your thumb, make a fist, or spread your fingers apart.<br>* **Total Numbness:** If you lose feeling entirely in your hand or arm, rather than just having a "fuzzy" sensation.<br>* **The "Wait and See" Rule:** If any numbness or tingling lasts for more than **48 hours** after the shoulder has been put back in, it needs to be evaluated by an orthopedic specialist to ensure there isn't a more significant nerve injury.<br><br>### What you should check right now<br>A simple way to monitor this at home is the **"Sensation Check"**:<br>1. Touch the skin on your thumb, your pinky, and the side of your shoulder.<br>2. Compare the feeling to the other (uninjured) arm.<br>3. If they feel identical, the nerves are likely just irritated. If there is a significant difference, make sure to mention this at your follow-up appointment.<br><br>Are you noticing the tingling in a specific spot, like your fingers, or is it more toward the top of your shoulder?	- **Location:** The numbness is usually over the **outside of the shoulder cap**, roughly where a short sleeve t-shirt ends or a vaccine shot is given. This area is about the size of a silver dollar or an orange.<br>- **Cause:** This is due to the **Axillary Nerve** getting stretched or bruised when the ball of the shoulder popped out the front. The nerve runs around the back of the arm, and when the shoulder dislocates, the nerve gets pulled like a guitar string.<br>- **Sensation:** It feels like that patch of skin is asleep, or like you are touching it through a thick coat.<br>- **Timeline:** This numbness can last for **several weeks** and slowly shrink down to a smaller and smaller spot before disappearing completely. As long as the feeling is just *weird* (not completely absent) and the muscle works, this is a normal part of recovery.<br><br>### What is NOT Normal (Warning Sign)<br>You should be concerned and call your orthopedic surgeon's office **today or tomorrow** if the numbness or tingling has any of these three characteristics:<br><br>**1. It is in the Hand or Fingers, Not the Shoulder**<br>- If you have "pins and needles" in your **thumb, index, or middle finger**, that is not the Axillary Nerve. That is the Median or Musculocutaneous nerve or part of the Brachial Plexus that runs deeper. While this can still be temporary "stretch" injury, it requires a doctor to document it now because it could affect your grip strength later.<br><br>**2. It is Getting WORSE Instead of Better**<br>- It is normal for numbness to be stable or slowly shrinking. It is **not normal** for the numb patch to get bigger, or for tingling to spread down to the elbow when it was previously just at the shoulder. This suggests swelling inside the joint is increasing and putting new pressure on the nerve.<br><br>**3. It is Accompanied by TRUE Weakness (Not Just Pain Avoidance)**<br>- You likely cannot lift the arm because it **hurts** or feels unstable. That's fine.<br>- The test: Can you **shrug** your shoulder up to your ear? (Trapezius nerve). Can you make a tight **fist**? (Hand nerves).<br>- If you cannot do either of those simple things because the muscle simply will not fire, that is more than numbness—that is a motor deficit.<br><br>### The "Pocket" Test for Safety

			Since you are at home without medical equipment, here is a simple way to track this:<br>Put your **good hand** in your pocket and feel the fabric. Now put your **injured side's hand** in the other pocket. Does it feel the same? If you cannot feel the keys or phone in that pocket at all, or if it feels like you're wearing an extra-thick glove, you need to mention this to your doctor.<br><br>**Summary:** Numbness on the **side of the shoulder** is part of the injury. Numbness in the **hand** or **spreading down the arm** is a reason to call the doctor before your scheduled appointment.
7	It depends on the situation—but in general:<br><br>**If your shoulder is currently out of place or you think it has dislocated again, do NOT wait at home.**<br>You should go to the emergency room right away. A dislocated shoulder needs prompt reduction and assessment.<br><br>**If your shoulder has already been put back in place (like in your case), then yes—some pain at home is normal**, and it's reasonable to monitor it as long as:<br><br>* The pain is **gradually improving**, not getting worse<br>* It's manageable with rest, a sling, and pain medication<br>* There are **no warning signs**<br><br>**Do NOT just wait at home—seek care urgently if you notice:**<br><br>* Pain that is **severe or worsening** instead of improving<br>* A feeling that the shoulder has **come out again**<br>* **Numbness, tingling, or weakness** that persists or worsens<br>* Your arm becoming **cold, pale, or discolored**<br><br>**Bottom line:**<br><br>* After reduction → some pain is expected, and you can monitor it<br>* Suspected dislocation or concerning symptoms → **don't wait, get checked immediately**<br><br>If you're unsure whether what you're feeling is "normal recovery pain" or something more concerning, you can describe it and I'll help you sort it out.	Since your shoulder is **already back in place** and you have been cleared by the ER, it is okay to be at home to manage the initial pain. However, there is a big difference between "waiting for pain to go down" and "waiting to see a doctor."<br><br>Because you are 20 years old, "waiting and seeing" without a professional follow-up is actually one of the riskier things you can do for your long-term health. Here is why:<br><br>### The "False Sense of Security"<br>A few days from now, your pain will likely drop significantly. You might feel like you are "healed" because the ache is gone. **This is a trap.**<br>* The pain goes away much faster than the ligaments heal.<br>* If you assume you're fine and start lifting things or playing sports just because the pain stopped, you are highly likely to dislocate it again.<br>* At your age, a first-time dislocation almost always involves a **labral tear**. This tear doesn't "pain" you once the initial inflammation is gone, but it leaves the joint unstable.<br><br>### When "Wait and See" is Dangerous<br>You should **not** wait at home if you experience:<br>* A return of the "out of place" feeling.<br>* Numbness that isn't going away.<br>* A hand that feels cold or looks a different color.<br>* Pain that is so severe you can't sleep, even with the medication the ER gave you.<br><br>### The Correct Way to "Wait"<br>You should be resting at home, but you must use that time to schedule your next steps. You should see an **orthopedic specialist** within the next **1 to 2 weeks**, regardless of how much your pain has improved.<br><br>**While you are at home, follow the R.I.C.E. method:**	Yes, it is perfectly okay and expected to wait at home for the pain to improve **with one very important condition**: you must be certain that the shoulder is **in the socket** and there are no new "red flag" symptoms.<br><br>Since you are asking this after being discharged, here is the realistic, safe framework for waiting at home versus seeking care.<br><br>### When Waiting at Home is the RIGHT Plan<br>You are safe to manage this at home with ice and rest if:<br><br>- **The Pain is Dull, Achy, and Throbbing:** This is inflammatory pain from stretched ligaments and the joint capsule filling with fluid/blood. It peaks around Day 2 or 3 and then slowly eases.<br>- **Pain is Controlled by Sling + Ice:** The shoulder should feel okay when it is completely still and supported by the sling. If taking the weight off the arm makes the pain drop from a 7 to a 3 on the pain scale, you are on the right track.<br>- **The Shoulder Looks "Round":** Compare your injured shoulder to the uninjured one in a mirror. Does it look the same shape? With an anterior dislocation, the shoulder looks **square** (like an epaulet on a military uniform) and there is a hollow divot under the acromion bone. If your shoulder looks like a smooth, normal curve again, the bone is where it belongs.<br><br>### When Waiting at Home is the WRONG Plan (Do Not Wait)<br>Do **not** adopt a "wait and see" approach if the pain has changed in **quality**. You are not waiting for *pain* to get better; you are waiting for *inflammation* to settle. There is a difference.<br><br>**Go to the ER or Urgent Ortho Care if:**<br>- **The Shoulder Popped Out Again:** This is the most common scenario for a 20-year-old. You took off the sling to change clothes, felt a huge clunk, and now the pain is 10/10

		* **Rest:** Keep the arm in the sling. Do not "test" it to see if it still hurts.<br>* **Ice:** 20 minutes on, 20 minutes off. This is the best way to reduce the deep joint ache.<br>* **Compression:** Your sling acts as your compression; keep it snug but not cutting off circulation.<br>* **Elevation:** When sitting or sleeping, prop your arm up on pillows so it doesn't "drag" on the shoulder socket.<br><br>### Summary<br>It is okay to recover at home, but **do not skip the specialist follow-up** just because the pain gets better. The goal isn't just to stop the pain; it's to prevent your shoulder from ever coming out again.<br><br>How has your pain been since you got home? Is it manageable with the instructions the ER gave you?	**and** the shoulder looks square again. You cannot wait this one out. Every hour the joint is out increases nerve stretch and makes it harder to reduce.<br>- **Pain is Unrelenting and Requires High Doses of Meds:** If you need the maximum dose of painkillers just to keep the arm **still** in the sling, that suggests a large amount of blood in the joint (hemarthrosis) causing pressure. This may need to be drained.<br>- **Muscle Spasms Won't Stop:** If the bicep or pectoral muscle is hard as a rock and twitching uncontrollably for hours.<br><br>### The Critical Advice for "Waiting" at Your Age<br>Since you are 20, the goal of waiting is to get to **your orthopedic clinic appointment**, not to avoid all medical care.<br><br>**You are not waiting for the shoulder to heal itself.** You are waiting for the swelling to go down enough so the doctor can perform a physical exam and an MRI. If you wait 4 weeks and the pain is gone, you might be tempted to skip the specialist visit. **That is a mistake.** A painless shoulder with a torn labrum (Bankart tear) is a ticking time bomb for re-dislocation during sleep or sports.<br><br>**Summary:** Wait at home for the *swelling and soreness* to improve (Days 1-7). Do **not** wait at home if the *position* of the bone feels wrong, or if the pain is *escalating* uncontrollably. Keep the sling on, ice it 3-4 times a day, and keep that orthopedic appointment.
8	Yes—after a first-time shoulder dislocation, there are definitely **things you should avoid** to let it heal properly and reduce the chance of it happening again.<br><br>**In the early phase (first few weeks), avoid:**<br><br>* **Overhead movements**<br>(raising your arm above shoulder level)<br><br>* **Outward rotation of your arm**<br>(for example, the motion of throwing a ball or placing your hand behind your head)<br><br>* **Lifting, pushing, or pulling anything heavy**<br><br>* **Sudden or jerky movements**<br><br>* **Sports or high-risk activities**<br>(especially contact sports or anything involving the arm overhead)	Since your shoulder is back in place but the tissues are still very fragile, your main job right now is to **avoid movements that "leverage" the ball out of the socket.** Here is a list of specific things you should strictly avoid during these early weeks of recovery:<br><br>### 1. Avoid the "Stop Sign" Position<br>This is the single most dangerous movement for your injury. Do not reach your arm out to the side and rotate your hand upward (like you are waving or signaling "stop"). This position pulls the front of the shoulder joint open and is the most common way to cause a **re-dislocation.**<br><br>### 2. No Reaching Behind You<br>Avoid reaching into the backseat of a car, reaching behind your back to tuck in a shirt, or reaching for your wallet in a back pocket. This motion forces the head of the arm bone forward against the newly injured ligaments.<br><br>### 3. No Heavy Lifting or Carrying	Yes. What you avoid in the next four to six weeks is far more important than what you do. At 20 years old, the tissue inside your shoulder is healing in a vulnerable position, and one wrong move can undo all the progress of the sling and rest.<br><br>Here is a plain-language list of specific activities and positions you must avoid, grouped by daily life scenarios. These are the movements that most commonly cause a second dislocation in young patients.<br><br>### The "Danger Zone" Positions (Absolute No)<br>These are the angles where the ball of your shoulder is most likely to slip out of the socket again. Avoid these **even if you are out of the sling** for showering or exercises.<br><br>- **NO External Rotation:** Do **not** turn your palm forward. Imagine holding a tray of food or reaching to shake hands with someone standing to your side. Keep your thumb pointed toward the ceiling or toward your body.<br>- **NO Reaching Behind You:** Do **not** reach for your back pocket to get a wallet or phone. Do **not** reach behind

	* **Taking your arm out of the sling too much**, if you were told to wear one<br>(but still move your elbow, wrist, and hand regularly)<br><br>These positions—especially **arm out to the side and rotated back**—are the ones most likely to cause another anterior dislocation.<br><br>**Also be careful with everyday activities:**<br><br>* Getting dressed (avoid forcing your arm into sleeves)<br>* Reaching behind you (like into a back pocket or car seat)<br>* Sleeping positions (don’t lie on the injured shoulder)<br><br>**What is okay:**<br><br>* Gentle, pain-free movements (if advised)<br>* Moving your elbow, wrist, and hand<br>* Gradually increasing activity as guided by your doctor or physiotherapist<br><br>**Why this matters:**<br>Right now, your shoulder is more unstable because the supporting tissues are healing. Doing the wrong movements too soon can increase the risk of **repeat dislocation**, which is more common in people your age.<br><br>If you want, I can give you a simple “do’s and don’ts” plan for daily activities like sleeping, dressing, and using your phone or computer.	Do not carry anything in the hand of your injured arm—not even a heavy coffee mug or a laptop bag. The weight creates "traction," pulling the arm bone downward and stretching the healing capsule.<br><br>### 4. Don't Lean on Your Elbow<br>Avoid propping yourself up on your elbow while lying in bed or sitting on the couch. This pushes the arm bone upward into the "roof" of the shoulder and can irritate the rotator cuff and the labrum.<br><br>### 5. No "Testing" the Joint<br>It is tempting to move your arm around to see "if it still hurts" or to see how far you can move it. **Don't do this.** Every time you push the range of motion, you risk pulling apart the internal "scab" that is trying to form to stabilize the joint.<br><br>### 6. Avoid Sleeping on the Injured Side<br>Sleeping directly on that shoulder can cut off blood flow to the healing tissues and cause significant pain.<br>* **Pro Tip:** Sleep on your back or the uninjured side, and hug a pillow against your chest to support the weight of the injured arm so it doesn't "fall" forward or backward while you sleep.<br><br>### 7. No Sports or High-Impact Activity<br>Even if you feel "fine" in a week, avoid any activity where you might fall, be bumped, or have to move your arm suddenly. This includes running (the bouncing motion is hard on the shoulder) and certainly any contact sports.<br><br>---<br><br>### Summary of "Safe" vs. "Unsafe"<br>* **Unsafe:** Reaching up, reaching out, or reaching behind.<br>* **Safe:** Keeping your elbow "glued" to your side. Most doctors allow you to move your arm from the elbow down (to type or eat) as long as your upper arm stays tucked against your ribs.<br><br>**Quick Check:** Are you having any trouble performing daily tasks (like dressing or bathing) while keeping your arm immobilized? If you need tips on how to do those things safely, I can help!	to tuck in a shirt or fasten a bra. Use your other hand or lean forward and let the arm dangle to dress.<br>- **NO Overhead Motion:** Do **not** lift the elbow higher than your shoulder. This includes reaching for a glass on a high shelf or trying to wash your opposite armpit with the injured arm.<br><br>### Daily Life Activities to Modify or Avoid Completely<br><br>**1. Dressing and Undressing**<br>- **Avoid:** Putting the injured arm through a sleeve first.<br>**Do this:** Put the injured arm in the sleeve **last** when dressing, and take it out **first** when undressing. Lean forward and let gravity help the arm into the sleeve; do not force the arm backward to find the hole.<br>- **Avoid:** Pulling a tight shirt over your head with both arms.<br>**Do this:** Use button-down shirts or large hoodies for the next few weeks.<br><br>**2. Sleeping**<br>- **Avoid:** Rolling onto the injured side. Even if it feels okay in the moment, the weight of your body pushes the ball of the shoulder forward against the torn area all night. This causes swelling and loosens the repair.<br>- **Avoid:** Sleeping with the arm free at your side. **Do this:** Sleep in the sling if instructed, or sleep on your back with a pillow propped **under** the injured elbow to keep the arm slightly away from the body.<br><br>**3. Carrying and Lifting**<br>- **Avoid:** Carrying **anything** with the injured arm while it is out of the sling. Even a grocery bag weighing 5 pounds pulls the ball of the shoulder down and forward.<br>- **Avoid:** Pushing or pulling doors open with the injured arm.<br>- **Avoid:** Lifting a gallon of milk or a heavy pot with that hand. Use the good arm.<br><br>**4. Personal Hygiene**<br>- **Avoid:** Reaching across your body to wash the opposite armpit. **Do this:** Bend forward at the waist so the arm dangles down. Use that dangling position to swing the arm gently to wash.<br>- **Avoid:** Reaching behind your back to dry off with a towel. **Do this:** Drape the towel over your shoulder and pat dry, or use the good arm.<br><br>**5. Sports and Gym (Crucial for You)**

			- **Avoid:** Push-ups, bench press, or dips. These are the most dangerous exercises for an anterior dislocation because they place the arm in external rotation under load.<br>- **Avoid:** Throwing a ball, swinging a racket, or swimming freestyle. These motions require the exact movement that caused the injury.<br><br>### A Specific Warning for a 20-Year-Old<br>You might feel "back to normal" surprisingly fast—often within 7 to 10 days. This is a **trap**. The pain fibers heal faster than the structural collagen fibers. You will be tempted to take the sling off to carry a backpack, drive a manual car, or help a friend move a couch.<br><br>**Do not let pain be your guide.** Let **time** and your **orthopedic surgeon's exam** be your guide. If you avoid these positions strictly for the first 3-4 weeks, you give that torn labrum the only chance it has to scar down and possibly help you avoid surgery. If you ignore these restrictions, you are almost guaranteed a second dislocation, which means surgery becomes a certainty.
9	Yes—**in most cases, you do need to wear a sling after a first-time shoulder dislocation**, at least for a short period.<br><br>**Why the sling matters:**<br><br>* It **protects the shoulder** while the injured tissues start to heal<br>* It **reduces pain** by limiting movement<br>* It helps prevent positions that could cause the shoulder to **dislocate again**<br><br>**How long to wear it:**<br><br>* Typically **1 to 3 weeks**, depending on your doctor’s advice and your pain level<br>* Younger patients (like you) are often advised to be a bit more cautious early on because the risk of repeat dislocation is higher<br><br>**Important balance:**<br><br>* You **should wear the sling most of the time** in the early phase<br>* But you should still **remove it at times** (as instructed) to:<br><br>* Move your **elbow, wrist, and hand**<br>* Do **gentle exercises** if recommended<br><br>**What not to do:**<br><br>* Don’t ditch the sling too early because you “feel okay”	Yes, wearing the sling is one of the most important things you can do right now. While it might feel like a nuisance or a "fashion statement" you didn't ask for, it serves three critical functions for a 20-year-old’s recovery:<br><br>### 1. It Acts as a "Safety Belt"<br>The sling keeps your arm in a neutral position where the ball of the joint is centered in the socket. This prevents you from making accidental, sudden movements—like reaching out to catch a falling phone or reacting to a trip—that could pull the shoulder right back out of place.<br><br>### 2. It Allows the "Glue" to Set<br>Think of your injured ligaments and labrum like a piece of wood you’ve just applied wood glue to. If you keep moving the pieces, the glue will never dry, and the bond will be weak. The sling holds the tissues still so they can knit back together tightly. If they heal "loose," you may deal with a lifetime of your shoulder slipping out.<br><br>### 3. It Prevents "Muscle Fatigue" Pain<br>Your shoulder muscles (the rotator cuff) are currently exhausted and traumatized. Without a sling, the weight of your arm (which is surprisingly heavy) constantly pulls down on the joint. This "gravity stretch" can cause a deep, throbbing ache and prevent the muscles from recovering.<br><br>### When Can You Take It Off?<br>Usually, you are allowed to remove it for:	Yes, you really do need to wear the sling. This is one of the most common points of frustration for young, active patients because the sling feels restrictive and the arm often stops hurting quickly. However, at 20 years old, wearing the sling as directed is the single most important factor in determining whether this becomes a one-time injury or a recurring problem requiring surgery.<br><br>Let me explain exactly **why** it matters and **how** you should actually wear it, because the "why" is not just about comfort—it is about internal healing.<br><br>### The Purpose of the Sling is NOT Pain Relief<br>Most people think: *"My shoulder doesn't hurt anymore, so I don't need the sling."*<br>**The sling is not a painkiller. It is a positioning splint.**<br><br>When your shoulder dislocated anteriorly (forward), it tore the **inferior glenohumeral ligament** and the **labrum** off the front of the socket. In a 20-year-old, that tissue is like a rubber band that has been ripped off its anchor point on the bone.<br><br>- **Arm at Your Side (Sling Position):** The torn ligament and labrum sit **flush against the bone** where they belong. This allows fibrin and early scar tissue to form between the torn edge and the bone. Think of it as gluing two pieces of paper together and holding them still while the glue dries.

	* Don’t keep it on all the time without any movement at all (that can lead to stiffness)<br><br>**Bottom line:**<br>The sling is a **temporary but important part of recovery**—it protects your shoulder early on, while you gradually transition to movement and strengthening.<br><br>If you’re finding it uncomfortable or inconvenient, I can show you ways to wear it properly and make daily activities easier.	* **Showering:** Keep your arm tucked against your side; don't let it hang completely limp if it feels unstable.<br>* **Elbow/Wrist Exercises:** You should periodically take your arm out to straighten your elbow and move your wrist to prevent those joints from getting stiff.<br>* **Dressing:** Lean forward slightly and let the arm hang naturally while sliding a sleeve on, rather than reaching up into the shirt.<br><br>### How Long?<br>Most orthopedic protocols for a first-time dislocation at your age require **2 to 4 weeks** in a sling. Your specialist will give you a specific timeline based on your X-rays and physical exam.<br><br>**Pro Tip:** If the sling is rubbing your neck, you can wrap a small towel around the strap or ensure the strap is crossing over your shoulder blade rather than directly against your neck.<br><br>Are you finding the sling particularly uncomfortable or painful, or is it just getting in the way of your daily routine?	- **Arm Hanging Free (No Sling):** Gravity pulls the weight of your arm down. This pulls the ball of the shoulder (humerus) **away** from the torn labrum. Every time you take a step, that torn tissue flaps around in the joint fluid. It cannot stick down and heal if it is constantly moving.<br><br>### How Long Do You REALLY Need to Wear It?<br>This is where the advice for a **20-year-old** differs from someone who is 60.<br>- **General Rule:** You need to wear the sling **full-time (including sleeping)** for **at least 2 to 3 weeks** after a first-time dislocation.<br>- **Exceptions (When to Remove It):** You can take the arm out of the sling 3-4 times a day for **hygiene** (showering), **elbow bending** (to prevent stiffness), and **pendulum exercises** (as instructed by your doctor). During these times, the arm should hang like a dead weight; you should **not** use the muscles to move the shoulder.<br>- **The Temptation:** By Day 5, you will feel 80% better. **Do not stop wearing the sling at Day 5.** That is the exact moment most 20-year-olds dislocate again—reaching for a seatbelt or a door handle without the sling on.<br><br>### What Happens if I Don't Wear the Sling?<br>If you discard the sling early, you are essentially signing up for the **"Surgical Pathway."**<br>- **With Sling Compliance:** There is a chance (though not 100%) that the tissue scars down enough to provide stability. Some studies show that in young athletes, immobilization in external rotation *might* help the labrum heal in a better position, but standard internal rotation sling use is still the minimum standard to protect the repair.<br>- **Without Sling Compliance:** The labrum heals in a **medialized** position (too far toward the middle of the body). This leaves the front of the socket completely empty and unprotected. The next time you throw a ball or even roll over in bed, the ball will slide right off the front of the socket.<br><br>### How to Make the Sling More Tolerable<br>Since you're 20 and likely active, the sling can be annoying. Here is how to wear it correctly to get the benefit without the common pitfalls:<br>- **The Pillow Trick:** Place a small, soft towel or piece of foam between your elbow and your ribs **under** the sling. This absorbs sweat and prevents skin irritation.<br>- **Sleep Setup:** Sleep in the sling, but prop a pillow **underneath** the sling/elbow to prevent the weight of the arm from pulling on your neck strap while you sleep.

			- **Wrist Check:** Make sure the sling supports your **wrist**, not just your forearm. If your hand hangs limp out the end, it will swell up.<br><br>**Summary:** Wearing the sling is non-negotiable for the first 2-3 weeks. It is the only way to give the torn cartilage a chance to scar to the bone. If you skip the sling, you are choosing a path that very frequently leads to an operating table for a Bankart repair within the next 12 months. Wear it now so you don't have to wear it for 6 weeks *after* surgery later.
10	For a **first-time anterior shoulder dislocation**, most people are advised to wear a sling for about **1 to 3 weeks**.<br><br>For someone your age (20), doctors often lean toward **closer to 2–3 weeks**, because younger patients have a higher risk of the shoulder dislocating again, so protecting it early is important.<br><br>**That said, the exact timing depends on:**<br><br>* Your **pain level**<br>* How stable your shoulder feels<br>* Your doctor's specific instructions<br><br>**Typical approach:**<br><br>* **First 1–2 weeks:** Wear the sling most of the time (especially when up and moving around or sleeping)<br>* You can take it off briefly for **hygiene and gentle movements** (elbow, wrist, hand, and any exercises you were given)<br>* **After 2–3 weeks:** Gradually reduce sling use as pain improves and you start guided movement/physiotherapy<br><br>**Important points:**<br><br>* Don't stop using the sling too early just because it feels better<br>* But also don't keep it on constantly without any movement—this can lead to stiffness<br>* Follow your doctor or physiotherapist's plan if they gave you one, as that overrides general advice<br><br>**A simple rule:**<br>Use the sling until your pain is clearly improving and you can safely start gentle, controlled movements without feeling like the shoulder might slip.<br><br>If you tell me how many days it's been since your injury, I can give you a more tailored idea of what you should be doing right now.	The exact timing depends on your orthopedic surgeon's preference and the severity of the tear, but for a 20-year-old with a first-time dislocation, the standard timeframe is usually **2 to 4 weeks**.<br><br>While it feels like a long time, here is the general breakdown of why those weeks matter:<br><br>### Week 1: The Protection Phase<br>This is the most critical week. The sling is meant to be worn **nearly 24/7**, including while you sleep. The goal is to keep the joint completely quiet so the initial internal bleeding and swelling can subside.<br><br>### Weeks 2 to 4: The Healing Phase<br>Depending on your doctor's advice, you may start taking the sling off more often while you are sitting safely at home. However, you should still wear it:<br>* **In Public:** It serves as a visual "shield" so people don't bump into you.<br>* **While Sleeping:** To prevent you from rolling onto the arm or reaching overhead in your sleep.<br>* **While Walking:** To prevent your arm from swinging, which creates "traction" on the joint.<br><br>### Why You Can't Just Take It Off Early<br>At your age, your tissues are very elastic. If you stop using the sling after only a few days because the pain is gone, the ligaments may heal in a "stretched" position. This leads to **chronic instability**, where the shoulder feels "loose" or "sloppy" for years to come.<br><br>### What to Watch For<br>If you reach the **4-week mark** and your shoulder feels extremely stiff (you can't move it even if you try), your doctor may have you start physical therapy immediately to prevent "frozen shoulder." On the flip side, if it still feels like it's going to "fall out" of the socket, they may extend the sling time or move toward an MRI.	For a first-time anterior shoulder dislocation without a fracture, the recommended time to keep your arm in a sling is typically **between 1 and 3 weeks**. The goal is to balance initial healing with preventing long-term stiffness.<br><br>Medical guidance varies, but recent practice favors shorter periods of immobilization. Here is a summary of current recommendations from authoritative sources:<br><br>* **Up to 1 week:** Some NHS hospital guidelines now advise using the sling mainly for comfort. They recommend weaning off it within the first week, with two weeks being the expected maximum, as staying in the sling longer has not been shown to reduce the risk of another dislocation .<br>* **1 to 3 weeks:** This is a widely accepted standard timeframe for conservative treatment, supported by reviews of medical literature . A study in the British Medical Journal also notes that immobilizing the shoulder for one week is a common recommendation .<br>* **2 to 3 weeks:** This period is also frequently cited in non-operative management protocols for related shoulder injuries .<br><br>**Important Note on Your Age:**<br>As a 20-year-old, you are in an age group that statistically has a higher risk of the shoulder dislocating again . Because of this, it is absolutely essential that the decision on when to stop using the sling and begin exercises is made in partnership with an orthopedic specialist or physical therapist. This is not a decision to make on your own based on comfort alone. Following a guided rehabilitation plan is the most critical step you can take to regain function and minimize the chance of recurrence.

		**The most common mistake:** Taking the sling off because the pain stopped. Remember, **pain goes away in days, but ligaments take weeks to scar down.**<br><br>Did the emergency department give you a specific date for a follow-up appointment, or are you responsible for finding an orthopedic doctor on your own?	
11	Yes—**taking the sling off too early can cause problems**, especially after a first-time shoulder dislocation.<br><br>The main risk is that your shoulder is still **unstable while the ligaments and capsule are healing**. If you stop using the sling too soon:<br><br>* **Higher chance of re-dislocation**<br>The tissues are still loose and vulnerable in the early weeks.<br><br>* **Slower or incomplete healing**<br>The shoulder may not stabilize properly if it's stressed too early.<br><br>* **More pain and inflammation**<br>Early movement can irritate injured structures.<br><br>This is especially relevant in younger people (like you), because the **risk of repeat dislocation is already higher** after a first event.<br><br>At the same time, wearing it *too long without any movement* can also cause issues like:<br><br>* Shoulder stiffness<br>* Slower recovery of motion<br>So the goal is balance: **protection early, then gradual movement.**<br><br>**Bottom line:**<br>Yes, removing the sling too early can increase the risk of the shoulder coming out again and can affect healing. It's best to follow the timeline given by your doctor or physiotherapist, even if you start feeling better.<br><br>If you want, I can help you judge whether your current stage sounds like "too early" based on how many days it's been and how your shoulder feels now.	Yes, taking the sling off too early is one of the most common reasons young patients end up needing surgery later on. Because you are 20, your body is very good at "turning off" pain, which can trick you into thinking you are healed when your ligaments are actually still very fragile.<br><br>Here are the specific problems caused by ditching the sling too soon:<br><br>### 1. The "Stretched Ligament" Problem<br>Think of the ligaments in your shoulder like a piece of plastic wrap. When you dislocated it, that wrap got stretched and torn.<br>* If you keep the sling on, the edges of the tear stay close together and heal tightly.<br>* If you take it off early, the weight of your arm pulls those edges apart. They will still "heal," but they will heal **long and loose**. This makes your shoulder joint "sloppy," which is the leading cause of repeat dislocations.<br><br>### 2. Recurrent Dislocation (The 90% Risk)<br>Statistically, people in your age group (under 25) have a very high risk of dislocating again. Taking the sling off early significantly increases these odds.<br>* Every time the shoulder comes out, it acts like an ice cream scoop, carving out a bit of the bone or cartilage.<br>* Once you have bone loss from multiple dislocations, a simple sling or physical therapy won't fix it anymore—you would likely need a complex bone-grafting surgery.<br><br>### 3. "Micro-instability" and Pain<br>Even if the shoulder doesn't fully pop out again, taking the sling off too soon can lead to "micro-slips."<br>* This causes the ball to grind unevenly against the socket, leading to chronic inflammation and "tendonitis" that can make it painful to do even simple things like lifting a grocery bag or sleeping on your side.<br><br>### 4. Delayed Return to Sports<br>It feels like taking the sling off early gets you back to your life faster, but it usually does the opposite. If you re-injure the shoulder because it wasn't protected, you have to "reset the clock" back to day one. A few weeks of discipline now can save you from a year of rehab or surgery later.	Yes, taking the sling off too early can definitely cause problems. For a first-time anterior shoulder dislocation, the sling is not just for comfort—it is a critical part of healing the structures that were torn when the ball popped out of the socket.<br><br>Here is exactly why removing it before your doctor says it is safe can lead to issues, and what you need to know about the timeline.<br><br>**The Risk of Recurrent Instability (The Biggest Problem)**<br>When you dislocate your shoulder forward, you almost always tear the labrum. This is a ring of cartilage that acts like a suction cup or bumper to keep the ball in the socket. This specific tear is called a **Bankart lesion**.<br>- **What happens if you remove the sling too soon:** Gravity and the weight of your arm pull down on that fresh tear. Without the sling, the tear is stretched apart instead of being allowed to heal in a tight position.<br>- **The Consequence:** The labrum heals in a **loose, stretched position** rather than a tight, snug position. This significantly increases the chance that your shoulder will dislocate again the next time you throw a ball, reach overhead, or even roll over in bed.<br>- **Important Statistic for a 20-Year-Old:** In patients under 20-25 years old, the re-dislocation rate is already very high (sometimes 70-90%) simply because you are young and active. **Keeping the sling on for the prescribed time is the single best non-surgical thing you can do to lower that percentage.**<br><br>**Risk of Stiffness and Improper Scar Tissue**<br>If the shoulder is not supported, you will naturally start using those muscles to guard against pain (shrugging or holding the arm stiffly). This can cause the muscles to "freeze" in a bad pattern. The sling actually allows the muscles to relax completely, which prevents the rotator cuff from going into spasm.<br><br>**The Importance of the First 1-2 Weeks for Healing**<br>In the first 10-14 days, the body is laying down **collagen scaffolding** in the torn labrum. This is the "glue" for future healing. This process stops if the tissue is constantly moving. Taking the sling off for long periods or doing active arm

		### 5. Increased Muscle Spasms<br>Without the support of the sling, your rotator cuff muscles have to work 24/7 to hold your arm up. They aren't strong enough to do this right after a major injury, so they will eventually fatigue and go into painful spasms, which can actually hurt worse than the original dislocation.<br><br>**The Golden Rule:** Keep the sling on until an orthopedic specialist performs a "stress test" on your shoulder to see if the ligaments have tightened up enough to handle the weight of your arm.<br><br>Are you feeling tempted to take it off because of a specific event coming up, or is it just becoming uncomfortable to wear all day?	movement during this window essentially erases any healing progress made overnight.<br><br>**What *IS* Safe to Do Early?**<br>It is important to distinguish between **taking the sling off** and **moving the elbow/wrist**. You can and should remove the sling briefly (while seated or lying down) to:<br>- Let the elbow straighten fully to prevent elbow stiffness.<br>- Move the wrist and make a fist to keep blood flowing.<br>- Shower (keeping the arm at your side like a dead weight, not lifting it).<br><br>**The Crucial Timeline for a First-Time Dislocation**<br>Since you are 20 with a first-time dislocation, the standard protocol is conservative but strict:<br>- **Weeks 1 to 3:** Sling use **all the time**, except for hygiene and elbow exercises. Removing it to walk around the house or type on a computer with the arm unsupported puts the repair at risk.<br>- **After 3-4 Weeks:** Your doctor will usually clear you to wean out of the sling gradually, but only **after** starting physical therapy.<br><br>**The Bottom Line for You**<br>Because you are young and athletic, the next 3-6 months are a "danger zone" for re-dislocation. **Do not prioritize early comfort over long-term stability.** Follow the sling instructions strictly for the first 3 weeks. If you feel like you "need" to take it off because it's annoying or you feel fine, remind yourself that the **inside** of the joint is still wide open and trying to heal. That healing takes time, and the sling is the cast for the inside of the shoulder.
12	Yes—**in most cases, it is okay to take your arm out of the sling sometimes**, as long as you do it carefully and follow your doctor’s instructions.<br><br>After a shoulder dislocation, the sling is mainly for **protection**, not absolute immobilization.<br><br>**It is usually safe to remove the sling briefly for:**<br><br>* **Moving your elbow, wrist, and fingers** (this is important to prevent stiffness)<br>* **Showering or hygiene**<br>* Doing **gentle, prescribed exercises** (if you were told to)<br>* Short periods of rest when sitting still and your arm is well supported<br><br>**But be careful about:**	Yes, it is actually **recommended** to take your arm out of the sling at specific times, but only for very controlled movements. Keeping your arm "glued" to your side for weeks without any movement at all can lead to extreme stiffness or a condition called "frozen shoulder."<br><br>However, there is a right way and a wrong way to do it.<br><br>### When it is OK to take the arm out:<br>* **Hygiene:** You can remove the sling to shower or wash under your arm. While doing this, keep your injured arm tucked against your side or let it hang straight down. Do not reach up with that hand to wash your hair or reach across your body.<br>* **Elbow, Wrist, and Hand Health:** Every few hours, you should take your arm out of the sling to:<br>* Slowly straighten and bend your elbow.<br>* Rotate your wrist in circles.	Yes, it is okay and actually **necessary** to take your arm out of the sling sometimes—but only under very specific conditions and with very specific movements. The key is understanding the difference between **safe, controlled time out of the sling** versus **risky, unsupported use of the arm**.<br><br>Here is exactly what is safe and what is not safe during the first few weeks of healing.<br><br>**What is SAFE to do out of the sling (You should do this):**<br>You can and should remove the sling 2 to 3 times per day while **seated or lying down with your arm supported**. The purpose of this is to prevent your elbow and wrist from becoming permanently stiff.<br><br>While the sling is off, you can safely do the following "gravity-eliminated" movements:

	* Not letting the arm hang freely for long periods early on<br>* Avoiding sudden movements when the sling is off<br>* Not doing any lifting, pushing, or reaching away from your body<br>* Avoiding positions where the arm is **out to the side or rotated outward**, which can stress the healing shoulder<br><br>**A simple rule:**<br>It’s fine to take it out briefly for necessary and controlled activities, but your shoulder should still feel **supported and protected most of the time** in the early phase.<br><br>If you notice that taking it out causes **sharp pain, a slipping feeling, or instability**, then you should keep it in more consistently and get medical advice.<br><br>If you want, I can explain exactly how to safely move your elbow and shoulder while the sling is off so you don’t accidentally stress it.	* Open and close your fist (squeeze a stress ball if you have one).<br>* **Why?** This prevents your elbow from locking up and helps move blood and fluid out of your hand to prevent swelling.<br>* **Getting Dressed:** You can take the arm out to slide it into a sleeve. The "lean-forward" method is best: lean over slightly and let your arm hang like a pendulum so gravity helps the sleeve slide on, rather than you using your muscles to lift the arm.<br><br>### When it is NOT OK to take the arm out:<br>* **While Sleeping:** You have no control over your movements when you’re asleep. You might roll over or reach behind your head, which could cause a re-dislocation.<br>* **In Crowds or Public:** Always wear the sling when leaving the house. It acts as a "bumper" so people don't accidentally bump into your shoulder or grab your arm.<br>* **When Walking Long Distances:** The swinging motion of walking can irritate the joint. The sling keeps the arm stable.<br><br>### The "Golden Rule" for Slings<br>When the arm is out of the sling, **your upper arm (the humerus) should stay "heavy" and vertical.** Imagine there is a magnet between your elbow and your ribs. You can move your forearm and hand all you want, but your elbow should generally stay close to your side.<br><br>### A Note on "Pendulum" Exercises<br>If your doctor has cleared them, you may take the arm out of the sling to do **pendulum swings**. This involves leaning over a table, letting the arm hang limp toward the floor, and gently swaying your body so the arm moves in tiny circles. This keeps the joint lubricated without using the muscles that were injured.<br><br>**Pro-Tip:** When you take the sling off, does your shoulder feel "heavy" or like it's pulling down uncomfortably? If so, that’s a sign that the tissues aren't ready to support the weight of the arm yet, and you should put the sling back on.	- **Elbow Straightening:** Let the arm hang gently at your side or rest it on a table. Slowly straighten the elbow all the way, then bend it back up. Do not lift the arm away from your body.<br>- **Wrist Circles and Grip:** Make a fist, open your hand wide, and rotate your wrist. This keeps blood flowing and reduces swelling in the fingers.<br>- **Pendulum Swings (Only if cleared by doctor):** Lean forward, let the injured arm dangle like a dead weight toward the floor. Use your body weight to sway gently so the arm swings like a pendulum. **Do not use your shoulder muscles to swing the arm.**<br><br>**What is NOT SAFE to do out of the sling (Avoid this completely for 3 weeks):**<br>This is where the damage happens. When you take the sling off, **gravity pulls the ball of the shoulder down and away from the socket**. This stretches the torn labrum (cartilage) that is trying to heal.<br>- **Carrying the arm at your side while walking:** Even if you aren't "using" the arm, walking around the house with the arm unslung lets the weight of the arm pull on the healing tear. **If you are upright and moving, the sling must be on.**<br>- **Reaching for anything:** Do not take the sling off to reach for a glass of water, type on a keyboard, or open a door with that hand.<br>- **Resting the arm on an armrest:** This pushes the ball of the shoulder upward, which can pinch or disrupt the healing front of the socket.<br><br>**Specific Advice for Sleeping and Showering:**<br>- **Sleeping:** It is generally recommended to **keep the sling on while sleeping** for the first 3 weeks. Rolling onto the shoulder while asleep without the sling is a very common cause of re-dislocation in young people.<br>- **Showering:** You **must** take the sling off to shower. Keep the injured arm hanging straight down like a wet noodle. Do not lift it to wash your hair or scrub your back. Let the good arm do all the work.<br><br>**Summary Rule for the First 3 Weeks:**<br>- **Sling ON:** Standing, Walking, Sleeping, Sitting in a chair without a pillow under the elbow.<br>- **Sling OFF (Briefly):** Sitting with elbow supported on a pillow or table, doing elbow/wrist exercises, showering.<br><br>If you take the arm out just to let it "breathe" but keep it hanging at your side, you are unintentionally undoing the healing that happened overnight. Keep the sling on for all upright activities

			until your doctor or physical therapist specifically tells you it is safe to wean off.
13	No—you should **not keep your shoulder completely still all the time**, even while wearing a sling.<br><br>The sling is meant to **protect your shoulder from risky movements**, not freeze everything.<br><br>**What you should do:**<br><br>* Keep the shoulder **supported and mostly relaxed in the sling** when you're walking around or resting.<br>* But regularly move:<br><br>* **Elbow**<br>* **Wrist**<br>* **Fingers**<br><br>This helps prevent stiffness and circulation problems.<br><br>**Sometimes, and only if your doctor allowed it:**<br><br>* You may do **very gentle shoulder movements within a safe range**, usually small pendulum-type movements or slight guided motion.<br>* But you should **not actively lift, rotate outward, or reach away from your body** in the early phase.<br><br>**What "resting in the sling" should feel like:**<br><br>* Shoulder is protected and not being used for daily activities<br>* Arm is not hanging unsupported<br>* No sudden or forceful movements<br><br>**Important balance:**<br><br>* Too much movement too early → higher risk of re-dislocation<br>* No movement at all → stiffness and slower recovery<br><br>So the goal is **protected rest + safe movement of the lower arm + gradual rehab when advised**.<br><br>If you tell me how many days it's been since your dislocation, I can give you a clearer idea of how much movement is typically appropriate right now.	While the goal of the sling is to keep the **shoulder joint** stable, you definitely should not keep your entire arm and body completely still. Total immobilization can actually lead to secondary problems like blood clots, extreme stiffness, or even a "frozen shoulder."<br><br>Here is how to strike the right balance between "still" and "moving":<br><br>### 1. Keep the Shoulder Joint Still<br>The "ball and socket" part of your shoulder should remain quiet. You want to avoid:<br>* **Shrugging:** Don't use your neck muscles to "hike" the shoulder up toward your ear.<br>* **Reaching:** Don't try to move your upper arm away from your torso.<br>* **Internal/External Rotation:** Don't swing your forearm in and out like a swinging door.<br><br>### 2. Move Your "Periphery" (Hand, Wrist, Elbow)<br>This is actually very important for your recovery. While your shoulder stays tucked in the sling, you should frequently:<br>* **Wiggle your fingers:** Do this throughout the day to keep circulation moving.<br>* **Squeeze a ball:** Gently squeezing a soft stress ball or a rolled-up sock helps maintain forearm strength and pumps swelling out of the arm.<br>* **Rotate your wrist:** You can do this while the arm is still supported by the sling.<br>* **Pump your elbow:** As mentioned before, taking the arm out 3–4 times a day to gently straighten and bend the elbow prevents it from becoming painfully stiff.<br><br>### 3. Move Your Body<br>Don't become a "couch potato" just because your arm is in a sling.<br>* **Walk:** Gentle walking is great for circulation and prevents blood clots. Just be careful not to trip, and avoid power-walking where your body jolts significantly.<br>* **Posture:** Pay attention to your back. People in slings tend to slouch forward and "hunch" to protect the arm. Try to sit up straight and gently squeeze your shoulder blades together occasionally to keep your posture muscles active.<br><br>### 4. Neck Stretches<br>Because the weight of the sling pulls on your neck and trapezius muscle, you might get "tension headaches" or a very stiff neck. Gently tilting your head from side to side (ear to shoulder) can help relieve this tension.	That's an excellent and very common question, because there is a big difference between **immobilizing the shoulder joint** and **letting the whole arm go stiff**. The answer is no—you should **not** keep everything completely still, but you **must** be very specific about *what* you move.<br><br>Here is the clear guidance on what should stay still and what should move while the sling is on.<br><br>**1. The Shoulder Joint Itself: Yes, Keep This Still (Mostly)**<br>While wearing the sling, the goal is to keep the "ball in the socket" quiet. That means **no active lifting, no reaching forward, and no rotating the arm outward** (like turning a doorknob or holding your palm up to the sky).<br>- **Why?** That outward rotation motion is exactly what popped the shoulder out in the first place. Activating those muscles pulls on the fresh tear inside.<br>- **The Rule:** Do not initiate movement from the shoulder. Let the sling do the work of holding the arm up.<br><br>**2. The Rest of the Arm: No, Move This Frequently**<br>If you keep the elbow perfectly bent and the wrist perfectly still for three weeks, you will have a very painful and stiff recovery when the sling comes off. You can do **all of the following exercises** *without taking the sling off* (or by taking it off briefly while seated, as discussed before):<br><br>- **The "Grip and Pump":** Squeeze a stress ball or just make a tight fist, hold for 3 seconds, release. Do this 20-30 times an hour. This is crucial because it acts like a pump to push swelling out of the hand and forearm.<br>- **Wrist Circles:** Rotate your wrist clockwise and counterclockwise.<br>- **Elbow Straightening (While Supported):** When you are seated, you can unclip the sling strap (leaving the forearm cradle on your arm) or remove the sling entirely and rest your forearm on a table. Slide your hand forward on the table to straighten the elbow completely. **This is non-negotiable for preventing elbow stiffness.** Bend it back to 90 degrees and put the sling back on.<br><br>**3. The Hidden Danger: "Protective Shrugging"**<br>This is the most important part of the answer for a 20-year-old patient. Even with the sling on, many people unconsciously keep the shoulder **shrugged up toward the ear** all day because it feels "safer."

		### Summary<br>* **Shoulder:** Still and protected.<br>* **Rest of the arm:** Moving gently to stay healthy.<br><br>If you keep the **shoulder** still but the **hand and wrist** moving, you’ll have a much easier time when you finally start physical therapy.<br><br>Are you noticing any stiffness or "cramping" in your neck or hand since you started wearing the sling?	- **The Problem:** This trains your trapezius and neck muscles to overwork and prevents the rotator cuff muscles from relaxing. If you keep your shoulder shrugged for three weeks, you will have significant neck pain and it will be very hard to lower the arm later.<br>- **The Fix:** Several times a day, consciously tell yourself: **"Drop my shoulder blade down my back."** Let the weight of the arm sink fully into the sling and let the shoulder droop away from your ear.<br><br>**4. The One Small Motion to Avoid Even in the Sling**<br>Even with the sling on, be careful not to let your hand drift backward. If you are standing and you lean forward slightly, the sling can act like a hammock and let the arm swing **backward**. That is the position of instability. Keep the hand in front of your belly button.<br><br>**Summary for Your Recovery:**<br>- **Shoulder:** Still and heavy.<br>- **Elbow:** Straighten it fully once or twice a day (while supported).<br>- **Fingers/Wrist:** Move them constantly.<br>- **Neck/Shoulder Blade:** Relax it down; don't shrug.
14	Yes—**pain after your shoulder has been put back in place is very normal**, especially in the first days to weeks after a dislocation.<br><br>After a dislocation, several things are injured or irritated:<br><br>* The joint capsule and ligaments are **stretched or torn**<br>* Muscles around the shoulder go into **spasm**<br>* The joint lining becomes **inflamed**<br>* Sometimes there is small bone or cartilage bruising even if no fracture was seen<br><br>Because of this, it’s common to have:<br><br>* **Deep aching pain** in the shoulder<br>* Pain when moving the arm<br>* Discomfort at night or when lying on the injured side<br>* Soreness that gradually improves over time<br><br>**What is expected (normal):**<br><br>* Pain that slowly gets better day by day<br>* Mild to moderate pain controlled with rest, sling, and prescribed painkillers<br>* Stiffness as the shoulder protects itself<br><br>**What is NOT normal and should be checked:**	Yes, it is **completely normal** to have pain even after the shoulder is back in the socket. While the "emergency" pain of the bone being out of place usually goes away immediately after the doctor fixes it, you are now dealing with the "injury" pain.<br><br>Think of a shoulder dislocation like a bad ankle sprain, but inside a very complex joint. Even though the bones are aligned, the soft tissues around them have been through a major trauma.<br><br>### Why it still hurts:<br>* **Tissue Tearing:** When the shoulder popped out, it stretched or tore the **labrum** (cartilage) and the **joint capsule**. These tissues are now inflamed and "angry" as they start to heal.<br>* **Bone Bruising:** Often, the ball of the humerus hits the edge of the socket during the dislocation, causing a "bone bruise." Bone bruises can ache for several weeks.<br>* **Muscle Spasms:** Your rotator cuff and large shoulder muscles (like the deltoid and pectoral muscles) often go into a protective "guarding" mode. They stay tight to act as a natural splint, which can cause a dull, heavy ache or sharp cramping.<br>* **Inflammation:** Your body is sending extra blood and fluid to the area to start the repair process. This swelling creates pressure inside the joint, which usually feels like a deep throb.<br><br>### What kind of pain is "normal"?	Yes, experiencing pain after the shoulder has been successfully put back in place is not only normal—it is **expected**. Many patients are surprised by this because they assume once the "pop" is fixed, the pain should disappear. The reality is that the reduction (putting it back) solves the emergency of the dislocation, but it does not instantly heal the injury that caused it.<br><br>Here is exactly why you are still hurting and what kind of pain is considered part of the normal healing process versus a warning sign.<br><br>**Why You Still Have Pain (The Internal Injury)**<br>When the ball of the shoulder comes out of the socket forward, it stretches and tears the ligaments and the labrum (cartilage bumper) at the **front** of the socket. It also bruises the bone at the **back** of the ball (a Hill-Sachs lesion).<br>- **The Timeline:** Immediately after reduction, the body floods the area with inflammation to start healing. This inflammatory phase peaks around **Day 3 to Day 5**.<br>- **What this feels like:** A deep, aching soreness in the front of the shoulder and possibly sharp twinges in the back of the shoulder. You may also feel a heavy, numb ache down the outside of the arm toward the elbow (this is from the axillary nerve being stretched and is very common).<br><br>**What is NORMAL Pain at This Stage:**

	* Pain that is **getting worse instead of better**<br>* A sudden return of severe pain with a “slipping” feeling<br>* Pain with **new numbness, tingling, or weakness**<br>* Severe pain that is not controlled at all with rest and medication<br><br>**Bottom line:**<br>Some pain after reduction is expected and part of healing—but it should **gradually improve over time, not escalate**.<br><br>If you want, you can tell me how strong your pain is (and when it hurts most), and I can help you judge whether it sounds like a normal recovery pattern.	* **A Dull Ache:** Especially at night or when you’ve been standing/walking for a while.<br>* **Soreness to the Touch:** The front of your shoulder will likely be very tender for 1 to 2 weeks.<br>* **Pain with Sudden Movement:** If you sneeze, cough, or move too quickly, you might feel a sharp "reminder" pain.<br><br>### How to manage this "Normal" pain:<br>1. **Ice is your best friend:** Apply ice for 15–20 minutes every few hours. This numbs the nerves and physically shrinks the swelling.<br>2. **Elevation:** When resting, use pillows to prop up your arm so it’s slightly above the level of your heart. This helps the "throbbing" sensation.<br>3. **Medication:** Use whatever anti-inflammatories or pain relievers the ER prescribed or recommended, but stay on a regular schedule for the first few days rather than waiting for the pain to get bad.<br><br>### When is pain NOT normal?<br>While aching is expected, you should be concerned if:<br>* The pain is getting **worse** every day instead of slowly getting better.<br>* The pain is accompanied by a high fever or redness (which could mean infection).<br>* You feel a sharp, electric-like "stabbing" pain that travels all the way down to your fingertips.<br><br>In most cases, the worst of the pain settles down after the first **3 to 5 days**, leaving you with a more manageable soreness.<br><br>Are you having more trouble with a constant dull ache, or is it more of a sharp pain when you try to move?	- **Aching at rest:** A dull, deep ache even when the arm is in the sling and you are watching TV.<br>- **Pain when transitioning:** Sharp, brief pain when you stand up from lying down (gravity pulls on the arm) or when you first put the sling on in the morning.<br>- **Muscle Spasms in the Neck/Upper Back:** Because the shoulder muscles are "offline," the neck and trapezius muscles take over to protect the arm. This causes a knot-like pain above the shoulder blade.<br><br>**What is NOT NORMAL (When to Call Your Doctor):**<br>While some pain is expected, you should be on alert for signs that something has shifted or the nerve is being compressed more than expected.<br>- **Return of the "Dead Arm" Feeling:** If the arm suddenly becomes **more** numb or heavy than it was when you left the ER, or if you cannot move your fingers or wrist at all.<br>- **Pain that Wakes You from Deep Sleep:** A low ache is normal, but a sudden, severe, 10/10 stabbing pain that jolts you awake could indicate the shoulder has partially slipped (subluxed) in your sleep.<br>- **Swelling or Blue Discoloration in the Hand:** The sling can sometimes be adjusted too tightly behind the neck, compressing the veins in the armpit. If your hand looks puffy and purple compared to the other hand, the sling strap needs loosening.<br><br>**How to Manage This Expected Pain Without Overdoing Medication**<br>Since you are 20 and active, you want to minimize heavy painkillers while still being comfortable enough to sleep. Here is the specific protocol for the first 5-7 days:<br>1. **Ice is More Effective Than Heat:** Ice the **back** of the shoulder (the posterior cuff) for 15-20 minutes every 2-3 hours. This is where the bone bruise is located. Do not put ice on the front of the shoulder; it is too sensitive there right now.<br>2. **Pillow Fort for Sleeping:** The worst pain usually comes at night. Sleep in a recliner if possible, or prop **two pillows under the elbow of the injured arm** while you lie on your back. This supports the weight of the arm so the shoulder muscles can finally relax.<br><br>**How Long Will This Last?**<br>- **Days 1-7:** Pain requiring consistent ice and possibly anti-inflammatories (if cleared by your doctor).<br>- **Weeks 2-3:** The sharp, acute pain usually fades into a **positional ache**. You will feel fine sitting still but sore when you move around.<br>- **Important Note:** If the pain **plateaus** and does not improve at all between week 2 and week 4, that is a sign you

			need to discuss it with an orthopedic specialist sooner rather than later. It can sometimes indicate a larger labral tear that is getting caught in the joint.<br><br>In short: **The ache is the sound of your body welding the joint back together.** As long as you can control it with ice and rest, and your hand/fingers are moving fine, it is a normal part of this injury
15	Yes—**swelling and stiffness after your shoulder has been put back in place is very common**, especially after a first-time dislocation.<br><br>After the joint comes out and is reduced, the body reacts with:<br><br>* **Inflammation**, which causes swelling and warmth<br>* **Muscle spasm**, which makes the shoulder feel tight or stiff<br>* **Protective guarding**, where your body limits movement to prevent re-injury<br>* **Soft tissue injury healing**, since ligaments and the joint capsule were stretched or torn<br><br>So it's normal to feel:<br><br>* A **puffy or swollen shoulder area**<br>* **Limited movement or stiffness**<br>* A "tight" feeling when trying to move the arm<br><br>This usually improves gradually over days to a few weeks.<br><br>**What helps (if your doctor hasn't said otherwise):**<br><br>* Keep using the sling as instructed<br>* **Ice packs** (15–20 minutes at a time, several times a day) can reduce swelling and pain<br>* Move your **fingers, wrist, and elbow** regularly<br>* Take prescribed or recommended pain relief if needed<br><br>**What would NOT be normal and should be checked:**<br><br>* Swelling that is rapidly getting worse<br>* Severe pain that is increasing instead of improving<br>* New numbness, tingling, or weakness<br>* The shoulder feeling like it has come out again<br>* Arm becoming cold, pale, or discolored<br><br>**Bottom line:**<br>Mild to moderate swelling and stiffness after reduction is expected—but it should **slowly improve, not worsen**.	Yes, both **swelling and stiffness are completely normal**—and expected—after a shoulder dislocation. In fact, they are part of your body's natural defense mechanism to protect the injured joint.<br><br>Here is a breakdown of why this is happening and what it means for your recovery:<br><br>### Why the Swelling?<br>When your shoulder dislocated, it caused internal "trauma." This likely included tearing of the labrum, stretching of the capsule, and possibly some minor bleeding inside the joint.<br>* **Inflammatory Response:** Your body sends a rush of fluid and white blood cells to the area to start the "cleanup" and repair process. This is what causes the visible puffiness.<br>* **Pressure:** This extra fluid creates pressure inside the joint, which is why your shoulder might feel "full," tight, or warm to the touch.<br><br>### Why the Stiffness?<br>Stiffness after a dislocation usually comes from two places:<br>* **"Muscle Guarding":** Your brain is telling your muscles to stay tight to prevent the arm from moving and dislocating again. This acts like a "natural splint."<br>* **Fluid Friction:** Just like a door hinge with too much gunk in it, the extra inflammatory fluid makes the joint harder to move.<br><br>---<br><br>### How to Manage the Swelling and Stiffness<br>Since you are in the early stages of recovery, your goal isn't to "force" the stiffness away, but to manage it safely:<br><br>* **Ice, Ice, Ice:** Applying ice for **15–20 minutes** several times a day is the most effective way to reduce the "puffiness" and the heat in the joint.<br>* **Gentle Hand/Wrist Movement:** As we discussed, moving your hand and wrist while in the sling helps "pump" the fluid back up the arm and reduces swelling in your lower arm.<br>* **Do Not "Stretch" It Yet:** It is tempting to try and stretch out the stiffness. **Do not do this.** At this stage, stiffness is actually	Yes, this is completely normal and actually an expected part of the healing process for a first-time shoulder dislocation. Many patients are caught off guard by the swelling and stiffness *after* the joint is back in place, so let me explain exactly why this is happening and when you should expect it to improve.<br><br>**Why Swelling Happens After Reduction**<br>When the shoulder dislocates, it causes significant soft tissue trauma. Think of it like a severe ankle sprain, but deep inside the shoulder joint.<br>- **The Inflammatory Response:** The moment the injury occurred, your body sent fluid and blood cells to the area to start repairs. This fluid (swelling) doesn't disappear just because the bone is back in the socket. In fact, it often **peaks 48 to 72 hours after the injury**. Since you are likely in that window right now, the swelling may actually feel worse today than it did in the emergency room.<br>- **Location of Swelling:** You might notice the puffiness is not just on top of the shoulder. It often travels down the arm due to gravity, making the **outer part of the upper arm (deltoid area)** look thicker than the other side. This is normal.<br><br>**Why Stiffness Happens (It's Not Just the Swelling)**<br>The stiffness you feel is a combination of three things:<br>1. **Fluid in the Joint:** The joint capsule is filled with inflammatory fluid, which physically restricts movement—like trying to bend a water balloon.<br>2. **Muscle Guarding:** Even though you are *trying* to relax, the muscles around the shoulder (especially the rotator cuff and deltoid) are in a state of involuntary spasm. This is the brain's way of putting a "cast" on the inside to prevent you from moving it into a dangerous position again.<br>3. **The Sling Itself:** Wearing the sling keeps the arm in one position for hours. This naturally leads to a sensation of "rustiness" when you take it off for elbow exercises.<br><br>**How to Differentiate Normal Stiffness from a Problem**<br>You described a general stiffness. Here is the specific guide to know if it's just normal healing or something needing attention:

	If you want, you can describe how many days it’s been and how severe the swelling is, and I can help you gauge whether it sounds typical for your stage of recovery.	helping keep the joint stable while the tissues are weak. Forcing a stretch could re-tear the healing ligaments.<br><br>### When to Worry<br>While normal swelling and stiffness are expected, you should call your doctor if:<br>* The swelling is so severe that your skin looks shiny or you lose the "nooks and crannies" of your collarbone area.<br>* Your hand or fingers become significantly swollen or turn blue/purple.<br>* The stiffness is accompanied by a fever or the joint feels hot to the touch (which could be a sign of infection).<br><br>### Summary<br>Expect the swelling to peak within the first **3 to 5 days** and then slowly subside. The stiffness will likely last much longer—often until you begin formal physical therapy—but that is actually a good thing for now because it keeps your shoulder "locked" in a safe position while it heals.<br><br>Does the swelling seem to be mostly at the top of the shoulder, or is it traveling down toward your elbow?	- **Normal:** The shoulder feels "frozen" or "locked up" when you try to lift the arm actively. You can only move it a few inches before pain or a block stops you. This is **protective guarding** and is good for healing right now.<br>- **Not Normal (Mechanical Block):** If you feel a hard, bony **clunk** or **catch** that physically stops the arm from moving *even when you are completely relaxed and someone else tries to move it for you*, that needs to be reported to your doctor. This is rare but can indicate a piece of loose cartilage.<br><br>**What to Do About Swelling and Stiffness Right Now**<br>Since you cannot move the shoulder joint itself to "pump out" the swelling (that would risk re-injury), you have to use gravity and the muscles further down the arm.<br><br>1. **Fist Pumps (The Hidden Pump):** While in the sling, aggressively open and close your hand into a tight fist 20-30 times every hour. This squeezes the veins in the forearm and helps pull fluid out of the shoulder and upper arm.<br>2. **Elevation Adjustment:** A sling holds the arm at the belly. To drain swelling, you need the hand higher than the elbow. When sitting, place a **pillow on the armrest of the chair** or on your lap so the hand is propped up higher than the sling strap. This uses gravity to drain the fluid toward the armpit.<br>3. **Ice Placement Matters:** Do not just drape ice over the top of the shoulder. Place the ice pack **under the armpit and on the back of the shoulder blade**. That is where the most swelling collects after an anterior dislocation.<br><br>**Timeline for Relief**<br>- **Swelling:** Should start noticeably decreasing around **Day 5 to Day 7**.<br>- **Stiffness:** Will persist for several **weeks** while the sling is on. The real work of regaining range of motion begins *after* the sling comes off in physical therapy. For now, stiffness is a **protective feature**, not a bug. It is your body's way of ensuring the torn labrum stays still enough to scar down.
16	After a **first-time anterior shoulder dislocation at age 20**, there is a **real risk of it happening again**, but it’s not guaranteed.<br><br>### How likely is recurrence?<br><br>For people in your age group:<br><br>* The risk of another dislocation can be **fairly high (often around 30–60% or more)** over time, especially if you return to sports or overhead activities.<br>* The highest risk period is **the first few months after the injury**, while the ligaments and joint capsule are still healing.	To be candid, because you are **20 years old**, you are in the highest risk category for a repeat dislocation. While it’s not a guarantee, statistics show that young, active patients have a significantly higher chance of the shoulder "popping out" again compared to older adults.<br><br>Here is the breakdown of why your age matters and what determines your risk:<br><br>### The "Age Factor"<br>The younger you are when the first dislocation happens, the more likely it is to recur.	It's completely understandable to be concerned about whether your shoulder might dislocate again. The honest answer is that after a first-time anterior shoulder dislocation, **the risk of recurrence is significant, especially for someone your age.** However, this risk is not random; it's heavily influenced by specific factors, and there are clear steps you can take to manage it.<br><br>### What the Statistics Show for Your Age Group<br>Medical research consistently shows that age is the single most important factor in predicting whether a shoulder will dislocate again. For a 20-year-old active individual, the recurrence rate

	### Why the risk is higher in young people<br><br>* The shoulder structures were **stretched or torn** during the dislocation (often the labrum and capsule)<br>* These tissues may not fully “tighten back” to their original strength<br>* Sports, especially contact or overhead sports, put extra stress on the joint<br><br>### What increases the risk<br><br>* Returning to sport too early<br>* Not doing physiotherapy/rehabilitation<br>* Repeated overhead or contact movements<br>* Previous dislocation (each one increases risk further)<br><br>### What lowers the risk<br><br>* Wearing the sling as advised in the early phase<br>* Completing a proper **rehabilitation/physiotherapy program**<br>* Strengthening the shoulder stabilizer muscles (especially rotator cuff)<br>* Gradual return to sports only when cleared<br><br>### Bottom line<br><br>Yes, your shoulder is **more likely than average to dislocate again**, especially early on—but with proper healing and rehab, many people recover well and avoid recurrence.<br><br>If you want, I can explain the **early warning signs of instability** (feeling like it might “slip out”) and what to do if that happens.	* **For those under age 25:** Some studies suggest the recurrence rate can be as high as **70% to 90%** if not managed strictly.<br>* **Why?** Younger people typically have more flexible "stretchy" ligaments and tend to return to high-impact activities (sports, gym, manual labor) where the shoulder is put under stress.<br><br>### What Increases Your Risk?<br>Several factors determine if your shoulder will stay in place long-term:<br>* **A "Bankart Lesion":** This is a tear in the labrum (the cartilage ring around the socket). In young patients, this tear almost always happens during the first dislocation. If it doesn't heal tightly, the "bumper" that holds the arm bone in place is gone.<br>* **Bone Loss:** If the dislocation "chipped" the edge of the socket or dented the ball of the arm bone, there is less surface area to keep the joint stable.<br>* **Premature Return to Activity:** If you go back to the gym or sports before your rotator cuff is strong enough to compensate for the loose ligaments, the shoulder will likely slip again.<br><br>### How to Lower Your Risk<br>You aren't just a statistic; you can actively lower these odds by following three steps:<br>1. **Immobilization:** Wearing your sling as directed gives the tissues the best chance to "scar down" tightly.<br>2. **High-Quality Physical Therapy:** Since your ligaments are now stretched, you need your **muscles** to do the extra work. A physical therapist will help you build "dynamic stability" by strengthening the rotator cuff.<br>3. **Specialist Consultation:** See an orthopedic sports medicine surgeon. They may order an **MRI** to see the extent of the damage. For a 20-year-old athlete, sometimes a small procedure to "tack" the labrum back down is recommended early to prevent the joint from becoming permanently loose.<br><br>### The "Rule of Threes"<br>Every time the shoulder comes out, it gets easier for it to happen again. Your goal is to make sure this first time is the **only** time. If it happens a second or third time, the damage to the bone and cartilage becomes much harder to fix.<br><br>Are you planning on returning to any specific sports or heavy lifting once you feel better? This can help determine how aggressive your rehab needs to be.	with non-surgical treatment is notably high. Studies indicate that patients under 20 have up to a 90% chance of recurrence, and the rate remains substantial for those in their early twenties . A review of multiple studies found that in the 15-20 age bracket, a significant majority experienced at least one more episode of instability . A landmark study on young athletes (average age of 20) found that **80% of those treated with just a sling and rehab developed recurrent instability** .<br><br>### Why Recurrence is More Likely in Younger Patients<br>The reason for this high rate isn't just about being active; it's anatomical. When your shoulder dislocated, it almost certainly tore the **labrum**, a cartilage bumper that helps hold the ball in the socket. This injury is called a Bankart lesion . In a young, healthy person, the tissues are strong, so the force of the dislocation tends to tear this bumper cleanly from the bone. Because cartilage has poor blood supply, this tear rarely heals back in the correct, tight position on its own. Consequently, the main stabilizer of your shoulder is permanently loose, making it easier for the ball to slip out again.<br><br>### Key Risk Factors That Affect Your Personal Odds<br>While your age is the primary factor, other things can increase or decrease your personal risk:<br>- **Activity Level and Sport Type:** Returning to contact sports (like football or rugby) or overhead sports (like swimming or throwing) places high stress on the healing shoulder and dramatically increases the risk of a repeat injury .<br>- **Bone Damage:** Sometimes, the dislocation chips a piece of bone off the socket (a bony Bankart lesion) or dents the ball (a Hill-Sachs lesion). These bone defects can make the joint even more unstable. If the bone loss is significant, the risk of recurrence is much higher .<br><br>### A Crucial Note on Surgical Consultation<br>Given the statistics for someone your age, the standard of care has evolved. It's now widely recommended that **all patients under 20-25 with a first-time dislocation should be evaluated by an orthopedic surgeon** to discuss surgical options . This doesn't automatically mean you need surgery, but it's a crucial conversation to have early. A study showed that young athletes who had surgery (arthroscopic Bankart repair) after a first dislocation had a recurrence rate of only **14%** , compared to the 80% rate in the non-surgical group .<br><br>### What This Means for Your Recovery<br>Right now, the most important thing you can do is to **strictly follow the non-surgical treatment protocol**. Even if you

			consider surgery later, or if you and your doctor decide against it, the quality of your healing in the first 3-6 weeks is critical.<br>- **Commit to the Sling:** Protecting the injury now gives the soft tissues the best possible chance to scar down, even if that scar isn't as strong as the original bumper.<br>- **Prioritize Physical Therapy:** Once the sling comes off, a dedicated physical therapy program is non-negotiable. The goal is to strengthen the muscles around your shoulder blade and rotator cuff so they can act as dynamic stabilizers, compensating for the torn labrum .<br>- **Have an Honest Conversation with a Specialist:** An orthopedic surgeon can review your specific case, potentially order an MRI to see the extent of the tear, and help you weigh the pros and cons of continued conservative care versus early surgical stabilization. This shared decision-making is the best way to protect your shoulder for the long term.
17	If your shoulder **comes out again (a repeat dislocation)**, it's usually similar to the first time—but there are a few important differences.<br><br>### What you would likely feel<br><br>* Sudden **severe pain** in the shoulder<br>* A feeling that the joint has **slipped or popped out**<br>* **Inability to move the arm normally**<br>* The shoulder may look **visibly deformed or "out of place"**<br>* Possible **numbness or tingling** down the arm<br><br>### What you should do<br><br>* **Go to the emergency department right away**<br>* Keep the arm **still and supported (in a sling position if possible)**<br>* Do **not try to force it back in yourself**<br><br>In the hospital:<br><br>* Doctors will usually perform a **reduction** (putting it back in place)<br>* Pain relief or sedation may be used to relax the muscles<br>* X-rays may be taken again to check for injury<br><br>### Is a second dislocation more serious?<br><br>* It's often **easier to reduce** than the first one<br>* But it is a **sign that the shoulder is unstable**<br>* The risk of **future dislocations becomes higher after each episode**<br><br>### What happens after repeated dislocations	If your shoulder comes out a second time, it's a signal that the joint has become **unstable**. While the immediate treatment is the same—going to the ER to have it put back in—the long-term implications for your health change significantly.<br><br>Here is what happens if you experience a repeat dislocation:<br><br>### 1. Increased Physical Damage<br>Every time the "ball" of your arm bone slides out of the socket, it acts like a chisel. It can cause:<br>* **Bone Loss:** It can wear down the edge of the socket (the glenoid) or create a deeper dent in the arm bone (a Hill-Sachs lesion). Once you lose enough bone, the shoulder becomes like a golf ball trying to sit on a flat tee—it just won't stay put.<br>* **Worse Tearing:** A second dislocation often makes the original labrum tear larger, making a "natural" recovery much less likely.<br><br>### 2. The "Ease" of Dislocation<br>You may notice that while the first time required a major sports collision, the second or third time might happen during a simple move, like reaching for a seatbelt, sneezing, or even rolling over in bed. This is because the "doorway" for the bone to slip through has now been permanently widened.<br><br>### 3. A Shift Toward Surgery<br>After a **first** dislocation at age 20, doctors often try physical therapy first. However, if it happens a **second** time, many orthopedic surgeons will move away from "wait and see" and strongly recommend surgery (usually a labral repair).<br>* **The Logic:** At that point, the ligaments have proven they aren't strong enough to hold the joint, and continuing to let it pop out will only lead to permanent arthritis and bone damage.	That is a very important question to ask, and preparing for that possibility mentally is a smart part of recovery. While the goal is absolutely to prevent a second dislocation, knowing what changes if it *does* happen again will help you understand why the next few months of care are so critical.<br><br>Here is exactly what changes physically, medically, and in terms of treatment options if your shoulder dislocates a second time.<br><br>### 1. The Damage Inside Gets Worse<br>A first-time dislocation usually causes one specific injury pattern: a tear in the front of the labrum (the cartilage bumper) and a dent in the back of the ball (Hill-Sachs lesion). A second dislocation amplifies this damage.<br>- **The Labrum Tear Grows:** The initial tear is like a small rip in a piece of fabric. When the shoulder pops out a second time, that rip often extends further around the rim of the socket. This makes the shoulder progressively looser and more difficult to stabilize without surgery.<br>- **Bone Loss:** With each dislocation, the hard bone of the socket rim can get ground down or chipped off. Once you lose more than about 15-20% of the socket bone, a standard soft tissue repair (Bankart repair) is no longer enough to hold the shoulder in. You would then require a more complex surgery called a **Latarjet procedure**, which involves moving a piece of bone from your shoulder blade to the front of the socket.<br><br>### 2. The "Easy" Reduction May Become Difficult<br>You mentioned your shoulder was put back in successfully in the emergency department. A first-time dislocation often goes back in relatively smoothly (or even pops in on its own).<br>- **With Recurrence:** The muscles around the shoulder learn to spasm **harder and faster** to protect the joint. A second or

If it happens again (especially in young active people), doctors may:

* Recommend more intensive **physiotherapy and strengthening**
* Investigate structural damage (like a labrum tear)
* In some cases, consider **surgical stabilization** if it keeps recurring

Bottom line

If your shoulder comes out again, it is not usually a life-threatening emergency, but it **does require urgent medical care the same day** to safely put it back and reassess stability.

If you want, I can also explain how to tell the difference between **normal post-injury pain vs early warning signs of instability before a full dislocation happens**.

4. Psychological "Apprehension"
One of the biggest impacts of a second dislocation is the loss of trust in your own body. You might start avoiding certain movements, quit sports you love, or feel a constant sense of "dread" that the arm might slip. This is called **shoulder apprehension**, and it can be very limiting for someone your age.

5. Higher Risk of Arthritis
Each dislocation causes "chondral damage"—trauma to the smooth cartilage that lines your joint. Multiple dislocations lead to early-onset osteoarthritis, which can cause chronic pain and stiffness by the time you are in your 30s or 40s.

What you should do if it happens again:
1. **Don't panic, but get to the ER:** Follow the same safety steps as the first time.
2. **Request an MRI:** If you haven't had one yet, a second dislocation makes an MRI essential to see exactly what is broken or torn.
3. **Consult a Sports Specialist:** Ensure you are seeing a surgeon who specializes in shoulder "instability," as they have the most experience in keeping the joint in place for young, active patients.

The Bottom Line: Your goal right now is to be so diligent with your sling and your upcoming physical therapy that a second time **never happens.**

Are you currently worried that the shoulder feels "loose" right now, or does it feel relatively secure in the sling?

third dislocation often requires **conscious sedation** or heavier medication in the ER to relax the muscles enough to get the bone back over the rim. It becomes a more traumatic event for the body each time.

3. The Treatment Recommendation Almost Always Shifts to Surgery
This is the biggest change. For a **first-time** dislocation, it is very reasonable to try a sling and physical therapy (non-operative management) to see if you can avoid surgery.
- **After a Second Dislocation (in a 20-year-old):** The conversation changes from *"Should we consider surgery?"* to *"When should we schedule surgery?"*
- **Why?** The statistics shift dramatically. After a second dislocation in a young patient, the chance of having a **third, fourth, or fifth** dislocation is nearly **90-95%** . The shoulder has proven that it is mechanically unstable. Continuing to let it dislocate only causes more arthritis and bone loss, making a future surgery harder and less successful.

4. The Risk of Nerve or Rotator Cuff Injury Increases
You were fortunate that your first dislocation had no nerve injury. With each subsequent event, the axillary nerve (which powers the deltoid muscle and gives feeling to the skin on the outside of the shoulder) gets stretched again.
- **The Consequence:** You could develop permanent numbness over the "badge" area of the shoulder or, in more severe cases, weakness lifting the arm to the side. While rare, the risk of a rotator cuff tear also increases with each dislocation, even in a 20-year-old.

5. The "Window of Recovery" Shortens
If the shoulder comes out a second time, you are essentially back to Day 0.
- **Healing Reset:** Any healing that occurred in the labrum over the past few weeks is undone. You are looking at another 3-4 weeks in a sling and another 3-6 months of restricted activity.
- **Psychological Impact:** Many patients report that the **fear and anxiety** are much worse the second time. You stop trusting your arm during simple activities like sleeping or reaching for a seatbelt. This can significantly impact your quality of life and sports performance.

What to Do If It Happens Again
If you feel the shoulder slip or come completely out again:
1. **Do NOT let a friend try to pull it or yank it.** Untrained reduction attempts cause fractures.
2. **Keep the arm supported** exactly where it is most comfortable (usually across the belly).

			3. **Go to the ER.** Even if it "pops back in" on its own, you need an X-ray to ensure no new fracture occurred.<br>4. **Follow up with an Orthopedic Surgeon immediately.** Do not pass go, do not wait 4 weeks. At that point, the path forward is likely surgical stabilization.<br><br>**Summary:** If it happens again, it is not the end of the world, but it is a clear signal that the shoulder is not stable enough for your level of activity. It moves the needle decisively from "rehab and hope" to "surgical repair." That is why protecting it now with the sling and strict movement restrictions is so important for the next few weeks.
18	Yes—**your age is one of the strongest factors affecting whether your shoulder dislocates again.**<br><br>At **20 years old**, you are in the **highest-risk age group** for recurrence after a first-time anterior shoulder dislocation.<br><br>### Why age matters<br><br>Younger people tend to have:<br><br>* **More elastic tissues**, which can stretch more during injury<br>* Higher activity levels (sports, overhead movement, contact activity)<br>* Greater stress on the shoulder before full healing is complete<br><br>After a first dislocation, the stabilizing structures (especially the **labrum and joint capsule**) are often injured. In younger patients, these structures are more likely to heal in a way that still leaves some **looseness or instability**.<br><br>### What this means for you<br><br>* People in their teens and early 20s have the **highest recurrence risk**<br>* The risk is **significantly lower in older adults** (because they tend to be less physically active and have different injury patterns)<br>* The chance of recurrence is highest in the **first 6–12 months**, especially if returning to sport too early<br><br>### Important but reassuring point<br><br>Even though the risk is higher at your age, it is **not inevitable**. You can significantly reduce the chance of it happening again by:<br><br>* Wearing the sling as directed in the early phase<br>* Doing proper **physiotherapy and strengthening** (very important)<br>* Avoiding risky positions and early return to sport<br>* Gradually rebuilding shoulder stability	Yes, your age is actually the **most significant factor** in predicting whether your shoulder will dislocate again.<br><br>Because you are **20 years old**, you are in the highest-risk group. While it might seem unfair, younger bodies react differently to this injury than older ones. Here is why your age plays such a huge role:<br><br>### 1. Higher Activity Levels<br>At 20, you are likely more active than a 50 or 60-year-old. Whether it's sports, the gym, or just a busy lifestyle, you are more likely to put your shoulder in "high-demand" positions. The more you use the joint for reaching, lifting, or impact, the more opportunities there are for a loose joint to slip.<br><br>### 2. Tissue Elasticity<br>Younger ligaments and tendons are more "stretchy" (elastic). While this is usually a good thing for avoiding breaks, in a dislocation, it means the joint capsule can stretch out like a balloon that has been over-inflated. Once it's stretched, it doesn't always "snap back" to its original, tight shape, leaving the joint "sloppy."<br><br>### 3. The Statistics<br>The numbers for your age group are quite striking:<br>* **Under Age 25:** Studies show that the risk of a repeat dislocation can be as high as **70% to 90%** if the injury is only treated with a sling and no specific rehabilitation.<br>* **Over Age 40:** The risk drops significantly, often down to **below 10% or 20%**. In older patients, the shoulder tends to get *stiffer* after an injury, which actually helps keep it in place. In younger patients, the shoulder stays *looser*, which makes it easier to pop out again.<br><br>### 4. Labral Tears (Bankart Lesions)<br>In patients your age, a first-time dislocation almost always causes a **Bankart lesion** (a tear in the cartilage ring that deepens the socket). In older patients, the bone might break or the rotator cuff might tear instead. A torn labrum in a 20-year-old is like removing	Yes, absolutely. Your age is actually the single most important factor in predicting the likelihood of your shoulder dislocating again . Because you are 20 years old, your recurrence risk is significantly higher than it would be for someone even just 10 or 15 years older.<br><br>### How Age Impacts Recurrence Rates<br>The primary reason for this is anatomical. A shoulder dislocation in a young person almost always involves tearing the labrum (the cartilage bumper that stabilizes the joint). Because your tissues are strong and healthy, the force of the injury pulls this bumper cleanly off the bone, and due to poor blood supply in that area, it rarely heals back in the correct position by itself . This leaves the shoulder structurally loose. In contrast, dislocations in older adults often involve different injury patterns, like rotator cuff tears or fractures, which behave differently in terms of stability .<br><br>Here is a summary of how the risk changes with age, based on clinical studies of non-surgical management:<br><br>* **Under 20 Years Old:** Recurrence rates can be as high as **70-90%**. One study showed a recurrence rate of **52%**, but other estimates for this age group are even higher . This is the highest risk category.<br><br>* **21-30 Years Old:** The risk remains substantial but begins to decrease. Studies show a recurrence rate closer to **37-41%** in this age bracket .<br><br>* **31-40 Years Old:** The likelihood of the shoulder coming out again drops dramatically. Research indicates a recurrence rate of approximately **21-26%** for this age group .<br><br>* **Over 40-50 Years Old:** Recurrence is much less common, with rates typically below **10-14%** . In this population, the primary concern often shifts from instability to an associated rotator cuff tear .

	### Bottom line<br><br>Yes—being **20 increases the likelihood of recurrence compared with older adults**, but good rehab and protection in the early phase can make a big difference in preventing it.<br><br>If you want, I can explain what a **good recovery timeline and safe return-to-sport plan** usually looks like for someone your age.	the "bumper" that keeps a car on the road—it makes it much easier to slide off again.<br><br>### How to Beat the Odds<br>Even though the statistics seem high, you can change your personal outcome. Your age also means you have an **incredible ability to heal and build muscle.** * **The Strategy:** Since your ligaments (the "tape" holding the joint) are now stretched, you must make your muscles (the "engines" moving the joint) incredibly strong.<br>* **The Result:** If you follow a strict physical therapy program to strengthen your rotator cuff, those muscles can act as "dynamic stabilizers," pulling the ball into the socket and making up for the loose ligaments.<br><br>Because of your age, most doctors will be much more proactive with your care—often suggesting an MRI sooner rather than later to see if you are a candidate for a stabilization procedure before a second dislocation can cause more damage.<br><br>Do you currently play any sports or have a job that requires you to lift your arms over your head frequently?	### What This Means for You<br>These numbers are not meant to be discouraging but to emphasize why the next few weeks of wearing your sling and the following months of physical therapy are so critical. For a 20-year-old, non-surgical treatment can still be successful, but it requires strict adherence to the protocol to give the injured tissues the best possible environment to heal.<br><br>Because of your age and the high statistical risk, it is also why orthopedic specialists often have a detailed conversation with patients like you about the option of early surgical stabilization (repairing the torn labrum). Surgery has been shown to reduce the recurrence rate to around **10-16%** in young, active populations . This is a conversation to have with your surgeon after they have had a chance to examine you and review your imaging. For now, the best thing you can do is protect the healing that is happening by keeping the shoulder immobilized as instructed.
19	You usually can't predict a dislocation with certainty, but there are **warning signs of shoulder instability** that can suggest your shoulder is still at risk.<br><br>### Early warning signs your shoulder may be unstable<br><br>These are the most important things to watch for:<br><br>* **Feeling like the shoulder is "slipping," "shifting," or "about to pop out"**<br>This is one of the strongest signs of instability.<br><br>* **Apprehension in certain positions**<br>Especially when your arm is:<br><br>* Out to the side<br>* Rotated outward (like preparing to throw)<br>If you feel fear, discomfort, or a "danger sensation" in these positions, that matters clinically.<br><br>* **Sharp pain in specific movements**<br>Not general soreness, but a sudden "catching" or sharp pain when moving in certain directions.<br><br>* **Clicking or clunking deep in the shoulder**<br>Especially if it feels like something is moving out of place.<br><br>* **Repeated near-events**	While you can't always predict a sudden accident, there are very specific physical and "gut" feelings that act as early warning signs of an unstable shoulder. Orthopedic specialists actually have a name for the most common sign: **The Apprehension Sign.**<br><br>Here is how you can tell if your shoulder is at high risk of coming out again:<br><br>### 1. The "Apprehension" Feeling<br>This is the most reliable indicator. It is a sudden, overwhelming sense of "dread" or "guarding" when your arm is in certain positions.<br>* **The Test:** If you reach out to the side (like reaching for a seatbelt) or overhead, and you instinctively feel like the joint is about to "slip" or "give way," that is your body telling you the ligaments are too loose to hold the bone in place.<br><br>### 2. "Subluxation" (The Mini-Slip)<br>Sometimes the shoulder doesn't come all the way out, but it "subluxes"—meaning it slides halfway out and then snaps back in.<br>* **What it feels like:** You might feel a sharp, "electric" pain, a sudden weakness in the arm (often called "Dead Arm Syndrome"), or a loud, painful **clunk**. If this happens during normal daily activities, it is a sign that the joint is not stable.<br><br>### 3. Increased "Sloppiness" or Play<br>You might notice that your injured shoulder feels "looser" than your healthy one.	That is a critical question, and learning to recognize the warning signs is one of the most valuable skills you can develop during this recovery. The body often sends very clear signals in the moments or seconds before a dislocation occurs. Knowing these signals can help you freeze, adjust your position, and prevent the joint from slipping all the way out.<br><br>Here are the specific sensations and warning signs to watch for.<br><br>### 1. The "Subluxation" Sensation (The Warning Shot)<br>Often, the shoulder doesn't go from "fine" to "fully out" instantly. It goes through a stage called **subluxation**. This is when the ball slides partially up the rim of the socket but doesn't completely jump over it.<br>- **What it feels like:** A sudden, sickening **"clunk," "slide," or "shift"** deep inside the joint, followed immediately by a millisecond of sharp pain and then a dull ache.<br>- **The Instinct:** Your brain will immediately scream "Something bad just moved!" You will likely catch your breath and freeze.<br>- **Action to Take:** If you feel this clunk/slide, **immediately bring your arm tightly to your stomach and drop your shoulder down away from your ear.** Do not try to shake it out or see "if it's okay." That small slide is a sign the ball is perched precariously on the edge of the socket.<br><br>### 2. The "Apprehension Sign" (Positional Fear)

	Moments where it feels like it almost dislocates but doesn’t fully.<br><br>### What is NOT a reliable sign<br><br>* General soreness or aching pain after injury<br>* Mild stiffness (this is usually normal healing)<br>* Fatigue in the muscles<br><br>### Important context for you<br><br>After a first dislocation:<br><br>* It is **normal to feel cautious or “unstable” for a while**, even if the joint is actually still in place<br>* True instability symptoms usually appear with **specific positions or movements**, not at rest<br><br>### When to get checked<br><br>You should see a doctor or physiotherapist if:<br><br>* You repeatedly feel **slipping or near-dislocation sensations**<br>* You are avoiding normal arm movements because it feels unsafe<br>* Symptoms are not improving over a few weeks of recovery<br><br>### Bottom line<br><br>You can’t always tell in advance, but **a “slipping,” “about to come out,” or fear in certain positions is the key warning sign** of real instability.<br><br>If you want, I can also tell you which specific movements are most likely to provoke a dislocation so you can avoid them during recovery.	* **The Check:** When you move your arm, does it feel like there is "slack" in the joint before the bone starts to move? If the ball feels like it is sliding around inside the socket rather than rotating smoothly, the capsule is likely stretched out.<br><br>### 4. Night Pain and Aching<br>If the shoulder feels unstable, the surrounding muscles (the rotator cuff) have to work overtime to hold it in place.<br>* **The Sign:** Constant aching in the "deltoid" (the outer shoulder muscle) even when you aren't doing much. This is muscle fatigue from those muscles trying to do the job the torn ligaments can no longer do.<br><br>### 5. Catching or Popping<br>Frequent, painful popping or a sensation that something is "catching" inside the joint can indicate that a piece of the torn labrum (the cartilage ring) is flipping into the joint. This can act like a wedge that "unseats" the ball from the socket.<br><br>### How to Stay Safe<br>If you start feeling these signs, it means your shoulder is "unreliable." At this point, you should:<br>* **Avoid the "Stop Sign" position:** Keep your elbow tucked near your side.<br>* **See your specialist:** Tell them you are feeling "apprehension." This is a key clinical term that will help them decide if you need an MRI or a change in your physical therapy.<br>* **Don't "test" it:** Don't keep moving the arm into the position that feels scary just to see if it still feels that way. Every time you "test" it, you stretch the tissue further.<br><br>**The Expert Guide:**<br>Most people feel a bit nervous after a dislocation, which is normal. The real question is whether that feeling goes away as you get stronger. Have you tried to reach for anything yet and felt that "slipping" sensation, or have you been keeping it strictly in the sling?	This is a very specific and reliable indicator for anterior dislocations. You will notice a wave of anxiety or nausea when you put your arm in a specific position, even if you are just *thinking* about doing it.<br>- **The Position:** **Arm out to the side, elbow bent 90 degrees, hand rotated backward** (like a baseball pitcher in the "cocked" position or reaching back to throw a ball).<br>- **What it feels like:** You won't necessarily feel pain. You will feel a deep, visceral **fear** that the shoulder is about to pop out. Your muscles will involuntarily spasm to stop you from moving further.<br>- **Action to Take:** **Respect this feeling.** It is your brain interpreting signals from the stretched ligaments and torn labrum. Avoid this position entirely during the early recovery phase.<br><br>### 3. Sudden Muscle Spasms or "Dead Arm"<br>Sometimes the warning sign isn't a mechanical clunk, but a neurological response.<br>- **What it feels like:** You might be reaching for something low-risk (like a glass on a table) and suddenly the deltoid or bicep muscle **cramps or seizes up violently**. The arm might feel heavy and weak for a few seconds, like it's not connected to your brain.<br>- **Why this happens:** The nerves that control the muscles detect the joint becoming unstable and fire a "shutdown" command to prevent you from moving further.<br>- **Action to Take:** Stop the activity immediately and support the arm with the other hand or the sling.<br><br>### 4. Differentiating "Healing Pain" from "Instability Pain"<br>This is the hardest part of recovery. How do you know if the sharp pain you just felt is normal scar tissue stretching, or the joint about to fail?<br>- **Healing Pain:** Usually achy, dull, or a "burning stretch" in the front or back of the shoulder. It gets better when you rest or change position. It does **not** feel like the joint is slipping.<br>- **Instability Pain:** **Sharp, electric, and sudden.** It is accompanied by that distinct sensation of movement or shifting *inside* the joint. It makes you want to grab your shoulder to hold it still.<br><br>### 5. Nighttime Instability (The Silent Threat)<br>Many second dislocations happen **in bed**. You cannot rely on conscious warning signs here.<br>- **The Warning Sign (Before Bed):** If you lie on your back and let the injured arm fall out to the side even slightly, and you feel that "apprehension" or pulling in the front of the shoulder, **do not sleep without the sling**.

			- **Prevention:** Even if you feel "fine," sleeping with the sling on (or with a large pillow wedged under the armpit to prevent the arm from rolling back) is the only way to guard against this.<br><br>### What to Do If You Feel the Warning Signs<br>If you feel that slip, slide, or intense apprehension:<br>1. **FREEZE.** Do not finish the motion you were making.<br>2. **BRING THE ARM IN.** Move the hand toward your opposite hip.<br>3. **RELAX THE TRAP.** Consciously drop your shoulder blade down. Shrugging up toward the ear makes it easier for the ball to slip forward.<br>4. **APPLY THE SLING.** Put the sling back on for a few hours to let the inflammation and muscle guarding settle down.<br><br>**When to See the Doctor Immediately:**<br>If you feel a **"clunk" followed by relief**, it's possible the shoulder briefly slipped partially out and reduced itself. You should let your orthopedic surgeon know. If you feel the **arm go completely dead or you see a visible deformity** (a bulge in the front or a hollow in the back), that is a full dislocation and requires an ER visit.
20	Even if your shoulder starts feeling better, there are still situations where you should **see a doctor or follow up with orthopedics/physiotherapy**, because recovery after a first dislocation is not just about pain going away.<br><br>### You should definitely follow up (even if you feel better) if:<br><br>* You had a **first-time shoulder dislocation** (this alone is usually a reason for follow-up)<br>* You haven't already been given a clear **rehabilitation or physiotherapy plan**<br>* You are supposed to have a **check-up appointment** (this is common after ED treatment)<br><br>This follow-up is important to assess:<br><br>* Joint stability<br>* Risk of repeat dislocation<br>* Whether you need structured physiotherapy<br>* Sometimes hidden injuries (like a labrum tear)<br><br>### You should see a doctor sooner if you notice:<br><br>* A feeling that the shoulder is **slipping, shifting, or unstable**<br>* **Repeated "near dislocations"**<br>* Pain that **comes back or doesn't keep improving**<br>* **Persistent stiffness** that is not gradually improving	Even if your shoulder feels significantly better in a few days, you should still see an orthopedic specialist within **7 to 14 days** of the injury.<br><br>In the world of shoulder injuries, "feeling better" can be deceptive. The pain and swelling usually subside long before the internal damage has actually healed. Here is why that follow-up appointment is non-negotiable for someone your age:<br><br>### 1. The "Invisible" Damage Check<br>The emergency room's job was to make sure your bones were in place and your nerves were working. A specialist's job is to look at the "soft tissues." Because you are 20, there is a very high chance you have a **Bankart lesion** (a torn labrum). This won't show up on a standard X-ray, but it is the main reason shoulders come out a second time.<br><br>### 2. Getting an MRI<br>An orthopedic surgeon will decide if you need an MRI. This is the only way to see the extent of the tearing. Knowing exactly what is torn allows them to give you a realistic "return to play" timeline for sports or the gym.<br><br>### 3. Professional "Stability Testing"<br>A doctor will perform specific, gentle physical tests (like the "Apprehension Test") to see how much "play" or looseness is in the	Yes, you should absolutely still see a doctor for follow-up even if your shoulder feels better. In fact, feeling "better" can be misleading during the early stages of healing for a shoulder dislocation.<br><br>The absence of pain does not equal complete healing of the internal structures (like the labrum) that were damaged. The follow-up appointment is a critical checkpoint to assess whether the shoulder is healing in a way that will be stable for the long term, especially since you are young and active.<br><br>Here are the key milestones for when you should plan to see a doctor again, regardless of how good you feel:<br><br>* **Routine Follow-up (Approximately 3 Weeks Post-Injury)**: You should attend a scheduled follow-up with an orthopedic specialist roughly 3 weeks after the injury . The doctor will assess your progress, check for any developing issues with stability, and give you clearance to begin the next phase of rehabilitation. Do not skip this appointment just because you are not in pain.<br><br>* **If You Feel Instability or a "Clunk"**: This is a non-negotiable reason to see the doctor immediately. If you experience a sensation that the shoulder is slipping, shifting, or if you feel a "clunk" during movement, this could be a sign of a **Bankart lesion** (a tear in the labrum) that is not healing on

	* Any **numbness, tingling, or weakness** that continues<br>* You cannot return to basic daily use of the arm over time<br><br>### You should also get checked before returning to sport if:<br><br>* You play contact sports (football, wrestling, basketball, etc.)<br>* You do overhead sports (volleyball, swimming, throwing sports)<br>* You are unsure if the shoulder is “strong enough” yet<br><br>### Typical follow-up timing<br><br>Many people are reviewed within:<br><br>* **1–2 weeks after injury** (initial check)<br>* Then again during **rehabilitation (3–6 weeks or longer)**<br><br>### Bottom line<br><br>Even if your shoulder feels better, a **first-time dislocation at your age should usually still be followed up**, because the main goal is not just pain relief—it’s preventing it from happening again.<br><br>If you want, I can help you figure out whether your current symptoms sound like you’re ready for rehab progression or if you should be seen sooner.	joint. They can feel things you can't, helping to determine if your shoulder is "stable" or "unstable."<br><br>### 4. Setting Up Physical Therapy<br>You shouldn't start exercising on your own. A doctor needs to write a specific prescription for physical therapy that tells the therapist exactly which movements to avoid and which muscles to target. Starting the *wrong* exercises too early can actually make the instability worse.<br><br>### 5. Discussing Your Risk Profile<br>Since you are in the highest risk group for re-injury, a specialist will discuss whether your lifestyle (e.g., playing contact sports, heavy lifting) requires a more aggressive treatment plan now to prevent a lifetime of dislocations.<br><br>---<br><br>### Summary Checklist for Your Visit:<br>When you see the doctor, make sure to ask these three questions:<br>* "Based on my age and activity level, what are the chances this happens again?"<br>* "Do I need an MRI to check for a labral tear?"<br>* "When can I safely start physical therapy to strengthen my rotator cuff?"<br><br>**The Bottom Line:** Don't let the lack of pain fool you. The first few weeks are the "golden window" for proper healing. If you miss this window by assuming you're fine, you might end up dealing with a "loose" shoulder for years.<br><br>Do you already have a follow-up appointment on the calendar, or do you need help knowing what kind of specialist to look for in your area?	its own and may require surgical discussion . In young patients, a true feeling of the joint moving is a red flag.<br><br>* **If Pain or Function Plateaus or Worsens**: Recovery is not always a straight line, but you should see consistent, gradual improvement week over week. If your pain, stiffness, or weakness **stops improving for 2-3 weeks** or suddenly gets worse, this warrants a check-up. It could indicate that scar tissue is forming improperly or that there is a mechanical block in the joint .<br><br>* **Before Returning to Sport (At least 6-12 Weeks+)**: Even when you feel 100% in daily life, you **must** be cleared by a doctor or physiotherapist before returning to sports, especially contact or overhead activities. Returning too early based on "feeling fine" is the most common cause of a second dislocation. Most protocols require a formal assessment of your strength and range of motion against specific benchmarks, not just time passed .<br><br>**Summary:** The follow-up appointment at the 3-week mark is a **mandatory** part of the treatment plan, even if your pain is completely gone. It ensures the shoulder is on the right track and prevents you from accidentally progressing to activities that feel okay but put the internal healing at risk.
21	Yes—if your shoulder comes out again, you should go to the hospital or an emergency department right away.<br><br>A repeat dislocation can be very painful and may cause additional damage to the joint, surrounding ligaments, or nearby nerves and blood vessels. Even if it seems similar to the first time, it’s important to have it checked and safely put back in place by a medical professional. Trying to force it back yourself or waiting too long can make things worse.<br><br>You should seek immediate care especially if:<br><br>* The shoulder looks visibly out of place<br>* You have severe pain or cannot move your arm<br>* You feel numbness, tingling, or weakness in your arm or hand	### **Yes, you should go to the hospital immediately.**<br><br>Even if you’ve been through it once and think you know what to expect, every dislocation is different. If your shoulder comes out again, it is considered an **orthopedic emergency**.<br><br>Here is exactly why you need professional medical help right away:<br><br>### 1. The Risk of Nerve and Vessel Damage<br>Every time the "ball" of your arm bone sits outside the socket, it is pressing against a major highway of nerves and blood vessels. While you were lucky the first time, a second dislocation could pinch or stretch these structures differently, leading to permanent numbness or loss of hand function if not corrected quickly.	Yes, absolutely. If your shoulder comes out again, you should go to the hospital or emergency department right away. Do not wait to see if it will "fix itself" and do not attempt to have a friend or coach put it back in for you.<br><br>Here is exactly why immediate medical attention is necessary, even though you have been through this process before.<br><br>### 1. The Risk of a Fracture is Higher the Second Time<br>You were fortunate that your first dislocation had no fractures. However, with a recurrent dislocation, the pattern of injury changes. Because the soft tissues (ligaments and labrum) are already loose or torn from the first event, the force of the second dislocation is often transferred directly to the **bone**.

	* Your arm looks pale or feels cold (which could signal a circulation problem)<br><br>After a first dislocation, the risk of it happening again is higher—especially in younger people and during sports. Following your doctor’s advice about rest, wearing a sling, and doing rehabilitation exercises can help reduce that risk.<br><br>If you want, I can also explain how to lower the chances of it happening again or what warning signs to watch for during recovery.	### 2. Muscle Spasms Make it Harder to Fix<br>As soon as the shoulder dislocates, your muscles go into "guarding" mode—they tighten up to protect the area. The longer you wait, the more these muscles spasm. If you wait too long, the muscles become so tight that doctors may have to use heavy sedation or even general anesthesia to get the joint back in place.<br><br>### 3. You Need a "Pre-Reduction" X-ray<br>You cannot tell from the outside if a bone broke while the shoulder was popping out. If a doctor tries to put the shoulder back in while there is an undiagnosed fracture (bone break), they could accidentally cause much more severe damage or displace the broken bone further.<br><br>### 4. Avoiding "Self-Reduction" Hazards<br>It is tempting to try to "pop" it back in yourself to save a trip to the ER. **Do not do this.** Forced maneuvers without professional training can:<br>* Tear your labrum (cartilage) further.<br>* Cause a fracture of the socket.<br>* Trap soft tissue inside the joint, which would then require surgery to fix.<br><br>### 5. Proper Pain Management<br>The ER can provide the necessary pain relief and muscle relaxants to make the "reduction" (putting it back in) as smooth and painless as possible.<br><br>---<br><br>### **What to do on the way to the hospital:**<br>* **Immobilize it:** Use a sling or a makeshift wrap (like a scarf or a tucked-up shirt) to keep the arm from dangling.<br>* **Ice it:** Apply an ice pack to the area to help with the pain and keep the swelling down.<br>* **Stay "NPO" (Fast):** Do not eat or drink anything on the way. If the doctors need to give you sedation to put the shoulder back in, having an empty stomach makes the process much safer.<br><br>### **The Bottom Line**<br>Because you are 20, if it happens a second time, it is a clear sign that your shoulder is "unstable." After the hospital puts it back in, your next priority should be seeing an **orthopedic sports specialist** as soon as possible to discuss a long-term fix, as the risk of it happening a third or fourth time is extremely high once the "seal" has been broken twice.	- **What can happen:** The edge of the socket (glenoid rim) can chip off, or the ball (humeral head) can sustain a larger compression fracture (Hill-Sachs lesion).<br>- **Why the ER is necessary:** You cannot feel a fracture. You need an **X-ray** to rule out a new bony injury before anyone attempts to put the shoulder back in place. Attempting a reduction on a fractured shoulder can displace the bone fragment and turn a simple injury into a surgical emergency.<br><br>### 2. Reduction Becomes More Difficult<br>As mentioned previously, the muscles around a shoulder that has dislocated multiple times learn to spasm **harder and faster**.<br>- **The Experience:** While the first reduction might have been relatively quick in the ER, a second attempt is often much more painful and resistant to manual manipulation.<br>- **Medical Need:** You will likely require **conscious sedation** (strong medication through an IV) to relax the muscles enough to allow the joint to slide back into the socket. This can only be done safely in a monitored hospital setting, not at home or on the sideline.<br><br>### 3. The "Self-Reduction" Trap<br>Sometimes, a recurrent dislocation will "pop" back into the socket on its own while you are moving or on the way to the hospital. This is called a spontaneous reduction.<br>- **Do NOT cancel the ER visit.** Even if the shoulder feels back in place and the severe pain is gone, **you still need an X-ray**.<br>- **The Hidden Danger:** That "pop" back in could have been the result of a piece of cartilage or bone snapping off and allowing the joint to settle. You may now have a **loose body** floating inside the joint. These loose bodies act like a pebble in a gear; they can scratch the smooth joint surface, cause arthritis, and lock the shoulder up unexpectedly. An X-ray and follow-up MRI are needed to check for this.<br><br>### 4. Pain Management and Muscle Spasm<br>A recurrent dislocation often triggers a more intense inflammatory response and muscle spasm than the first one. Even if the shoulder is reduced, the muscles may remain in a painful spasm (guarding) for hours.<br>- **ER Benefit:** The hospital can provide short-term, stronger anti-inflammatory and muscle relaxant medications to break the spasm cycle so you can rest comfortably in the sling. Over-the-counter medication is often insufficient for the first 24 hours after a second dislocation.<br><br>### 5. What to Do En Route to the Hospital

			While waiting for transport or driving (as a passenger), follow these steps:<br>- **Sling or Self-Support:** Use the sling if you have it, or cradle the injured arm across your belly with the opposite hand.<br>- **Pillow in Armpit:** If the arm is stuck out to the side (less common in anterior, but possible), place a small pillow or folded jacket **between the elbow and your ribs** to support the weight. Do not force the arm against the body.<br>- **Ice:** Apply ice to the **back** of the shoulder if it is tolerable. Avoid pressing on the front bulge.<br>- **Do Not Eat or Drink:** If the shoulder is out, there is a high likelihood you will need sedation for the reduction. Having food in your stomach will delay the procedure and increase the risk of complications.<br><br>**The Bottom Line:** A second dislocation is a major event. It signals that non-surgical management is failing. You should go to the hospital for safe reduction, fracture assessment, and to begin the conversation about **definitive surgical stabilization** to prevent a third and fourth occurrence.
22	In your situation—a first-time anterior shoulder dislocation at age 20 with no fractures or nerve injury—surgery is **not automatically required**, but your risk of needing it later is higher than average.<br><br>Here’s how it generally breaks down:<br><br>* **Many people are treated without surgery at first.** This usually includes a short period in a sling, followed by physical therapy to restore strength and stability.<br>* **However, younger patients (especially under 25)** have a **higher chance of the shoulder dislocating again**. This is because the soft tissues that stabilize the joint can remain stretched or torn after the first injury.<br><br>So, will you need surgery later?<br><br>* **You might not** if:<br><br>* Your shoulder heals well<br>* You regain good strength and control<br>* You don’t experience repeated dislocations or instability<br><br>* **Surgery becomes more likely if:**<br><br>* Your shoulder dislocates again (even once more increases concern)<br>* You feel ongoing instability (like it might “slip out”)<br>* You want to return to high-risk sports (contact sports, overhead sports)	Whether or not you will need surgery is the "million-dollar question" for every 20-year-old with a shoulder dislocation. There is no way to know for sure without an MRI and a physical exam, but we can look at the factors that tilt the scales toward or away from the operating room.<br><br>In modern orthopedics, surgery for a first-time dislocation is becoming more common for people your age, but it is not always a requirement.<br><br>### Factors That Make Surgery **More Likely**<br>If any of these apply to you, a surgeon is more likely to recommend an "early" repair (usually a procedure called a **Bankart Repair**):<br>* **A Large Labral Tear:** If an MRI shows the cartilage (labrum) is completely torn off the bone, it acts like a loose bumper. It rarely heals back to the bone perfectly on its own.<br>* **Bone Loss:** If the dislocation "dented" the ball (Hill-Sachs lesion) or "chipped" the socket (Bony Bankart), the joint is physically less stable.<br>* **High-Impact Lifestyle:** if you play contact sports (football, rugby, wrestling) or have a job that requires heavy overhead lifting, the failure rate of "just physical therapy" is much higher.<br>* **Recurrence:** If your shoulder pops out a **second time**, most surgeons will stop recommending therapy and start recommending surgery, as the risk of permanent joint damage increases with every "pop."<br><br>### Factors That Make Surgery **Less Likely**<br>You might be able to avoid surgery if:	Whether you will need surgery later is a very fair question, and it's one that should be answered through a shared decision-making process with an orthopedic specialist. The decision is not automatic, but because you are 20 years old and active, the conversation about surgery is an important one to have now, rather than waiting to see if the shoulder dislocates again.<br><br>Here is a balanced look at the factors that will guide this decision, based on current medical evidence.<br><br>### The Two Main Treatment Paths<br>After a first-time anterior shoulder dislocation, management generally falls into one of two categories:<br><br>* **Non-operative Management:** This is the path you are currently on. It involves a period of immobilization in a sling followed by a structured course of physical therapy to strengthen the muscles that stabilize the shoulder. The goal is to compensate for the torn ligaments with muscle control .<br>* **Surgical Stabilization (Arthroscopic Bankart Repair):** This is a minimally invasive procedure where the surgeon reattaches the torn labrum (the cartilage bumper) to the socket using small anchors and sutures. The goal is to restore the normal anatomy and reduce the risk of future dislocations .<br><br>### Why Your Age Makes This a Critical Conversation<br>The primary reason surgery is a serious consideration for someone your age is the high rate of recurrence without it. The

	Because you're 20 and likely active, some doctors may **discuss early surgery as an option** even after the first dislocation to reduce recurrence risk—but this depends on your activity level and goals.<br><br>**What you should do now:**<br><br>* Follow your rehab program carefully<br>* Avoid risky movements early on<br>* Keep follow-up appointments with your orthopedic specialist<br><br>If you want, I can explain your personal risk of recurrence or how to decide between rehab alone vs early surgery.	* **It was a "Low Energy" Injury:** For example, you tripped and fell rather than being hit at high speed.<br>* **The MRI is Clean:** If the ligaments were just stretched but not detached from the bone.<br>* **You Respond Well to Physical Therapy:** If, after 6 weeks of rehab, your shoulder feels strong and you don't feel any "apprehension" (the feeling it will pop out).<br><br>---<br><br>### What does the surgery actually do?<br>If you do end up needing it, the most common surgery is **arthroscopic** (using tiny cameras and small incisions). The surgeon essentially:<br>1. Cleans up any frayed cartilage.<br>2. Uses tiny "anchors" (like specialized drywall anchors for bone) and heavy-duty thread to "tack" the labrum back onto the socket.<br>3. Tightens the "capsule" (the bag of ligaments) to take the slack out of the joint.<br><br>### The Trade-Off<br>* **Without Surgery:** You avoid the operating room now, but you carry a roughly **70–90% risk** (statistically) of it happening again at some point in your 20s.<br>* **With Surgery:** You have to deal with a longer initial recovery (usually 6 months before returning to sports), but your risk of another dislocation drops to **below 10%**.<br><br>### My Advice for Your Next Step<br>When you see the orthopedic specialist, ask them about your **"ISIS Score" (Instability Severity Index Score)**. It's a tool they use to calculate your risk of re-dislocation based on your age, the type of sport you play, and what your X-rays show. It can help you make an objective decision rather than just "guessing."<br><br>Do you feel like your shoulder is your "dominant" arm (the one you write or throw with)? This sometimes influences how aggressive people want to be with treatment.	shoulder's stabilizing ligaments rarely heal back to their original tight position on their own.<br><br>* **Statistical Reality:** Research shows that for active patients around the age of 20, the chance of the shoulder dislocating again with non-operative treatment alone can be as high as **80-90%** . This is compared to a recurrence rate that can be lowered to approximately **10-28%** with surgical repair .<br>* **Long-Term Benefit:** A 2025 randomized controlled trial with 6-year follow-up on patients under 25 found that those who had surgery had significantly better functional outcomes and a much higher rate of returning to sports compared to those treated without surgery .<br><br>### Factors That Help Determine the Best Path<br>Your orthopedic surgeon will help you weigh the pros and cons based on more than just age. Key considerations include:<br><br>* **Your Specific Injury:** An MRI will be crucial to assess the extent of the damage. A large, displaced labral tear (Bankart lesion) or any amount of bone loss in the socket makes future dislocations more likely and may tip the scales toward surgery .<br>* **Your Activity Level and Sports:** If you plan to return to contact sports (like football or rugby) or overhead sports (like swimming, tennis, or throwing), the demands on your shoulder will be high. Surgery is often recommended in these cases as it provides a more reliable foundation for return to play .<br>* **The Course of Non-Operative Treatment:** This is a "wait and see" approach with clear rules. If you strictly follow the sling and physical therapy protocol but still experience a **second dislocation**, the conversation is effectively over. At that point, surgery becomes the standard recommendation to prevent further damage to the joint and a third or fourth dislocation .<br><br>### The Risks of Delaying a Decision<br>It's important to know that recurrent instability isn't just an inconvenience. Each time the shoulder dislocates, it can cause additional damage. The bone of the socket can be worn away, and the dent in the humeral head (the ball) can get larger . This accumulated damage can make a future surgical repair more complex. A straightforward soft-tissue repair may no longer be an option, and a more involved operation to reconstruct the bone might be needed .<br><br>### Summary of Your Situation<br>* **Will you need surgery later?** **Possibly, maybe even likely**, but it is not an absolute certainty right now.

<table>
<tr><td></td><td></td><td></td><td>*   **The Next Step:** You are on the right track with non-operative care for the moment. The best course of action is to schedule a follow-up appointment with an orthopedic surgeon. They can examine you, review any imaging, and discuss your personal risk factors and goals.
*   **The Goal:** Whether through dedicated rehabilitation or a surgical repair, the objective is to give you a shoulder that is stable and reliable enough for you to live your life and play your sports without fear of it popping out again.</td></tr>
<tr><td>23</td><td>This is a very common and important question after a first shoulder dislocation—especially at your age. The honest answer is: **both options can be reasonable**, but they come with different trade-offs.

Healing without surgery (non-operative treatment)

Your shoulder *can* heal and function well without surgery, and this is usually the first approach.

Pros:

* No surgical risks
* Many people regain good strength and return to normal daily activities
* Avoids recovery time from an operation

Cons:

* At age 20, the risk of the shoulder dislocating again is relatively high (often estimated around 50% or more in young active people)
* Each repeat dislocation can cause more damage to the joint

This option is often chosen if:

* You don’t play high-risk sports (like football, basketball, or overhead sports)
* You’re okay accepting some risk of recurrence
* Your shoulder feels stable after rehab

Early surgery (stabilization procedure)

This usually involves repairing the torn stabilizing structures of the shoulder.

Pros:

* Significantly lowers the risk of future dislocations
* May better protect the joint from long-term damage</td><td>This is the core debate in sports medicine today, especially for a **20-year-old**. There isn't a single "right" answer, but there are two distinct paths. Think of it as a choice between **risk management** and **recovery time**.

Here is how the two options stack up:

Path A: Healing Without Surgery (Conservative Management)
Your shoulder *can* heal without surgery, but it heals with "scar tissue" rather than a perfect structural repair.
* **The Goal:** Use physical therapy to make your **rotator cuff muscles** so strong that they act as a "sleeve" to hold the joint in place, compensating for the torn or stretched ligaments.
* **The Pros:** You avoid the risks of surgery (infection, anesthesia, etc.) and you can get back to light activity and the gym much faster—usually within **6 to 8 weeks**.
* **The Cons:** You are playing a numbers game. Because you are young, the risk of it popping out again remains high (often over **70%**). If it pops out again, you may lose more bone, making a future surgery more complicated.

Path B: Surgery Early (Stabilization)
In active 20-year-olds, many surgeons now lean toward "early stabilization" (Bankart repair).
* **The Goal:** Physically re-attach the torn labrum to the bone using tiny anchors to restore the "bumper" that keeps the arm in place.
* **The Pros:** It provides the most stable shoulder possible. It drops your risk of another dislocation from roughly **80% down to less than 10%**. It’s the "fix it once and fix it right" approach.
* **The Cons:** It is a long road. You are in a sling for 6 weeks, and you typically cannot return to contact sports or heavy overhead lifting for **6 months**.

Comparison at a Glance

| Feature | No Surgery (PT Only) | Early Surgery |
| :--- | :--- | :--- |</td><td>This is the central question for someone your age after a first-time dislocation, and the answer is nuanced because it truly depends on balancing the high success rate of surgery against the potential for your shoulder to heal well without it.

The decision is not black and white. Both paths—early surgery and an initial trial of non-operative treatment—are valid and can lead to excellent outcomes. The key is understanding the trade-offs to make an informed choice with your orthopedic surgeon.

The Case for Early Surgery (Arthroscopic Stabilization)
Surgical treatment, typically an arthroscopic Bankart repair, is very effective at restoring stability because it mechanically reattaches the torn labrum (the cartilage bumper) to the socket . The primary advantage is a significant reduction in the chance of a second dislocation.

*   **Significantly Lower Recurrence:** Multiple high-level studies show that surgery reduces the risk of another dislocation. For a young, active person, the risk of recurrence with non-operative treatment can be quite high. Research indicates that arthroscopic Bankart repair can lower the 2-year recurrence rate to the range of **2-14%** , which is a substantial improvement over non-operative management .
*   **Better Long-Term Function:** Studies with long-term follow-up (over a decade) show that patients who have early surgery report excellent shoulder function and are able to return to high-level activities, including sports, with a high degree of confidence in their shoulder .
*   **Cost-Effectiveness:** Although surgery has a higher upfront cost, a 2024 cost-effectiveness analysis found that for a 20-year-old patient, early surgery is actually more cost-effective in the long run compared to a trial of non-operative care. This is because it avoids the costs and quality-of-life impact of repeated dislocations, ER visits, and the eventual high likelihood of needing surgery anyway .

The Case for a Trial of Non-Operative Treatment (Sling + Physical Therapy)</td></tr>
</table>

<table>
<tr>
<td></td>
<td>* Often preferred for athletes or very active individuals

Cons:

* Requires surgery and recovery time
* Small risks (infection, stiffness, complications)
* Rehab is still needed afterward

What matters most in your decision

At your age (20), doctors often lean *more toward surgery* **if you are very active or play sports**, because:

* Younger patients have a higher recurrence risk
* Preventing repeated dislocations can protect your shoulder long-term

But if you're less active or prefer to avoid surgery, trying rehab first is completely reasonable.

A simple way to think about it

* **Low activity + stable shoulder after rehab →** Try non-surgical treatment
* **High activity (sports) or concern about recurrence →** Consider early surgery

---

Bottom line

Your shoulder *can heal well without surgery*, but **there’s a meaningful risk it may dislocate again at your age**. Early surgery reduces that risk but isn’t mandatory after a first dislocation.

If you want, I can help you estimate your personal recurrence risk based on your sport and activity level, or explain what surgery and recovery actually look like.</td>
<td>| **Initial Recovery** | Fast (Weeks) | Slow (Months) |
| **Long-Term Stability** | Lower (Risk of re-injury) | Much Higher |
| **Risk of Arthritis** | Higher (if it keeps popping) | Lower (protects the joint) |
| **Best For...** | Low-impact lifestyle | Athletes/Active 20-somethings |

How to Decide?
A "healing well" outcome without surgery is very possible if you are willing to be incredibly disciplined with physical therapy and perhaps "dial back" high-risk activities (like wrestling or football) for a year.

However, surgery is often considered "better" early if:
1. **Your MRI shows a "displaced" tear:** The labrum is so far off the bone that it physically *cannot* knit back together on its own.
2. **You have "Bony" damage:** You have a chip in the socket.
3. **You cannot live with the "Apprehension":** If the fear of it popping out keeps you from enjoying your life or sports.

The "Middle Ground"
Most doctors will start you with **Physical Therapy for 6 weeks**. If at the end of that time your shoulder still feels "loose" or you feel that "apprehension" (the dread that it will slip), that is usually the signal that your body cannot heal sufficiently on its own and surgery is the better path.

Does your current lifestyle or hobby involve "high-risk" movements, like throwing a ball, rock climbing, or contact sports?</td>
<td>Your shoulder **can** heal well without surgery, and this is still a very reasonable first step. The goal here is to see if your body can form enough scar tissue and muscle control to create a stable, functional joint without an operation.

* **Avoiding Surgery:** This path allows you to avoid the inherent, though small, risks of any surgical procedure (infection, nerve injury, stiffness) and the post-operative rehabilitation.
* **High Success is Possible:** The statistics for non-operative treatment aren't all negative. Studies show that a significant portion of patients—up to **50-75%** in some analyses—do *not* experience a second dislocation . For these individuals, they have successfully avoided surgery.
* **Reserved Option:** Choosing non-operative treatment first doesn't "burn a bridge." If your shoulder were to dislocate again, you can still have the stabilization surgery at that point, with results that are comparable to having it done immediately .

Key Factors to Weigh in Your Decision
The "best" choice often comes down to your personal circumstances and goals. Here is a framework for thinking about the two options:

* **Your Sport/Activity Level:**
  * **Favors Early Surgery:** You are a competitive athlete or regularly participate in contact sports (football, rugby, martial arts) or overhead sports (baseball, swimming, tennis). The demands of these activities place high stress on the shoulder.
  * **Favors Non-Operative Trial:** You are active recreationally but can modify your activities to avoid high-risk arm positions during and after recovery.

* **Your Tolerance for Risk:**
  * **Favors Early Surgery:** The idea of the shoulder "slipping" or dislocating again during an important life event, vacation, or game is unacceptable to you.
  * **Favors Non-Operative Trial:** You are willing to accept the higher statistical risk of a second event, with the understanding that you would then proceed with surgery.

* **Time and Life Demands:**
  * **Favors Early Surgery:** You have a defined "off-season" or a window of time where a 4-6 month recovery from surgery would be less disruptive to your life or career.
  * **Favors Non-Operative Trial:** You cannot afford the downtime associated with surgical recovery right now (e.g., starting a new job, school commitments).</td>
</tr>
</table>

			### Making the Decision with Your Doctor<br>The most important next step is to **discuss this choice with an orthopedic surgeon**. They will examine your shoulder and likely order an MRI to assess the exact nature of the injury. The size and location of the labral tear, or the presence of any bone damage, can tip the scales strongly toward one option over the other.<br><br>The decision should be a **shared one**, where your surgeon explains the medical findings and you share your personal goals and expectations. There is no single right answer, but there is a best answer *for you*.
24	Surgery after a first anterior shoulder dislocation isn’t routine, but there are **clear situations where doctors are more likely to recommend it**—either early on or after a trial of rehabilitation.<br><br>### Surgery is more strongly recommended if:<br><br>**1. The shoulder dislocates again (recurrent instability)**<br><br>* This is the most common reason<br>* Even one repeat dislocation in a young person often leads doctors to suggest surgery<br>* Multiple episodes make surgery very likely<br><br>**2. You feel ongoing instability**<br><br>* Sensation that the shoulder might “slip out”<br>* Apprehension when your arm is in certain positions (especially overhead or rotated outward)<br>* Difficulty trusting your shoulder in daily activities or sports<br><br>**3. You play high-risk or competitive sports**<br><br>* Contact sports (e.g., football, martial arts)<br>* Overhead or throwing sports (e.g., basketball, volleyball)<br>* In these cases, surgery may be recommended **even after the first dislocation** to reduce the chance of recurrence<br><br>**4. Imaging shows significant structural damage**<br>Even if this wasn’t seen initially, further scans (like MRI) might reveal:<br><br>* A labral tear (common after dislocation)<br>* Bone loss from the socket or humeral head<br>These findings increase the risk of repeat dislocation and favor surgery<br><br>**5. You are young and very active**	Surgeons generally don't want to operate unless they have to, but for a 20-year-old, there are specific "tipping points" where the medical community agrees that surgery is the more reliable option.<br><br>Here are the specific situations where surgery would likely be recommended:<br><br>### 1. Significant Bone Loss (The "Bony Bankart")<br>If your X-ray or CT scan shows that a piece of the **socket (glenoid)** broke off when the shoulder dislocated, the "platform" the arm sits on is now too small. Muscles cannot fix a missing piece of bone. Surgery is almost always required to secure that bone fragment or rebuild the socket to prevent constant dislocations.<br><br>### 2. Large or "Displaced" Labral Tears<br>A **Bankart Lesion** is a tear of the cartilage ring (labrum). If an MRI shows the labrum is torn and has flipped away from where it belongs:<br>* It cannot "crawl" back to the bone to heal.<br>* It essentially becomes a "flap" that can get caught in the joint, causing clicking, pain, and instability.<br><br>### 3. Chronic "Apprehension" or Failed Rehab<br>If you complete 6 to 12 weeks of high-quality physical therapy and your shoulder still:<br>* Feels like it is "giving way."<br>* Prevents you from lifting your arm over your head due to fear.<br>* Slips out during low-impact activities (like putting on a jacket or sleeping).<br>This means your muscles—no matter how strong—cannot compensate for the structural damage.<br><br>### 4. High-Demand Lifestyle (The "Athlete" Rule)<br>In your 20s, if you play **contact sports** (football, rugby, MMA) or have a **heavy manual labor job** (construction, firefighting), surgeons often recommend surgery after the *first* dislocation.	That is an excellent follow-up question. It gets to the heart of how orthopedic surgeons make the decision between continuing with a sling and physical therapy versus proceeding with an operation.<br><br>While the choice is sometimes a shared decision based on lifestyle, there are specific clinical situations where surgery shifts from being an "option" to being a **strong recommendation**. Here are the clear scenarios where an orthopedic surgeon would likely advise you to have surgery.<br><br>### 1. Failure of Non-Operative Management (A Second Dislocation)<br>This is the most straightforward and common reason. If you complete your sling immobilization, do your physical therapy diligently, and the shoulder **still dislocates a second time**, the recommendation is almost universally for surgery.<br>- **Why:** The shoulder has proven that the soft tissue healing (or the muscle compensation) is insufficient to keep the joint stable during normal or athletic activity. Continuing non-operative care after a second dislocation in a 20-year-old carries a recurrence risk approaching 90-95%. You would be delaying the inevitable while risking further bone damage.<br><br>### 2. Significant Bone Loss on Imaging (The "Inverted Pear")<br>When you had your initial injury, the force of the dislocation may have chipped the bone. An MRI or CT scan can measure this precisely.<br>- **Critical Glenoid Bone Loss:** The socket (glenoid) is shaped like a pear. If the front rim of that socket has been worn down or fractured off—typically more than **13.5% to 20%** of the surface area—the socket is no longer concave enough to hold the ball in place. This is called an "inverted pear" deformity.<br>- **The Recommendation:** In this scenario, soft tissue (labrum repair) alone has a very high failure rate. You would need surgery to either repair the bone fragment or reconstruct the

	* People under ~25 have a higher recurrence risk<br>* If you want a stable shoulder for long-term activity, surgery may be advised earlier<br><br>**6. Rehab doesn’t restore stability**<br><br>* If after proper physiotherapy your shoulder still feels weak, loose, or unreliable<br><br>---<br><br>### When surgery is usually *not* immediately needed:<br><br>* First-time dislocation<br>* Shoulder feels stable after rehab<br>* No major structural damage<br>* Lower activity demands<br><br>---<br><br>### Bottom line<br><br>Surgery is recommended when the **risk of future dislocation is high or instability is already a problem**—especially in young, active individuals like you.<br><br>If you’re unsure which category you fall into, your lifestyle (sports, activity level) and how your shoulder feels during recovery are key factors. I can help you weigh those if you want to go more personal.	* **The Reason:** The chance of a second dislocation in these environments is near 90%. Surgery "stabilizes the foundation" before you go back into the line of fire.<br><br>### 5. Recurrent Dislocations<br>Once the shoulder has come out **two or more times**, the joint capsule becomes like an overstretched rubber band. It loses its elasticity. At this stage, surgery is usually recommended to "tighten the bag" and prevent the long-term, permanent arthritis that comes from the bones grinding against each other every time it slips.<br><br>---<br><br>### What to ask your doctor to see if you fit these categories:<br>If you want to know if you are headed for surgery, ask your specialist these specific questions after they look at your imaging:<br><br>1. **"Is there any bone loss on the glenoid (socket) or a Hill-Sachs lesion on the humerus?"**<br>2. **"Is the labrum fully detached (displaced), or just frayed?"**<br>3. **"On a scale of 1 to 10, how 'loose' is my joint capsule?"**<br><br>### Summary<br>If your MRI is clean and your only hobby is gaming or walking, you likely won't need surgery. If your MRI shows a detachment and you want to be back in the gym or playing sports at a high level, surgery is often the standard recommendation to ensure your shoulder lasts the next 60 years.<br><br>Knowing what you do for fun or work—do you have to do a lot of overhead reaching or heavy lifting?	socket rim (a procedure called a Latarjet). Delaying surgery in this case allows the bone to resorb (dissolve) over time, making the eventual reconstruction more difficult.<br><br>### 3. A Large, Engaging Hill-Sachs Lesion<br>This is the "dent" in the back of the ball part of the shoulder.<br>- **The Mechanism:** When you lift and rotate your arm, that dent can catch or "engage" on the front rim of the socket, acting like a pry bar that levers the shoulder out of place.<br>- **The Recommendation:** If imaging shows a large, engaging Hill-Sachs lesion, surgery is recommended to either fill the dent (Remplissage procedure) or tighten the capsule to prevent that catching motion.<br><br>### 4. High-Risk Sport Participation or Occupational Demand<br>Sometimes the recommendation for surgery is not based on what *has* happened again, but on what is *likely* to happen based on what you do every day.<br>- **Contact/Overhead Athletes:** If you are a competitive football player, wrestler, rock climber, swimmer, or baseball pitcher, the specific motions of your sport replicate the exact mechanism of dislocation hundreds of times per practice. Studies show that non-operative management in this group leads to re-dislocation rates exceeding 70-80% during the first season back.<br>- **Occupational Hazard:** Jobs that require working at heights (firefighter, electrician, construction) where a sudden dislocation could result in a catastrophic fall. In these cases, the risk to life and limb justifies early surgical stabilization to ensure the shoulder is reliable.<br><br>### 5. Associated Rotator Cuff Tear (Less Common at 20, but Possible)<br>While rare in a 20-year-old compared to a 50-year-old, a violent dislocation can tear the rotator cuff tendon.<br>- **The Finding:** If an MRI reveals a **full-thickness rotator cuff tear** in conjunction with the labral tear.<br>- **The Recommendation:** The rotator cuff will not heal on its own and will only retract further over time. This requires surgical repair to restore strength and stability. Delaying this surgery can lead to irreversible muscle atrophy and fatty infiltration, making the tear irreparable in the future.<br><br>### 6. Persistent Symptoms of Instability (Apprehension) After Rehab<br>Even if the shoulder *never* fully dislocates a second time, you might complete 3-6 months of physical therapy and still feel like the shoulder is "loose" or you are terrified to use it.<br>- **The Definition:** You experience daily clicking, clunking, or the "dead arm" sensation when reaching overhead. You

			cannot sleep on that side. You self-limit all sports because you don't trust the joint.<br>- **The Recommendation:** Quality of life matters. If the shoulder is a constant source of anxiety and functional limitation despite good rehab effort, surgery is recommended to restore confidence and function.<br><br>**Summary for Your Situation:**<br>Right now, you are in the window of **"Watchful Waiting."** Surgery is not mandatory *today* because you haven't failed rehab yet, and we assume no critical bone loss was seen on your initial ER X-ray. However, if you fall into **Category 1 (Second Dislocation)** or **Category 4 (High-Risk Athlete)**, the conversation will shift decisively toward the operating room.
25	In most first-time anterior shoulder dislocations like yours, **permanent damage is not inevitable**—and many people recover fully and return to normal activities.<br><br>That said, there are a few important things to understand.<br><br>### What kind of “damage” can happen?<br><br>When the shoulder dislocates, some structures are almost always stretched or injured:<br><br>* The **labrum** (a ring of cartilage that stabilizes the joint) is often torn<br>* Ligaments can become loosened<br>* Sometimes there are small bone dents or defects<br><br>These changes don’t always cause long-term problems, especially if the shoulder becomes stable again.<br><br>### When does it become a long-term issue?<br><br>**1. If the shoulder keeps dislocating**<br><br>* Repeated dislocations can lead to cumulative damage<br>* This increases the risk of chronic instability and, over time, arthritis<br><br>**2. If instability persists**<br><br>* Feeling like the shoulder might slip out can limit activity and confidence<br>* This can become a chronic condition if not addressed<br><br>**3. If there was significant structural injury**	The short answer is: **It is unlikely to be "permanently damaged" in a way that ruins your life, but it is "permanently changed."**<br><br>Because you are 20, your body has an incredible capacity to adapt. However, a dislocation is a major structural event. Whether that change becomes a lifelong problem or just a "minor quirk" depends entirely on how you manage the next few months.<br><br>Here is how "permanent" the various types of damage are:<br><br>### 1. The Ligaments (Permanent Stretching)<br>Ligaments are like heavy-duty plastic straps. Once they are stretched or torn during a dislocation, they rarely return to their original, "factory-tight" tension.<br>* **The Reality:** The joint capsule will likely always be slightly looser than your uninjured side.<br>* **The Solution:** This is why physical therapy is so crucial. You have to train your **muscles** to take over the job the ligaments used to do.<br><br>### 2. The Labrum (Permanent Tearing)<br>The labrum (the cartilage ring) does not have a great blood supply, so it doesn't "knit" back together like a skin cut does.<br>* **The Reality:** If it is torn off the bone, it stays torn unless a surgeon tacks it back down.<br>* **The Risk:** A permanently torn labrum makes the "cup" of your shoulder shallower, which is why the risk of re-dislocation is higher.<br><br>### 3. The Bones (Hill-Sachs and Bankart Lesions)<br>If the bones hit each other during the dislocation, they can leave "dents" (Hill-Sachs lesion) or "chips" (Bony Bankart).<br>* **The Reality:** These are permanent changes to the shape of your bones.<br>* **The Impact:** Small dents usually don't matter. Large dents can act like a "speed bump" that makes the joint catch or slip.	That is a very understandable concern, and it is important to address it with honesty and clarity. The term "permanent damage" can mean different things, so let's break down exactly what changes inside your shoulder and what that means for you over the long term.<br><br>**The Short Answer:**<br>The structural injury that occurred during the dislocation—the tear in the labrum (cartilage bumper)—**rarely heals itself completely back to its original, perfect state.** In that specific anatomical sense, there is a permanent change inside the shoulder. **However**, that does not mean your shoulder is doomed to a lifetime of pain or dysfunction. Many people with this "permanent" labral tear live active, full lives without surgery, while others require a repair to achieve that stability.<br><br>Here are the specific ways permanent damage can manifest, and more importantly, how you can influence the outcome.<br><br>### 1. The Labral Tear and Long-Term Instability<br>This is the primary concern for a 20-year-old. The torn labrum creates a permanent mechanical **vulnerability**.<br>- **What is Permanent?** The cartilage itself has poor blood supply. It scars down, but it rarely reattaches tightly to the bone rim on its own. This means the "bumper" in the front of your shoulder is permanently shallower than it was before the injury.<br>- **The Variable (What You Can Control):** **Whether this vulnerability turns into *recurrent dislocations* is not set in stone.** Strict adherence to physical therapy strengthens the rotator cuff and shoulder blade muscles. These muscles act like a dynamic seatbelt, holding the ball in the shallow socket. If the muscles become strong enough, they can overcome the mechanical deficit of the torn labrum.<br>- **The Risk:** If the shoulder dislocates multiple times (3, 4, 5+ times), the permanent damage **expands**. Each pop

	* Larger tears or bone loss (even if not obvious at first) can affect long-term stability<br><br>### The good news for you<br><br>* You had **no fractures or nerve injury**, which is a very positive sign<br>* Many people in your situation recover well with proper rehab<br>* If needed, surgery later can still effectively restore stability<br><br>### Bottom line<br><br>Your shoulder is **not necessarily permanently damaged**, but there is a **risk of future instability**, especially at your age. The key factor is what happens next—whether your shoulder stays stable or dislocates again.<br><br>### What you can do to protect it<br><br>* Take rehabilitation seriously (this is crucial)<br>* Avoid rushing back into high-risk movements too soon<br>* Pay attention to any feeling of looseness or instability<br><br>If your shoulder remains stable, you may never have long-term problems. If instability develops, it’s something that can still be treated effectively.	 ### 4. Long-Term Arthritis Risk<br>This is the main concern with "permanent damage." Every time a shoulder dislocates, it scrapes the smooth, protective cartilage (the "teflon" coating) of the joint.<br>* **The Outcome:** If you dislocate it once and rehab it perfectly, your risk of arthritis is low.<br>* **The Danger:** If it keeps popping out for years, you can wear that coating away, leading to early-onset arthritis in your 30s or 40s.<br><br>---<br><br>### How to Prevent "Permanent" Disability<br>The "damage" may be permanent, but **disability is optional.** Most people who have one dislocation go on to lead completely normal, athletic lives.<br><br>**You are on the right track if you:**<br>* **Don't ignore it:** Treat it as a serious injury even when the pain stops.<br>* **Build "Armor":** Strong rotator cuff muscles are the best defense against permanent instability.<br>* **Follow Up:** Ensure a specialist checks that your nerves and blood vessels aren't compromised.<br><br>### The Bottom Line<br>Your shoulder is currently "compromised," but it isn't "broken" forever. With the right rehab (and potentially a minor procedure if the tear is large), you can get back to **100% function.**<br><br>Does your shoulder feel "weak" or "untrustworthy" when you move your arm, or is it mostly just hurting right now?	scrapes more cartilage off the socket rim and deepens the dent in the ball. This cumulative damage is what leads to severe long-term problems.<br><br>### 2. Post-Traumatic Arthritis (The Long-Term Wear and Tear)<br>This is the most significant form of "permanent damage" that can develop **years or decades** later.<br>- **The Mechanism:** Every time the shoulder dislocates or even partially slips (subluxes), the ball grinds against the edge of the socket. This is like a door scraping against a frame that is out of alignment. Over 10, 20, or 30 years, this abnormal contact can wear down the smooth articular cartilage, leading to **osteoarthritis**.<br>- **What the Studies Show:** A single dislocation increases the risk of developing arthritis in that shoulder later in life compared to a shoulder that never dislocated. The risk goes up exponentially with **every subsequent dislocation**.<br>- **How to Mitigate This:** **Preventing the second dislocation is the single most effective way to prevent early arthritis.** Whether you achieve that prevention through excellent physical therapy or through surgical stabilization, keeping the joint from popping out again protects the long-term health of the cartilage.<br><br>### 3. Nerve Damage (Axillary Nerve)<br>You were told there was no nerve injury in the ER, which is excellent news.<br>- **Permanent Risk:** In most first-time dislocations with no initial nerve symptoms, permanent nerve damage is **very rare**.<br>- **Recurrence Risk:** However, if the shoulder dislocates again, the nerve gets stretched again. Repeated stretching of the axillary nerve can lead to permanent numbness in a patch of skin over the deltoid muscle (the "badge" area). It is usually a sensory issue (numbness) rather than a motor issue (paralysis), but it is a permanent change.<br><br>### 4. Loss of External Rotation (Stiffness)<br>- **What Happens:** The front of the shoulder capsule scars down during healing. This is the body's natural way of protecting the joint from going into the dangerous "cocked" position again.<br>- **Is it Permanent?** **No, not necessarily.** With dedicated physical therapy and stretching (once cleared by your doctor), you can regain most, if not all, of your range of motion. Some elite throwers may notice a permanent 5-10 degree loss of maximum external rotation compared to before the injury, but for daily life and most sports, this is functionally unnoticeable.<br><br>### 5. The Psychological Damage (Kinesiophobia)

<table>
<tr><td></td><td></td><td></td><td>This is a form of "damage" that is often overlooked but is very real.
- **What Happens:** Your brain creates a hardwired fear response (apprehension) to prevent you from putting the arm in a dangerous position. Even if the shoulder is mechanically "fine" after rehab, your brain may never fully trust it again.
- **Is it Permanent?** Only if you let it be. This is why the **confidence-building phase of physical therapy** (plyometrics, sport-specific drills) is just as important as the strength phase. Learning to trust the shoulder again is a neurological process that takes time and guided exposure.

Summary: Can You Avoid "Permanent" Problems?
Yes, the trajectory is still largely in your hands and your doctor's hands.
- **Best Case Scenario (Non-Op):** The labrum scars down just enough. You do 6 months of excellent PT. Your muscles become a perfect seatbelt. You never dislocate again. **Result:** The labrum is permanently torn, but the **shoulder is permanently functional**. You win.
- **Best Case Scenario (Surgery):** You have an arthroscopic repair. The labrum is mechanically fixed back to the bone. You do 6-9 months of rehab. **Result:** The labrum is permanently *repaired*, and the **risk of arthritis is significantly reduced**.
- **Worst Case Scenario:** You ignore the sling and PT. You dislocate 4 times. You develop bone loss. You develop arthritis at age 35. **Result:** Permanent, structural damage to the joint surfaces.

You are at a crossroads right now. The decisions you make in the next 3-6 months regarding your sling use, physical therapy compliance, and follow-up with an orthopedic surgeon will determine which of those paths you go down.</td></tr>
<tr><td>26</td><td>Yes—you can take painkillers after a shoulder dislocation, and they’re commonly used to keep you comfortable while the joint heals. The key is choosing **safe options and using them correctly**. ### Common safe options **1. Paracetamol (acetaminophen)** * Usually the **first choice** * Helps with pain but not much with inflammation * Generally safe if you stay within the recommended dose * Avoid exceeding the daily maximum (often 3,000–4,000 mg depending on guidance and your health) **2. Anti-inflammatory drugs (NSAIDs)** Examples:</td><td>Yes, taking painkillers is a standard part of managing a shoulder dislocation, but at **20 years old**, you want to be strategic about which ones you use to balance pain relief with actual healing. Most doctors recommend a combination of two different types of over-the-counter medications because they work on different "pathways" of pain. ### 1. NSAIDs (Anti-inflammatories) These are usually the "gold standard" for dislocations because they don't just dull the pain; they physically reduce the swelling inside the joint. * **Common Examples:** Ibuprofen (**Advil, Motrin**) or Naproxen (**Aleve**). * **How they help:** They target the inflammation in the torn ligaments and the "angry" joint capsule.</td><td>Yes, you can and should take painkillers to manage discomfort after a shoulder dislocation. Controlling pain is important for rest and allows you to perform gentle elbow and wrist exercises comfortably . Over-the-counter options are typically sufficient, and you can choose from two main types: **Paracetamol (Acetaminophen)** and **Nonsteroidal Anti-inflammatory Drugs (NSAIDs)** like Ibuprofen. Here is a breakdown of the safe options and how to use them: | Medication Type	Examples	Primary Function	Key Considerations for Your Situation
Paracetamol	Tylenol, Panadol	Pain relief	Gentle on the stomach; safe to use as directed. Do not exceed 4,000 mg (4 grams) in 24 hours . |</td></tr>
</table>

* Ibuprofen
* Naproxen

These help with both **pain and inflammation**.

* Take with food to protect your stomach
* Avoid if you have stomach ulcers, kidney problems, or certain other conditions
* Don't combine multiple NSAIDs together

Are stronger painkillers needed?

Sometimes, doctors prescribe short-term stronger medications right after the injury, but for most people, **paracetamol or ibuprofen is enough after the first few days**.

Important safety tips

* Stick to recommended doses on the package or from your doctor
* Don't mix medications unless you're sure it's safe (for example, paracetamol + ibuprofen is usually okay, but two NSAIDs together is not)
* Avoid alcohol when taking painkillers
* If you have other medical conditions or take regular medications, check with a doctor or pharmacist

---

When to seek advice

* Pain is severe or not improving after a few days
* You need painkillers for more than 1–2 weeks
* You notice side effects (stomach pain, nausea, dizziness, etc.)

Bottom line

Yes, you can safely use painkillers—**paracetamol is the safest starting point**, and **ibuprofen or naproxen can help more if inflammation is an issue**, as long as you don't have reasons to avoid them.

* **Precaution:** These can be tough on your stomach. Always take them with food. If you have a history of stomach ulcers or asthma, check with a pharmacist first.

2. Acetaminophen (Pain Blockers)
* **Common Example:** Tylenol.
* **How it helps:** It works on the central nervous system to change how your brain perceives pain. It doesn't reduce inflammation, but it's great for the dull, heavy ache of the bone bruise.
* **Precaution:** Be very careful not to exceed the daily limit (usually 3,000mg to 4,000mg for a healthy adult), as too much can damage your liver. Remember that some "cold and flu" meds also contain Tylenol!

The "Staged" Approach to Pain
Doctors often suggest a "multi-modal" approach to keep you comfortable without needing heavy prescription drugs:

Timeframe	Recommended Approach
**Days 1–3** (Acute Phase)	Take Ibuprofen and Tylenol on a **rotating schedule** (e.g., Tylenol at noon, Ibuprofen at 3 PM) to keep a steady level in your system.
**Days 4–7** (Soreness Phase)	Transition to taking them only "as needed" before bed or before doing your gentle elbow/wrist movements.
**Week 2+** (Rehab Phase)	Use Ibuprofen specifically after physical therapy sessions if the joint feels "hot" or swollen.

A Warning on Prescription "Heavy" Painkillers
If the ER gave you a prescription for opioids (like Oxycodone or Hydrocodone), be very cautious.
* They are highly addictive and can cause severe constipation.
* They can make you drowsy, which increases your risk of falling and re-dislocating your shoulder.
* **Tip:** Try to switch to over-the-counter options as soon as the "sharp" pain settles into a "dull" ache.

What about "Natural" Painkillers?
* **Ice:** Do not underestimate ice. It is a "mechanical" painkiller. It numbs the nerves and is often more effective than a pill for that deep, throbbing pain.
* **Topical Creams:** Creams like **Voltaren (Diclofenac)** can be rubbed directly on the shoulder. This is great because it delivers

| **NSAIDs** | Ibuprofen (Advil, Motrin), Naproxen (Aleve) | Pain relief & reduces inflammation | May be more effective for the inflammatory pain you have now . Take with food. |

Key Advice on Taking Painkillers Safely
- **Follow a Schedule:** For the first few days, take painkillers regularly rather than waiting for pain to become severe. A consistent schedule helps maintain comfort . An example adult dose for ibuprofen is 400 mg every 8 hours; for paracetamol, it is 1,000 mg every 6 hours, never exceeding the maximum daily limit .
- **Alternating Medications:** If pain is not controlled by one medication alone, you can alternate between paracetamol and ibuprofen. For example, take paracetamol, then six hours later take ibuprofen, and continue alternating. This provides steady relief while staying within safe dosage limits for each drug .
- **Short-Term Use:** These medications are for short-term pain management, typically for a few days up to a week. Prolonged use of narcotic painkillers is not considered appropriate for this type of injury . If you find you need strong pain relief for longer than a few days, you should discuss it with your doctor .
- **Avoid Alcohol:** Do not drink alcohol while taking paracetamol or NSAIDs, as this can increase the risk of side effects, particularly liver damage with paracetamol .

An Important Note on NSAIDs and Healing
You may have heard concerns about anti-inflammatory medications (NSAIDs like ibuprofen) affecting bone or tissue healing. **In your specific case, this is generally not a significant concern for two reasons:**
1. **The injury is not a fracture:** The primary healing concern with NSAIDs relates to bone healing. Your injury involves soft tissues (ligaments and labrum).
2. **Short-term use is acceptable:** Research indicates that **non-selective NSAIDs** (which include standard ibuprofen) taken for a short period (up to 10-14 days) have not been shown to negatively impact the healing of soft tissues like the labrum in the shoulder .

If you have any pre-existing medical conditions like stomach ulcers, asthma, kidney, or liver problems, or if you are taking other medications, you should check with a doctor or pharmacist before starting new painkillers .

	If you want, I can suggest a simple day-by-day pain control plan for the first week of recovery.	anti-inflammatory medicine right to the spot without affecting your stomach.<br><br>### When to stop taking them:<br>If you find you are still needing maximum doses of painkillers after **10 days**, it's a sign that the inflammation isn't settling down on its own. This is a good reason to call your specialist for a follow-up.<br><br>**Important Note:** I am an AI, not a doctor. Always double-check with your pharmacist or the instructions on the bottle to ensure these don't interfere with any other medications you might be taking.<br><br>Are you currently taking anything, or were you waiting for advice before starting?	
27	You can take pain medication regularly for the first few days, but it's important to **follow safe timing and dose limits**. Here's a simple, patient-friendly guide:<br><br>### Paracetamol<br><br>* Typical dose: 500–1000 mg per dose<br>* **How often:** every 4–6 hours as needed<br>* **Maximum:** usually 3,000–4,000 mg per day (stay on the lower end if unsure)<br><br>This is usually the safest option and can be taken consistently for a few days.<br><br>---<br><br>### Ibuprofen<br><br>* Typical dose: 200–400 mg per dose (sometimes up to 600 mg if advised)<br>* **How often:** every 6–8 hours<br>* **Maximum:** 1,200 mg/day without a prescription (higher only if your doctor says so)<br><br>Take it with food to avoid stomach irritation.<br><br>---<br><br>### Naproxen<br><br>* Typical dose: 220–250 mg per dose<br>* **How often:** every 8–12 hours<br>* **Maximum:** about 500–1,000 mg per day depending on guidance	The frequency of taking pain medication depends entirely on the **type** of medicine and the **dosage** on the bottle. Because you are 20 and likely have a healthy liver and kidneys, you can usually follow the standard "max dose" protocols, but you must be careful not to overlap products.<br><br>Here is the standard breakdown for the most common medications used for a shoulder dislocation:<br><br>### 1. Ibuprofen (Advil, Motrin)<br>This is an NSAID that reduces inflammation.<br>* **How often:** Usually every **4 to 6 hours**.<br>* **Daily Max:** Do not exceed **3,200mg** in 24 hours (though most doctors suggest staying under **1,200mg–2,400mg** unless directed otherwise).<br>* **Pro-Tip:** Always take this with food to protect your stomach lining.<br><br>### 2. Naproxen (Aleve)<br>This is also an NSAID, but it lasts much longer than Ibuprofen.<br>* **How often:** Usually every **8 to 12 hours**.<br>* **Daily Max:** Do not exceed **660mg** (typically 3 pills) in 24 hours.<br>* **Pro-Tip:** This is often better for "night pain" because it stays in your system while you sleep.<br><br>### 3. Acetaminophen (Tylenol)<br>This is a pain blocker, not an anti-inflammatory.<br>* **How often:** Every **4 to 6 hours**.<br>* **Daily Max:** **Strictly do not exceed 3,000mg to 4,000mg** in 24 hours.<br>* **Warning:** Taking too much Tylenol can cause permanent liver damage. Many prescription painkillers (like Percocet or Vicodin)	For managing pain after your shoulder dislocation, it's generally best to take pain medication on a **regular schedule** rather than waiting for the pain to become severe. This approach helps maintain a consistent level of comfort, especially in the first few days following your injury .<br><br>Here is the standard dosing information for the common over-the-counter options you are likely using:<br><br>**Paracetamol (Acetaminophen)**<br>* **Typical Adult Dose**: 500 mg to 1000 mg every 4 to 6 hours as needed .<br>* **Maximum Daily Limit**: Do not exceed 4000 mg in 24 hours. Some guidelines recommend a more conservative limit of 3000 mg .<br><br>**Ibuprofen**<br>* **Typical Adult Dose**: 200 mg to 400 mg every 6 to 8 hours as needed .<br>* **Maximum Daily Limit**: Do not exceed 1200 mg in 24 hours for over-the-counter use .<br><br>For more effective, around-the-clock relief, you can alternate between taking ibuprofen and paracetamol. This means you can take them a few hours apart. For example, if you take ibuprofen at 8:00 AM, you could take paracetamol at 12:00 PM, and then ibuprofen again at 4:00 PM, continuing this pattern throughout the day . This method works well because the two drugs relieve pain through different mechanisms and have minimal overlapping side effects .<br><br>**Important safety reminders:**

	---<br><br>### Can you combine them?<br><br>* Yes: **paracetamol + ibuprofen** can be used together safely (they work differently)<br>* No: **don’t take ibuprofen and naproxen together** (both are NSAIDs)<br><br>---<br><br>### How long should you take them?<br><br>* Regular use for **3–5 days** after injury is common<br>* After that, take them **only when needed** as pain improves<br><br>---<br><br>### When to be cautious<br><br>* If you need them regularly beyond **1–2 weeks**<br>* If pain isn’t improving<br>* If you develop stomach pain, nausea, or other side effects<br><br>---<br><br>### Bottom line<br><br>You can safely take painkillers **every 4–8 hours depending on the medication**, as long as you **stay within daily limits**. Start simple (paracetamol), and add an anti-inflammatory if needed.<br><br>If you want, I can tailor a schedule based on how severe your pain is right now.	already contain Tylenol, so **never** mix them without checking the labels.<br><br>---<br><br>### The "Staggered" Schedule (The Professional Method)<br>If you are in significant pain, doctors often recommend "cycling" Tylenol and Ibuprofen so that you always have one medication working while the other is wearing off.<br><br><br><br>**Example Schedule:**<br>* **8:00 AM:** Take Ibuprofen (with breakfast).<br>* **11:00 AM:** Take Tylenol.<br>* **2:00 PM:** Take Ibuprofen (with a snack).<br>* **5:00 PM:** Take Tylenol.<br><br>*This keeps a steady level of pain relief in your bloodstream without hitting the maximum daily dose of either one too quickly.*<br><br>---<br><br>### Non-Pill "Medication"<br>Don't forget that **Ice** is a powerful painkiller that you can use frequently:<br>* **How often:** 15–20 minutes every hour.<br>* **Why:** It numbs the local nerves and constricts blood vessels to physically push swelling out of the joint.<br><br>### When to Slow Down<br>Pain medication is meant to "take the edge off," not to make you feel zero pain. If you feel **no pain at all**, you might accidentally move your arm in a way that re-injures the shoulder. You want to feel enough "reminders" to keep your arm still.<br><br>**Critical Safety Note:** If you notice any of the following, stop taking NSAIDs (Ibuprofen/Naproxen) immediately and call a doctor:<br>* Stomach pain or "heartburn" that won't go away.<br>* Dark, tarry stools.<br>* Ringing in your ears.<br><br>Are you currently taking a prescription from the ER, or are you just using what you had in your medicine cabinet at home?	* **Check Other Medications**: Be aware that some prescription pain relievers or cold/flu medications may already contain paracetamol (often labelled as APAP) or ibuprofen. Check the labels of all products you are taking to ensure you do not exceed the maximum daily dose of either ingredient .<br>* **Take Ibuprofen with Food**: To help prevent stomach upset, it's best to take ibuprofen with a small meal or snack.<br>* **Short-Term Use**: These medications are intended for short-term management of acute pain. If you find that your pain is not well-controlled or you need to take them for more than a few days to a week, you should consult with your doctor .
28	In your case—a first-time anterior shoulder dislocation that was reduced successfully and **no fractures or nerve injury on initial assessment**—you usually **do not need an MRI right away**.<br><br>### What imaging is typically done first?	For a **20-year-old** with a shoulder dislocation, the answer is almost certainly **yes**—you will likely need an MRI, but usually not on the very first day.	The short answer is that you may need an MRI, but not necessarily as an emergency. For a first-time dislocation without a fracture, the decision about advanced imaging like MRI or CT is typically made at your follow-up appointment with an orthopedic specialist, usually within a few weeks.

	* **X-rays** are standard in the emergency setting<br><br>* To confirm the shoulder is back in place<br>* To rule out fractures<br><br>If those were normal (as in your case), that’s often enough initially.<br><br>---<br><br>### When would an MRI be needed?<br><br>An MRI is not routine, but doctors may order one if:<br><br>**1. You have ongoing symptoms after a few weeks**<br><br>* Persistent pain<br>* Feeling of instability or “slipping”<br>* Limited movement that isn’t improving<br><br>**2. You’re considering surgery**<br><br>* MRI helps identify injuries like a labral tear or ligament damage<br>* It guides surgical planning<br><br>**3. You’re very active or an athlete**<br><br>* Some specialists order early MRI in young patients to assess the extent of soft tissue injury and help decide between rehab vs early surgery<br><br>**4. There’s concern something was missed**<br><br>* For example, subtle bone injuries or cartilage damage not seen on X-ray<br><br>---<br><br>### Will an MRI change your treatment right now?<br><br>In many cases, **no—not immediately**.<br>Initial treatment (rest, sling, physiotherapy) is usually the same whether or not an MRI is done right away.<br><br>---<br><br>### Bottom line<br><br>* **You likely don’t need an MRI at this stage**	While the ER took an X-ray to make sure the bones were back in place, an X-ray is "blind" to the soft tissues (cartilage and ligaments) that actually hold your shoulder together.<br><br>---<br><br>## 1. Why an X-ray isn't enough<br>X-rays are great for seeing **bones**, but they cannot see:<br>* **The Labrum:** The "rubber bumper" that deepens your shoulder socket.<br>* **Ligaments:** The "straps" that prevent the arm from sliding out.<br>* **The Rotator Cuff:** The muscles that stabilize the joint.<br><br>In your age group, the bone is rarely the problem—the **soft tissue** is what gets damaged.<br><br>## 2. The Gold Standard: MRI (or MRA)<br>An MRI is the only way to see if you have a **Bankart Lesion** (a torn labrum). This is the "signature injury" of young people who dislocate their shoulders.<br>* **MRI Arthrogram (MRA):** Sometimes, the doctor will suggest an "Arthrogram." This involves injecting a small amount of dye (contrast) into the joint before the scan. This dye fills in the cracks of any tears, making them much easier for the radiologist to see. It’s significantly more accurate for detecting labral tears than a standard MRI.<br><br>## 3. When should you get it?<br>Most surgeons prefer to wait **7 to 14 days** after the injury before doing an MRI.<br>* **Why the wait?** Right after the injury, there is too much swelling and "noise" inside the joint. Waiting a week or two allows the inflammation to settle so the doctor can get a crystal-clear picture of what is actually torn versus what is just swollen.<br><br>## 4. Do you need a CT Scan?<br>A CT scan is usually only ordered if the doctor suspects **bone loss**.<br>* If your X-ray showed a "Bony Bankart" (a piece of the socket chipped off) or a deep "Hill-Sachs" lesion (a dent in the ball), a CT scan provides a 3D map of the bone. This helps the surgeon decide if they need to use a bone graft to fix the stability.<br><br>---<br><br>## Summary: What you should do next<br>1. **See a Specialist:** Schedule an appointment with an **Orthopedic Sports Medicine Surgeon**.	Here is the breakdown of what imaging is standard now versus what might be recommended later, and why.<br><br>**Standard Imaging: You've Likely Already Had This**<br>After your shoulder was put back in place, the emergency department would have taken post-reduction X-rays. These are essential to confirm the joint is properly aligned and to rule out any fractures that might have been missed initially . X-rays are excellent for viewing bone, but they cannot show damage to soft tissues like ligaments or cartilage (the labrum).<br><br>**Advanced Imaging: MRI and CT Scans**<br>This is where the decision-making comes in, and it depends heavily on your age and activity level. An MRI is the gold standard for viewing the soft tissue "bumper" of the shoulder (the labrum) and the surrounding ligaments, which are almost always torn during a dislocation .<br><br>Given that you are 20 years old and active in sports, an orthopedic surgeon will likely discuss an MRI with you for two main reasons:<br>- **To Assess Recurrence Risk:** In young, active patients, the risk of the shoulder dislocating again is high. An MRI can reveal the specific pattern and severity of your labral tear. Some tears heal well with just rest and physical therapy, while others leave the shoulder so loose that surgery is the better option to prevent a second dislocation . The MRI provides a "road map" of the internal damage to help you and your doctor make an informed decision between continued non-surgical care or early surgery .<br>- **Timing Matters:** If an MRI is ordered, performing it within the first week or two after injury can actually provide excellent images without needing an injection of contrast dye. This is because the natural fluid and blood from the injury act as a contrast agent, making the tear very visible .<br><br>A CT scan is usually reserved for specific situations, most notably if there is a concern about **bone loss**. If the dislocation chipped off a piece of the socket (glenoid bone loss), a CT scan is the best way to measure the size of that defect, which is a critical factor in deciding if surgery is needed and what type of surgery to perform .<br><br>**What This Means for Your Next Steps**<br>You do not need to rush to get an MRI tomorrow. The immediate priority is protecting the shoulder in the sling to let the initial inflammation settle. The appropriate time to address advanced imaging is at your follow-up orthopedic appointment, which is typically scheduled **2 to 4 weeks** after the injury .

	* It becomes useful **if recovery isn’t going well, instability develops, or surgery is being considered**<br><br>---<br><br>If you want, I can explain what specific injuries (like labral tears) mean and how often they heal without surgery.	2. **Ask for the Scan:** Even if you feel "fine" in two weeks, ask: *"Since I'm only 20, should we get an MRI to check the status of my labrum?"*<br>3. **The Result Matters:** If the MRI shows a "clean" shoulder with just stretched ligaments, you can focus purely on physical therapy. If it shows a fully detached labrum, you can have a real conversation about whether surgery is necessary to prevent it from happening again.<br><br>**Quick Check:** Did the ER doctor mention seeing any "chips" or "dents" on your X-rays, or did they just say the joint was "back in place"?	When you see the specialist, they will examine your shoulder and discuss your goals for returning to sports. At that point, they can order an MRI if it's clinically necessary to guide your treatment plan .
29	Yes, you can sleep normally after a shoulder dislocation, but in the early phase it’s often more comfortable—and a bit safer for pain control—to **adjust your position**.<br><br>### Best ways to sleep in the first 1–2 weeks<br><br>**1. On your back (best option)**<br><br>* Keep your arm in a sling if your doctor gave you one<br>* You can place a small pillow under your forearm or elbow<br>* This keeps the shoulder supported and reduces strain<br><br>**2. On your uninjured side**<br><br>* Lie on the opposite shoulder<br>* Hug a pillow in front of your chest to support the injured arm<br>* This prevents the arm from falling backward or outward<br><br>---<br><br>### Positions to avoid early on<br><br>* Sleeping on the injured shoulder (can increase pain and irritation)<br>* Positions where your arm is stretched backward or rotated outward (this position is more likely to stress the healing tissues)<br><br>---<br><br>### Do you need to sleep in a sling?<br><br>* Many doctors recommend wearing the sling **especially during the first few days or at night**<br>* It helps prevent accidental movement during sleep<br>* After about 1–2 weeks, depending on pain and stability, some people can sleep without it<br><br>---	Sleeping "normally" is usually the hardest part of the first two weeks. Because you are 20, you likely move around in your sleep more than an older person, which is a major risk for a re-dislocation.<br><br>To keep your shoulder safe and actually get some rest, you need to ditch the flat-on-your-back style for a few weeks.<br><br>### 1. The Best Position: The "Lazy Boy" Recline<br>The gold standard for sleeping after a shoulder injury is at a **45-degree angle**.<br>* **Why?** When you lie flat, gravity pulls the ball of your shoulder backward, which puts immense pressure on the injured front ligaments and the labrum.<br>* **How to do it:** Use a wedge pillow or stack 3–4 firm pillows behind your back. If you have a recliner chair, sleeping there for the first few nights is often the most comfortable option.<br><br>### 2. The "Pillow Fort" (Crucial Support)<br>Even if you are reclined, your arm needs its own support.<br>* **Under the Elbow:** Place a pillow behind your tricep/elbow on the injured side. This prevents the elbow from dropping back toward the mattress, which "opens up" the front of the joint and causes sharp pain.<br>* **In front of the Chest:** Hug a small pillow against your stomach. This gives your arm a place to rest so the weight of the limb isn't pulling on the joint.<br><br>### 3. Should you wear the sling?<br>**Yes.** You should wear your sling to sleep for at least the first **2 to 3 weeks**.<br>* While you are asleep, your brain "turns off" the muscles that guard the joint. If you have a dream or roll over, you could easily reach behind your head or roll onto the arm, causing a re-dislocation.<br>* The sling acts as a "seatbelt" for your arm, keeping it in a safe, neutral position all night.<br><br>---	It is understandable to be concerned about your sleep after a shoulder dislocation. You cannot sleep in your normal position right away; you will need to use a special position and support for a period to protect the joint and ensure it heals correctly .<br><br>Here is the essential information for sleeping safely and comfortably:<br><br>* **Wear Your Sling to Bed**: You should wear the sling provided by the hospital while sleeping for the first 1-2 weeks. This includes at night, as it prevents accidental movements that could cause pain or re-injury. You can remove it for showering and dressing .<br>* **Best Position: Sleep on Your Back or Propped Up**: The safest position is sleeping on your back or propped up with pillows. This position keeps your shoulder in a neutral and stable alignment, distributing your weight evenly and avoiding any pressure on the injured joint .<br>* **Use Pillows for Support**: To make back-sleeping more comfortable and to prevent you from rolling over in the night, place a pillow under the elbow and forearm of your injured arm for support. You can also place pillows behind you or under your knees for added comfort .<br>* **If You Must Sleep on Your Side**: If you cannot tolerate sleeping on your back, lie on your **uninjured side**. Hug a pillow to your chest to support the arm of your dislocated shoulder, which prevents it from dropping forward and pulling on the healing tissues .<br>* **Positions to Strictly Avoid**:<br>* Do **not** sleep on your injured shoulder. The pressure and weight can cause significant pain and potential damage .<br>* Do **not** sleep on your stomach. This position forces your neck and shoulders into a twisted, stressful position that can strain the joint .

	### What to expect<br><br>* It’s normal for sleep to be uncomfortable at first<br>* Pain usually improves significantly over 1–2 weeks<br>* Pain medication before bed (like paracetamol or ibuprofen if safe for you) can help<br><br>---<br><br>### Bottom line<br><br>You don’t need a special rigid position, but **back or uninjured-side sleeping with support is best early on**, and a sling at night can help protect your shoulder while it heals.<br><br>If you want, I can also tell you how long the sling is typically needed and when you can start moving the shoulder safely.	### Positions to ABSOLUTELY Avoid:<br>* **The "Superhuman" (Arm over head):** Never sleep with your arm tucked under your pillow or stretched above your head. This is the most unstable position for the shoulder.<br>* **Side Sleeping (Injured Side):** Obviously, do not lie directly on the injured shoulder.<br>* **Side Sleeping (Healthy Side):** Be careful here. If you sleep on your good side, the injured arm tends to "drape" across your body, which pulls the joint out of alignment. If you must sleep on your good side, you **must** hug a large body pillow to keep the injured arm elevated and parallel to the bed.<br><br>---<br><br>### Tips for Better Sleep:<br>1. **Medicate 30 minutes before bed:** Take your Naproxen (Aleve) or Tylenol about a half-hour before you plan to sleep so it’s at full strength when you close your eyes.<br>2. **Ice before bed:** Apply an ice pack for 20 minutes right before laying down to numb the joint and settle the inflammation from the day's movements.<br>3. **Tighten the sling:** Make sure the sling is snug enough that your arm can't slide out the back of it while you toss and turn.<br><br>**The "Expert Guide" Question:**<br>Do you usually sleep on your back, or are you a side-sleeper? Knowing your habit can help me suggest a specific pillow setup to keep you from rolling over.	In the initial nights, you may find it most comfortable to sleep in a semi-upright position, propped up with several pillows. This can be easier to manage than lying completely flat .<br><br>Your first-time shoulder dislocation is expected to heal well with proper care, and how you sleep is a key part of that process. Focus on protecting the joint by sleeping on your back and keeping it supported for the first couple of weeks. Once you are out of the sling, you can gradually return to your normal sleeping habits, but be sure to follow the guidance of your doctor or physiotherapist.
30	In the early period after a first anterior shoulder dislocation, **it’s generally not recommended to sleep on the injured shoulder**.<br><br>### Why it’s not a good idea at first<br><br>* The tissues (labrum and ligaments) are **stretched or torn and still healing**<br>* Lying on that side can:<br><br>* Increase pain<br>* Put pressure on the joint<br>* Encourage positions that stress the shoulder (slight outward rotation or backward movement), which is the position where dislocations often happen<br><br>### When it becomes safer<br><br>* After the first **1–3 weeks**, once pain settles and your doctor/physiotherapist confirms stability	Short answer: **No, absolutely not.** At least for the first **4 to 6 weeks**, you should avoid sleeping directly on the injured shoulder. While it might be your favorite side, putting your full body weight on a recently dislocated joint is a recipe for pain and potential re-injury.<br><br>Here is why sleeping on that side is a bad idea right now:<br><br>### 1. The "Crush" Factor<br>When you lie on your shoulder, you are compressing the **labrum** and **ligaments** that are currently trying to knit back together. This pressure restricts blood flow to the exact area that needs it most for healing.<br><br>### 2. Inflammation & Throbbing<br>Pressure on an inflamed joint increases the "throbbing" sensation. You’ll likely find that even if you manage to fall asleep on that side, you’ll wake up 20 minutes later with sharp, pulsing pain because the internal swelling has nowhere to go.<br><br>### 3. The Risk of "Subluxation" (Partial Dislocation)	No, it is **not okay** to sleep on your injured shoulder at this stage of recovery.<br><br>Here is exactly why you should avoid it and what it can cause:<br><br>- **Mechanical Stress:** Even though the joint is back in place, the ligaments and labrum (the cartilage rim) were stretched or torn during the dislocation. Lying on that side compresses the joint, which can cause the ball of the humerus to slide partially backward or forward, stressing the repair tissue.<br>- **Pain and Stiffness:** Sleeping directly on the injured side restricts blood flow to the area. This can cause you to wake up with significantly more pain and "frozen" stiffness in the morning compared to sleeping on your back.<br>- **Nerve Compression:** The shoulder area has several nerves (the brachial plexus) that are often bruised after a dislocation. Direct pressure from your body weight can cause your arm or fingers to tingle or "fall asleep" more intensely.

	* Gradually, many people can return to sleeping on that side as comfort allows<br>* It should feel **comfortable and stable**, not painful or “unstable”<br><br>### What to do instead for now<br><br>* Sleep on your **back** with your arm supported by a pillow, or<br>* Sleep on your **uninjured side**, hugging a pillow to support the injured arm<br>* If you were given a sling, using it at night in the early days can help prevent accidental movement<br><br>### Bottom line<br><br>Sleeping on the injured shoulder is **best avoided initially**, but it is usually **safe again later in recovery once pain and instability have improved**.<br><br>If you want, I can give you a rough timeline of what movements and sleeping positions are usually safe week by week after this type of injury.	When you are asleep, your muscles are totally relaxed. If you lie on the injured side, the bed pushes the ball of the humerus upward while your body weight pushes the socket downward. Without your muscles "guarding" the joint, the bone can easily slip into an unstable position.<br><br>---<br><br>### When can I sleep on that side again?<br>Usually, you have to wait until:<br>* **The "Apprehension" is gone:** You can move your arm without feeling like it’s going to pop out.<br>* **Rotator Cuff Strength is back:** You’ve done enough physical therapy that your muscles can "hold" the joint even when you're relaxed.<br>* **Doctor's Clearance:** Typically after your 6-week follow-up.<br><br>### How to stay off that side (The "Log" Trick)<br>Since you're 20, you probably toss and turn. If you are a natural side-sleeper and keep rolling onto the "bad" side:<br>* **The Back-Stop:** Place a long **body pillow** or a rolled-up heavy blanket behind your back. If you try to roll onto your injured side in the middle of the night, the pillow acts as a "speed bump" and keeps you on your back or slightly tilted.<br>* **The "Hug" Position:** If you sleep on your **healthy** side, hug a large pillow to your chest. Rest your injured arm on top of that pillow. This keeps the injured shoulder from "dropping" forward and pulling on the socket.<br><br>### A Wit's Warning<br>Sleeping on your injured shoulder right now is like trying to heal a broken toe by walking on it in high heels—it’s technically possible to do, but you’re going to regret every second of it.<br><br>**Pro-tip:** If you find yourself waking up on your side anyway, try sleeping in a **recliner** or propped up in a "pillow throne" for a few more nights until the initial "looseness" of the joint settles down.<br><br>Do you feel like you're rolling onto that side accidentally, or is it just really hard to get comfortable on your back?	**When can you sleep on that side again?**<br>You generally should wait until you are **out of the sling** and have been cleared by a doctor or physiotherapist to start range-of-motion exercises. Even then, it will likely be **4 to 6 weeks** before lying flat on that shoulder feels comfortable. If you try before that time, your body will likely wake you up immediately with a sharp ache as a warning sign.<br><br>For now, please continue to sleep on your back or your **uninjured** side with a pillow hugged to your chest
31	Yes—you can shower or bathe after a shoulder dislocation, but you need to be a bit careful in the early days.<br><br>### Showering<br><br>**Usually safe**, and it’s actually encouraged for hygiene.<br><br>* You can remove the sling briefly if your doctor hasn’t told you otherwise	You can definitely stay clean, but you need to treat your shower like a tactical mission for the first week. Because you’re **20**, your instinct might be to just hop in and scrub away, but a slippery floor and a heavy, "unstable" arm are a dangerous mix.<br><br>Here is the safest way to handle it:<br><br>### 1. The Shower Is Safer Than the Bath<br>Avoid taking a bath for the first week or two.	This is a very important question because water and slippery surfaces pose a real risk to a healing shoulder. The short answer is: **You can shower, but you must take specific precautions. You should avoid taking a bath until you are out of the sling.**<br><br>Here is exactly how to manage washing safely with a first-time anterior shoulder dislocation.<br><br>### Showering: Yes, With Strict Rules

	* Keep your injured arm **close to your body**<br>* Avoid reaching, stretching, or lifting the arm while showering<br>* Let the water run over the shoulder gently—this is fine<br><br>**Tip:** It often helps to stand with your back or uninjured side facing the water.<br><br>---<br><br>### Bathing<br><br>* Also safe, but **less practical** in the first days<br>* Getting in and out of a bathtub can risk slipping or sudden arm movement<br>* If you do take a bath, keep the injured arm supported and avoid leaning on it<br><br>---<br><br>### Important precautions<br><br>* Avoid **slippery floors** (falling is the main risk, not the water itself)<br>* Don’t force the arm into any position that causes pain<br>* If you still have a sling, put it back on carefully after drying<br><br>---<br><br>### When to be extra careful<br><br>* First few days after injury (pain is higher, shoulder is less stable)<br>* If you feel any “looseness” or instability during movement<br><br>---<br><br>### Bottom line<br><br>Yes, you can shower and even bathe, but **move slowly, keep the arm close to your body, and avoid using the injured shoulder actively**.<br><br>If you want, I can also explain when you can safely start washing your hair normally or lifting your arm in the shower.	* **Why?** Getting in and out of a bathtub requires you to push off or pull yourself up with your arms. If you accidentally put weight on your injured arm to steady yourself, you could re-dislocate it instantly.<br>* **The Rule:** Stick to standing or sitting in a shower.<br><br>### 2. The "Dead Arm" Technique<br>When you take your sling off to wash, your arm is going to feel heavy and "shaky."<br>* **Do NOT** lift your arm up to wash your armpit.<br>* **The "Lean" Method:** Keep your injured arm relaxed at your side. Slightly lean your upper body forward and toward the injured side. Gravity will cause your arm to naturally "dangle" away from your body. You can then use your good hand to wash under the arm without using your shoulder muscles.<br><br>### 3. Essential Safety Tips<br>* **The "No-Reach" Zone:** Do not reach behind your back to wash or reach up for the shampoo bottle with your injured hand. This "reaching back and up" move is the #1 way shoulders pop back out.<br>* **Non-Slip Mat:** This is the most important part. If you slip in the shower, your natural reflex will be to throw both arms out to catch yourself. That sudden jerk is extremely high-risk for your shoulder.<br>* **Keep it Lukewarm:** Very hot water increases blood flow and can actually make the swelling and throbbing in your shoulder worse. Stick to a comfortable, warm temperature.<br><br>### 4. How to Dry Off<br>Pat yourself dry with a towel rather than using a vigorous "sawing" motion. If you have someone who can help you dry your back or your good arm, don't be afraid to ask.<br><br>### 5. Managing the Sling<br>You obviously shouldn't wear your "good" sling in the shower (it will get soggy and gross).<br>* **The "Naked" Option:** Some people feel stable enough to shower without any support as long as they keep the arm tucked against their stomach.<br>* **The "Shower Sling":** If you feel very unstable, you can buy a cheap mesh sling specifically for the shower, or even use a long piece of gauze or a plastic bag tied in a loop just to take the weight of the arm off the joint while you wash.<br><br>---<br><br>### The "Expert Guide" Checklist<br>1. **Prepare first:** Open all your shampoo/soap bottles before you take the sling off.	You are allowed to shower, but you must keep the arm completely immobile. The sling is not waterproof, so you will take it off for the shower.<br><br>**The Critical Rule: Do Not Let the Arm Dangle**<br>When you remove the sling, gravity pulls down on the arm. This puts traction on the injured ligaments and can cause the shoulder to sublux (slide partially out) or spasm painfully. You must keep the elbow bent at 90 degrees and the arm supported by your body.<br><br>**Safe Showering Technique (The "Hand in Pocket" Method):**<br>1. **Undress Carefully:** Undress your uninjured arm first, then slide the sling off the injured arm last. Keep the injured arm bent and resting against your stomach/chest.<br>2. **Position:** Face the showerhead. Do not turn your back to the water or try to wash your back with the injured arm.<br>3. **Arm Placement:** Imagine your hand is stuck in your front pants pocket. Keep the forearm horizontal across your belly. **Do not let the elbow drift backward or away from your body.**<br>4. **Washing:** Use your **good hand** to do all the washing. A long-handled shower brush or loofah on a stick is very helpful for reaching your good armpit and legs.<br>5. **Drying:** Pat dry with the towel using one hand. Do not "fling" the towel over your shoulder to dry your back; just dab the area you can reach.<br><br>### Bathing: No, Not Recommended<br>You should **not** take a bath or use a hot tub at this stage.<br>- **Getting In/Out Danger:** Getting out of a bathtub requires you to push up with both arms. Even if you try to use just one arm, the instinct to push or catch yourself if you slip will cause a violent contraction in the injured shoulder, which can easily cause a re-dislocation.<br>- **Position:** Lying back in a tub often requires you to lean on the injured elbow or shoulder.<br><br>### What About the Armpit?<br>You will notice that keeping the arm tight to your side makes the armpit sweaty and damp. This can lead to skin irritation or rash.<br>- **Solution:** After drying off, place a dry, thin piece of gauze or a clean, dry washcloth **between your arm and chest** (inside the armpit area) **before** putting the sling back on. This wicks away moisture. Change this cloth daily.<br><br>### When to Put the Sling Back On

		2. **Wash the "Good" side first:** This gets the hardest part out of the way while you're still focused.<br>3. **Be quick:** The longer you stand there without support, the more your shoulder muscles will fatigue.<br><br>Does your shoulder feel stable enough to let it hang at your side for a few minutes, or does it feel like it "needs" to be held up right now?	Put the sling back on **immediately after drying off**, while still seated. Apply the sling first, then dress your injured arm, then dress your good arm. If you have a simple immobilizer sling (not a complicated shoulder brace), it is often easiest to wear a loose button-down shirt or zip-up hoodie backwards over the sling for the rest of the day rather than trying to wrestle a t-shirt over your head.
32	Yes—**you can and should use your arm for light, everyday activities**, as long as you keep movements gentle and avoid positions that stress the shoulder.<br><br>After a first anterior shoulder dislocation, the goal in the early phase is usually **“protected movement”**: staying active enough to avoid stiffness, but not moving the shoulder in risky ways.<br><br>---<br><br>### Safe activities (generally OK early on)<br><br>**Eating**<br><br>* Yes, this is fine<br>* Keep the elbow close to your body<br>* Avoid reaching far across your body or lifting your arm high<br><br>**Brushing teeth / washing face**<br><br>* Yes<br>* Do it slowly and keep movements small<br>* Try not to lift the elbow away from your side too much<br><br>**Using your phone / computer**<br><br>* Yes<br>* Best if your arm is supported (on a pillow, armrest, or lap)<br>* Avoid holding your arm up in the air for long periods<br><br>---<br><br>### What to avoid in the early days<br><br>These positions put the shoulder at higher risk of instability:<br><br>* Reaching the arm **out to the side and rotating it outward**<br>* Lifting your arm **above shoulder level**<br>* Sudden or forceful movements<br>* Carrying anything heavy in the injured arm<br>* Supporting your full body weight on that arm	The short answer is **yes, but with a very strict "short-leash" policy.** At 20, you’re likely used to moving fast, but for the next **2 to 3 weeks**, you need to treat your injured arm like it’s "tethered" to your ribs. You can use your **hand and wrist**, but you should avoid moving your **shoulder** to do it.<br><br>Here is the breakdown of how to handle daily tasks safely:<br><br>### 1. Using Your Phone (Safe-ish)<br>* **The Rule:** Keep your elbow tucked against your side.<br>* **The Technique:** Bring your phone to your face, not your face to your phone. Use your healthy hand to hold the phone and your injured hand just for light scrolling/typing while your forearm rests on your stomach or a pillow.<br>* **Avoid:** Holding the phone up to your ear with the injured arm. That "arm-up-and-out" position is the most dangerous spot for a loose shoulder. Use **speakerphone or earbuds** instead.<br><br>### 2. Brushing Your Teeth (The "T-Rex" Method)<br>* **The Rule:** If you are right-handed and it’s your right shoulder, **switch to your left hand** for a week.<br>* **The Technique:** If you *must* use the injured side, keep your elbow pinned to your torso. Move your head back and forth rather than moving your arm.<br>* **Avoid:** Reaching up and across your face.<br><br>### 3. Eating and Drinking<br>* **The Rule:** Use your hand, not your shoulder.<br>* **The Technique:** Lean forward slightly toward your plate so you don't have to lift your hand as high. Rest your forearm on the table.<br>* **Avoid:** Reaching across the table for the salt or a napkin. If it’s more than 6 inches away, use your "good" arm.<br><br>### 4. Typing and Computer Work<br>* **The Rule:** Forearms must be supported.<br>* **The Technique:** If you’re at a desk, pull your chair in close so your forearms rest completely on the desk surface. This prevents the weight of your arm from pulling down on the shoulder socket.<br>* **Avoid:** Using a mouse that requires you to reach far out to the side.	This is an excellent question because the answer is not simply "yes" or "no"—it depends entirely on **how** you move the arm. There is a crucial distinction between moving the **elbow and hand** (which is safe) and moving the **shoulder joint** (which is not safe right now).<br><br>Here is the clear guidance for eating, brushing teeth, and using your phone.<br><br>### The Golden Rule: The Elbow Must Stay Glued to Your Side<br>For the first 1 to 2 weeks (or until your follow-up appointment), your shoulder blade and upper arm bone should not move independently. Think of your arm as a robot arm that is **frozen at the shoulder hinge** but works fine from the elbow down.<br><br>- **Safe Movement:** Elbow bending, wrist turning, finger wiggling.<br>- **Unsafe Movement:** Lifting the elbow away from your body, reaching forward, or rotating the shoulder outward.<br><br>### Eating<br>**Yes, you can eat with that hand, but with strict modifications.**<br>- **The Setup:** Do not sit at a high table or counter where you have to lift your elbow to reach your mouth. You need to **bring the food to you**.<br>- **The Technique:** Keep your elbow pinned to your ribs/side. Lean your head **down and forward** slightly to meet the fork or spoon. **Do not lift the elbow up** to bring the fork to a straight head.<br>- **Cutting Food:** You cannot use that hand to hold a knife or steady a plate. You will need to eat "one-handed" style meals (soups, stews, pre-cut pasta, sandwiches) or have someone cut your meat for you.<br><br>### Brushing Teeth<br>**Yes, but be careful with the toothpaste.**<br>- **Brushing:** Use the same principle as eating. Keep the elbow tucked at your side. Bring your **head down** toward the brush, do not bring the brush **up** to your head. This prevents the small, painful rotation of the shoulder that happens when you lift the elbow even a few inches.

	---<br><br>### About the sling<br><br>* If you were given a sling, it’s mainly for **protection and comfort**<br>* You can usually remove it briefly for hygiene and light activities<br>* Put it back on when walking around or resting early on<br><br>---<br><br>### Bottom line<br><br>Yes—you can use your arm for **basic daily tasks**, and it’s actually helpful for recovery. Just keep the shoulder **close to your body, relaxed, and avoid stretching or overhead movements**.<br><br>If you want, I can give you a simple “safe vs unsafe movements” list for the first 2–3 weeks so you know exactly what to avoid.	---<br><br>### The "Golden Rule" of Daily Tasks<br>**If your elbow leaves your side, you are taking a risk.** In the medical world, we call the safe zone your **"Functional Cylinder."** Imagine you are standing inside a narrow tube or a phone booth. Anything you can do with your hands *inside* that tube without hitting the walls is generally okay. Anything that requires you to reach "outside the tube" (up, out to the side, or behind you) is a no-go.<br><br>### Why be so strict?<br>Even "simple" things like reaching for a heavy coffee mug can cause a sudden muscle twitch. If your labrum is torn, that tiny twitch can be enough to make the joint shift, causing pain and resetting your healing clock.<br><br>**Pro-Tip:** If you feel "aching" in your bicep or the side of your shoulder after using your phone for 10 minutes, it’s a sign your muscles are fatigued. Put the arm back in the sling and give it a break.<br><br>Which arm did you injure—your dominant one (the one you write with) or your "off" hand?	- **The Spit Step:** **Do not lean over the sink and support your body weight with the injured arm.** This is a common way people re-injure the shoulder in the first week. Stand up straight, keep the injured arm limp at your side, and bend only at the waist/hips.<br><br>### Using Your Phone<br>**Yes, but only if the elbow is supported.**<br>- **Safe:** Holding the phone directly in front of your belly button while sitting. Your elbow should be resting on your lap or a pillow in your lap.<br>- **Unsafe:** Holding the phone up to your ear with the injured arm. This requires lifting the elbow away from the body and externally rotating the shoulder (the exact position of dislocation).<br>- **Unsafe:** Lying on your back in bed and holding the phone above your face with the injured arm. This puts the weight of the arm and gravity against the healing labrum.<br><br>### Important Warning Sign to Watch For<br>If you feel a **dull ache deep in the shoulder joint** or a **sharp pinch** in the front of the shoulder while doing any of these small tasks, **stop immediately**. That is the joint telling you that you moved the shoulder socket even a millimeter more than it is ready for.<br><br>**Summary for Daily Tasks:** Use the hand, **do not** use the shoulder. If the task requires the elbow to leave your ribs, use your **uninjured arm** instead.
33	In most cases, **yes—you can go back to school after a shoulder dislocation**, as long as your pain is manageable and your shoulder is protected.<br><br>### Why it’s usually okay<br><br>A first-time anterior shoulder dislocation (like yours, with no fracture or nerve injury) is typically treated with:<br><br>* Sling (for a short period)<br>* Pain control<br>* Early gentle movement + later physiotherapy<br><br>School activities are generally **low-risk** if you avoid using the injured shoulder.<br><br>---<br><br>### When it is safe to return	You can definitely go back to school, but you need to approach it like an "injured athlete" rather than a regular student for the next few weeks. At **20**, you’re likely navigating a campus, heavy doors, and crowded hallways—all of which are "high-risk environments" for a loose shoulder.<br><br>Here is your survival guide for heading back to class:<br><br>### 1. The "Crowded Hallway" Rule<br>The biggest risk at school isn't your schoolwork; it's **other people**. A sudden bump from someone in a crowded hallway or a "high-five" from a friend who doesn't know you’re injured can easily jar the shoulder out of place.<br>* **The Strategy:** Always wear your **sling** in public, even if you feel okay. It acts as a "visual warning" to others to give you space.<br>* **Pro-Tip:** Leave class two minutes early or wait five minutes after the bell to avoid the "crush" in the hallways.<br><br>### 2. Backpacks are the Enemy	Yes, it is generally okay to go back to school, but you need to plan for **protection** and **assistance**. Because you are 20, I am assuming this is likely a college or university setting (large campus, crowded hallways, heavy backpack), but the same principles apply to any school environment.<br><br>Here is what you need to consider to stay safe and comfortable during the recovery period.<br><br>### The Biggest Risk: The Hallway and Crowds<br>The most dangerous part of your day will not be sitting in class; it will be moving between classes.<br>- **Bumping:** People in a hurry will not notice your sling. A sudden bump or jostle to your arm or shoulder can cause a severe muscle spasm and pain, even if it doesn't dislocate the joint again.<br>- **Instinctive Reactions:** If someone bumps you, your natural reflex is to catch yourself or swing your arm out for balance. You must consciously override this reflex.

	You can usually go back when:<br><br>* Pain is controlled with simple painkillers (like paracetamol or ibuprofen)<br>* You can sit and move around comfortably<br>* You can keep the arm protected in a sling (if still prescribed)<br><br>For many people, this is **within a few days to a week** after the injury.<br><br>---<br><br>### How to manage school safely<br><br>**At school:**<br><br>* Keep your arm in a sling if you were given one<br>* Sit where your arm won't get bumped (e.g., aisle or corner seat)<br>* Ask for help carrying your bag<br>* Avoid physical education or sports completely for now<br><br>**In class:**<br><br>* You can write, use your phone, and take notes if comfortable<br>* If writing is painful, you may need adjustments (extra time or typing instead)<br><br>---<br><br>### What to avoid at school<br><br>* Sports, running, or physical education<br>* Carrying heavy backpacks on the injured side<br>* Crowded situations where your shoulder could be bumped<br>* Sudden arm movements or reaching overhead<br><br>---<br><br>### When to wait a bit longer<br><br>Consider delaying return if:<br><br>* Pain is still strong even at rest<br>* You cannot sit comfortably for long periods<br>* You feel the shoulder is unstable even in a sling<br><br>---<br><br>### Bottom line	You cannot wear a backpack normally. Putting a strap over your injured shoulder puts direct downward pressure on the joint, and the act of "shrugging" a backpack on is a classic way to re-dislocate.<br>* **The Fix:** Carry a light messenger bag on your **healthy** shoulder, use a rolling bag, or just carry a single notebook. If you have heavy textbooks, see if you can access digital versions for now.<br><br>### 3. Taking Notes<br>If you injured your **dominant** arm (the one you write with), you are going to have a hard time.<br>* **The Fix:** Don't try to "power through" writing with a heavy arm. Use a laptop with your forearms supported on the desk, or use a voice-recording app for lectures (with the professor's permission).<br>* **The "Leaning" Rule:** When sitting at a desk, don't lean your body weight onto your injured elbow. It pushes the bone up into the "angry" part of the socket.<br><br>### 4. Talk to your Instructors (or Disability Services)<br>You aren't being "extra"—you have a legitimate physical limitation.<br>* **What to ask for:** Extra time for exams (if you write slowly), a more ergonomic chair, or permission to leave class early to avoid the hallway rush. Most universities can provide a temporary "medical accommodation" letter if you show them a note from the ER or your specialist.<br><br>### 5. Fatigue is Real<br>Managing a dislocation is physically exhausting. Your brain is constantly "on guard" to protect the joint, and your muscles are working 24/7.<br>* **The Advice:** If you have a long day of classes, plan "ice breaks" in the student lounge or the nurse's office. If the pain starts throbbing, it's your body's way of saying you've done too much.<br><br>---<br><br>### **The "Going Back" Timeline**<br>* **Days 1–3:** Stay home. The pain and the "brain fog" from painkillers make it hard to focus.<br>* **Days 4–7:** Return to class, but only for half-days if possible.<br>* **Week 2+:** Full days are usually fine, provided you are disciplined about your sling and avoiding "reaching" movements.<br><br>**One final tip:** If you commute by bus or train, try to get a seat. Standing on a moving vehicle requires you to hang onto a rail or "brace" yourself—both of which are very dangerous for a fresh shoulder injury.<br><br>Are you in high school or college? The size of the campus might change how you plan your "commute" between classes.	**How to Manage This:**<br>- **Walk with your injured side facing the wall.** Keep your good side facing the flow of traffic.<br>- **Leave class 2-3 minutes late or early** to avoid the crush of the crowd.<br>- **Do not use stairs in a crowd.** Use the elevator or wait until the stairwell is empty so you can hold the railing with your **good** hand without being pushed.<br><br>### The Backpack Problem<br>**You cannot wear a standard two-strap backpack.** Pulling a strap over the injured shoulder is impossible right now, and carrying weight on one shoulder throws off your posture, causing neck and back pain.<br><br>**Solutions:**<br>1. **Rolling Backpack:** This is the gold standard for shoulder injuries. It allows you to move around campus with zero weight on the body.<br>2. **Crossbody Bag (on the UNINJURED shoulder):** A small messenger bag worn across the **good** shoulder is acceptable for a laptop or a single notebook. Ensure the strap does not cross near the injured side.<br>3. **Digital Only:** If possible, use a tablet or phone for notes and leave books at home for the first week.<br><br>### Taking Notes and Sitting in Class<br>- **Desk Choice:** Look for a **left-handed desk** if your injury is on the **right** side (or vice versa). This allows you to rest the elbow of your injured arm on the larger, open side of the desk rather than having it hang off the edge. If no such desk exists, sit at the end of a long table.<br>- **Writing:** You can write with the injured hand (since the elbow is bent and the movement is in the fingers/wrist), but it may tire quickly. It is a good idea to ask the professor or disability services office for permission to **audio record lectures** or use a **voice-to-text app** on your phone for the first week or two.<br><br>### Fatigue Factor<br>Healing a ligament injury requires significant energy. You will likely feel **exhausted** by midday, even if you are just sitting in class. The constant, low-grade muscle guarding (your body subconsciously tensing up to protect the joint) is draining. Do not plan long library sessions or social events after school for the first few days. Go home and rest.<br><br>### When You Should Stay Home<br>You should consider staying home from school if:

	Yes, going back to school is usually fine and even encouraged, as long as you **protect the shoulder, avoid sports, and limit strain**.<br><br>If you want, I can help you figure out exactly when you can return to sports and gym class, which is usually the more restricted part of recovery.		- You are taking strong pain medication (opioids) that makes you drowsy or unable to focus.<br>- You have a long, icy walk or commute where you are at risk of falling.<br>- You cannot figure out a safe way to transport your books/laptop.
34	You should be **careful with backpacks early after a shoulder dislocation**, especially in the first 1–3 weeks.<br><br>### In the early phase (first days to ~2 weeks)<br><br>* **Avoid carrying a backpack on the injured shoulder**<br>* Even light weight can:<br><br>* Pull the shoulder downward<br>* Increase pain<br>* Stress healing ligaments and labrum<br><br>If you need to carry a bag:<br><br>* Use the **uninjured shoulder only**, or<br>* Use a **light bag held in the hand on the uninjured side**, or<br>* Ask someone to help you carry it<br><br>---<br><br>### If you must use a backpack<br><br>Only if pain is mild and your doctor allows it:<br><br>* Wear it on **both shoulders (not one side only)**, but still keep weight very light<br>* Tighten straps so it doesn’t swing or pull<br>* Avoid putting it on or taking it off quickly (those movements can strain the shoulder)<br><br>---<br><br>### Safer alternatives for school<br><br>* Leave heavy books at school if possible<br>* Use digital notes or lighter materials<br>* Ask for assistance with carrying items<br><br>---<br><br>### When it becomes safer	The short answer is **no, you should not put a backpack strap over your injured shoulder** for at least the next **4 to 6 weeks**.<br><br>At 20, you probably live out of your backpack, but right now, that bag is a "weight and lever" system that is perfectly designed to pull your shoulder joint apart.<br><br>Here is why you need to change your bag habits immediately:<br><br>### 1. The "Traction" Problem<br>A backpack works by pulling down on the top of your shoulder. Because your ligaments are currently stretched or torn, your shoulder doesn't have its "natural tape" to hold the bone up. The weight of a heavy bag creates **traction**, which pulls the ball of the arm bone down and out of the socket. This can cause severe pain and prevent your labrum from healing.<br><br>### 2. The "Swing" Danger<br>The most dangerous moment for a shoulder is the "swing" — that move where you grab one strap and whip the bag around onto your back. That motion involves **external rotation and abduction**, which is the exact "danger zone" position that causes a shoulder to pop out.<br><br>### 3. Crowded Hallways<br>A backpack makes your "footprint" larger. If someone bumps into your bag in a crowded hallway, it creates a "lever effect" that jerks your shoulder backward.<br><br>---<br><br>### Safe Alternatives for School<br>Since you still need to get your books to class, here are the "ortho-approved" ways to do it:<br><br>* **The "One-Shoulder" Messenger Style:** Carry a light bag using only your **healthy** shoulder. Use a cross-body strap if possible to distribute the weight across your chest and away from the injury.<br>* **The "Rolling" Bag:** It might not be the most "stylish" look for a 20-year-old, but a rolling suitcase or backpack is the safest way to move heavy books without risking your joint.	No, **you cannot carry a traditional backpack or shoulder bag using your injured side.** In fact, attempting to carry weight on or across the injured shoulder is one of the most common ways young people accidentally aggravate a first-time dislocation.<br><br>Here is the detailed guidance on what you can and cannot do with bags right now.<br><br>### What You Absolutely Cannot Do<br><br>1. **Two-Strap Backpack (Worn Normally):** This is strictly off-limits. Putting the strap over the injured shoulder requires you to reach back and externally rotate the arm—the exact motion that risks re-dislocation. Additionally, the weight of the bag pulling down on the healing joint is harmful.<br>2. **Single-Strap Bag on the Injured Shoulder:** Do not sling a purse, messenger bag, or tote bag over the injured shoulder. Even a "light" bag causes the trapezius and deltoid muscles to fire constantly to keep the strap from sliding off. This muscle tension pulls on the injured capsule and causes pain and swelling.<br>3. **Crossbody Bag Across the Injured Side:** If the strap crosses from your good shoulder down to your injured hip, the weight of the bag will swing and hit your injured arm with every step. The constant vibration and impact are very uncomfortable.<br><br>### What You CAN Use (The Safe Alternatives)<br><br>Since you are 20 and likely need to carry a laptop or books, you have three safe options. Choose based on how much you need to carry.<br><br>**Option 1: Rolling Backpack (Best for Heavy Loads)**<br>This is the only way to carry a full day's worth of books and a laptop without causing pain. It allows the ground to do the work. You pull it with your **good hand**. Do not try to pull it with the injured hand, even if it feels okay for a moment—the twisting motion of the wrist can translate up to the shoulder.<br><br>**Option 2: Crossbody Bag on the GOOD Shoulder (Best for Light Loads)**

<table>
<tr><td></td><td>* As pain improves and rehab progresses (often after **2–4 weeks**), light backpack use may be allowed * Full, normal carrying usually returns gradually after strengthening begins in physiotherapy --- ### Bottom line Right now, **you should avoid carrying a backpack on the injured shoulder**, especially if it has any weight. Use the uninjured side or reduce load until your shoulder feels stable and your doctor/physiotherapist clears you. If you want, I can tell you what activities typically come back week by week so you know what to expect in recovery.</td><td>* **The "Locker Strategy":** Minimize what you carry. Ask your teachers if you can keep a second set of books at home or use digital versions so you only have to carry a single tablet or notebook. * **The "Helper" System:** If you have a long trek across campus, don't be afraid to let a friend carry your heavy bag for a few days. ### If you *must* use a backpack: If you have no other choice, follow these strict rules: 1. **Lighten the Load:** Keep it under 5 lbs (basically just a laptop and a thin notebook). 2. **Both Straps (eventually):** Once your doctor says you can stop using the sling, always use **both straps** and tighten them so the bag is high and tight against your upper back. This prevents it from "sagging" and pulling on the joint. 3. **The "Strap-On" Assistance:** Have someone else lift the bag and place the straps on your shoulders for you so you don't have to "swing" it. > **Bottom Line:** For the first 2 weeks, your injured shoulder should carry **zero** weight. Your focus is on letting those tissues tighten back up. Are your classes spread far apart across a big campus, or is everything in one building? That will help determine if you can get away with just carrying a single folder.</td><td>- **The Setup:** Place the strap on the shoulder of your **uninjured arm**. - **The Bag Position:** The bag itself should hang on the **side of your uninjured hip** (the same side as the strap). - **Why this works:** Your injured arm remains completely free. You can use your good hand to stabilize the bag while walking. - **Caution:** Keep it light (just a tablet, wallet, and keys). A heavy bag on one side of the body will cause you to lean, which can make your neck and back sore. **Option 3: The "Slide" Tote (Emergency Use Only)** If you only have a tote bag or a single-strap backpack, **do not put the strap on your shoulder.** Instead, hold the strap in your **good hand** and let the bag hang straight down like a briefcase. This is awkward for long walks but safe for moving from the car to a locker or classroom. ### Summary Comparison for You | Bag Type	Verdict	Why?
Normal Backpack	**NO**	Requires reaching back; weight on healing joint.
Injured Side Sling Bag	**NO**	Muscle guarding causes spasm and pain.
Good Side Crossbody	**YES**	Keeps injured side completely free.
Rolling Bag	**BEST**	Zero stress on the upper body. | **One Final Tip:** If you must wear a jacket, do not try to put the injured arm through the sleeve. Drape the jacket over the injured shoulder like a cape, and put your good arm through the other sleeve. This keeps you warm without moving the joint.</td></tr>
<tr><td>35</td><td>In the early phase after a shoulder dislocation, **you should be very cautious with carrying anything in the injured arm**. The main concern is not just weight, but the *pulling force* on a healing shoulder.

Short answer

* **Light, close-to-body use:** sometimes okay
* **Carrying bags, books, or shopping bags:** generally **avoid at first**

What is usually safe early on (first 1–2 weeks)</td><td>The short answer is **no**. For at least the first **2 to 4 weeks**, you should not carry any weight with your injured arm.

Think of your shoulder right now like a house with a damaged foundation. Even a "light" grocery bag acts like a weight at the end of a lever, pulling the ball of your arm bone down and away from the socket.

Here is why you need to keep that hand empty:

1. The "Traction" Effect
When you hold a bag by your side, gravity creates **traction**. Since your ligaments are currently stretched or torn, they can’t "hold the fort." The weight pulls the joint apart, stretching the healing</td><td>The direct answer is **no, you should not carry anything with the injured arm.** Even a light item creates a lever that pulls directly on the healing ligaments inside the shoulder socket.

Here is the breakdown of why this is different from just holding your phone, and what is safe versus unsafe.

The "Dead Weight" Effect
When you hold something in your hand with the elbow straight or slightly bent, gravity pulls the weight down. Because your shoulder joint is unstable right now, the muscles are the only thing holding the ball in the socket. If you add a bag of groceries or a book to the end of that lever (your arm), the weight can literally **pull the ball of the shoulder joint slightly downward and forward** away from the socket. This is called **inferior</td></tr>
</table>

You can use the injured arm for:

* Very light objects (like a phone or a light water bottle)
* Holding something briefly **close to your body**
* Activities where the arm is **supported and not bearing weight**

Key rule: the elbow should stay **close to your side**.

What you should avoid for now

These increase risk of pain or instability:

* Carrying **shopping bags**
* Holding **school books or heavy items**
* Letting the arm hang with weight pulling it down
* Carrying anything that causes the shoulder to stretch or feel “pulled”

Even moderate weight can stress the healing structures (like the labrum and ligaments).

Why this matters

After an anterior dislocation, the shoulder is temporarily:

* Less stable in certain positions
* More vulnerable when the arm is extended or loaded
* Healing soft tissues are still “tightening up”

Safer alternatives

* Use your **uninjured arm**
* Use a **backpack evenly on both shoulders (very light load) if allowed**
* Ask for help with heavier items
* Keep loads close to your body (not hanging at arm’s length)

When you can gradually carry more

* Light carrying often starts after **2–3 weeks**, depending on pain and stability

tissues further and making it much more likely that your shoulder will feel "loose" or "sloppy" in the future.

2. Reflexive Jerks
If you are carrying a bag and someone bumps into you, or you trip, your natural reflex is to grip tighter and yank the bag up. That sudden, violent contraction of your biceps and shoulder muscles can be enough to pop a vulnerable joint right back out.

3. The Bicep Connection
Your long-head bicep tendon actually attaches directly to the **labrum** (the cartilage you likely tore). When you carry a heavy book or a shopping bag, your bicep tenses up and pulls directly on that tear, preventing it from knitting back together.

The "Rules of the Road" for Your 20s

Activity	Can I use my injured arm?	The "Safe" Way
**Light Shopping**	**No**	Use a cart or carry bags with your "good" arm only.
**Single Book/Phone**	**Yes (with caution)**	Only if your elbow is **glued to your side** and the object is very light (under 1 lb).
**Opening Doors**	**No**	Always use your healthy arm. Heavy doors are a major re-injury risk.
**Carrying a Coffee**	**No**	One stumble could cause you to jerk your arm to save the drink.

How to manage "The One-Arm Life"
* **The "Two-Trip" Rule:** If you’re bringing things in from the car or moving stuff around your room, take twice as many trips using only your good arm.
* **Use Your Chest:** If you have to move a light, bulky item (like a pillow or a jacket), hug it against your chest with both arms, keeping your injured arm tucked in the sling position.
* **Ask for Help:** Seriously. At 20, it feels weird to ask someone to carry your stuff, but a "thank you" to a friend is much cheaper and less painful than a $20,000 shoulder surgery.

When can I start carrying things again?
You’ll typically start "graduated loading" in physical therapy around **week 6**. Your therapist will have you start with tiny 1 lb weights in a controlled environment before they let you go back to carrying a gallon of milk or a stack of textbooks.

subluxation**. It causes a deep, aching pain and stretches the ligaments that you are trying to let heal.

Specific Scenarios

Item	Can You Carry It?	Explanation
**A Phone or Fork**	**Yes** (with elbow tucked)	Weight is minimal. The risk is the *position* of the shoulder, not the weight.
**A Full Water Bottle**	**No**	This is the weight threshold where gravity starts to pull the shoulder joint apart.
**A Textbook / Laptop**	**Absolutely Not**	This requires the bicep to contract hard to hold the weight. The bicep tendon attaches inside the shoulder joint. Tensing it pulls on the inflamed labrum.
**A Plastic Shopping Bag**	**No**	Even if you just let it hang by your side, the 2-5 lbs of groceries will cause the arm to sway as you walk. That constant micro-motion is bad for healing.
**Carrying with **Forearm** (Waiter Carry)**	**No**	Some people try to bypass the hand by placing a book flat on the forearm like a waiter. **Avoid this.** This forces you to lift the elbow away from the body to keep the arm level.

The Only Safe Way to Carry Items Right Now
You have one "good" arm for the next few weeks. All carrying must be done with the **uninjured arm**.

- **If the bag is heavy:** Hold it in your good hand and keep that arm straight down at your side. Let the weight go straight to the ground.
- **If you need to carry two items:** Use a **backpack slung over ONE shoulder (the GOOD shoulder only)**. This frees up your good hand to open doors or hold a railing.

What About Leaning on the Injured Arm?
This is equally important: **Do not carry a heavy bag in your GOOD hand while leaning on the INJURED elbow.**

Example: Sitting at a desk, resting the injured elbow on the table while using the good hand to lift a heavy laptop bag off the floor. The weight on the good side translates to compression on the injured side. Keep the injured arm completely unloaded (resting in the sling) whenever you are lifting anything with the other arm.

	* Normal carrying typically returns as strength improves in physiotherapy (often **4–8+ weeks**)<br><br>---<br><br>### Bottom line<br><br>For now, **avoid carrying bags or books with the injured arm**, especially anything with weight or that pulls the arm downward. Light, close-to-body use is fine, but loading the arm should wait until your shoulder stabilizes more.<br><br>If you want, I can give you a simple timeline of when daily activities (lifting, sports, gym) usually come back after this injury.	**A quick tip:** If you find you absolutely have to carry something small (like your keys), keep them in a pocket on your **healthy** side. Reaching into a pocket with your injured arm involves "internal rotation," which can be surprisingly painful right now.<br><br>Does it feel like your arm is "hanging heavy" when you take it out of the sling, or does it feel relatively supported?	
36	Yes—after a first anterior shoulder dislocation, there are a few **specific movements that you should avoid in daily life**, especially in the first few weeks, because they put the shoulder in positions where it is most likely to feel unstable.<br><br>### Key movements to avoid early on<br><br>**1. Arm out to the side + rotated outward**<br><br>* Example: reaching for something far to your side or behind you<br>* This is the **most risky position** for anterior dislocation recurrence<br><br>**2. Reaching overhead**<br><br>* Putting items on high shelves<br>* Hanging or lifting objects above shoulder level<br>* This can strain healing tissues and cause pain or instability<br><br>**3. Reaching behind your body**<br><br>* Putting on or taking off tight clothing (like jackets)<br>* Reaching into a back pocket or behind your back<br>* This combines extension and rotation, which stresses the joint<br><br>**4. Sudden or forceful movements**<br><br>* Catching something quickly<br>* Being bumped in crowded spaces<br>* Quick twisting of the upper body while the arm is fixed<br><br>**5. Carrying weight with the arm hanging down**<br><br>* Shopping bags, heavy books, backpacks on one side<br>* The downward pull can irritate the healing structures	Since you are 20 and likely move with a lot of "autopilot" speed, your biggest challenge isn't the big activities—it's the small, reflexive movements you do without thinking.<br><br>There are **three specific "Danger Zones"** for a dislocated shoulder. Avoid any movement that puts your arm in these positions:<br><br>### 1. The "High-Five" Position (The Red Zone)<br>This is technically called **Abduction and External Rotation**. It is the most unstable position for the human shoulder.<br>* **Avoid:** Reaching up and back to put on a jacket.<br>* **Avoid:** Reaching for the seatbelt with your injured arm.<br>* **Avoid:** Washing your hair with both hands (use only the "good" hand for the top and back of your head).<br>* **Avoid:** Rolling over in bed with your arm behind your head.<br><br>### 2. Reaching Behind Your Back<br>This puts immense strain on the front of the joint capsule, which is exactly where most dislocations occur.<br>* **Avoid:** Tucking in your shirt or reaching for a back pocket.<br>* **Avoid:** Reaching into the backseat of a car while sitting in the front.<br>* **Avoid:** "Pulling" a heavy door shut behind you.<br><br>### 3. Sudden "Jerking" or Bracing<br>Your muscles are currently the only thing holding your shoulder together. Sudden movements can overwhelm them.<br>* **Avoid:** Bracing yourself against a wall or desk if you stumble.<br>* **Avoid:** Pushing off a chair or the bed to stand up. Use your legs and your healthy arm only.<br>* **Avoid:** Catching a falling object. If you drop your phone or a book, **let it fall.** Trying to "snatch" it out of the air is a classic way to re-dislocate.<br><br>---	Yes, absolutely. There are several everyday movements that seem harmless but are actually high-risk for a recently dislocated shoulder. Because you have a first-time anterior dislocation, the front of the joint capsule is stretched and healing. Your main goal for the next 3 to 4 weeks is to avoid any position where the arm moves **away from the body** or **rotates outward**.<br><br>Here are the specific movements at home and school you need to avoid or modify completely.<br><br>### Reaching Movements (The Most Dangerous Category)<br><br>- **Reaching for the Seatbelt:** Do not use your injured arm to grab the seatbelt and pull it across your body. This forces the arm across the chest and rotates the shoulder outward. Use your **good arm** to grab the belt and buckle it near your hip.<br>- **Reaching into the Back Seat:** Do not twist your torso and reach your injured arm behind you to grab a backpack or jacket from the back seat of a car. Even without weight, the twisting motion can cause a spasm.<br>- **Putting on a Coat or Jacket:** Do not try to put the injured arm into the sleeve first or behind your back. Drape the jacket over the injured shoulder like a cape, put your **good arm** in the sleeve, and just let the jacket hang over the injured side for warmth.<br>- **Closing a Heavy Door:** Do not push a door closed with the palm of your injured hand. Use your hip, your backside, or your good hand only.<br><br>### Lifting and Pushing<br><br>- **Lifting a Backpack onto a Desk:** You cannot lift even a light bag from the floor to a table with the injured arm. The bicep muscle attaches at the shoulder; when you lift, you pull the ball of the shoulder downward in the socket.

<table>
<tr><td></td><td>--- ### At school or home, be cautious with: * Writing for long periods if your arm is unsupported (fatigue can cause awkward positions) * Leaning on the injured arm * Opening heavy doors with that arm * Any activity that makes you feel “instability” or apprehension --- ### What is generally safe * Eating, typing, phone use (with arm supported) * Light daily hygiene (brushing teeth, washing face) * Gentle movement close to your body * Walking and normal daily activities with your arm protected (often in a sling early on) --- ### Simple rule to remember If your arm is: * **Far from your body** * **Raised above shoulder level** * **Twisted outward** ☞ it is probably not safe yet. --- ### Bottom line You don’t need to completely stop using your arm, but you should **avoid overhead, outward-rotated, and behind-the-back movements**, as well as any lifting or sudden force. If you want, I can give you a very simple “do/don’t checklist” for the first 2–3 weeks so you can use it day-to-day without thinking too much about it.</td><td>### Daily "Survival" Tweaks | Task	The Danger	The Safe Way
Dressing	Reaching back/up	**Sleeve First:** Put your injured arm into the sleeve first, then pull the rest over. When undressing, take the injured arm out **last**.
Opening Doors	Pulling/Pushing weight	Always use your **healthy arm**, or push with your back/hip if the door is heavy.
Using a Laptop	Elbows "floating"	Pull your chair all the way under the desk so your **entire forearm** is supported by the table.
Socializing	High-fives/Bumps	Keep your **sling on** in public. It acts as a "Do Not Touch" sign for friends and classmates. | ### A Note on the "Bicep Hook" Your bicep muscle has a tendon that hooks directly into the cartilage (labrum) of your shoulder. * **Avoid:** Lifting a heavy water bottle or a textbook with your palm facing up. * **Why?** This "curling" motion pulls directly on the torn labrum. If you have to lift something small, keep your palm facing your body (neutral grip). ### The "90-Degree Rule" For the next few weeks, try to keep your elbow **below the level of your shoulder** and **in front of your body.** If you can see your elbow in your peripheral vision, you are generally in a "safe" zone. If your elbow disappears behind your shoulder or goes above your ear, you are in the "danger zone." **The Expert Guide Question:** Which of these "autopilot" moves do you think will be the hardest for you to stop—reaching for your seatbelt, or maybe how you put on your backpack?</td><td>- **Pushing Yourself Up from a Chair or Bed:** **This is critical.** Do not push off the armrests of a chair with both hands to stand up. Use your **good arm** and your **leg muscles** only. Pushing up with the injured arm compresses the joint and can cause it to slide backward painfully. ### Overhead and Behind-the-Back Movements - **Washing the Opposite Armpit or Back:** In the shower, do not reach the injured arm across your body to wash under the uninjured arm. Let the water and soap run down. Do not attempt to wash your own back. - **Tucking in a Shirt:** Do not try to reach behind your back with the injured hand to tuck in a shirt or adjust your waistband. - **Brushing or Styling Hair:** Use your **uninjured arm** to brush your hair for the first week or two. If you must use the injured hand, keep the elbow pinned to your ribs and bring your **head down** to meet the brush (do not lift the brush up to your head). - **Sleeping with Arm Under Pillow or Partner:** This is a common cause of nighttime pain. The weight of a head or pillow on the upper arm forces the shoulder into a stretched position. Keep the arm in the sling or resting on a pillow **in front** of your body. ### Sudden Reflexes (The "Catch" Instinct) - **Tripping or Slipping:** When you stumble, your brain automatically tells both arms to shoot out to catch you. You **must** consciously override this. If you feel yourself falling, try to tuck the injured arm tight to your chest and take the fall on the **uninjured** side or on your buttocks. This is why walking on ice or in crowded hallways is so risky right now. **Summary Rule of Thumb:** If a movement causes you to hold your breath or makes your upper trap muscle (between neck and shoulder) tighten up, **stop immediately**. That is your body guarding the joint. If you have to think twice about whether a movement is okay, it is better to use the other arm or ask for help.</td></tr>
<tr><td>37</td><td>Yes—**it’s very normal** to feel tired, weak, or uncomfortable when using your arm after a shoulder dislocation, especially in the first days to weeks.</td><td>It is **completely normal**, and honestly, it would be more surprising if you *didn’t* feel exhausted. Even though you aren't lifting weights or running marathons, your body is doing a massive amount of "invisible" work right now.</td><td>Yes, it is absolutely normal and expected to feel both tired and uncomfortable when using your arm right now, even for very small tasks. You are 20 years old and likely used to your body recovering quickly from things, but a shoulder dislocation is</td></tr>
</table>

	### Why this happens<br><br>After the injury:<br><br>* The stabilizing tissues (labrum and ligaments) are **stretched or torn**<br>* The shoulder muscles temporarily **stop working normally to protect the joint**<br>* Your body is still “guarding” the area, which makes movement feel awkward or tiring<br>* The sling (if you’re using one) can also contribute to stiffness and fatigue<br><br>So even simple tasks can feel more effortful than usual.<br><br>---<br><br>### What you might feel (all normal early on)<br><br>* Tiredness in the shoulder or upper arm after light use<br>* Mild aching or soreness during or after activity<br>* Feeling like the arm is “heavy” or weak<br>* Cautious or stiff movements (your body protecting the joint)<br><br>---<br><br>### What is NOT normal and should be checked<br><br>* Sharp pain or sudden catching sensation<br>* Feeling like the shoulder is slipping or unstable<br>* Increasing pain day by day instead of gradual improvement<br>* Numbness or tingling in the arm or hand<br><br>---<br><br>### How to manage it<br><br>* Use your arm for **light activities only**<br>* Keep the elbow close to your body when possible<br>* Take short breaks if it gets tired<br>* Use pain relief if needed (e.g., paracetamol or ibuprofen if safe for you)<br>* Don’t push through pain or fatigue—this is your body’s warning sign<br><br>---<br><br>### Bottom line	Here is why your arm feels like it’s made of lead and why daily life feels so draining:<br><br>### 1. Muscle "Guarding" (The 24/7 Workout)<br>When a joint is unstable, your brain sends a signal to all the surrounding muscles (the rotator cuff, deltoid, and traps) to "clamp down" and hold the bone in place.<br>* **The Result:** Your muscles are essentially doing a "plank" all day long. They never get a chance to relax. This constant tension leads to that heavy, "burning" fatigue and a dull ache.<br><br>### 2. Neurological Exhaustion<br>Your brain is currently on high alert. Every time you reach for a cup or shift in your seat, your nervous system has to calculate: *"Is this move safe? Will it pop out?"* * **The Result:** This "hyper-vigilance" is mentally exhausting. By the end of a school day, you might feel like you've taken a three-hour exam just from the effort of protecting your arm in a crowd.<br><br>### 3. The "Bicep Ache"<br>Many people feel a specific ache in the front of their arm (the bicep). Because the bicep tendon attaches to the torn cartilage (labrum) in your shoulder, it is under constant tension trying to help stabilize the joint.<br>* **The Result:** Simple things like holding a phone or a fork feel much harder because the "anchor" of the muscle is compromised.<br><br>### 4. Poor Posture (The "Crip Slouch")<br>Because you are likely wearing a sling or protecting one side, you are probably leaning or hunching your shoulder toward your ear.<br>* **The Result:** This creates "referred pain" in your neck and upper back. You aren't just tired in your shoulder; your whole upper body is compensating for the injury.<br><br>---<br><br>### How to manage the discomfort:<br><br>* **The "Sling Break":** If you feel the muscles in your neck getting tight, sit in a supportive chair with your arm resting on a pillow and take the sling off for 10 minutes. Let your shoulder "drop" away from your ear.<br>* **Ice is your Reset Button:** When the arm feels heavy and "angry" after class, ice it for 20 minutes. It slows down the nerve signals and gives those "guarding" muscles a break.<br>* **Micro-Rest:** Don't try to go a full 8 hours at school without a break. Find 5 minutes between classes to sit down and let the weight of the arm be fully supported by a table or your lap.	different from a bruise or a cut. The fatigue and discomfort you are feeling are not a sign that something is wrong; they are a sign that your body is doing exactly what it needs to do to protect the healing joint.<br><br>Here is the detailed explanation of why this happens and what it means.<br><br>### 1. Muscle Guarding (The Hidden Workout)<br>Even when you think your arm is relaxed, the muscles around your shoulder blade and rotator cuff are **constantly slightly tensed**. This is called "muscle guarding" or "protective spasm." It is an involuntary reflex your brain uses to create a natural brace around the loose joint so you don't move it too far by accident.<br><br>- **The Effect:** Imagine holding your fist clenched at 10% strength all day long. By lunchtime, your forearm would be exhausted. That is what is happening inside your shoulder. This constant low-grade contraction is **exhausting**. It is the primary reason you feel wiped out by mid-afternoon even if you just sat in class.<br><br>### 2. The "Good Arm" Compensation<br>Because you cannot use the injured arm for carrying or lifting, your **uninjured arm** is now doing the work of two arms. It is opening heavy doors, carrying your backpack (on one shoulder), and brushing your teeth.<br>- **The Effect:** Your good arm, neck, and upper back muscles are being overloaded. You might notice that your **uninjured** shoulder or neck actually feels more sore and tight than the injured one. This is normal, but it contributes to overall body fatigue.<br><br>### 3. Swelling and Healing Energy<br>Inside the joint, there is still inflammation (swelling) from the stretch injury to the ligaments and labrum. Your body is sending a massive amount of blood flow and energy to that area to lay down new collagen and repair the tissue.<br>- **The Effect:** Healing a significant soft tissue injury is metabolically expensive. Your body is burning extra calories and energy to repair that joint, which leaves less energy for things like staying alert in a 3 PM lecture. **This is why you should prioritize sleep right now more than usual.**<br><br>### 4. The Type of Discomfort You Should Expect vs. Warn You About

<table>
<tr>
<td></td>
<td>Yes, **fatigue and mild discomfort with daily use are expected** after this injury. It usually improves gradually over the first few weeks as the shoulder stabilizes and you begin physiotherapy.

If you want, I can explain what recovery typically feels like week by week so you know what improvements to expect.</td>
<td>
When is it NOT normal?
While fatigue and dull aches are expected, you should call your doctor if you feel:
* **Sharp, "electric" shocks** down your arm.
* **Numbness or tingling** in your fingers that doesn't go away.
* **A feeling of "slipping"** just from moving your hand.

The Bottom Line
At 20, you're used to your body just "working" without you thinking about it. Having to manually "manage" an arm is a huge energy drain. Be patient with yourself—the first 14 days are the peak of this exhaustion, and it will start to improve as the internal swelling goes down.

Are you feeling the fatigue mostly in the shoulder itself, or is it starting to make your neck and back feel stiff too?</td>
<td>It is important to distinguish between "healing fatigue" and "re-injury pain." | **Normal "Healing" Sensations**	**Warning Signs (Stop the Activity)**
A dull, deep ache in the **back** of the shoulder or shoulder blade.	A sharp, sudden **pinch** in the **front** of the shoulder joint.
Feeling like your arm weighs 50 lbs by the end of the day.	Feeling the ball of the shoulder "slide" or "clunk" out of place for a split second.
Soreness in the bicep muscle from holding the elbow bent.	Numbness or tingling shooting down to the fingers (this is a nerve issue).
General whole-body tiredness/fatigue.	Increased swelling or visible bruising that wasn't there before. | **The Bottom Line:** Do not try to "push through" this fatigue. If you feel the deep ache or your arm feels heavy, **put the arm back in the sling** (even if you are just sitting at a desk) and rest it for 20-30 minutes. The sling acts like a kickstand for a bicycle, taking the weight off the muscles so they can stop guarding for a bit. This will help you get through the rest of the day with less discomfort.</td>
</tr>
<tr>
<td>38</td>
<td>Yes—**sitting at a desk, writing, and using a computer are generally safe after a shoulder dislocation**, and most people are encouraged to continue these activities as soon as they feel comfortable.

Writing and desk work

* You can write normally **if it doesn’t cause pain**
* Keep your **elbow close to your body**
* Don’t lean forward or reach far across the desk
* If your arm gets tired, take short breaks

If writing is uncomfortable at first, it’s often due to muscle guarding and fatigue, not damage.

Using a computer or phone

* Completely fine in most cases
* Try to:

  * Rest your forearm on a desk, armrest, or pillow
  * Keep the shoulder relaxed (not lifted or held in the air)
  * Avoid long periods without breaks at first</td>
<td>You can sit at a desk and use a computer, but the word **"normally"** is the catch. If you sit the way most 20-year-olds do—slumped forward with your elbows hovering in the air—you will be in significant pain within 15 minutes.

To work safely, you have to follow the **"Supported Forearm"** rule.

1. The Setup: The "Desk-as-a-Sling" Technique
Your goal is to let the desk take the weight of your arm so your shoulder muscles don't have to.
* **Pull your chair all the way in:** Your stomach should almost be touching the edge of the desk.
* **Rest your entire forearm on the desk:** Do not just rest your wrist on the edge. Your elbow and forearm should be fully supported by the table surface.
* **Keyboard placement:** Slide your keyboard and mouse further away from you so your arms can stretch out slightly while remaining flat on the desk.

2. Typing vs. Writing
* **Typing:** This is generally safe because your hands stay in a "neutral" position. As long as your elbows stay tucked by your ribs and supported by the desk, you can type for a decent amount of time.</td>
<td>Yes, you can sit at a desk, write, and use a computer normally fairly soon after a first-time shoulder dislocation, but it's important to do so with some modifications to protect the healing joint.

Since there were no fractures or nerve injuries, the main restriction is the sling and avoiding specific shoulder movements.

General Guidelines for Desk Work
- **Return Timeline:** You can generally return to desk-based work or study as soon as you feel comfortable managing it. One source notes that for sedentary jobs, returning "as tolerated" is a reasonable guide . However, for the first few weeks, you will likely be wearing your sling .
- **Writing and Using a Mouse:** You can use your hand for light activities while your arm is in the sling. This includes writing, typing, and using a computer mouse . It's a good idea to keep these sessions short at first to see how your shoulder feels.

Practical Tips for Setting Up Your Workspace
To avoid discomfort and prevent straining your shoulder while it heals, try to set up your workstation thoughtfully:</td>
</tr>
</table>

<table>
<tr>
<td></td>
<td>---

Things to avoid while working

* Reaching for objects far away repeatedly
* Sitting in a position where your shoulder is unsupported and hanging
* Sudden twisting or stretching while focused on work
* Carrying or lifting heavy items at the same time

If you’re wearing a sling

* You can still do desk work with the injured arm supported in the sling
* For typing or writing, some people briefly remove the sling if it feels comfortable, then put it back on afterward

---

Bottom line

Yes, **you can safely go to school or work at a desk and use a computer normally**, as long as you keep movements small, avoid strain, and support the arm.

If you want, I can also tell you how long it usually takes before you can return to full sports or gym activity, which is the part that takes longer.</td>
<td>* **Writing (Handwriting):** This is much harder. Handwriting requires small, repetitive "tugging" motions that can irritate the bicep tendon and the front of the shoulder. If it's your **dominant** arm, try to keep your writing sessions short (10–15 minutes) and take "ice breaks." ### 3. The Mouse: The "Danger Zone" The biggest mistake people make is reaching out to the side for a mouse. * **The Risk:** Reaching out wide to use a mouse puts your shoulder in "external rotation," which is an unstable position. * **The Fix:** Move your mouse closer to the center of your body, right next to the side of your keyboard. Use your wrist to move it, not your whole arm. ### 4. Posture: Don't "Hike" Your Shoulder When we're in pain, we instinctively pull our injured shoulder up toward our ear. * **The Result:** This causes massive tension headaches and neck pain. * **The Fix:** Every 20 minutes, do a "posture check." Consciously drop your shoulders down and tuck your shoulder blades back and down (like you're trying to put them in your back pockets). --- ### **Desk Work Summary Table** | Activity	Recommendation
Laptop Use	Use a separate mouse/keyboard if possible so the screen can be at eye level.
Writing	Lean forward so you aren't "reaching" for the paper.
Phone Use	Prop your phone up on a stand; don't hold it up with your injured hand.
Duration	45 mins on / 15 mins off. You need to move around to prevent stiffness. | ### **The "Red Flag" at the Desk** If you start to feel a **burning sensation** between your shoulder blades or a **numbing/tingling** feeling in your pinky and ring finger, your posture is likely pinching a nerve or overworking a muscle. That is your signal to stop, put the sling back on, and go lie down or use an ice pack. **A quick tip for school:** If your desks at school are those tiny "swing-arm" tablets attached to the chair, **avoid them.** They are terrible for shoulder injuries. Try to find a full-sized table or a desk in the back of the room where you can fully support your arm.</td>
<td>- **Keep Everything Close:** Arrange your desk so your mouse, keyboard, and other frequently used items are close to your body. This prevents you from having to reach out or forward, which can strain your shoulder .
- **Use Arm Support:** If your chair has armrests, adjust them so they can support your elbows with your arms bent at roughly a 90-degree angle. This helps keep your shoulders relaxed . A chair with good lumbar support will also help you maintain a posture that is less tiring for your upper body .
- **Take Frequent Breaks:** Avoid staying in one position for too long. Aim to take a short, 5-minute break at least once every hour to stand up, walk around, and gently move . This helps prevent general muscle stiffness and fatigue.
- **Practice Good Posture:** Try to sit upright with your shoulders back in a relaxed position rather than slumping forward .

Important Cautions
- **Follow Medical Advice:** This guidance is for general desk activities. You must follow the specific instructions from your doctor or physiotherapist regarding your sling use, exercises, and when you can return to other activities like driving or sports .
- **Listen to Your Body:** Some aching is normal as you start using the arm more, but if you feel sharp pain, stop the activity and rest. If you have concerns about your progress, contact your physiotherapist or healthcare team .

You can gradually return to your normal desk routine by making these small adjustments to your workspace and taking regular breaks. The key is to listen to your body and avoid movements that cause sharp pain.</td>
</tr>
</table>

		Are you using a laptop or a desktop computer mostly?	
39	You don't need to "baby" the arm completely, but you also **shouldn't use it normally yet**. After a first anterior shoulder dislocation, the right approach is **controlled, careful use**.<br><br>### The correct balance: "use it, but protect it"<br><br>You can:<br><br>* Use your arm for **light daily activities**<br>* Keep the shoulder gently moving in safe positions<br>* Do normal things like eating, writing, using your phone<br><br>But you should avoid:<br><br>* Overhead movements<br>* Reaching far away from your body<br>* Carrying weight (bags, books, groceries)<br>* Sudden or forceful movements<br>* Positions where the arm is out to the side and rotated outward<br><br>---<br><br>### Why this matters<br><br>Right now:<br><br>* The stabilizing tissues are still healing<br>* The shoulder is temporarily more "loose" in certain positions<br>* Early overuse doesn't strengthen it—it can **increase risk of another dislocation**<br><br>---<br><br>### A simple rule to guide you<br><br>If your arm is:<br><br>* **Close to your body → OK**<br>* **Light use → OK**<br>* **Far from your body or under load → be careful / avoid**<br><br>---<br><br>### What "normal use" really means later<br><br>You'll return to full normal use gradually:	At **20 years old**, your body feels like it should bounce back instantly, but you need to be **very careful.** You cannot use your arm "normally" yet.<br><br>Think of your shoulder right now as a door that has been ripped off its hinges. The door might be back in the frame, but the screws are loose and the wood is splintered. If you swing it open "normally," it's going to fall off again.<br><br>For the next **4 to 6 weeks**, you are in a "protection phase." Here is the reality of what "careful" actually looks like:<br><br>### 1. The "T-Rex" Rule<br>For daily activities (eating, typing, brushing teeth), keep your elbow **glued to your side.** * **Safe:** Moving your wrist and hand as long as your elbow stays tucked in.<br>* **Dangerous:** Reaching out to the side, reaching behind you, or reaching above your head.<br><br>### 2. No "Auto-Pilot"<br>Most re-injuries at your age happen during "reflex" movements. You have to consciously think about your arm before you move.<br>* **Do not** reach for a falling phone.<br>* **Do not** reach into the backseat of a car.<br>* **Do not** push off the bed with your injured arm to stand up.<br><br>### 3. The Weight Limit<br>Your lifting limit for the next few weeks is **zero.**<br>* Carrying a gallon of milk, a heavy textbook, or a full laundry basket can create "traction" that pulls the healing labrum apart. Even if it doesn't pop out, it prevents the tissues from tightening back up.<br><br>### 4. Why You Can't Be "Normal" Yet<br>In your 20s, your ligaments are naturally more elastic. This is usually a good thing, but after a dislocation, it means the joint is "sloppy." If you use it normally before your rotator cuff muscles are strong enough to hold it in place, you are significantly increasing your risk of a **permanent, life-long instability.**<br><br>---<br><br>### **The "Safe vs. Danger" Guide**<br><br>| Activity | Can I do it normally? | The "Careful" Way | | :--- | :--- | :--- | | **Dressing** | **No** | Put the injured arm in the sleeve **first**. |	This is a crucial distinction to make for proper healing. **You need to be very careful with your arm right now, and you cannot use it "normally" in the way you did before the injury.**<br><br>While the last answer clarified that you *can* do light desk work, this question is about *how* you use the arm in daily life. The difference is important: you can use the hand for fine tasks, but you must protect the shoulder joint itself.<br><br>Here is the clear, patient-focused guidance for daily activities during the first 3 to 6 weeks after a first-time anterior dislocation.<br><br>**1. The "Safe Zone" Rule**<br>You should keep your elbow tucked near your side or in front of your belly button. As long as your elbow stays glued to your side, you can generally use your hands for:<br>- Writing or typing.<br>- Eating (bring the food to your mouth by bending your neck forward slightly, not by lifting the arm high).<br>- Brushing your teeth.<br>- Washing your face (using one hand).<br><br>**2. Movements You Must Strictly Avoid (This is Non-Negotiable)**<br>You must be **very careful** to avoid two specific positions for the next several weeks. These movements stretch the exact area that was torn during the dislocation.<br>- **Reaching Outward:** Do not reach for the coffee cup in the drive-thru, grab a seatbelt with that arm, or push open a heavy door.<br>- **Reaching Backward:** Do not reach into the back seat of the car or stretch to put on a coat sleeve behind your back.<br>- **External Rotation:** This is the most dangerous movement. It is the motion of holding your elbow at your side but turning your hand outward, like you are opening a door knob or reaching into your back pocket. **Do not do this.**<br><br>**3. How to Use the Sling as a "Reminder"**<br>You may have been told you can take the sling off to shower or write. However, when you are walking around the house, in a crowded hallway, or sleeping, **wear the sling.**<br>- **Why?** Even if you feel okay, a sudden trip, a slip on a wet floor, or instinctively grabbing a falling object with that arm can cause the shoulder to pop out again. The sling protects you from your own reflexes.

	* Early phase (now): light, protected use<br>* A few weeks later: more movement + strengthening<br>* After rehab: full use, including sports (if stable)<br><br>---<br><br>### Bottom line<br><br>You should **not treat it completely normally yet**, but you also don’t need to avoid using it entirely. Think of it as **“careful normal use” rather than full normal use**.<br><br>If you want, I can give you a very simple list of what is safe vs unsafe in the first 2–3 weeks so you don’t have to second-guess every movement.	| **Showering** | **No** | Use the "Lean" method to wash under your arm. | | **Socializing** | **No** | Wear your sling as a "shield" to prevent bumps. | | **Using a Phone** | **No** | Elbow on your stomach; bring the phone to your face. | <br>### **The Bottom Line**<br>You should be **very careful** for about **6 weeks**. After that, once you’ve started Physical Therapy and your therapist confirms your "cuff" is strong, you can gradually start returning to normal movements.<br><br>If you try to rush back to "normal" in week 2, you might end up in the ER in week 3. It’s better to be bored and careful for a month than to need surgery and be out of commission for six months.<br><br>**The Expert Guide Question:**<br>What is the one thing you’re most tempted to do "normally" right now—is it sports, driving, or just getting through your school day?	**4. Specific Daily Activity Warnings**<br>- **Putting on Shirts:** **ALWAYS** put the injured arm in the sleeve **first**. When taking the shirt off, take the injured arm out **last**. Lean forward and let gravity help you; do not lift or twist the arm to get dressed.<br>- **Sleeping:** Sleep on your back or on the **opposite** side. If you sleep on the injured side, you will wake up in extreme pain. Propping a pillow behind the arm while on your back can help prevent it from rolling outward at night.<br>- **Lifting Objects:** Do not lift anything heavier than a coffee mug or a smartphone with that hand. Do not use that arm to push yourself up out of a chair or bed.<br><br>**Summary for the Next Month**<br>**Can I use it normally?** No. The shoulder joint is loose and healing. Treat the arm like it has a broken bone, even though it doesn't—because the **soft tissue** (ligaments) is torn and needs just as much rest as a fracture would.<br><br>**Can I use my hand and fingers?** Yes, as long as your elbow stays by your side.<br><br>If you are unsure whether a movement is safe, the safest answer is **don't do it** until you see a physical therapist for specific clearance. Being "very careful" now significantly lowers the 80% to 90% chance of this happening again at your age.
40	You can start exercising your shoulder fairly early—but it depends on **what type of exercise** and **how far you are from the dislocation**.<br><br>After a first anterior shoulder dislocation, rehab is usually done in phases:<br><br>---<br><br>## 1. Early phase (first few days to ~1–2 weeks)<br><br>**Goal: protect the shoulder while preventing stiffness**<br><br>You can usually start **very gentle, pain-free movements**, such as:<br><br>* Pendulum exercises (arm hanging and gently swinging)<br>* Very small assisted range-of-motion movements (only if advised)<br>* Hand, wrist, and elbow movements (important to keep them active)<br><br>Avoid:<br><br>* Any lifting or resistance<br>* Raising the arm above shoulder level	Since you are 20, your body is probably itching to move, but you have to resist the urge to "test" your strength too early. Exercising a shoulder that was recently dislocated is a staged process. If you jump straight into the gym, you risk turning a temporary injury into a permanent problem.<br><br>Here is the general timeline for when and how you can start moving again:<br><br>### Phase 1: The "Activation" Phase (Days 3–14)<br>You can usually start **Isometric** exercises within a few days, provided your pain is under control. These are exercises where you contract the muscle **without moving the joint.**<br>* **What you can do:** Gently squeeze your shoulder blades together or "pulse" your hand against your stomach.<br>* **The Goal:** Keep the muscles from "falling asleep" while the ligaments are still in the early stages of knitting back together.<br>* **Wrist and Elbow:** You should exercise your wrist and elbow (circles and curls) immediately to prevent stiffness, as long as the shoulder stays still.<br><br>### Phase 2: The "Safe Range" Phase (Weeks 2–6)	You can and should start **gentle, specific exercises almost immediately**, but this is very different from returning to "working out" or weightlifting. The timeline is generally broken down into distinct phases. For a young patient with a first-time dislocation, early movement is essential to prevent long-term stiffness (frozen shoulder), even though the joint needs protection to heal .<br><br>Here is the general timeline for what you can expect:<br><br>**Phase 1: Immediate (First 1-2 Weeks)**<br>You should begin **gentle range of motion** as soon as pain allows, often within the first few days. The goal here is not to build muscle, but to keep the elbow, wrist, and fingers from getting stiff while you protect the shoulder .<br>- **Pendulum Swings:** Lean over and let the injured arm dangle like a dead weight. Use your hips to gently swing the arm in small circles. **Do not use your shoulder muscles to lift the arm** .<br>- **Assisted Movement:** Use your good arm to help lift the injured arm forward (flexion) or out to the side, *only* as far as is completely pain-free .

<table>
<tr>
<td></td>
<td>* Outward rotation (arm turned out and away from body)

---

2. Early strengthening phase (~2–6 weeks)

(Only after pain improves and your doctor/physio allows it)

You may begin:

* Light isometric exercises (muscle tightening without movement)
* Band exercises with very light resistance
* Controlled range-of-motion work

Still avoid:

* Heavy lifting
* Overhead exercises
* Contact or high-risk activities

---

3. Strengthening + control phase (~6–12 weeks)

* More structured physiotherapy
* Resistance band training
* Shoulder stability and rotator cuff strengthening
* Gradual return to normal movement patterns

---

4. Return to sport / full activity (often 3–6 months)

* Depends on stability and sport type
* Contact or overhead sports usually take longer
* Only after strength and stability are fully restored

Important point for your case

Because you are **20 years old**, your shoulder is:

* More likely to become unstable again if pushed too early
* Very responsive to proper rehab if done correctly

So **starting too aggressively is the main risk—not starting too early with gentle rehab**</td>
<td>Once you see a Physical Therapist (PT), they will start you on **Passive or Active-Assisted Range of Motion.** * **What you can do:** "Pendulums" (leaning over and letting your arm dangle in small, tiny circles) and using a stick or a pulley to gently move the arm without using the shoulder muscles themselves. * **The Rule:** You generally stay below shoulder height. No overhead movements and **zero** weight. ### Phase 3: The "Strengthening" Phase (Weeks 6–12) This is where the real work begins. Once the "glue" of the labrum has had about six weeks to set, you start building the "armor." * **What you can do:** Internal and external rotation using light resistance bands. * **The Focus:** You aren't training for "beach muscles" (biceps/chest); you are training the **Rotator Cuff**. These small muscles act as the "suction" that keeps the ball in the socket. --- ### **When can I go back to the gym?** | Activity	Estimated Timeline
Lower Body (Leg Press/Extensions)	**Week 2** (If you can do it without holding onto handles)
Cardio (Stationary Bike)	**Week 2** (Don't lean on your arms)
Light Resistance Bands	**Week 6**
Bench Press / Overhead Press	**3–6 Months** (Only with PT clearance)
Contact Sports / Pull-ups	**6+ Months** | --- ### **The "No-Go" Signs** Stop immediately if you feel: * **A "shifting" sensation:** Like the bone is moving over a speed bump. * **Sharp pain:** Exercise should feel like a "burn," not a "stab." * **Apprehension:** That sudden "Oh no!" feeling that it's about to pop out. ### **The Golden Rule** **Do not start a "shoulder workout" you found on YouTube.** Because your shoulder dislocated, certain standard exercises (like "Lat Pull-downs" behind the head or "Chest Flies") are actually dangerous for you right now.</td>
<td>**Phase 2: Early Strengthening (Starting around 2-4 Weeks)**
Once you are out of the sling and have a little more comfort, you can start **isometric exercises**. These are muscle contractions **without moving the joint**. They activate the rotator cuff safely .
- **Wall Push:** Stand facing a wall with your elbow bent 90 degrees, make a gentle fist, and push forward into the wall as if you are trying to punch it lightly. Hold for 5-10 seconds, relax, and repeat. You should not see your shoulder actually move .
- **Outward Press:** Stand sideways to a wall with the injured arm closest to the wall. Bend the elbow 90 degrees and press the back of your wrist outward against the wall (like you are trying to rotate the arm away from your body, but the wall stops you) .

Phase 3: Resistance Bands (Starting around 4-6 Weeks)
If you are progressing well, you will advance to **resistance bands** for internal and external rotation. This is where you begin moving the joint through its range against light tension .

Phase 4: Weight Training (Starting around 6-12+ Weeks)
This is when you can *gradually* introduce very light weights or machines. However, you must strictly avoid **abduction (lifting arm sideways away from body) combined with external rotation (the "throwing" position)** for at least **6 weeks** as this is the most vulnerable position for re-dislocation .

Crucial Caution Regarding Pain
It is normal for the shoulder to **ache** during and after these exercises. However, if the ache does not settle within **30 minutes** of stopping the exercise, you are doing too much. You should reduce the intensity or repetitions .

Important Disclaimer
The above timeline is a general guide for a standard first-time dislocation without fracture. Because recurrence rates are highest in patients under 30, you should not self-manage the entire recovery. You need an assessment by a physiotherapist to ensure you aren't developing excessive looseness. They will clear you for the next phase based on your specific strength, not just the calendar .</td>
</tr>
</table>

	---<br><br>## Bottom line<br><br>* You can start **very light, controlled exercises within days**<br>* Real strengthening begins **after a few weeks**<br>* Full sports-level training usually takes **months**<br><br>---<br><br>If you want, I can give you a **simple safe home exercise plan for the first 2 weeks** so you know exactly what to do daily without risking your shoulder.	**Your Next Step:** Get a referral for **Physical Therapy**. They will give you a specific "home exercise program" (HEP) tailored to exactly how your shoulder dislocated.<br><br>Are you already a regular at the gym, or were you planning on starting a new routine once you felt better?	
41	You *can* do some shoulder exercises at home, but with an important condition: **you should only do very basic early exercises on your own, and ideally have a physiotherapist guide your progression.**<br><br>After a first anterior shoulder dislocation, the issue isn't whether exercise is allowed—it's **which exercises, how soon, and how correctly they're done**.<br><br>---<br><br>## What you can safely do at home (early stage)<br><br>These are usually safe in the first days to 1–2 weeks **if pain allows**:<br><br>* **Pendulum exercises** (arm relaxed, small gentle swings)<br>* **Elbow, wrist, and hand movements** (to prevent stiffness)<br>* Very gentle assisted movements *only if they don't cause pain or instability*<br><br>Key rule: **no pain, no forcing, no resistance**<br><br>---<br><br>## What you should NOT do on your own early<br><br>Avoid anything that involves:<br><br>* Resistance bands or weights<br>* Lifting the arm against gravity (especially above shoulder level)<br>* Outward rotation (arm turning away from the body)<br>* "Strength training" type exercises<br><br>These are the types of movements that usually need **physiotherapist supervision first**, especially in young patients like you.	At **20 years old**, your recovery isn't just about getting the pain to stop; it's about preventing your shoulder from becoming "loose" for the rest of your life. Because of that, **you absolutely need a professional (Physical Therapist) to show you how to start.**<br><br>While you will eventually do most of your exercises at home, doing them solo right now is risky for two main reasons:<br><br>### 1. The "False Sense of Security"<br>A week from now, you might feel 80% better. You'll be tempted to try a "shoulder stability" move you saw online. However, if your **form** is off by even an inch (e.g., your elbow is too far back), you could accidentally lever the ball of the joint against the torn part of your labrum and pop it right back out.<br><br>### 2. Muscle "Cheating"<br>Your body is smart. To avoid pain, it will try to "cheat" by using your neck or back muscles to move your arm. A PT's job is to watch you and say, *"Stop—you're using your trap muscle, not your rotator cuff."* If you train the wrong muscles at home, you'll end up with a stiff neck and a shoulder that is still unstable.<br><br>---<br><br>### What you CAN do at home right now (Safe & Simple)<br>Until you see a therapist, you can do these "non-moving" exercises to keep your blood flowing:<br><br>* **Hand and Wrist Drills:** Squeeze a stress ball or make a fist and rotate your wrist. This keeps the forearm muscles from wasting away while you're in the sling.<br>* **Scapular Squeezes:** While sitting up straight, gently pinch your shoulder blades together like you're trying to hold a pencil between them. Do **not** move your arm; just move the shoulder blade.	For the first phase of recovery, specifically the first 2 to 4 weeks, **it is strongly recommended that you have a professional show you how to do the exercises at least once.**<br><br>While many shoulder exercises look simple on paper or in a video, they rely on a specific feeling of using the *correct* muscles. Doing them wrong, especially after a dislocation, can actually increase your risk of the shoulder slipping out again.<br><br>Here is the honest breakdown of what you can safely do alone versus what requires a trained eye.<br><br>**What You Can Likely Start Safely at Home Immediately**<br>These are "passive" exercises that require almost no muscle activation in the injured shoulder. They are very safe to do on your own as long as you follow the rule of no sharp pain:<br>- **Pendulum Swings:** Leaning over and letting gravity do the work. It's very hard to hurt yourself doing this.<br>- **Hand and Wrist Squeezes:** Making a fist or squeezing a soft ball to keep blood flowing.<br><br>**Why You Need Someone to Show You the Rest**<br>Once you move beyond pendulums (usually around Week 2-4), the exercises become "isometric" or involve light resistance bands. This is where guidance becomes critical for three specific reasons:<br><br>1. **Avoiding "Shoulder Shrugging":** When you try to lift your arm, your brain will instinctively want to hike your shoulder blade up toward your ear to compensate for the weak rotator cuff. You won't feel this happening, but a physical therapist will see it immediately. If you do all your rehab with a shrugged shoulder, you strengthen the wrong muscle (the upper trap) and leave the dislocated area weak and unstable.<br>2. **Protecting the Front of the Shoulder:** For an anterior dislocation, you must avoid pushing the ball of the shoulder

	---<br><br>## Do you need someone to show you?<br><br>**Yes, ideally a physiotherapist should guide you**, because:<br><br>* Correct technique matters a lot for shoulder stability<br>* The wrong movement pattern can increase risk of repeat dislocation<br>* Progression (when to add resistance or range) should be individualized<br><br>Even 1–2 sessions can be very helpful to:<br><br>* Teach safe exercises<br>* Show correct form<br>* Give you a structured plan to follow at home<br><br>---<br><br>## Why guidance is especially important in your case<br><br>At age 20:<br><br>* The shoulder is more likely to become unstable again if rehab is too aggressive<br>* But also very capable of full recovery if rehab is done properly<br><br>So the goal is **precision, not intensity**<br><br>---<br><br>## Bottom line<br><br>* **Yes, you can start simple exercises at home safely**<br>* But **you should ideally have a physiotherapist guide your program**, especially before progressing to strengthening work<br><br>---<br><br>If you want, I can outline a **safe day-by-day home routine for the first 1–2 weeks**, so you know exactly what you can do without needing equipment or risk.	* **Elbow Extensions:** Very carefully take your arm out of the sling while sitting down. Let it hang straight down to your side for a moment to stretch the elbow, then put it back. (Do this only if it doesn't cause shoulder pain).<br><br>### What a PT will teach you first<br>When you go to your first appointment, they will show you the **"Big Three"** foundational moves:<br>1. **Pendulums:** How to use gravity to "dangle" the arm without using your muscles.<br>2. **Isometrics:** How to push against a wall or your other hand to wake up the rotator cuff without moving the joint.<br>3. **Proper Sling Use:** How to get in and out of your clothes and sling without "opening" the joint.<br><br>---<br><br>### **The "Home Exercise" Plan**<br>Once a PT shows you the correct way to do these moves, they will give you a "Home Exercise Program" (HEP). **This is where the real healing happens.**<br>* **PT's Role:** To teach, adjust, and "test" your stability.<br>* **Your Role:** To do those 10–15 minutes of boring, small movements every single day at home.<br><br>### **The Warning Signs**<br>If you try to "self-teach" and you feel **sharp pain, a "clicking" sound, or a "slipping" sensation**, stop immediately. That is a sign that the exercise is too advanced or your form is incorrect.<br><br>**The Expert Guide Question:**<br>Have you already been referred to a Physical Therapist by the ER or your doctor, or are you still looking for one?	forward in the socket. A therapist can place their hands on your shoulder to ensure the movement is coming from the joint *rotation* and not a forward *thrust*.<br>3. **Setting the Right Tension:** When using a resistance band for rotation, the angle of your elbow matters down to the inch. If your elbow drifts away from your side, you lose the benefit of the exercise and can strain the healing ligaments.<br><br>**The "Compromise" Approach for a 20-Year-Old**<br>Given that you are in a high-risk age group for re-dislocation, the ideal path is:<br>- **Visit a Physiotherapist (PT) for 2-3 sessions.** You do **not** need to go three times a week for months. Many PTs are happy to do a "consult and home program." They will teach you the 4-5 specific moves you need for this stage, watch you perform them, correct your form, and then send you home with a clear sheet to follow for the next 4 weeks.<br>- **If that is impossible:** Use a **mirror**. If you are following a video online, place a full-length mirror to your side. Look at your neck and shoulders. If your injured shoulder is creeping up toward your ear, stop, relax, and try again.<br><br>**Summary**<br>You can do the *resting* phase exercises at home. For the *rebuilding* phase, having someone watch you once ensures you aren't training the shoulder to be unstable. At 20 years old, this small investment of time now makes a significant difference in whether you are back on the field in 3 months or back in the emergency room in 6 months.
42	Yes—**starting the wrong exercises too early, or doing too much too soon, can increase the risk of another dislocation.** But it's important to understand the nuance: it's not "exercise in general" that's risky, it's **specific movements and loads in the early healing phase**.<br><br>---	**Yes, absolutely.** At 20, "over-eagerness" is actually one of the leading causes of re-dislocation.<br><br>When you dislocate your shoulder, the **labrum** (the cartilage ring) and the **ligaments** (the "straps" holding the bone in place) are often physically detached or severely stretched. These tissues	That is an excellent and very important question. The answer is **yes, starting the *wrong* exercises too early can absolutely cause the shoulder to come out again.** However, not moving at all also causes problems.

	## Why early overloading can be risky<br><br>After a first anterior shoulder dislocation:<br><br>* The **labrum and ligaments are torn or stretched**<br>* The joint is temporarily **less stable in certain positions**<br>* Healing tissues are still “soft” and not fully secured<br><br>If you stress the shoulder too early with the wrong type of movement, you can:<br><br>* Stretch healing tissues further<br>* Trigger instability episodes<br>* In some cases, cause a repeat dislocation<br><br>---<br><br>## Movements that are most risky early on<br><br>These are the positions most associated with redislocation:<br><br>* Arm **out to the side + rotated outward**<br>* Reaching overhead with force<br>* Sudden pushing or pulling<br>* Lifting weights or resistance bands too early<br>* Catching yourself with the injured arm during a slip/fall<br><br>---<br><br>## What is actually safe (and important)<br><br>Early, **gentle, controlled movement is not only safe—it helps recovery**, such as:<br><br>* Pendulum exercises<br>* Elbow/wrist/hand movement<br>* Very light assisted range of motion (if pain-free)<br><br>These do **not increase dislocation risk when done correctly**.<br><br>---<br><br>## The key difference<br><br>* **Safe rehab → controlled, low-force, close to body**<br>* **Risky activity → forceful, stretched, or loaded positions**<br><br>---	have very poor blood supply, which means they heal slowly—think of it like waiting for wet glue to dry.<br><br>Here is why jumping into exercises too early is a major risk:<br><br>### 1. The "Lever" Effect<br>Your arm is essentially a long, heavy lever. If you try to do a lateral raise or reach out to the side before your muscles are ready, you are applying massive force to those "wet glue" tissues. It takes very little force in the wrong direction to peel that labrum away again or cause the ball to slide over the "speed bump" of the socket.<br><br>### 2. Muscle Fatigue = Zero Protection<br>Right now, your muscles are "guarding" the joint. If you exercise them too early and they get tired, they "let go." Once those muscles fatigue, there is **nothing** left to hold the joint in place except your torn ligaments. This is usually when the shoulder slips out again—not during the exercise itself, but right after when the muscles are exhausted.<br><br>### 3. The "False Sense of Security"<br>Because you’re young, your pain might disappear in 10 days. You’ll feel like you’re ready to hit the gym. But **pain levels do not equal healing levels.** The tissues won't have actual structural integrity for at least **6 weeks**.<br><br>---<br><br>### The "Danger" Moves to Avoid<br>If you start these specific movements too soon, you are statistically at a much higher risk for a second dislocation:<br><br>* **External Rotation:** Turning your hand outward (like reaching for a seatbelt).<br>* **Abduction:** Lifting your arm out to the side (like a jumping jack).<br>* **Combined Movement:** Lifting your arm up AND out (the "High-Five" position). **This is the #1 way shoulders re-dislocate.**<br><br>---<br><br>### How to know if you're doing "Too Much"<br>If you experience any of these during or after an exercise, you are risking a re-dislocation:<br>* **Apprehension:** A sudden, overwhelming feeling that the arm is "vulnerable" or about to slip.<br>* **Shifting/Clunking:** Feeling the bone "thud" inside the joint.	It helps to think of this as two completely different categories of movement: **Safe Early Movement** versus **Dangerous Early Exercise**.<br><br>Here is the clear distinction for a 20-year-old with a first-time anterior dislocation.<br><br>### The Danger: Premature Strengthening or Stretching<br>If you skip the gentle "range of motion" phase and jump straight into what most people think of as "exercise" (lifting weights, push-ups, resistance bands with full motion), you are at high risk for re-dislocation. Here is why:<br><br>- **The Ligaments Are Loose:** When the shoulder popped out, it stretched or tore the labrum and ligaments in the *front* of the socket. These tissues take **6 to 8 weeks** to even begin scarring down firmly. Before that, the shoulder is like a golf ball sitting on a tee that has a chip missing from the front edge—it takes very little force to knock it off again.<br>- **The Vulnerable Position:** The position that caused the dislocation (arm out to the side and rotated back, like throwing a ball) is the exact position you must avoid. If you do a push-up or a bench press **too early**, you are putting stress on that exact front pocket of the shoulder.<br>- **Muscle Spasm Protection Fades:** In the first week, your muscles are in spasm, which ironically acts like a natural cast holding the joint in. As that spasm wears off around Week 2 or 3, the shoulder actually feels *more* loose and vulnerable. If you decide at that moment, "Hey, it feels better, I'm going to do some shoulder presses," you are at peak risk for it slipping out again.<br><br>### What Happens if You Do *Nothing*?<br>On the flip side, if you keep the arm completely frozen in the sling for 4-6 weeks without doing the **gentle, safe exercises** (Pendulums, elbow bending), you won't dislocate it, but you will develop **Frozen Shoulder (Adhesive Capsulitis)** . This is where the joint capsule shrinks and becomes so stiff you cannot lift your arm to put on a shirt. This is painful and takes months of painful stretching to fix.<br><br>### How to Know if You're "Too Early"<br>You are likely pushing too hard, too soon if:<br>1. You feel a **sharp, pinching pain** in the **front** of the shoulder during the movement (the same spot where it popped out).<br>2. You feel a sensation that you need to **hike your shoulder up to your ear** to complete the movement.

	## Why guidance matters at your age<br><br>At 20:<br><br>* Your shoulder is more likely to recur if overstressed early<br>* But also very responsive to structured rehab<br>* So timing and technique matter more than “doing more exercise”<br><br>---<br><br>## Bottom line<br><br>Yes, **doing the wrong exercises too early can increase the chance of another dislocation**, but **proper, gentle rehabilitation exercises are safe and important for healing**.<br><br>---<br><br>If you want, I can give you a very clear “safe movement map” (what positions are green/yellow/red) so you can confidently know what is safe in daily life and exercise.	* **Increased Night Pain:** If your shoulder throbs more at night after "exercising," you have caused micro-tearing in the healing tissue.<br><br>### The Success Path<br>The goal of Physical Therapy is to keep you in a **"Safe Zone"** where you strengthen the muscles without stretching the ligaments.<br>* **Weeks 1-2:** Stay in the sling; do only "non-moving" (isometric) squeezes.<br>* **Weeks 2-6:** Movement only within a very small, "protected" range.<br>* **After Week 6:** Gradual strengthening.<br><br>> **The Reality Check:** Every time your shoulder pops out, the damage gets worse and the chance of needing major surgery goes up by about **50%**. Taking it slow for one month is a small price to pay to avoid a lifetime of "loose" shoulders.<br><br>Do you feel any "clicking" or "slipping" when you move your arm even slightly right now?	3. You feel a sensation of **"apprehension"** or anxiety in your brain before you even start the movement. Your body knows the joint is loose and is sending a warning signal. **Listen to that signal.**<br><br>### Summary for a 20-Year-Old<br>At your age, the tissues are elastic and heal quickly, but the **re-dislocation rate is also at its highest** because young people tend to feel "fine" and return to normal activity before the ligament has tightened up.<br><br>**The Safe Path:**<br>- **Start Early:** Gentle pendulums and elbow/wrist motion (within 3-5 days).<br>- **Delay Resistance:** Wait for **clearance from a doctor or PT** before using bands or weights to *rotate* the arm away from the body.<br>- **Red Flag:** If you ever feel the joint "slip" or "shift" slightly even without full dislocation, stop immediately and see your doctor. That is called a subluxation and means the shoulder is not ready for that load.
43	After a first anterior shoulder dislocation, the **first exercises are very gentle and focused on movement and muscle activation without stressing the joint**. The goal early on is *not strength*, but **protecting stability while preventing stiffness**.<br><br>Here are the usual first exercises (safe early-phase options), assuming your doctor has allowed gentle movement:<br><br>---<br><br>## 1. Pendulum (Codman) exercises<br><br>This is usually the **first and safest exercise**.<br><br>How to do it:<br><br>* Lean forward slightly (support yourself with the other arm on a table)<br>* Let the injured arm hang relaxed<br>* Gently swing it in small circles or forward/backward<br><br>Key points:<br><br>* The arm stays completely relaxed<br>* Movement comes from your body, not the shoulder muscles<br>* Very small, pain-free motion only<br><br>---	Since you are in the very early stages of recovery, your "exercises" shouldn't actually look like a workout. At **20 years old**, your goal right now is **activation**, not strength. You want to wake up the muscles without actually moving the "hinge" of the shoulder.<br><br>Here are the standard "Phase 1" exercises that are generally safe to start with, provided you have no sharp pain.<br><br>---<br><br>### 1. The "Posture" Exercise: Scapular Squeezes<br>This is the most important move. It trains the muscles that hold your shoulder blade steady, which is the foundation for your whole arm.<br>* **How to do it:** Sit or stand up straight. Gently pinch your shoulder blades together and downward (imagine trying to put them in your back pockets).<br>* **The Rule:** Hold for 5 seconds, relax, and repeat 10 times. **Do not move your arm at all.**<br><br>### 2. The "Stability" Exercise: Grip Strengthening<br>There is a neurological link between your grip and your shoulder stability. Squeezing your hand actually helps "turn on" the rotator cuff.<br>* **How to do it:** Use a stress ball, a rolled-up sock, or just make a firm fist.<br>* **The Rule:** Squeeze hard for 5 seconds, then release. Do this throughout the day, especially while wearing your sling.	Based on your specific situation—a 20-year-old with a first-time anterior dislocation and no fractures—the first exercises you should do are **Pendulum Swings** and **Isometric Internal/External Rotation**. These are the safest and most effective ways to wake up the muscles without moving the joint into a dangerous position.<br><br>You should **not** start with lifting the arm up overhead or using resistance bands just yet.<br><br>Here are the three specific exercises to begin with, ideally within the first week, as long as you are comfortable.<br><br>### 1. Pendulum Swings (Codman's Exercises)<br>**Goal:** To gently distract the joint, reduce pain, and keep the socket lubricated without using the torn muscles.<br>**How to do it:**<br>- Lean forward and support yourself with your **good arm** on a table or chair.<br>- Let the injured arm dangle completely loose like a dead weight.<br>- **Do not use your shoulder muscles.** Use your hips and legs to gently rock your body in a circle. This motion will cause your dangling arm to swing slightly.<br>- **Duration:** 2-3 minutes, 3 times per day.<br><br>### 2. Isometric External Rotation (Pushing Outward)<br>**Goal:** To safely activate the rotator cuff muscles that hold the ball in the socket.

	## 2. Hand, wrist, and elbow movements<br><br>These are very important even though they’re not directly the shoulder.<br><br>You can:<br><br>* Open and close your fist<br>* Bend and straighten your elbow<br>* Rotate your wrist<br><br>These help circulation and prevent stiffness while your shoulder rests.<br><br>---<br><br>## 3. Assisted gentle range-of-motion (only if comfortable)<br><br>Later in the early phase, you may add:<br><br>* Using your other hand to gently help lift the injured arm slightly forward<br>* Small, controlled movements close to your body<br><br>Important:<br><br>* No pushing into pain<br>* No forced stretching<br>* Stop if you feel instability<br><br>---<br><br>## 4. Scapular (shoulder blade) setting<br><br>Very gentle activation of the shoulder blade muscles:<br><br>* Sit or stand upright<br>* Gently pull your shoulder blades “back and down” slightly<br>* Hold for a few seconds, then relax<br><br>This helps build early stability without moving the joint.<br><br>---<br><br>## What you should NOT do yet<br><br>Avoid:<br><br>* Weights or resistance bands<br>* Push-ups or planks<br>* Overhead lifting	### 3. The "Stiffness" Preventer: Wrist & Elbow Drills<br>The shoulder is immobilized, but the rest of the arm shouldn't be.<br>* **Wrist:** Make circles with your hand and flex your wrist up and down.<br>* **Elbow:** Gently take your arm out of the sling (while sitting) and let it hang straight toward the floor to "unlock" the elbow. Slowly fold it back up and replace the sling.<br>* **The Rule:** Do this 3–5 times a day to prevent "frozen elbow."<br><br>### 4. The "Invisible" Workout: Isometrics<br>These are the most "advanced" moves for the first week. You contract the muscle, but **the arm does not move.**<br>* **Internal Rotation Pulse:** Place your "good" hand over the wrist of your injured arm. Gently try to pull your injured hand toward your stomach while resisting with your good hand.<br>* **The Rule:** You should feel the muscle in the front of your shoulder tighten, but the arm stays perfectly still. Hold for 5 seconds.<br><br>---<br><br>### Three Moves to AVOID (The "Never" List)<br>Until a Physical Therapist clears you, do **NOT** do these:<br>1. **Pendulums:** Leaning over and circling the arm. (Wait until at least Day 7-10 for this).<br>2. **Wall Crawls:** "Walking" your fingers up a wall. This puts the arm in a high-risk overhead position.<br>3. **Bicep Curls with Weight:** Even a light water bottle pulls on the labrum via the bicep tendon.<br><br>---<br><br>### **The "Safe Exercise" Checklist**<br>* **Pain Level:** It should never be more than a "2 out of 10." If it stabs, stop.<br>* **Frequency:** Short bursts (2–3 minutes) throughout the day are better than one long "workout."<br>* **Icing:** Always ice for 15 minutes **after** you do these movements to calm any irritation.<br><br>**The Expert Guide Question:**<br>Does it feel like your shoulder "twitches" or hurts when you try to squeeze your shoulder blades, or does that movement feel relatively stable?	**Important:** This exercise involves **no movement** of the shoulder joint. You push against an immovable object.<br>**How to do it:**<br>- Stand sideways next to a **door frame or a wall corner** with the injured arm closest to the wall.<br>- Bend your elbow to exactly **90 degrees** and keep it tucked tight against your ribs (this is crucial).<br>- Place the **back of your wrist** against the wall/door frame.<br>- Gently push outward as if you are trying to rotate your hand away from your body, but the wall stops you from moving.<br>- **Hold:** 5-10 seconds. **Rest:** 10 seconds. **Repeat:** 10 times.<br>- **Sensation:** You should feel a gentle ache deep in the back of the shoulder, **not** a sharp pinch in the front.<br><br>### 3. Isometric Internal Rotation (Pushing Inward)<br>**Goal:** Balance the muscles in the front of the shoulder safely.<br>**How to do it:**<br>- Stand facing the corner of a wall or the edge of a door frame.<br>- Bend your elbow 90 degrees, keep it tucked at your side.<br>- Place the **palm of your hand** flat against the edge of the door frame.<br>- Gently push inward as if trying to bring your hand toward your belly button.<br>- **Hold:** 5-10 seconds. **Repeat:** 10 times.<br><br>### 4. Scapular Retractions (Shoulder Blade Squeezes)<br>**Goal:** To prevent the "hunched" posture that makes the shoulder less stable.<br>**How to do it:**<br>- Sit or stand up straight.<br>- Without shrugging your shoulders up toward your ears, gently squeeze your shoulder blades together in the back.<br>- Imagine trying to hold a pencil between your shoulder blades.<br>- **Hold:** 5 seconds. **Repeat:** 15 times.<br><br>### The Exercise You Must Avoid Right Now<br>**Do NOT do "Empty Can" or "Full Can" Raises.** This is when you lift your arm out to the side or in front of you with your thumb pointing up or down. These are common rotator cuff exercises, but they place the arm in the **"apprehension position"** (abduction and external rotation). Doing these before Week 6-8 is a common cause of re-dislocation in young patients.<br><br>### How to Know It's Working (or Not)

	* Arm out to the side + rotated outward<br>* Any movement that feels like the shoulder might “slip”<br><br>---<br><br>## How often?<br><br>* 1–3 times per day is usually enough early on<br>* Each session should be short (5–10 minutes)<br>* Stop if pain increases or you feel instability<br><br>---<br><br>## Simple rule to follow<br><br>If the exercise:<br><br>* Feels easy ✓<br>* Keeps your arm relaxed ✓<br>* Does not move into uncomfortable positions ✓<br>→ it is likely safe<br><br>---<br><br>## Bottom line<br><br>Start with **pendulum exercises, elbow/wrist movement, and very gentle shoulder blade control**. These are the foundation of recovery and are designed to be safe while your shoulder heals.<br><br>---<br><br>If you want, I can build you a **simple 7-day home rehab plan** so you know exactly what to do each day without guessing or risking unsafe movements.		- **Good Sign:** Your shoulder feels a bit achy like you worked a muscle, but the ache fades within 20-30 minutes of stopping.<br>- **Bad Sign:** You feel a sharp, catching pain in the **front** of the shoulder (where the dislocation happened). If this happens, stop that specific exercise and try it again tomorrow with less force. If it keeps happening, wait until you see a physical therapist.
44	Yes—**you should stop (or reduce) the exercise if you feel pain**, especially sharp or increasing pain.<br><br>But it depends on *what kind of pain* you feel.<br><br>---<br><br>## 1. Pain you should STOP for<br><br>Stop the exercise immediately if you feel:<br><br>* Sharp or sudden pain in the shoulder<br>* A feeling that the shoulder is “slipping,” unstable, or shifting	**Yes, absolutely.** If you feel pain during your exercises, you need to stop immediately.<br><br>At 20, you might be used to the "no pain, no gain" mentality of the gym, but with a dislocated shoulder, **pain is a biological "stop sign"** telling you that you are pulling on tissues that aren't ready to be moved.<br><br>Here is how to tell the difference between "okay" sensations and "danger" pain:<br><br>### 1. The "2/10" Rule<br>In physical therapy, we use a 10-point scale.	Yes, you should stop doing that specific exercise if you feel a certain *type* of pain, but not all pain is a reason to stop completely. Understanding the difference between "healing ache" and "dangerous pain" is the key to a successful recovery without re-injury.<br><br>Here is the clear, plain-text guide for what to do when you feel discomfort during your shoulder rehab.<br><br>### The "Stop Immediately" Pain (Red Light)<br>You must stop the exercise right away if you experience any of the following:

	* Pain that increases as you continue the movement<br>* Catching, pinching, or a “giving way” sensation<br><br>These can be signs the movement is stressing healing structures too much.<br><br>---<br><br>## 2. Mild discomfort that can be normal<br><br>Some **light discomfort or muscle effort** can be expected, especially early on:<br><br>* Gentle stretching sensation<br>* Mild tiredness in the shoulder or upper arm<br>* Slight soreness after exercise (that settles within a few hours)<br><br>This type of discomfort is usually okay as long as it:<br><br>* Stays mild<br>* Does not worsen during the exercise<br>* Improves after rest<br><br>---<br><br>## 3. A simple rule to follow<br><br>* **Pain during movement = stop or reduce**<br>* **Mild effort or discomfort = okay**<br>* **Pain that lingers or increases afterward = you did too much**<br><br>---<br><br>## 4. What to do if pain happens<br><br>* Stop the exercise immediately<br>* Rest the arm in a comfortable position (close to your body or in a sling)<br>* Try again later with **smaller, gentler movement**<br>* If pain keeps happening, that exercise may be too advanced for now<br><br>---<br><br>## 5. Important reassurance<br><br>Early rehab is not about pushing through pain. For your type of injury:<br><br>* Progress comes from **safe, controlled movement**<br>* Not from intensity	* **0–2 (Safe):** A light stretch, a dull "tired" ache, or the feeling of the muscle working. This is usually fine.<br>* **3 or Higher (Stop):** Anything that feels like a **pinch, a stab, an electric shock, or a sharp catch.** This means you are putting stress on the tear in your labrum or a stretched ligament.<br><br>### 2. Pain "After" the Exercise<br>Sometimes the exercise feels fine while you're doing it, but two hours later your shoulder is **throbbing** or you can’t get comfortable at night.<br>* This is called **delayed inflammatory response.** It means you did too much and caused "micro-damage" to the healing tissue. If this happens, you should skip your exercises the next day and use ice.<br><br>### 3. The "Apprehension" Factor<br>This is unique to dislocations. Even if it doesn't "hurt," you might get a sudden, nauseating feeling that the arm is **unstable or about to slip.** * **Treat this exactly like pain.** If your brain is screaming *"No!"*, stop the movement. That is your nervous system's way of protecting you from a re-dislocation.<br><br>---<br><br>### What to do if you feel pain:<br>1. **Stop the movement immediately.** Don't try to "push through" to finish the set.<br>2. **Adjust the range:** If lifting your arm 20 degrees hurts, try lifting it only 5 degrees. If that still hurts, don't move it at all.<br>3. **Ice it:** Apply ice for 15–20 minutes to settle the nerves.<br>4. **Note the "Trigger":** Write down exactly which move caused the pain. When you see your Physical Therapist, tell them: *"I felt a sharp pinch in the front when I tried to move my hand outward."* This helps them diagnose exactly which part of your shoulder is damaged.<br><br>### The Risk of Pushing Through<br>If you ignore the pain and keep going, you can:<br>* **Turn a small tear into a big one.**<br>* **Create "Compensatory Patterns":** You’ll start using your neck and back muscles to "help" the shoulder, which leads to chronic neck pain and headaches.<br>* **Trigger a Spasm:** Your muscles might go into a protective "clinch" that makes the shoulder feel stiff for days.<br><br>> **Bottom Line:** You are 20 years old; you have plenty of time to get strong. Right now, your only job is to let the "glue" dry. If it hurts, the glue is being pulled apart.	- **Sharp, Pinching Pain in the Front:** If you feel a sudden, sharp catch or stab in the *front* of the shoulder—the exact same spot where the ball popped out of the socket. This is a sign that the ball is sliding forward in the loose capsule. Continuing the movement could cause a subluxation (partial slip) or full dislocation.<br>- **The Feeling of "Apprehension":** This is not exactly pain, but a deep, instinctive feeling of *"Oh no, this feels like it's going to pop out."* **Trust this feeling.** Your brain is warning you that the joint is nearing its unstable limit. If you push past this warning, the risk of re-dislocation is high.<br>- **Pain That Does Not Settle After Rest:** If you stop the exercise and sit down, but the shoulder continues to throb or ache intensely for more than **30-60 minutes**, you have irritated the healing tissues too much.<br><br>### The "Okay to Continue Gently" Discomfort (Yellow Light)<br>It is normal to feel the following sensations, and you can work *through* them, provided you reduce the intensity:<br><br>- **Muscle Burn or Fatigue:** A dull, tired ache in the back of the shoulder or around the shoulder blade. This is your rotator cuff waking up. It should feel like the burn you get when holding a plank, not a sharp knife.<br>- **General Stiffness:** After Pendulum Swings, the joint might feel a bit achy because you are moving fluid around in a swollen area. This is normal and will improve with gentle, consistent motion.<br>- **Stretch Sensation:** During the Pendulum or Scapular Squeezes, you might feel a pull in the back of the neck or upper back. That is fine.<br><br>### What to Do If You Feel the "Bad" Pain<br>1. **Stop the Repetition:** Do not finish the set.<br>2. **Check Your Form:** Look in a mirror. Did your elbow drift away from your side? Did your shoulder shrug up toward your ear? **Re-set your posture.** Pull your shoulder blades down and back, then try one more repetition with **50% less effort**.<br>3. **If It Still Hurts:** Skip that specific exercise for today. Try it again tomorrow or the day after. If a specific move (like the Isometric Outward Press) hurts every single time for 3-4 days in a row, remove it from your routine and wait until you can see a physical therapist for a modification.<br><br>### A Note for Your Age Group (20 Years Old)<br>Because you are young and the tissues are stretchy, the shoulder may feel "fine" even when it's dangerously loose. Do not rely on **strength** right now; rely on **control**. It is better to do the

<table>
<tr><td></td><td>* Avoiding re-injury is more important than “doing more”

Bottom line

Yes—**you should stop if the exercise causes pain that is sharp, worsening, or feels unstable**. Mild, controlled discomfort is okay, but pain is your signal to scale back.

If you want, I can help you distinguish exactly what “safe discomfort” feels like vs warning signs for shoulder instability in a very simple checklist.</td><td>Has a specific movement been causing you pain lately, or were you just asking to be prepared?</td><td>exercises with very light, perfect form than to push through a sharp pinch just to feel like you've done a "workout." The goal in the first month is not to get stronger—it is to prevent the joint from shifting forward while the scar tissue forms.</td></tr>
<tr><td>45</td><td>Yes—**it is very normal for early shoulder exercises to feel uncomfortable after a dislocation**, especially in the first days to a couple of weeks.

Why it feels uncomfortable

After your injury:

* The stabilizing tissues (labrum and ligaments) are still healing
* The muscles around your shoulder are “guarding” to protect it
* The joint is stiff from being in a sling and from inflammation
* Your brain is cautious, which makes movements feel strange or tense

So even gentle movements can feel:

* Tight
* Weak
* Slightly sore or awkward

What kind of discomfort is normal

You may feel:

* Mild stretching or pulling
* Muscle fatigue or weakness
* Light soreness during or after exercises
* Stiffness that slowly improves with movement

This type of discomfort should:

* Stay mild
* Not increase sharply during exercise</td><td>It is **completely normal** for these exercises to feel "weird," awkward, or even slightly uncomfortable at first. There is a big difference, however, between **discomfort** (which is expected) and **pain** (which is a warning).

Since you are 20 and used to your body just "working," this new sensation can be frustrating. Here is why those first few movements feel so strange:

1. Muscle "Amnesia"
When you dislocate your shoulder, your brain actually "shuts off" some of the stabilizer muscles (the rotator cuff) to protect them from further damage. When you start exercising, you are essentially trying to "wake up" muscles that have gone into hibernation. This creates a shaky, weak, and "clunky" feeling that can be very uncomfortable.

2. The "Rusty Hinge" Sensation
While you've been in a sling, the fluid in your joint (synovial fluid) has become thicker and the joint capsule has tightened up. Moving it for the first time is like opening a door with rusty hinges—it’s going to feel stiff, tight, and "creaky."

3. "Protective Guarding"
Your brain is currently "hyper-aware" of your shoulder. It is scanning for danger 24/7. When you move the arm, your brain sends a "danger" signal even if the movement is safe. This can manifest as a feeling of **apprehension** or a dull, heavy ache.

How to tell "Normal Discomfort" from "Danger Pain"

| It's Normal Discomfort If... | It's DANGER Pain If... |
| :--- | :--- |</td><td>Yes, it is **completely normal and expected** for these exercises to feel uncomfortable at first. In fact, if they felt completely normal and easy, it might be a sign that you are not actually engaging the specific muscles that need to be woken up.

The key distinction you need to make is between **Uncomfortable** and **Unsafe**. Here is what you should expect as a 20-year-old starting shoulder rehab after a first-time dislocation.

Why It Feels Uncomfortable (The Normal Reasons)
There are three specific reasons why the initial exercises feel "off" or achy, even when done correctly:

1. **Muscle Inhibition (Shutdown):** After a dislocation, the trauma causes your rotator cuff muscles to essentially "turn off" to protect the joint. When you do the Isometric Presses or Pendulums, you are forcing a muscle that has been napping to wake up and work. This feels **shaky, weak, and achy**. It's similar to the feeling of trying to write with your non-dominant hand.
2. **Fluid and Swelling:** The joint is still slightly swollen even if you can't see it on the outside. Moving it (even gently with Pendulums) pushes that fluid around. This creates a sensation of **pressure** or **dull ache** inside the joint.
3. **Scapular Fatigue:** You are likely holding your shoulder blade differently than normal. The muscles between your shoulder blade and spine are probably weak from sling use. Squeezing them (Scapular Retractions) will feel like a deep burn in the upper back. This is a **good sign**, even if it's not pleasant.

How to Differentiate "Good" Uncomfortable from "Bad" Pain</td></tr>
</table>

<table>
<tr><th></th><th></th><th></th><th></th></tr>
<tr><td></td><td>* Improve shortly after you stop

What is NOT normal

Stop and be cautious if you feel:

* Sharp or stabbing pain
* A feeling that the shoulder is slipping or unstable
* Pain that gets worse as you continue
* Increasing discomfort that doesn’t settle with rest

Key idea

Early rehab is about **retraining movement safely**, not pushing through pain. A bit of discomfort is expected, but it should feel like **gentle effort—not sharp or alarming pain**.

Bottom line

Yes, **mild discomfort at the start of exercises is normal and expected**, but it should be controlled and gradually improve over time—not worsen.

If you want, I can describe what normal progress feels like week by week so you know if your recovery is on track.</td><td>| It feels like a dull, tired ache in the muscle. | It feels like a **sharp, stabbing pinch** in the joint. |
| It feels "tight" or "stiff" like a hard stretch. | It feels like a **lightning bolt** or "zing" down the arm. |
| The feeling goes away within 5–10 minutes. | The pain lingers and makes it hard to sleep at night. |
| You feel "shaky" or weak while doing it. | You feel a **"clunk" or "shift"** inside the bone. |

Tips for Managing the Initial Discomfort
* **Warm up the "Good" side first:** Sometimes doing the movement with your uninjured arm first helps your brain "remember" the correct pattern, which makes the injured side feel less threatened.
* **Keep the movements "Micro":** If the exercise calls for a 5-inch movement and it’s uncomfortable, do a 1-inch movement. Success is about **repetition**, not distance.
* **Breathe:** Most people hold their breath when they're nervous about an injury. This tenses up your neck and makes the discomfort worse. Focus on slow, deep exhales while you move.
* **The "Ice Sandwich":** If you’re nervous, ice the shoulder for 5 minutes *before* you start to numb the surface nerves, and 15 minutes *after* to stop any inflammation.

The "Expert Guide" Question
Does the discomfort feel like it's deep inside the "ball" of the shoulder, or does it feel more like it's in the muscles on top (like your trap or bicep)?</td><td>This is the most important table for you to remember mentally. I'll describe it in plain text.

Sensation: Dull Ache / Muscle Burn
- **Location:** Deep in the back of the shoulder or between shoulder blades.
- **Timing:** Peaks during the hold, fades within 20 minutes of stopping.
- **Action:** **Continue.** This is healing. Reduce reps if it's too intense.

Sensation: Sharp Pinch / Catching
- **Location:** **Front** of the shoulder (the "dent" where the ball sits).
- **Timing:** Sudden, knife-like during the movement.
- **Action:** **STOP immediately.** Re-set form. Try again with 50% less effort. If still sharp, skip it for today.

Sensation: Apprehension / "About to Slip"
- **Location:** A mental/brain sensation more than a physical one.
- **Timing:** Feels like the joint is sliding forward.
- **Action:** **STOP immediately.** Do not push past this feeling.

A Realistic Expectation for the First Week
- **Pendulum Swings:** May feel like a dead weight dragging on the socket. This is normal. It should not cause a sharp pop.
- **Isometric External Rotation (Pushing Outward):** This will likely feel **very weak** and almost trembling. You might only be able to push at 10% effort without pain. That is **perfectly fine**. The goal is muscle activation, not moving a wall.

Summary for a 20-Year-Old Patient
You are used to your body being strong and responsive. After a dislocation, the shoulder is temporarily **stupid**. It has forgotten how to fire muscles in the right order. The discomfort you feel is your brain re-learning that connection. As long as the discomfort is a **dull ache in the back** and not a **sharp pinch in the front**, you are on the right track. Expect it to feel "clunky" and awkward for about 7 to 10 days before it starts to feel smoother</td></tr>
<tr><td>46</td><td>How often you should do shoulder exercises after a first dislocation depends on **how early you are in recovery and what type of exercises you’re doing**. The key is consistency, not intensity.

Early phase (first 1–2 weeks)</td><td>Since you’re 20 and your body heals relatively fast, you might be tempted to "grind" these exercises to get back to normal sooner. However, with a shoulder dislocation, **consistency and frequency** are much more important than **intensity**.</td><td>For the first phase of recovery after a first-time anterior dislocation, the general rule for frequency is **"Little and Often"** rather than "One Long Session."

You want to wake up the muscles and keep the joint fluid moving without causing inflammation or fatigue that leads to</td></tr>
</table>

	If you’re doing **gentle movements only** (like pendulums, elbow/wrist motion):<br><br>* **1–3 times per day**<br>* Each session: **5–10 minutes**<br>* Keep everything **light and pain-free**<br><br>This helps prevent stiffness without stressing the healing tissues.<br><br>---<br><br>## Early strengthening phase (usually after a few weeks, if cleared)<br><br>When your doctor/physio allows more active exercises:<br><br>* **1–2 times per day**<br>* Short sessions focusing on control and stability<br>* May include light isometric or band exercises (very low resistance)<br><br>---<br><br>## Later rehab phase (6+ weeks, guided progression)<br><br>As strength improves:<br><br>* **3–5 times per week**<br>* More structured strengthening sessions<br>* Longer sessions (15–30 minutes)<br><br>---<br><br>## Important rules regardless of stage<br><br>* Stop or reduce if pain increases<br>* Quality matters more than frequency<br>* Don’t “push through” instability or sharp pain<br>* Rest days are normal and helpful<br><br>---<br><br>## Simple summary<br><br>* Early: **small exercises, 1–3× daily**<br>* Later: **progress to strengthening 3–5× weekly**<br>* Always guided by pain and stability<br><br>---	Because these early exercises are low-intensity (mostly "waking up" the muscles), the goal is to do them **frequently throughout the day** rather than in one big workout session.<br><br>### 1. The "Short and Often" Rule<br>Think of your exercises like taking a dose of medicine. You want a steady stream of "stability signals" going to your brain.<br>* **Frequency:** **3 to 4 times per day.**<br>* **Duration:** Each "session" should only last about **5 to 10 minutes**.<br>* **The Benefit:** Short sessions prevent your muscles from getting too tired. Remember: a tired shoulder is an unstable shoulder.<br><br>### 2. The Daily Schedule (Example)<br>* **Morning:** Do a set of scapular squeezes and wrist/elbow movements before you head to class or start your day. This "resets" your posture after sleeping.<br>* **Lunchtime:** A quick round of isometric pulses (pushing against your stomach) to wake up the stabilizers after sitting at a desk.<br>* **After School/Work:** Another set of movements followed by 20 minutes of **icing**.<br>* **Before Bed:** A final round of gentle movements to ensure you aren't "tightening up" overnight.<br><br>### 3. Repetitions and Sets<br>For most Phase 1 exercises (the ones that don't move the joint):<br>* **Hold time:** 5 to 10 seconds per contraction.<br>* **Reps:** 10 repetitions.<br>* **Sets:** 1 to 2 sets per session.<br><br>### 4. Let Pain Be Your "Volume Knob"<br>If you find that your shoulder is **throbbing more at night**, you are likely doing your exercises too often or too hard.<br>* **If it's "Angry":** Drop down to 1 or 2 sessions a day until the inflammation settles.<br>* **If it feels "Easy":** Don't add weight! Instead, focus on the *quality* of the contraction. Squeeze the muscle harder and hold it longer while maintaining perfect posture.<br><br>---<br><br>### **A Note on "The Sling Gap"**<br>When you take your sling off to do your exercises, your shoulder is at its most vulnerable.<br>* **Always do your exercises while sitting or standing in a safe spot.** * **Never** do them while walking or in a crowded area.<br>* Once you finish your reps, put the sling back on immediately.	bad form. Here is the specific, plain-text guidance for a 20-year-old managing this at home.<br><br>### The Recommended Daily Schedule<br><br>**1. Pendulum Swings and Wrist/Elbow Motion**<br>- **Frequency:** **3 to 4 times per day.**<br>- **Duration:** 2 to 3 minutes each session.<br>- **Best Times:** Morning (to loosen stiffness from sleeping), Midday, and Evening.<br>- **Note:** You cannot really "overdo" pendulums as long as you are letting gravity do the work and not using muscle to swing the arm.<br><br>**2. Isometric Exercises (Pushing against wall/doorframe)**<br>- **Frequency:** **1 to 2 times per day.** (Doing them twice a day is ideal, but once is acceptable if you are sore).<br>- **Reps and Sets:** Aim for 2 sets of 10 holds. Each hold is 5-10 seconds.<br>- **Crucial Rule:** **Do NOT do these every hour.** These are activating the rotator cuff. Even though the joint isn't moving, the muscle is working. If you do them all day long, you will cause muscle fatigue and soreness that mimics injury pain.<br><br>**3. Scapular Squeezes (Shoulder Blade Pinches)**<br>- **Frequency:** **As often as you think about it.** (5 to 10 times per day).<br>- **Why so often?** You are fighting the natural tendency to slump and hunch over a desk or phone. Every time you check your phone or look at a screen, do 5 squeezes. This is about posture retraining more than pure exercise.<br><br>### The "Day On, Day Off" Debate<br>You might wonder if you should rest a day between exercises like you would with weightlifting.<br>- **For the first 3 weeks:** Do **not** take full rest days from the *gentle* range of motion. The shoulder joint fluid (synovial fluid) needs movement to nourish the cartilage. A full day of being completely still in the sling (except for sleeping) will make the shoulder feel **more** stiff and painful the next morning.<br>- **Exception:** If you had a very busy day using the arm a lot (carrying a heavy backpack with the good arm, writing a 3-hour exam, etc.), you may skip the *Isometric* exercises that night if the shoulder feels hot or throbbing. But still do 1 minute of Pendulums just to calm it down.<br><br>### Signs You Are Doing Too Much (Overtraining the Injury)

	## Bottom line<br><br>You should do exercises **regularly but gently**, starting with short daily sessions, then gradually reducing frequency as intensity increases later in rehab.<br><br>---<br><br>If you want, I can turn this into a **simple weekly schedule (day-by-day plan)** so you know exactly what to do at each stage of recovery.	### **The "Expert Guide" Question**<br>Does your shoulder tend to feel stiffer and more uncomfortable in the morning when you wake up, or at the end of the day after you've been moving around?	Because you are 20 and likely used to pushing through workouts, watch for these specific signs that you need to reduce frequency to **once per day**:<br><br>- **Increased Night Pain:** If you wake up in the middle of the night with a deep ache that wasn't there before. This is a classic sign of joint inflammation from too much activity.<br>- **Muscle Twitching:** If the muscles around the shoulder blade won't stop twitching or spasming, they are exhausted. Rest them for 24 hours.<br><br>### Summary Checklist for Your Phone Lock Screen<br>- **Pendulums:** 3x per day (3 min each).<br>- **Wall Pushes (Isometrics):** 2x per day (2 sets of 10).<br>- **Posture Squeezes:** Every time you look at TikTok/Instagram.<br><br>Consistency with this frequency is more important than intensity. Doing these small bouts of movement throughout the day is what prevents the dreaded "frozen shoulder" complication while keeping the joint safe.
47	A good question—because with shoulder rehab after a dislocation, **doing the right exercises the wrong way can matter as much as doing the right ones.** You don’t need perfection, but you *do* want safe, controlled movement.<br><br>Here are practical ways to check if you’re doing them correctly:<br><br>---<br><br>## 1. Pain pattern is your first guide<br><br>You’re likely doing it correctly if:<br><br>* Movement feels **mildly uncomfortable but not painful**<br>* Discomfort stays **steady and does not increase**<br>* Any soreness settles within a short time after finishing<br><br>You’re likely doing it incorrectly if:<br><br>* Sharp pain appears during the movement<br>* Pain increases the more you repeat it<br>* You feel “slipping,” instability, or catching<br><br>---<br><br>## 2. The movement feels “controlled,” not forced<br><br>Correct exercises feel like:	Knowing if you’re doing shoulder exercises correctly is tricky because, at 20, your body is very good at "cheating." If a muscle is weak or painful, your brain will automatically recruit a different muscle to do the heavy lifting without you even realizing it.<br><br>Here is how you can "audit" your form to make sure you’re actually helping the injury:<br><br>### 1. The Mirror Test<br>This is your most valuable tool. Take your shirt off (or wear a tank top) and stand in front of a mirror.<br>* **Look at your "Good" shoulder:** Do an exercise on that side first to see what a healthy shoulder looks like.<br>* **Watch for the "Shrug":** The #1 mistake is "hiking" your shoulder toward your ear. If you see your injured shoulder climbing up while you try to move your hand or squeeze your blades, you are using your **trapezius** (neck muscle) instead of your **rotator cuff**.<br>* **The Fix:** Consciously drive your shoulder blade down "into your back pocket" before you start any movement.<br><br>### 2. The "Touch" Test<br>Use your healthy hand to feel the muscles on your injured side while you do an isometric exercise.<br>* **Internal Rotation:** If you are pressing your hand against your stomach, you should feel the muscle in the front of your shoulder/armpit (subscapularis) get firm.	This is one of the most important questions to ask because with shoulder rehab, **how you do the exercise matters far more than how many you do.** Since you are likely doing these at home without a physical therapist watching your every move, you need to become your own "form coach."<br><br>Here are the specific, tangible checks you can use to confirm you are doing the three main early exercises correctly.<br><br>### 1. The "Mirror Test" for All Exercises<br>Before you even start the movement, stand sideways to a full-length mirror (or use your phone camera on video mode).<br>- **Check Your Neck:** Look at the muscle that runs from your neck to the top of your shoulder (the Upper Trapezius). **Is it bulging and tight? Is your shoulder hiked up toward your ear?**<br>- **Correct Form:** The neck should look relaxed and long. The top of the shoulder should be level with the other side.<br>- **If Wrong:** If you see your shoulder creeping up to your ear, stop. Take a deep breath, let the shoulder drop down, and try again with **half the effort**. At this stage, you are training the shoulder to move *without* the neck helping.<br><br>### 2. Specific Checks for Each Exercise<br><br>**For Pendulum Swings**<br>- **How You Know It's Wrong:** You feel the muscles on top of your shoulder (Deltoid) burning or getting tired. You see your arm swinging in a big, fast circle.

	 * Slow and smooth motion<br>* No sudden jerks<br>* You are not “forcing” range of motion<br><br>Incorrect form often feels like:<br><br>* You are pushing through tight resistance<br>* The shoulder feels like it is being stretched aggressively<br>* You lose control of the arm at any point<br><br>---<br><br>## 3. Your shoulder stays “relaxed and close”<br><br>For early rehab (like pendulums):<br><br>* The arm should feel **heavy and relaxed**<br>* The movement should come from your body (leaning/swaying), not the shoulder muscles<br><br>If you notice:<br><br>* Tensing or shrugging your shoulder up<br>* Actively lifting the arm instead of letting it move passively<br>→ you’re likely overusing it<br><br>---<br><br>## 4. No feeling of instability<br><br>A key safety sign:<br><br>* The shoulder should **feel stable, even if weak**<br><br>Warning sign:<br><br>* Feeling like it might “slip out,” shift, or not sit correctly in the joint<br><br>---<br><br>## 5. After-exercise response<br><br>Correct exercise usually:<br><br>* Causes mild tiredness or soreness<br>* Improves or stays the same over the next few hours<br><br>Incorrect exercise often:	* **The "Trap" Check:** Place your good hand on your neck. If those muscles feel like they are "firing" or getting hard while you're just trying to move your wrist or elbow, you're "cheating."<br><br>### 3. The "Peripheral Vision" Rule<br>For early exercises, your elbow should **always** be in your sight.<br>* If you look straight ahead and you can’t see your elbow out of the corner of your eye, your arm has drifted too far back. This puts your shoulder in an unstable "open" position.<br>* **The Fix:** Keep your elbow slightly in front of your ribcage.<br><br>### 4. Quality Over Quantity<br>In the gym, people focus on how many reps they can do. In rehab, we focus on **smoothness.**<br>* **The Sign of Success:** The movement should be slow, controlled, and "quiet."<br>* **The Sign of Failure:** If your arm is shaking, ratcheting (moving in little jumps), or making a "clunking" sound, the stabilizing muscles aren't firing correctly. Reduce the effort until the movement is smooth.<br><br>---<br><br>### **Signs You Are Doing It RIGHT:**<br>* You feel a dull "work" ache in the muscles, but no sharp pain in the joint.<br>* Your posture stays tall; you aren't leaning or twisting your torso to help the arm move.<br>* After the session, your shoulder feels "tired" but not "angry" or throbbing.<br><br>### **Signs You Are Doing It WRONG:**<br>* You have a tension headache or neck pain after your exercises.<br>* You feel a "pinch" in the front of the shoulder.<br>* You have to hold your breath to finish the repetitions.<br><br>### **The Gold Standard: Professional Eyes**<br>The reality is that nothing replaces a **Physical Therapist** watching you in person. They can see "micro-cheats" (like a slight wrist flick or a rib flare) that are impossible to catch yourself. Once they confirm your form is perfect for 1–2 sessions, you’ll have the "muscle memory" to do them safely at home.<br><br>**The Expert Guide Question:**<br>When you try to move your arm now, do you feel like you have to "hike" your shoulder up to get it to move, or can you keep your shoulder blade settled down while you move?	- **How You Know It's Correct:** Your arm should look like a **wet noodle** or a dead weight. You should feel the motion coming from your **hips and legs**, not your shoulder. If someone gently poked your shoulder muscle while you were doing it, it should feel soft and relaxed. The movement should be a small circle (about the size of a dinner plate), not a big windmill.<br><br>**For Isometric External Rotation (Pushing Outward Against Wall)**<br>- **The "Paper Towel" Trick:** This is the gold standard test for this exercise. Roll up a small hand towel or a few paper towels. **Place it between your injured elbow and your ribs.** Squeeze it gently to hold it there.<br>- **How You Know It's Wrong:** The towel drops to the floor. This means your elbow drifted away from your body. When the elbow drifts, the shoulder rotates into the **danger zone** for re-dislocation.<br>- **How You Know It's Correct:** You push outward with the back of your wrist, but the elbow stays glued to your side, keeping the towel in place. You should feel a dull ache deep in the **back** of the shoulder blade, not the top of the shoulder.<br><br>**For Isometric Internal Rotation (Pushing Inward Against Door Frame)**<br>- **How You Know It's Wrong:** You feel a sharp pinch in the **front** of the shoulder joint (the dent where the ball sits). You also see your whole body leaning into the door frame to help push.<br>- **How You Know It's Correct:** Only the palm moves toward the belly. You feel a muscle contraction in the front of the chest/armpit, but the shoulder joint itself feels **quiet and still**. Remember: **No visible movement of the arm.**<br><br>### 3. The "Day After" Test (Most Reliable Indicator)<br>How you feel **the next morning** is the ultimate report card on whether you did the exercises correctly the day before.<br><br>- **Sign You Did It Correctly:** You wake up with a mild, generalized stiffness that feels like you "slept funny." This stiffness goes away within 5-10 minutes of getting up and walking around or doing your morning Pendulums.<br>- **Sign You Did It Incorrectly (or Too Much):** You wake up and the shoulder feels **hot, swollen, or sharply painful** in the exact same spot as the original dislocation. This indicates you irritated the joint capsule. If this happens, take the day off from Isometrics and stick to just Pendulums.<br><br>### 4. The "Apprehension" Check (Mental Check)

	* Makes pain worse later in the day<br>* Increases stiffness or discomfort significantly<br><br>---<br><br>## 6. A simple self-check rule<br><br>After each session ask:<br><br>* Did I stay pain-free or only mildly uncomfortable?<br>* Did I avoid forcing the movement?<br>* Does my shoulder feel the same or better afterward?<br><br>If yes → you're likely doing it correctly.<br><br>---<br><br>## When to get help<br><br>It's a good idea to see a physiotherapist if:<br><br>* You're unsure about technique<br>* Pain keeps returning with basic exercises<br>* You feel instability even with gentle movement<br><br>Even one session can correct technique and give you confidence.<br><br>---<br><br>## Bottom line<br><br>You're doing exercises correctly if they are **slow, controlled, pain-free (or mildly uncomfortable), and do not cause instability or worsening symptoms afterward**.<br><br>---<br><br>If you want, I can also show you **what correct pendulum exercises and scapular exercises should feel like step-by-step**, since those are the most common ones people worry about.		This is particularly important for a 20-year-old. Your brain has a memory of the trauma.<br>- **Correct Feeling:** The exercise feels like a boring, annoying chore. You might feel a dull muscle burn, but your brain is calm.<br>- **Incorrect Feeling:** You feel a flash of anxiety or a mental image of the shoulder popping out just as you start to push. **Stop.** Even if it doesn't hurt, that mental signal means the joint is nearing its unstable limit. Back off the pressure by 50% until the anxiety fades.<br><br>**Summary Rule of Thumb**<br>If you are unsure, **do less pressure and fewer reps.** You cannot under-train this injury in the first 3 weeks, but you can absolutely over-stress it and cause a setback. When in doubt, film a 10-second video of yourself doing the exercise and save it to show your doctor or physical therapist at the next visit. They can spot a "shoulder shrug" or "elbow drift" in two seconds that you might not feel at all.
48	Yes—**skipping your shoulder exercises can slow recovery and sometimes lead to problems**, but the impact depends on how often you skip and what stage of healing you're in.<br><br>After a first anterior shoulder dislocation, rehab exercises are important because they help your shoulder regain **movement, strength, and stability**.	The short answer is **yes**. Skipping your exercises doesn't just slow you down; it can actually change the "quality" of your recovery.<br><br>At **20**, your body has a high capacity for healing, but it also has a high capacity for developing **bad habits.** If you skip the boring, small movements, you aren't just "resting"—you're letting the joint settle into a weakened state.	Yes, skipping your exercises can significantly slow your recovery and lead to two specific, long-term problems that are very common in 20-year-old patients after a first-time dislocation.<br><br>It is tempting to skip the exercises because they seem boring, uncomfortable, or because the shoulder "feels fine" just hanging

	---<br><br>## What can happen if you skip exercises often<br><br>### 1. Stiffness and reduced range of motion<br><br>* The shoulder can become tight, especially early on<br>* Simple movements (reaching, dressing, lifting arm) may stay difficult longer<br><br>---<br><br>### 2. Slower muscle recovery<br><br>* The small stabilizing muscles around your shoulder (rotator cuff) weaken quickly after injury<br>* Without activation, they take longer to “wake up” again<br><br>---<br><br>### 3. Poor coordination and stability<br><br>* The shoulder relies heavily on muscle control for stability<br>* Skipping rehab can delay your body relearning how to keep the joint secure<br><br>---<br><br>### 4. Longer overall recovery time<br><br>* You may still heal, but it can take longer to return to normal activities or sports<br><br>---<br><br>## What skipping does NOT usually do<br><br>* Missing a session or two **will not cause permanent damage**<br>* It does not automatically cause another dislocation by itself<br>* Occasional rest days are actually normal and sometimes helpful<br><br>---<br><br>## Important balance<br><br>* **Too little exercise → stiffness and slow recovery**<br>* **Too much too soon → risk of irritation or instability**<br>* The goal is **consistent, gentle, regular movement**	Here is why skipping the "homework" causes real problems:<br><br>### 1. The "Loose Hinge" Problem<br>When a shoulder dislocates, the ligaments get stretched out like an old rubber band. They never fully "snap back" on their own. The only way to keep the joint tight is to train the **rotator cuff muscles** to act like active "straps" that pull the bone into the socket. If you skip exercises, those muscles stay weak, and your shoulder remains "loose," making a second dislocation much more likely.<br><br>### 2. The "Frozen" Risk<br>The flip side of a loose shoulder is a **stiff** one. If you skip your gentle range-of-motion and elbow/wrist drills, your joint capsule can start to thicken and scar down. This is called **"Adhesive Capsulitis" (Frozen Shoulder).** If that happens, your recovery goes from a few weeks to several months of painful stretching to get your movement back.<br><br>### 3. Permanent "Cheating"<br>If you don't do the exercises to "wake up" the correct muscles, your brain will permanently delegate those jobs to your neck and upper back.<br>* **The Result:** You might get your shoulder moving again, but you'll end up with chronic tension headaches and neck pain because those muscles are doing a job they weren't designed for.<br><br>### 4. The "Scar Tissue" Quality<br>Scar tissue is like internal "glue." If you move the joint (gently and safely), that glue dries in a neat, organized way that allows for movement. If you stay completely still and skip exercises, the glue dries in a messy "clump" that is brittle and easy to re-tear later.<br><br>---<br><br>### **What happens if you skip just one day?**<br>Don't panic—skipping one day because you're exhausted or in extra pain won't ruin your life. In fact, **rest is part of recovery.** **The Danger Zone** is when you skip **3 or more days in a row.** That’s when:<br>* Muscles start to "atrophy" (waste away).<br>* The "neurological connection" between your brain and your shoulder gets fuzzy.<br>* Stiffness starts to set in.<br><br>### **The "Bare Minimum" Strategy**<br>If you’re having a busy day at school or you're just not feeling it, don't skip entirely. Do the **"Two-Minute Drill"**:<br>* 5 Scapular Squeezes (to fix your posture).	there in the sling. However, the consequences of inactivity during this critical 4-to-6-week window are very real.<br><br>Here is exactly what happens if you skip the routine we discussed earlier.<br><br>### Problem 1: Frozen Shoulder (Adhesive Capsulitis)<br>This is the most direct consequence of skipping Pendulums and gentle range of motion.<br>- **What Happens:** The capsule (the balloon of tissue surrounding the joint) is irritated from the dislocation. If you don't move it, the walls of that balloon become sticky and shrink-wrap around the ball of the shoulder. Scar tissue forms in the nooks and crannies of the joint.<br>- **The Result:** In about 2-3 months, you will try to reach up to grab a book or put on a shirt and **you physically will not be able to lift your arm past your shoulder.** It is like hitting a brick wall.<br>- **The Fix:** If Frozen Shoulder sets in, recovery is not measured in weeks—it is measured in **months to years**. It requires aggressive, painful stretching (sometimes under anesthesia) to tear that scar tissue loose. Doing **3 minutes of Pendulums 3 times a day** prevents this almost entirely.<br><br>### Problem 2: Muscle Imbalance and Re-Dislocation<br>This is the consequence of skipping the Isometric and Scapular exercises.<br>- **What Happens:** After a dislocation, the rotator cuff muscles (the tiny ones deep in the back) shut off. If you don't do the specific exercises to wake them up, your body will use the big, bulky "mirror muscles" (Deltoid and Pecs) to lift the arm instead.<br>- **The Result:** Those big muscles pull the ball of the shoulder **up and forward** in the socket. Since the ligaments in the *front* are already torn and loose, pulling the ball forward pushes it right into the unstable danger zone.<br>- **The Fix:** You will feel "recovered" and return to sports or the gym, but the first time you reach out to catch a ball or push open a heavy door, the shoulder will dislocate **again**. At age 20, the recurrence rate for non-operative treatment is already high (some studies suggest up to 80-90%). Skipping the exercises that strengthen the **back** of the shoulder makes that recurrence almost a certainty.<br><br>### What About Just "Resting" for a Few Weeks?<br>You might think resting is the safest option. In the first **5 to 7 days**, rest is indeed crucial. But after that first week, **rest becomes the enemy.**

	---<br><br>## What matters most<br><br>Consistency over time is more important than perfection:<br><br>* Short daily sessions are better than occasional long ones<br>* Gentle movement is more important than intensity early on<br><br>---<br><br>## Bottom line<br><br>Yes, **regular exercises help speed up recovery and improve shoulder stability**, while frequent skipping can slow progress—but occasional missed sessions are not a major problem.<br><br>---<br><br>If you want, I can help you set a **simple realistic routine that fits your school day**, so it's easier to stay consistent without feeling overwhelmed.	* 10 Fist Squeezes (to keep the forearm active).<br>* 1 minute of letting the arm hang (to prevent elbow stiffness).<br><br>Doing the "bare minimum" keeps the habit alive and tells your brain that the shoulder is still a "priority zone."<br><br>**The Expert Guide Question:**<br>Are you finding it hard to fit the exercises into your school schedule, or is it more that they feel too boring/small to be doing anything?	- **Weeks 1-2:** Skipping exercises = Stiffness begins. You lose the ability to lay your arm flat on a table.<br>- **Weeks 3-4:** Skipping exercises = Muscle atrophy. The back of your shoulder blade becomes visibly flatter and weaker than the other side.<br>- **Week 6+:** Skipping exercises = You are now at risk for both stiffness **and** instability simultaneously. This is the hardest scenario to treat.<br><br>### How to Stay on Track (Even When You Don't Want To)<br>If you find yourself skipping because you forget or it's a hassle:<br>- **Habit Stacking:** Do your Pendulum Swings while waiting for the coffee to brew or the microwave to finish. Do your Scapular Squeezes every time you stop at a red light.<br>- **One is Better Than None:** If you only have time for one set of Isometrics, **do it.** Partial compliance is infinitely better than zero compliance.<br><br>**Summary for a 20-Year-Old Patient**<br>Skipping these exercises does not just "delay" recovery; it changes the **destination** of your recovery. Doing them leads to a stable, functional shoulder. Skipping them leads to a stiff, unstable shoulder that is very likely to pop out again once you return to normal life. The small inconvenience of the home program now saves you from a much larger, more painful inconvenience later.
49	Yes—**strengthening your shoulder can significantly reduce the risk of it coming out again**, but it does not completely eliminate the risk on its own.<br><br>After a first anterior shoulder dislocation, the shoulder becomes unstable mainly because:<br><br>* The **labrum and ligaments are stretched or torn**<br>* The joint relies more heavily on muscles for stability<br><br>Rehab works by making those muscles better at "holding" the joint in place.<br><br>---<br><br>## How strengthening helps<br><br>### 1. Improves dynamic stability<br><br>* The rotator cuff and shoulder blade muscles act like "active stabilizers"<br>* Strong, well-coordinated muscles help keep the joint centered during movement	**Absolutely. In fact, strengthening is your primary defense against a lifelong problem.**<br><br>At 20, you are in a "golden window." Because your ligaments (the passive stabilizers) were likely stretched or torn during the dislocation, they are no longer as reliable at holding the "ball" in the "socket." Strengthening the muscles around the joint turns them into **active stabilizers.**<br><br>Here is why a strong shoulder is essentially a "biological brace":<br><br>### 1. The Rotator Cuff "Suction" Effect<br>The rotator cuff isn't just one muscle; it's a group of four. When they are strong and firing correctly, they act like a **suction cup**. They pull the head of the humerus (the ball) deep into the glenoid (the socket). Even if your ligaments are a bit loose, a strong rotator cuff can "clinch" the joint shut during movement, preventing it from sliding out.<br><br>### 2. "Armor" for the Joint<br>Think of your larger muscles (deltoids, pecs, and lats) as the outer armor. When you have good muscle mass and strength, these muscles absorb the "shock" of sudden movements—like catching a	Yes, **strengthening your shoulder is one of the most important things you can do to help stop it from coming out again, but it is not a guarantee.** Understanding the difference between what muscle strength *can* fix versus what it *cannot* fix is essential for managing your expectations as a 20-year-old patient.<br><br>Here is the clear breakdown of why rehab exercises are non-negotiable for stability, and where their limitations lie.<br><br>### How Strengthening Protects Your Shoulder<br>When your shoulder dislocated, it overstretched the passive restraints—the ligaments and labrum that act like a rubber band and bumper around the socket. These soft tissues heal, but they are often permanently looser than before the injury. This is where strengthening comes in to compensate.<br><br>- **Dynamic Stabilization:** Your rotator cuff and shoulder blade muscles act as the "active" control system. Strengthening these muscles helps press the ball of the shoulder firmly into the socket during movement, compensating for the looser ligaments<br>.<br>- **Scapular Control is Key:** Research emphasizes that you cannot just strengthen the rotator cuff in isolation. You must

	---<br><br>### 2. Reduces risky movement control problems<br><br>* After dislocation, the brain becomes cautious and coordination is off<br>* Rehab retrains your nervous system to control the shoulder safely<br><br>---<br><br>### 3. Protects you during daily activities and sports<br><br>* Strong muscles help absorb forces from:<br><br>* Lifting<br>* Reaching<br>* Falls or sudden movements<br><br>---<br><br>## But here’s the important limitation<br><br>Strengthening **cannot fully “repair” torn ligaments or a labral injury**. That means:<br><br>* Some structural looseness may remain<br>* Especially in young people (like age 20), the recurrence risk is naturally higher<br><br>So:<br><br>* Strength training = **major risk reduction**<br>* But not a 100% guarantee<br><br>---<br><br>## What lowers recurrence risk the most<br><br>The best protection comes from a combination of:<br><br>* Proper rehab (strength + control)<br>* Avoiding early risky positions (especially arm out + rotated outward)<br>* Gradual return to sports<br>* Sometimes surgery in high-risk or recurrent cases<br><br>---<br><br>## Bottom line	ball or being bumped in a hallway—before that force can reach the vulnerable internal ligaments.<br><br>### 3. Neuromuscular Control (The "Brain-Muscle" Connection)<br>Strengthening isn't just about size; it's about **proprioception** (your brain’s ability to know where your arm is without looking).<br>* **The Benefit:** A well-trained shoulder "reacts" faster. If you trip or stumble, a strong, well-trained shoulder will reflexively contract to protect itself. A weak shoulder will just hang there and let the momentum pull it out of place.<br><br>---<br><br>### The Reality Check: Strength vs. Ligaments<br>While strengthening is your best tool, it’s important to understand the limits:<br>* **Muscles can only do so much:** If the structural damage (the labrum tear) is severe enough, even the strongest muscles in the world might not be able to hold the joint during high-impact sports (like football or wrestling).<br>* **Consistency is Key:** Unlike a broken bone that heals and stays strong, muscles can get weak again if you stop training. For someone who has dislocated their shoulder, "Shoulder Day" becomes a permanent, lifelong part of your gym routine to keep that "brace" tight.<br><br>### **The Stats**<br>* For people in their 20s who **do not** follow a strict strengthening program after a dislocation, the re-dislocation rate can be as high as **70–90%**.<br>* For those who **consistently strengthen** and follow physical therapy, that risk drops significantly.<br><br>### **Where to Focus**<br>You don't want to just do "bench press" and "bicep curls." To prevent a re-dislocation, the most important muscles to strengthen are:<br>1. **The Rotator Cuff** (Internal and External rotation).<br>2. **The Scapular Stabilizers** (The muscles around your shoulder blades).<br>3. **The Serratus Anterior** (The muscles under your armpit).<br><br>> **Bottom Line:** Strengthening is the difference between a shoulder that works "most of the time" and a shoulder you can actually trust again.<br><br>Do you have any specific sports or activities (like swimming, lifting, or gaming) that you're hoping to get back to once your strength is up?	strengthen the muscles that control your shoulder blade (scapula). Proper scapular positioning ensures the shoulder socket is in the best possible orientation to catch the ball if it starts to slip. Without this, the rotator cuff is inefficient and the risk of re-injury increases .<br>- **The Result:** A dedicated, well-structured physical therapy program significantly improves the neuromuscular control of the joint. For many patients, this restores enough functional stability to return to daily life and non-contact sports without fear.<br><br>### The Important Limitation: What Strengthening *Cannot* Fix<br>You need to be aware of the statistics, particularly for patients under 25. While rehab helps, the structural damage inside the joint remains the primary driver of recurrence.<br>- **The Recurrence Reality:** In young, active patients treated with just a sling and rehab (non-operative management), the rate of the shoulder dislocating again can be very high. One analysis showed a recurrence rate of approximately **53%** with conservative treatment, compared to just **7.5%** for those who had surgery . Another study found that in 20-year-old athletes managed non-operatively, **80%** developed recurrent instability .<br>- **Why?** Muscle strength cannot fully replace a torn piece of cartilage (Bankart lesion). No matter how strong your muscles are, if you move your arm into the vulnerable "throwing" position with enough force, the ball can simply roll off the front of the socket because the physical bumper is gone.<br><br>### What This Means for Your Recovery<br>The approach is to **maximize the benefit of strengthening while acknowledging its limits.**<br>- **Commit to Rehab:** You should absolutely follow the exercise plan and progress to a full strengthening program under guidance. This is your best defense against a loose joint and is required regardless of whether you eventually need surgery.<br>- **Monitor for "Apprehension":** If, during your recovery or return to sport, you start to feel that the shoulder is "slipping," "shifting," or you feel a wave of anxiety with the arm in certain positions, this is called apprehension. In a young patient, this is often a sign that the structural damage is too significant for muscles alone to control.<br>- **Surgery as a Backup:** If strengthening fails and the shoulder feels unstable or dislocates again, **arthroscopic Bankart repair** (a surgery to stitch the torn labrum back down) is highly effective. In cost and quality-of-life models for 20-year-olds with a first-time dislocation, early surgical stabilization is often shown to be more cost-effective in the long run than

	Yes—**strengthening your shoulder is one of the most important things you can do to prevent it from dislocating again**, because it improves stability and control. But it works best as part of a full rehab process, not alone.<br><br>---<br><br>If you want, I can explain which specific muscle groups are most important for preventing redislocation and how they are trained in simple rehab steps.		repeating multiple rounds of failed rehab and suffering repeat dislocations .<br><br>**In summary:** Yes, strengthening your shoulder—especially the rotator cuff and scapular muscles—is critical for preventing another dislocation. It is the foundation of your recovery. However, be aware that due to the nature of the injury in young adults, strengthening alone fails to prevent recurrence in a significant number of cases. Stick to your rehab plan diligently, but stay in close communication with your doctor about any feelings of looseness or instability.
50	You can start **strengthening your shoulder only after the very early healing phase**, and it should be done in stages—not all at once.<br><br>For a first anterior shoulder dislocation like yours, the timeline is usually:<br><br>---<br><br>## 1. Early phase (first few days to ~1–2 weeks)<br><br>**Goal: protect + gentle movement only**<br><br>You should NOT do strengthening yet.<br><br>You can do:<br><br>* Pendulum exercises<br>* Hand, wrist, elbow movement<br>* Very gentle assisted motion (if pain-free)<br><br>No resistance, no weights, no bands.<br><br>---<br><br>## 2. Early activation phase (~2–4 weeks, depending on recovery)<br><br>**This is when very light strengthening may begin (if your doctor/physio allows it).**<br><br>You may start:<br><br>* Light isometric exercises (muscle tightening without movement)<br>* Gentle scapular (shoulder blade) control exercises<br><br>Examples:<br><br>* Pressing arm gently against a wall without moving it<br>* Light “shoulder blade squeeze” exercises	Since you're **20**, your muscles will likely respond quickly, but the "strength" phase of recovery is a marathon, not a sprint. You can't build a fortress on a foundation of wet cement.<br><br>The short answer: You usually start **true strengthening** (using resistance like bands or weights) around **6 weeks** after the injury.<br><br>Here is the step-by-step breakdown of how you "level up" to strength:<br><br>---<br><br>### Phase 1: The "Wake Up" (Now – Week 2)<br>You aren't building "muscle size" yet; you’re just making sure the muscles don't forget how to work.<br>* **What it looks like:** **Isometrics.** You push against a wall or your other hand, but the arm **does not move**.<br>* **Goal:** Keep the rotator cuff "awake" without tugging on the healing ligaments.<br><br>### Phase 2: The "Range" (Weeks 2 – 6)<br>You start moving the arm, but you aren't adding weight yet.<br>* **What it looks like:** **Active-Assisted Motion.** Using a broomstick or your other arm to help the injured arm move through "safe zones."<br>* **Goal:** Get your mobility back without the muscles having to fight gravity or resistance.<br><br>### Phase 3: The "Resistance" (Weeks 6 – 12)<br>This is the official start of **strengthening**.<br>* **What it looks like:** **Resistance Bands.** You’ll start with the lightest band (usually yellow) and do "internal and external rotations"—basically, movements that look like a swinging gate.<br>* **Goal:** Specifically target the 4 rotator cuff muscles that act as the "suction cup" for your joint.<br><br>### Phase 4: The "Load" (3 Months+)	Based on standard rehabilitation guidelines for a first-time anterior shoulder dislocation, you can typically begin gentle, formal strengthening exercises around **2 to 4 weeks** after the injury .<br><br>However, it is important to understand that this is not a return to weightlifting. The type of "strengthening" safe for this early phase is very specific and controlled. Your rehabilitation will follow a gradual progression from protection to full strength, and starting the wrong kind of exercises too early can put your shoulder at risk of re-dislocation.<br><br>### The Typical Timeline for Strengthening<br><br>Rehabilitation after a shoulder dislocation is managed in phases. Here is the standard timeline for introducing strengthening exercises:<br><br>**Phase 1: Weeks 0-2 (Protection and Gentle Motion)**<br>- **Focus:** This phase is for pain and swelling management. You should focus on gentle range-of-motion exercises like pendulums and active-assisted movements, where your good arm helps move the injured one .<br>- **Strengthening:** No strengthening should be performed in this phase. The tissues need time to begin the initial healing process without stress.<br><br>**Phase 2: Weeks 2-6 (Introduction of Gentle Strengthening)**<br>- **Focus:** This is when strengthening can safely begin. The key is to start with **isometric exercises**, which are muscle contractions without any joint movement .<br>- **Type of Exercise:**<br>- **Isometric External Rotation:** Stand next to a wall with your injured elbow bent to 90 degrees and tucked at your side. Gently push the back of your wrist outward against the wall as if trying to rotate your forearm away from your body. **Hold for 5-10 seconds. Repeat 10 times** .

Still avoid:

* Resistance bands
* Weights
* Overhead movements
* Outward rotation under load

---

3. Strengthening phase (~4–8+ weeks)

If your shoulder is stable and improving:

* Resistance band exercises (light → moderate)
* Rotator cuff strengthening
* Controlled range-of-motion strengthening

This is where real rebuilding of stability happens.

---

4. Advanced strengthening (8–12+ weeks)

* Heavier resistance
* Functional movements
* Sport-specific training (if needed)

Important for your case (age 20)

* Your shoulder is more likely to re-dislocate if strengthening is started too aggressively too early
* But it also responds very well to **structured, gradual rehab**
* Timing matters more than intensity

Simple rule to follow

You can start strengthening when:

* Pain is mild and improving
* Basic movements feel stable
* A physiotherapist or doctor clears you

This is when you can finally head back to the "real" gym.
* **What it looks like:** Light dumbbells and cables.
* **The Rule:** You start with high reps (15–20) and very low weight. You focus on **stability**, not "maxing out."

Why can't I start sooner?
In your 20s, the **bone** might feel fine by week 3, but the **Labrum** (the cartilage ring) takes about **6 to 8 weeks** to physically re-attach to the bone. If you start pulling on it with heavy weights in week 4, you're just peeling it right back off.

The "Green Light" Checklist
You are ready to start light strengthening when:
1. You have **zero pain** during normal daily activities (like eating or typing).
2. You have **full range of motion** (you can reach up and out without a "catch").
3. A **Physical Therapist** has tested your stability and told you it's safe.

The Golden Rule for 20-Year-Olds
> **Do not skip the "boring" band work to get to the "fun" bench press.** If you build big chest and shoulder muscles (the "movers") without strengthening the tiny rotator cuff muscles (the "stabilizers"), you're just building a bigger engine for a car with broken steering. It *will* crash again.

The Expert Guide Question: Are you planning on working with a Physical Therapist soon, or are you trying to map out a plan to do this on your own?

- **Isometric Internal Rotation:** Stand facing a door frame with the same elbow position. Push the palm of your hand inward against the door frame.
- **Progression:** As you gain comfort and your physiotherapist clears you (usually around **week 4-6**), you will progress to using light **resistance bands** for similar rotational movements .

Phase 3: Weeks 6-12 (Progressive Strengthening)
- **Focus:** This phase aims to restore full, pain-free range of motion and build functional strength. You can now move beyond isometrics into exercises that move the joint through a larger range against resistance .
- **Type of Exercise:** You will likely continue with resistance bands and may start light dumbbell exercises for the rotator cuff and shoulder blade (scapular) muscles. The emphasis remains on proper form and control, not on lifting heavy weight.

Phase 4: 12 Weeks and Beyond (Advanced Strengthening and Return to Activity)
- **Focus:** The goal now is to return to sport, work, or full daily activities. Strengthening exercises become more demanding and sport-specific . This phase includes proprioceptive (joint awareness) training and exercises in more challenging positions to ensure the shoulder is stable.
- **Full Recovery:** It's important to know that while much progress is made in the first 12 weeks, it can take **6 months or longer** to feel fully confident and return to all activities, especially contact or overhead sports .

Critical Precautions While Strengthening

- **Avoid the "Danger Position":** For at least the first **6 weeks**, you must strictly avoid combining **abduction** (lifting your arm out to the side) with **external rotation** (rotating your hand backward) . This is the position that most commonly causes an anterior dislocation to recur.
- **Pain as a Guide:** It is normal for your shoulder to ache during and after exercises. However, this ache should settle within **30 minutes** of stopping. If it doesn't, or if you experience sharp pain, you are doing too much and should take a step back .
- **Follow Professional Guidance:** The timelines above are general guidelines. Progressing from one phase to the next should always be done under the guidance of a doctor or physiotherapist, who will clear you based on your specific strength and stability, not just the date on the calendar .

	## Bottom line<br><br>* **No strengthening in the first 1–2 weeks**<br>* **Very light muscle activation after ~2–4 weeks (if stable)**<br>* **True strengthening usually starts around 4–6 weeks or later**<br><br>---<br><br>If you want, I can give you a **clear week-by-week plan from now until full recovery**, including exactly when to add each type of exercise.		
51	You can usually tell your shoulder is getting stronger and more stable by a combination of **how it feels, what you can do, and how it responds afterward**. It's not one single sign—it's a gradual pattern of improvement.<br><br>---<br><br>## 1. It feels more "secure" in daily life<br><br>Early on, your shoulder may feel uncertain or guarded. Improvement looks like:<br><br>* Less feeling of "slipping" or hesitation<br>* More confidence when moving the arm<br>* Less need to consciously protect it all the time<br>* You stop thinking about it constantly during normal activities<br><br>A key sign: **you start trusting the arm again**<br><br>---<br><br>## 2. Pain decreases and becomes less sensitive<br><br>You may notice:<br><br>* Movements that used to feel uncomfortable now feel easier<br>* Less soreness after light activity<br>* Reduced "sharp awareness" of the joint<br><br>Important: some mild discomfort can still be normal during rehab, but it should be improving overall.<br><br>---<br><br>## 3. You can move more without discomfort<br><br>Progress looks like:	Since you're 20, you're probably used to "strength" meaning you can lift more weight or see more muscle definition. However, after a dislocation, "shoulder strength" looks different. It's less about how much you can bench and more about how much you **trust** the arm.<br><br>Here are the four key ways to tell your shoulder is actually getting stronger and more secure:<br><br>### 1. The "Automatic" Feel (Decreased Guarding)<br>In the beginning, you have to think about every move. As you get stronger, the **"brain-to-shoulder"** connection repairs itself.<br>* **The Sign:** You find yourself reaching for a glass or opening a door with your injured arm without "pre-planning" the move.<br>* **Why it matters:** This means your rotator cuff is firing automatically again to stabilize the joint, rather than relying on you consciously protecting it.<br><br>### 2. The "Apprehension" Test<br>"Apprehension" is that nauseating, scary feeling that the arm is going to pop out when you move it a certain way.<br>* **The Sign:** You can move your arm into the "caution zones" (like reaching slightly to the side) without that "Oh no!" feeling in your gut.<br>* **Why it matters:** Security is as much mental as it is physical. When the muscles are strong, your nervous system stops sending "danger" signals because it feels the joint is physically supported.<br><br>### 3. Smoothness vs. "Ratcheting"<br>When a shoulder is weak, it moves in little "jumps" or shakes (this is called ratcheting).<br>* **The Sign:** When you slowly lower your arm from a raised position, it moves in one smooth, fluid motion.<br>* **Why it matters:** Smooth movement is a direct sign that the four muscles of the rotator cuff are working in perfect harmony. If it's "shaky," the muscles are still struggling to pass the weight of the arm back and forth to each other.	That is an excellent question because after a dislocation, "feeling secure" is just as important as measurable strength. Many patients mistake the absence of pain for a stable joint, but they are not the same thing.<br><br>For a 20-year-old recovering from a first-time anterior dislocation, there are specific, tangible signs you can look for to track both **Objective Strength** and **Subjective Security**. Here is how to know you are on the right track.<br><br>### Part 1: Signs Your Shoulder Is Objectively Getting Stronger<br><br>You should notice these changes gradually between Weeks 4 and 12.<br><br>**1. The "Shrug" Test Disappears**<br>- **Early Recovery:** When you try to lift your arm just a few inches, your shoulder blade hikes up to your ear immediately. Your neck looks tense.<br>- **Getting Stronger:** You can lift your arm to about 45-60 degrees (waist height) while keeping your shoulder blade **down and relaxed**. The motion comes from the arm bone, not the neck. This is a sign the rotator cuff is starting to do its job instead of the big neck muscles compensating.<br><br>**2. Improved Endurance with Daily Tasks**<br>- **Early Recovery:** Holding a hairdryer or a book with the elbow slightly away from the body causes a deep, burning ache within 30 seconds.<br>- **Getting Stronger:** You can write, type, or eat a full meal without having to rest the arm in your lap or shake it out due to fatigue. You might still feel a dull ache, but it takes much longer to appear.<br><br>**3. The "Towel Hold" Becomes Automatic**<br>- **Early Recovery:** When you do the Isometric External Rotation (pushing outward against the wall), you have to

* Easier lifting of the arm (still within safe limits)
* Better range of motion without tightness
* Less fatigue during simple tasks like writing or using your phone

---

4. Exercises feel easier and more controlled

During rehab:

* Pendulum or light exercises feel smoother
* You can repeat movements with less effort
* You feel more control rather than “awkwardness”

---

5. After-exercise recovery improves

A strong sign of improvement:

* Less soreness after exercises
* Faster recovery after activity
* No increase in pain later in the day

---

6. Fewer “warning sensations”

At the beginning you may feel:

* Apprehension in certain positions
* Muscle guarding or stiffness

As you improve:

* These warning feelings become less frequent or disappear

---

7. Better shoulder stability under light load

Later in rehab, you may notice:

* You can carry light items without discomfort
* The shoulder feels stable even when your arm is slightly away from your body (within safe limits)

---

4. End-of-Day Endurance
Early on, your shoulder might feel okay in the morning but like a "dead weight" by 4:00 PM.
* **The Sign:** You can get through a full day of school or work—carrying a backpack (on the good shoulder!), typing, and walking through crowds—without that heavy, throbbing ache at the end of the day.
* **Why it matters:** Strength is about endurance. A secure shoulder can handle the "micro-stress" of a long day without the stabilizers giving up and letting the joint sag.

How to "Test" Your Progress (Safely)
Don't test your strength by lifting something heavy. Use these **"Stability Checks"** instead:

The Test	What "Stronger" Feels Like
**The Wall Press**	Pressing your palm into a wall (arm at your side). It feels "solid" rather than "hollow" or shaky.
**The "Empty Hand" Reach**	Reaching for a shelf at eye level. You can do it without your shoulder "hiking" up toward your ear.
**The Sleep Test**	You can roll over in bed without a sudden "stab" of pain waking you up.

The "False Positive" Warning
Be careful! Around week 3 or 4, the **pain** often goes away completely, but the **strength** isn't back yet. This is the most dangerous time. You might feel "secure" because it doesn't hurt, but the ligaments are still weak. Always trust your **Physical Therapist's** objective tests (where they push against your arm) more than your own feeling of "being fine."

The Expert Guide Question: Do you still feel that "apprehension" or "fear" when you take your arm out of the sling to shower, or is that starting to fade away?

consciously think about keeping that rolled-up towel between your elbow and ribs. If you forget, the elbow flies out to the side.
- **Getting Stronger:** The elbow stays pinned to your side naturally, without you having to squeeze it there. This means the muscles are learning the correct movement pattern.

Part 2: Signs Your Shoulder Feels More Secure (The "Apprehension" Meter)

This is a psychological and neurological measure. A stable shoulder feels "quiet" in the brain.

1. The "Quick Move" Reflex Improves
- **Early Recovery:** If someone tosses you a set of keys or you reach to stop a door from closing, you feel a **flash of panic** or a sharp, protective spasm in the front of the shoulder—even if the arm doesn't actually move much.
- **Getting More Secure:** You find yourself reaching for things (like a light switch or a coffee cup) **without thinking about it first.** The motion is still slow and controlled, but the "pre-movement anxiety" is gone.

2. Sleeping Position Tolerance
- **Early Recovery:** You can **only** sleep on your back or the opposite side. If you even start to roll toward the injured side, you wake up immediately with a warning ache.
- **Getting More Secure:** You can sleep on the **injured side** with a pillow hugged to your chest for support, without waking up in pain or feeling like the joint shifted in your sleep.

3. The "Arm Behind Back" Milestone
This is a specific movement test for anterior stability.
- **Danger Zone:** Reaching behind your back (like to tuck in a shirt or reach a back pocket) puts the shoulder in external rotation and extension. This is the *exact* vulnerable position.
- **Positive Sign of Security:** You can slide your hand into your **back pocket** or touch the small of your back **without** holding your breath or feeling a "clunk" in the front of the joint. You might not be able to reach as high as the other arm yet, but the movement feels smooth and safe, not scary.

Part 3: Warning Signs That Suggest Instability (Not Weakness)

Even if you are getting stronger in the exercises, these specific sensations indicate the **ligament** may be too loose, regardless of muscle strength:

	## What is NOT a reliable sign<br><br>* Feeling strong in one movement doesn’t mean full stability yet<br>* No pain does not automatically mean full healing<br>* Confidence returns gradually, not all at once<br><br>---<br><br>## Simple summary<br><br>Your shoulder is getting stronger when:<br><br>* It feels more stable in daily life<br>* Movement becomes easier and smoother<br>* Pain and soreness decrease over time<br>* Exercises feel controlled, not shaky or alarming<br><br>---<br><br>## Important reminder for your case (age 20)<br><br>Even if it feels better quickly, the deeper stabilizing tissues take **weeks to months** to fully recover. Feeling “normal” comes before full structural healing.<br><br>---<br><br>## Bottom line<br><br>You can tell your shoulder is improving when it feels **steadier, less painful, more controlled, and more reliable in everyday movement over time**.<br><br>---<br><br>If you want, I can help you track your recovery with a simple checklist so you can clearly see week-by-week progress and know if you’re on track.		- **The "Clunk" Sensation:** When lowering your arm from an outstretched position, you feel a distinct **thud** or **shift** in the front of the shoulder. This is the ball of the shoulder sliding off the rim of the socket (subluxation). *This is not normal and should be reported to your doctor.*<br>- **Dead Arm After Reaching:** You reach up to grab something from a shelf. You don't feel pain, but the arm suddenly feels **weak, heavy, and numb** for a few seconds. This is the nerve/muscle reflex shutting down because the joint moved too much.<br><br>**Summary for Your Age Group**<br>At 20 years old, the shoulder may feel "strong" in a gym setting (lifting weights straight forward) long before it feels "secure" in a dynamic setting (turning a steering wheel sharply). **Trust the feeling of security over the feeling of strength.** When you can do the exercises without the neck shrugging **and** you can reach for a light switch without a second thought, you are in a very good place.
52	Yes—**it’s very normal to feel shaky, weak, or unsure when using your shoulder during exercises after a dislocation**, especially in the early stages of recovery.<br><br>### Why this happens<br><br>After a shoulder dislocation:<br><br>* The **stabilizing ligaments and labrum are injured**, so the joint is temporarily less stable	It is **100% normal** to feel like a "shaking leaf" when you first start exercising a dislocated shoulder. At 20, you’re used to having total control over your body, so this loss of stability can feel really unnerving—but it’s actually a predictable part of the healing process.<br><br>Here is why that "shaky and unsure" feeling happens:<br><br>### 1. The "Neurological Disconnect"<br>When your shoulder popped out, your brain basically "cut the power" to the surrounding muscles to prevent further damage. Now that	Yes, it is absolutely normal and expected to feel shaky, weak, or unsure when using your shoulder during these early exercises. In fact, this sensation is so common after a dislocation that it has a clinical name: **Apprehension and Neuromuscular Inhibition.**<br><br>For a 20-year-old who is likely used to feeling strong and coordinated, this shakiness can be unsettling. However, it is a predictable and temporary part of the healing process. Here is exactly why it happens and what it means.<br><br>### Reason 1: Neuromuscular Inhibition (The "Off Switch")

* The **rotator cuff muscles are weak and “inhibited”** (they don’t activate properly right away)
* Your nervous system becomes **protective and cautious**, which affects coordination
* You haven’t yet rebuilt the fine control that keeps the shoulder centered

So shakiness is often a mix of **weakness + lack of coordination + natural protection response**.

What is normal

You may notice:

* Mild trembling during gentle exercises
* Feeling like the shoulder is “not fully controlled”
* Extra effort needed for simple movements
* Fatigue or shaking after a few repetitions

This is especially common with:

* Pendulum exercises (early on)
* Scapular control exercises
* First attempts at light activation

What is NOT normal

Be more cautious if you feel:

* A sensation that the shoulder might slip out
* Sharp pain along with the shakiness
* Increasing instability with each repetition
* Sudden loss of control or “giving way”

How it should improve

As you recover:

* Shakiness should **gradually decrease over weeks**
* Movements should feel **more controlled and smoother**
* Confidence in the shoulder should slowly return
* Fatigue will still happen, but control improves

you’re starting to move again, the neural pathways between your brain and your muscles are like a bad Wi-Fi connection.
* **The Shake:** That trembling is your brain trying to figure out which muscle fibers to fire and in what order. It’s "re-learning" how to coordinate the joint.

2. Muscle Fatigue (The "Tired Stabilizer")
The tiny muscles of your **rotator cuff** are currently out of shape because they’ve been protected in a sling.
* **The Shake:** Just like your legs shake at the end of a long set of heavy squats, your shoulder stabilizers shake because they are exhausted after just a few seconds of work. They are small muscles, and right now, they have very little endurance.

3. "Apprehension" (The Mental Brake)
Your nervous system has a "memory" of the injury. When you move your arm, your brain is "braking" and "accelerating" at the same time.
* **The Feeling:** You feel "unsure" because your brain is literally hesitating, waiting to see if the bone is going to slip again. This is a survival mechanism called **Apprehension.**

How to Handle the Shakes

* **Reduce the Intensity:** If you are shaking violently, you are pushing too hard. Back off the effort by **50%**. Even a tiny, steady contraction is better for your brain than a big, shaky one.
* **Shorten the Hold:** If the shake starts at 5 seconds, only hold the exercise for 3 seconds. Quality and smoothness are your goals, not "toughing it out."
* **Use Your "Good" Side as a Guide:** Do the exercise with your healthy arm first. This gives your brain a "blueprint" of what a smooth, stable movement should feel like, which often reduces the shaking on the injured side.
* **Focus on Breath:** When we feel unsure, we tend to hold our breath. This tenses the neck and actually makes the shaking worse. **Exhale** during the hardest part of the movement to help stay calm.

When Should You Worry?
While **shaking** is normal, **shifting** is not.
* **Normal:** "My arm feels weak and trembles like I'm tired."
* **Not Normal:** "I feel the bone 'thudding' or 'clicking' in and out of place while it shakes."

When your shoulder dislocated, the trauma did not just stretch the ligaments; it temporarily scrambled the electrical signals between your brain and the deep muscles of the rotator cuff.
- **What You Feel:** When you try to do an Isometric Press (pushing outward against the wall), your arm feels weak, wobbly, or trembles like a leaf. You might push with 100% effort but feel like you are only giving 10% output.
- **Why It's Normal:** The brain is protecting the joint by refusing to fire those muscles at full power. The shakiness is a sign that you are asking a "sleeping" muscle to wake up. This is **good stress**. As you repeat the exercise over the next 2-3 weeks, the signals become clearer and the shakiness will smooth out.

Reason 2: Apprehension (The Brain's Memory)
Even if the muscle is capable of working, your subconscious brain remembers the trauma of the dislocation.
- **What You Feel:** You start to move the arm away from your body or rotate it slightly, and you get a **flash of anxiety or hesitation** mid-rep. It feels like you are holding your breath without realizing it.
- **Why It's Normal:** This is a protective reflex. The brain is anticipating the position of danger (the "throwing" position) and is slamming on the brakes. This uncertainty is your guide. **Do not push past this feeling.** If you push past the shakiness into a sharp pain, the brain will learn to be *more* protective, and the shakiness will get worse. Work *within* the range that feels shaky but safe.

Reason 3: Scapular Fatigue
You are likely using muscles around your shoulder blade (scapula) that you have never consciously used before.
- **What You Feel:** When you try to hold a Scapular Squeeze or Pendulum position, the muscles between your spine and shoulder blade burn and tremble within seconds.
- **Why It's Normal:** These postural muscles are endurance muscles, but they have been shut off by the sling and the injury. They are weak and deconditioned. The shakiness here is identical to the feeling of trying to hold a heavy grocery bag with an outstretched arm when you haven't worked out in a year.

How to Manage the Shakes During Your Workout
You do not want to stop exercising because of the shakes, but you do need to adjust *how* you exercise.

1. **Use the "Shake It Out" Rule:** When the trembling starts, stop the exercise. Let the arm hang down completely dead (like in the Pendulum position) for **10 seconds**. Shake the whole

	---<br><br>### How to handle it<br><br>* Slow down the movement<br>* Reduce range of motion if needed<br>* Do fewer repetitions but more controlled ones<br>* Stop if shakiness turns into instability or pain<br>* Focus on quality, not strength early on<br><br>---<br><br>### Bottom line<br><br>Yes—**shakiness and uncertainty are normal in early shoulder rehab** after dislocation and usually improve as the muscles and coordination recover. It's a sign your shoulder is still relearning stability, not necessarily that something is wrong.<br><br>---<br><br>If you want, I can explain how to tell the difference between **normal rehab shakiness vs signs of instability that need medical attention** in a simple way.	### The Bottom Line<br>Think of the shaking as **"re-calibration."** It's a sign that you are working exactly the muscles that need it most. As those neural pathways get stronger and the "Wi-Fi signal" improves, the shaking will disappear, and you'll start to feel that "solid" sense of security again.<br><br>**The Expert Guide Question:**<br>When you feel that shakiness, does it happen the moment you start the exercise, or does it only start after you've been holding the move for a few seconds?	arm gently to reset the nerves. Then try again with **50% less force**.<br>2. **Shorten the Lever:** If the shakiness is severe, bring the movement closer to your body. For example, instead of pushing with your hand far from the wall, slide your elbow closer to the wall or bend your elbow more. A shorter lever arm reduces the load on the weak rotator cuff.<br>3. **Exhale on Effort:** Many patients hold their breath when they feel unsure. This creates tension in the chest and neck that makes the shakiness worse. **Breathe out slowly** as you push or pull.<br><br>### When Does the Shaky Feeling Stop?<br>- **During an Exercise Session:** The shaking should stop within 30 seconds of resting.<br>- **Over the Course of Recovery:** You should notice a significant reduction in shakiness and "unsureness" by the end of **Week 3 or 4** of consistent, gentle exercise.<br><br>### Red Flag: When Shakiness Is Not Normal<br>While shakiness is expected during the exercise, you should contact your doctor or physical therapist if:<br>- The arm feels "shaky" and **dead/heavy** for hours **after** you have finished exercising.<br>- The shakiness is accompanied by a visible **shift** or **clunk** of the bone in the front of the shoulder (subluxation).<br>- The shakiness is getting **worse** week over week instead of better.<br><br>**Summary**<br>Think of the shakiness as the static on a radio station that is just slightly out of tune. Right now, the signal between your brain and shoulder is fuzzy. The exercises you are doing are the fine-tuning dial. Keep the volume (effort) low, and the signal will become clear and strong again.
53	That feeling is actually quite common after a first shoulder dislocation, even when you're doing the exercises correctly. It doesn't automatically mean something is going wrong.<br><br>### Why your shoulder still feels "like it could come out"<br><br>**1. The stabilizing structures are still healing**<br>After an anterior dislocation, the **labrum and ligaments are stretched or torn**. These are the main "static stabilizers" of the joint.<br>Even though pain improves quickly, **true mechanical stability takes weeks to months to recover**.<br><br>---	It is incredibly frustrating to be "doing everything right" and still feel like your shoulder is held together by a loose rubber band. At **20 years old**, your brain expects your body to respond instantly to training, but shoulder rehab has a very specific "lag time."<br><br>The reason you still feel that "slipping" sensation (called **instability**) or that "fear" (called **apprehension**) usually comes down to three things:<br><br>### 1. The "Structural Void"<br>Exercises strengthen **muscles**, but they don't fix **ligaments** or **cartilage** (the labrum).	This is one of the most psychologically challenging parts of recovering from a shoulder dislocation, especially for a 20-year-old. You are doing the work, you are being careful, and yet that nagging feeling of "it's not quite right" or "it might slip" persists.<br><br>The short, honest answer is: **Because the muscles get stronger much faster than the ligaments heal.** Your exercises are working, but they are working on the part of the injury that recovers *first*. The part that makes you feel "secure" takes much longer.<br><br>Here is the detailed, plain-text explanation of why you still feel this way and when that feeling should start to fade.

2. Muscles are still relearning how to protect the joint
Your shoulder relies heavily on muscles (rotator cuff + shoulder blade muscles) for stability. After injury:

* They become weak and “inhibited”
* They don’t react quickly yet
* Coordination is not fully restored

So even if they’re working, they’re not yet *efficient* at preventing that “slipping” sensation.

---

3. Your brain is in protective mode
After a dislocation, the nervous system becomes cautious. This causes:

* Heightened awareness of the shoulder
* “Guarding” sensations
* A false sense of instability in certain positions

This is a normal protective response, not necessarily real instability.

---

4. Certain positions are naturally risky early on
The shoulder is most vulnerable when the arm is:

* Out to the side
* Rotated outward
* Slightly extended backward

Even during rehab, if you accidentally approach these positions, you may feel that “it might come out” sensation.

Important distinction

There are two different feelings:

Normal early rehab feeling:

* Mild insecurity or caution
* Improves when you adjust position
* No actual slipping or sharp pain

Concerning instability:

* Repeated feeling of actual slipping or shifting

* When you dislocate, the "cup" of your shoulder socket (the labrum) often peels away. Exercises don't "glue" that back—they just train the muscles to compensate for it.
* **The Reality:** Until those tissues scar down and tighten (which takes **6–12 weeks**), your shoulder physically *is* looser than it used to be. The muscles are trying to do the job of both muscle and ligament, and they aren't strong enough to do both yet.

2. The "Proprioception" Gap
Your shoulder is full of tiny sensors that tell your brain where the bone is in space. A dislocation "blows the fuse" on these sensors.
* Even if your muscles are getting stronger, your **brain** hasn't updated its GPS. It still thinks the joint is in danger because the sensors are sending "garbage data."
* This is why you feel "unsure"—your brain is literally guessing if the bone is centered in the socket.

3. Muscle "Fatigue" Instability
Your rotator cuff muscles are tiny. They might be strong for the first 5 minutes of your exercises, but they tire out quickly.
* Once they fatigue, they "relax" their grip on the bone. If you’re doing your exercises but then going about your day, your shoulder might feel loose because the muscles are simply **spent** from the workout.

How to bridge the "Security Gap"

* **Focus on "Closed Chain" Moves:** Ask a PT about exercises where your hand is fixed against a surface (like gently pressing into a wall). This sends a "solid" signal to the brain that usually feels much more secure than "waving" the arm in the air.
* **The Sling is Your Safety Net:** If it feels loose, **wear the sling.** It’s not just for the first week; it’s for anytime your muscles feel too tired to hold the joint together.
* **Trust the Timeline:** There is a "crossover point" around **Month 3** where the muscle strength finally becomes high enough to truly take over for the loose ligaments. Before that, feeling "loose" is a normal part of the process.

When to talk to a Surgeon
If you have been doing **perfect** physical therapy for 3 months and it *still* feels like it’s going to pop out just by reaching for a glass of water, that is a sign that the structural damage (the tear) might be too large for muscles alone to fix.

The Two Layers of Shoulder Stability (The Race Analogy)

Imagine your shoulder stability is a car with two safety systems:
1. **The Seatbelt (Ligaments/Labrum):** This is the passive restraint. It's the thick, leathery tissue that holds the ball tight to the socket. When you dislocated, this seatbelt **stretched out or tore**. It is now loose.
2. **The Driver's Arms (Muscles):** This is the dynamic control. These are the rotator cuff and scapular muscles you are exercising.

Where You Are Now (Weeks 2-6):
You are strengthening the "Driver's Arms." They are getting better at gripping the wheel. **BUT**, the "Seatbelt" is still loose. It takes **6 to 12 weeks** for that torn ligament to lay down enough scar tissue to even begin to feel snug again, and it may never return to 100% of its original tightness.

Because the seatbelt is loose, every time you move to the edge of your safe range, your brain feels the ball of the shoulder slide just a tiny bit further forward than the other shoulder. The muscles catch it, but your brain registers that **micro-motion** as a warning: *"Whoa, the seatbelt didn't catch me there."*

Why This Feeling is Actually *Protective* Right Now

It is frustrating, but **this feeling of apprehension is a safety feature, not a failure of your rehab.**

- **Protecting the Repair:** If you felt 100% secure and confident right now (Week 3 or 4), you would be at **extremely high risk** of doing something stupid. You might instinctively reach out to catch a falling phone or stretch your arms overhead in the morning without thinking. That loose seatbelt wouldn't stop the dislocation.
- **The 20-Year-Old Factor:** Because you are young, your tissues are very elastic. The shoulder *can* move further in the socket than an older person's shoulder. This extra mobility (laxity) feels like instability. Your exercises are teaching your muscles to control that extra motion, but it takes **thousands of repetitions** to make that control automatic and subconscious.

How to Measure Progress Despite the Feeling

You should not expect the feeling of "could pop out" to vanish for at least **3 months**. Instead of waiting for the feeling to disappear, track these smaller, objective changes that prove your exercises are working:

	* Episodes where it feels like it almost dislocates<br>* Increasing episodes over time instead of improvement<br><br>---<br><br>### What this means for you<br><br>If you are:<br><br>* Doing gentle, guided exercises<br>* Not forcing range of motion<br>* Avoiding risky positions<br><br>☞ Then this feeling is usually part of normal recovery and should gradually reduce over time.<br><br>---<br><br>### When it should improve<br><br>* Often starts improving noticeably after a few weeks of rehab<br>* Becomes much less frequent as strength + control improve<br>* Should not progressively worsen<br><br>---<br><br>### Bottom line<br><br>That “it could come out” feeling is usually due to **healing tissues + weak coordination + nervous system protection**, not necessarily a new injury. It’s common early on and should slowly improve with proper rehab.<br><br>---<br><br>If you want, I can help you identify **which specific positions or movements are likely triggering that feeling in your case**, so you can avoid them while still recovering safely.	> **The Good News:** For most 20-year-olds, the "loose" feeling is temporary. It’s just your brain being extra cautious while the internal "glue" is still setting.<br><br>**The Expert Guide Question:**<br>Does it feel loose specifically when you reach **away** from your body, or do you feel it even when your arm is resting at your side?	- **The "Scary Range" Gets Smaller:** In Week 1, lifting your arm 10 inches felt scary. In Week 4, it might feel scary at 20 inches. The distance you can move **before** the apprehension kicks in is getting larger. That is progress.<br>- **Recovery from a "Scare":** If you move too fast and feel a "clunk" or a pinch, how long does the pain and anxiety last? In Week 1, it ruined your whole day. In Week 4, you might shake it off in 2 minutes. This means the joint is less reactive and more resilient.<br>- **Sleeping Position:** If you can now sleep on your back without a pillow under the elbow, or if you can roll slightly toward the injured side without waking up, the passive stability is improving, even if you don't feel it during the day.<br><br>### When to Be Concerned (The "Loose Screw" Sensation)<br><br>There is a difference between **Apprehension** (a brain warning) and **True Instability** (a mechanical failure).<br><br>- **Normal (Apprehension):** *"If I move my arm back there, it feels weird and I'm afraid it might pop."*<br>- **Abnormal (Instability):** *"I moved my arm back there, and it **actually slipped out of place** for a split second and then slid back in."* (This is a subluxation).<br><br>If you are experiencing actual **slipping** or **dead arm** (where the arm goes numb and heavy for a few seconds), that is a sign that the exercises alone may not be enough to compensate for the torn seatbelt. This is something you must report to your orthopedic surgeon, as it may indicate a need for surgical stabilization.<br><br>**Summary for Your Peace of Mind**<br>Feeling like it *could* come out while doing your exercises correctly is **expected and normal** at this stage. You are not doing anything wrong. Your brain is just smarter than your muscles right now. As long as the joint stays **in** the socket and the shakiness improves over the next 4-6 weeks, you are on exactly the right track.
54	For a **first-time anterior shoulder dislocation**, feeling “strong and stable again” usually takes **weeks to months**, not days. The exact timeline varies, but there are fairly typical stages.<br><br>---<br><br>## 🕒 Typical recovery timeline<br><br>### **0–2 weeks**	At **20 years old**, your timeline is a bit of a paradox: you’ll likely feel "back to normal" in your daily life relatively quickly, but "athletic" or "total" stability takes much longer than most people realize.<br><br>Here is the realistic roadmap for when that "rock-solid" feeling actually returns:<br><br>### 1. The "Daily Life" Milestone (**4–6 Weeks**)	Based on your situation as a 20-year-old recovering from a first-time anterior dislocation without fracture, you can expect a recovery journey that happens in distinct phases. While you will likely feel much better within a few months, achieving a sense of full strength and reliable stability is a longer process that typically extends to **6 months or more**.<br><br>The timeline below outlines the general progression from initial healing to returning to full activity. It's important to remember that these are guidelines, and your specific timeline should

* Main goal: protect the joint and reduce pain
* Shoulder feels weak, stiff, and “uncertain”
* Exercises are very gentle (movement only, no strengthening yet)

☞ At this stage, it’s normal that it does **not feel stable**

2–6 weeks

* Early strengthening begins (light activation, basic control)
* Range of motion improves gradually
* You may still feel:

  * Weakness
  * Shakiness
  * Occasional “could slip” sensation in certain positions

☞ Stability is improving, but still fragile

6–12 weeks

* Clear improvement in **muscle control and coordination**
* Strengthening becomes more structured
* Daily activities feel much more normal
* Shoulder starts to feel **more reliable**

☞ Many people first feel “noticeably stable” here

3–6 months

* Much stronger rotator cuff and shoulder blade control
* Return to sports or heavier activity often happens here (depending on rehab and doctor’s advice)
* Shoulder feels **close to normal for most daily use**

6+ months

* Full functional stability for most people
* Higher-risk sports still require careful progression

Around this mark, the initial swelling is gone and the "wet glue" phase of the labrum healing has finished.
* **How it feels:** You can usually reach for a glass of water, type comfortably, and get through a school day without the constant "heavy" ache.
* **The Trap:** This is the most dangerous time. You *feel* fine, so you might try to lift something heavy or throw a ball, but the internal structures are still only at about **50% strength**.

2. The "Trust" Milestone (**3 Months**)
This is usually when you’ve had about 6–8 weeks of consistent strengthening with resistance bands.
* **How it feels:** The "shakiness" during exercises disappears. You start to trust your arm when you’re in a crowd or reaching for a seatbelt.
* **The Progress:** This is typically when a Physical Therapist will clear you to return to the gym for light, controlled weightlifting (machines rather than free weights).

3. The "Full Recovery" Milestone (**6–9 Months**)
True stability—where you can play a pickup game of basketball, go swimming, or do a pull-up without a second thought—takes a minimum of half a year.
* **How it feels:** Your brain finally "unlearns" the fear of the injury. The rotator cuff has reached enough **hypertrophy** (size and strength) to act as a permanent, active brace for the joint.

Factors That Speed Up (or Slow Down) the Clock

**Factor**	**Effect on Timeline**
**Consistency**	Doing your "boring" PT exercises daily can shave weeks off the "shaky" phase.
**First-Time vs. Repeat**	If this is your **first** dislocation, you'll feel stable faster. If it's a repeat, it takes longer because the tissue is more stretched.
**Sleep/Nutrition**	At 20, your growth hormone is high. Getting 8 hours of sleep and eating enough protein actually speeds up the structural "knitting" of the labrum.
**"The Test"**	If you "test" it too early and have a "subluxation" (a partial slip), you effectively **reset the clock** back to Day 1.

The "90% Rule"
In the world of sports medicine, we often say that the last 10% of stability takes as long to gain as the first 90%. You might feel **90%

always be confirmed by your physiotherapist or doctor based on your individual progress rather than the calendar alone.

📅 Typical Recovery Timeline

- **Weeks 0-2: Protection and Pain Relief**
   The main goal is to protect the joint and allow initial healing. You'll wear a sling to rest the shoulder, except when doing gentle exercises to prevent your elbow, wrist, and hand from getting stiff. Pain and swelling will be managed with ice and possibly medication .

- **Weeks 2-6: Restoring Gentle Movement**
   With your therapist's guidance, you'll start exercises to gradually regain your shoulder's range of motion. The focus is on achieving full, pain-free movement. You'll also begin very gentle strengthening exercises for the rotator cuff and shoulder blade (scapular) muscles, which are crucial for stability .

- **Weeks 6-12: Building Foundational Strength**
   This phase focuses on a progressive strengthening program. You'll use resistance bands and light weights to improve the strength and endurance of the muscles that hold the shoulder in place. The aim is to restore functional strength for daily activities without pain .

- **Months 3-6: Advanced Strengthening and Control**
   Rehabilitation becomes more demanding. You'll work on advanced strengthening, and your physiotherapist will introduce exercises to improve **proprioception** (your joint's sense of position) and neuromuscular control. This helps your brain and shoulder muscles coordinate better to react to sudden movements .

- **6 Months and Beyond: Return to Sport and Full Function**
   A full return to sport or high-demand physical work is often expected around this time. The decision to clear you for full activity is based on meeting specific functional criteria, including having **symmetric range of motion and strength** equal to your uninjured shoulder, and being free of pain and apprehension (the fear that it might pop out) . For contact or overhead sports, it's wise to phase your return gradually in a training environment before competing .

🧑‍⚕️ A Note on Your Age and Recovery

As a patient under 30, you are in an age group that statistically has a higher risk of re-dislocation. For this reason, your doctor

	---<br><br>## ⚠️ Important for your age (20)<br><br>At your age:<br><br>* Healing is usually good with proper rehab<br>* But recurrence risk is higher if stability is not fully rebuilt<br>* So **feeling normal ≠ fully safe for high-risk activity yet**<br><br>---<br><br>## 🧠 Key idea<br><br>Even if pain improves quickly, the shoulder becomes truly stable when:<br><br>* Rotator cuff strength returns<br>* Shoulder blade control is solid<br>* Joint structures heal and adapt over time<br><br>This takes **months**, not weeks.<br><br>---<br><br>## ✔ What “getting better” feels like<br><br>You’ll notice:<br><br>* Less fear or hesitation in movement<br>* Fewer “slipping” sensations<br>* More confidence using the arm<br>* Better control during exercises<br><br>---<br><br>## Bottom line<br><br>Most people feel:<br><br>* **some improvement in stability in 4–6 weeks**<br>* **much stronger and more secure by 3 months**<br>* **near full functional stability by 3–6 months**<br><br>---	stable** by Month 3, but that final 10%—the part that prevents it from popping out during a fall or a sudden movement—requires you to stay committed to your exercises even after the pain is 100% gone.<br><br>### **The Bottom Line**<br>Expect to feel **"safe"** by Month 2, **"strong"** by Month 4, and **"bulletproof"** by Month 6 to 9.<br><br>**The Expert Guide Question:**<br>Are you aiming to get back to a specific "high-demand" activity, like a certain sport or a job that involves lifting, or are you just focused on getting through your daily routine pain-free?	and physiotherapist will be especially thorough in ensuring your shoulder is truly stable before clearing you for sports . The process of fully regaining confidence in your shoulder is both physical and psychological, and it's a key factor that healthcare providers consider during your recovery . Continue to follow your rehabilitation plan carefully and maintain open communication with your healthcare team about any feelings of instability or pain you experience along the way.

<table>
<tr><td></td><td>If you want, I can tell you **what signs mean your shoulder is NOT healing well or might be unstable again**, so you know when to seek follow-up care.</td><td></td><td></td></tr>
<tr><td>55</td><td>Yes—**doing the wrong exercises too early, or doing the right exercises incorrectly, can make your shoulder feel worse and in some cases increase the risk of another dislocation.** But it’s not something that happens from normal gentle rehab when done properly. --- ## When exercises can make things worse ### 1. Using the shoulder in risky positions too early The most dangerous positions after an anterior dislocation are: * Arm **out to the side** * Arm **rotated outward** * Arm slightly **behind your body** If you load or stretch the shoulder in these positions early on, it can: * Irritate healing tissues * Increase instability feeling * In rare cases, trigger another dislocation --- ### 2. Adding resistance too soon Starting: * Weights * Resistance bands * Push-ups or planks before your shoulder is ready can: * Overload weak stabilizing muscles * Stress healing ligaments * Increase pain and “loose” feeling afterward --- ### 3. Forcing range of motion Trying to “push through stiffness” can: * Irritate the joint</td><td>Short answer: **Yes, absolutely.** Doing the wrong exercises—or even the right exercises with the wrong form—can be a "fast track" back to the emergency room. At 20, your muscles are strong, but the **structural "anchors"** (the labrum and ligaments) are currently compromised. If you choose an exercise that uses your arm as a long lever against those anchors, the muscles can actually pull the bone right out of the socket. Here are the ways the wrong moves make things worse: ### 1. The "Pry Bar" Effect Some exercises put your arm in the **"High-Five" position** (elbow out to the side and hand rotated up/back). * **The Risk:** This position turns your humerus (upper arm bone) into a pry bar. Even a light weight in this position can "lever" the ball out of the front of the socket. * **Exercises to Avoid:** Behind-the-neck lat pulldowns, deep chest flies, or overhead presses if your elbow drifts behind your ear. ### 2. Bicep Tension Your bicep muscle is physically attached to the **labrum** (the cartilage ring in your shoulder). * **The Risk:** If you do heavy bicep curls or "pulling" movements too early, the bicep tendon can pull on the torn labrum like a loose thread on a sweater, making the tear larger or preventing it from ever healing. ### 3. "Shear" Force vs. "Compression" Force * **Good Exercises** create **Compression:** They push the ball into the socket (like gently pressing your hand against a wall). * **Bad Exercises** create **Shear:** They try to slide the ball *across* the socket (like doing a lateral raise with a dumbbell). * **The Result:** Shear force is what causes the "slipping" feeling and can lead to a re-dislocation. --- ### **The "Danger List" of Exercises (Wait for PT Clearance)** | Exercise	Why it's Dangerous Right Now
Bench Press	The "bottom" of the move over-stretches the front of the shoulder.

Dips	Puts extreme pressure on the front of the joint capsule. |</td><td>Yes, absolutely. Doing the wrong exercises—or even doing the *right* exercises with the wrong form—can definitely make your shoulder feel worse and significantly increase the risk of it coming out again. This is particularly true for a 20-year-old patient where the soft tissues are still loose and healing.
	
	You need to be very clear on the difference between **Safe Healing Discomfort** and **Dangerous Instability Stress**. Here is exactly how the wrong exercises cause problems.
	
	### Mechanism 1: The "Apprehension Position" (Direct Risk of Re-Dislocation)
	The most common way the wrong exercise causes a second dislocation is by placing the arm in the **vulnerable position** before the ligaments are ready.
	
	- **The Dangerous Position:** Abduction (arm lifted out to the side) combined with External Rotation (hand turned backward, like you are throwing a ball or reaching to the back seat of a car).
	- **The Wrong Exercises:**
	   - **Lateral Raises:** Lifting a weight out to the side with a straight arm. Even with 1-2 lbs, this puts the ball of the shoulder right on the torn front edge of the socket.
	   - **Chest Flys:** Opening the arms wide as if hugging a large tree.
	   - **Behind-the-Neck Presses or Pulldowns:** This forces the shoulder into extreme external rotation.
	   - **"Empty Can" Exercises:** Lifting the arm out to the side with the thumb pointed down. This is a classic rotator cuff exercise that is **contraindicated** early on because it pinches and stresses the front capsule.
	- **Outcome:** Doing these exercises before 6-8 weeks (and before being cleared by a PT) can cause the ball to lever against the loose labrum and slip right back out of the socket.
	
	### Mechanism 2: Muscle Substitution (Making It Feel Worse and Less Stable)
	Even if the exercise doesn't pop the shoulder out immediately, doing the wrong movement pattern trains the shoulder to be **unstable**.
	
	- **The Problem:** When the rotator cuff is weak, your body cheats by using the **Deltoid** (the big cap muscle on the outside of the shoulder) and the **Upper Trapezius** (the neck muscle) to lift the arm.</td></tr>

</table>

* Increase inflammation
* Make the shoulder feel more unstable temporarily

---

4. Poor technique (even with simple exercises)

Even basic movements can be problematic if:

* The shoulder is shrugged up instead of relaxed
* The arm is not supported and hangs with strain
* Movements are fast or uncontrolled

What usually does NOT cause harm

* Gentle pendulum exercises
* Slow, pain-free movement close to your body
* Light daily activity (eating, writing, phone use)
* Properly guided physiotherapy

Signs your exercises may be too much

Watch for:

* Increasing pain after exercise (not just during)
* More “slipping” or instability feelings
* Sharp pain in certain positions
* Shoulder feeling worse the next day instead of better

Important reassurance

A true re-dislocation from exercises alone is **uncommon** if:

* You avoid risky positions
* You progress gradually
* You stay within pain limits

Most problems come from **doing too much too early**, not from basic rehab movements.

Bottom line

| **Pull-ups** | The "hanging" weight creates traction that can pull the joint apart. |
| **Bicep Curls** | Directly tugs on the healing labrum. |

Signs You Are Doing the "Wrong" Move
If you feel any of these, **stop immediately**. You aren't "pushing through"—you are damaging the joint:
* **The "Zing":** A sharp, electric-like pain.
* **The "Clunk":** Feeling a physical shift or a "speed bump" inside the joint.
* **The "Apprehension":** That sudden, nauseating fear that the arm is "vulnerable."

The Bottom Line
In your 20s, a second dislocation is much easier to trigger than the first one. Every time it comes out, the "socket" gets shallower and the "anchors" get weaker. It is much better to do "boring" movements that feel too easy for a few weeks than to accidentally "pry" your shoulder out with a gym move you weren't ready for.

The Expert Guide Question:
Have you been looking at any specific gym routines or "shoulder stability" videos online that you were thinking about trying?

- **The Wrong Form:** You try to lift your arm, and your shoulder **shrugs up to your ear**.
- **The Consequence:** The Deltoid muscle pulls the ball of the shoulder **UP and FORWARD** in the socket. Since the front of the socket (where you tore the labrum) is the weak spot, pulling the ball forward pushes it directly into the danger zone. You are literally strengthening a movement pattern that encourages the shoulder to dislocate.
- **The Feeling:** If you do exercises with a shrugged shoulder, the shoulder will feel **pinched**, **achy**, and **looser** the next day. You might get a "catching" sensation in the front of the joint with overhead reaching.

Mechanism 3: Too Much, Too Soon (Causing Inflammation and Weakness)
In the early weeks (2-6), the joint is still chemically inflamed from the trauma. The soft tissue is like wet, fragile paper.
- **The Wrong Approach:** Doing too many sets of Isometrics or pushing into pain.
- **The Consequence:** You cause **reactive synovitis** (swelling inside the joint). When the joint swells, the muscles around it shut down (inhibition). So instead of getting stronger, the shoulder actually becomes **weaker** and **more painful**.
- **The Feeling:** The shoulder feels hot at night, throbs when you are resting, and feels stiffer in the morning than it did the day before. This is a clear sign you have done the *wrong dosage* of exercise.

How to Know If an Exercise Is "Wrong" for You Right Now
Use this checklist before trying any new movement you see online or remember from the gym:

1. **The "Front Pinch" Test:** Do you feel a sharp, specific pinch in the **front** of the shoulder joint (the crease where the arm meets the chest)? If yes, **Stop.**
2. **The "Shrug" Test:** Look in a mirror. Does your shoulder lift toward your ear before your arm even starts moving? If yes, **Stop.** You are using the wrong muscles.
3. **The "Apprehension" Test:** Does the thought of doing the movement make you hold your breath or feel a wave of anxiety? If yes, **Do Not Do It.** Your brain is sensing instability that you cannot consciously feel.

Summary
Stick strictly to the **Pendulums, Isometric Presses (elbow at side), and Scapular Squeezes** until a physical therapist physically places their hands on you and says, "Okay, now we

	Yes—**incorrect or too aggressive exercises can worsen symptoms and increase instability risk**, but **proper, gradual rehab is safe and actually reduces the chance of your shoulder coming out again**.<br><br>---<br><br>If you want, I can help you identify **exactly which exercises are safe vs unsafe for your current stage**, so you can avoid accidental mistakes.		are going to try X." A single set of "wrong" exercises can undo three weeks of careful healing.
56	Yes—**strengthening exercises are one of the main ways your shoulder regains confidence after a dislocation**, but the effect happens gradually, not immediately.<br><br>---<br><br>## Why strengthening improves confidence<br><br>### 1. Better joint stability<br><br>After a dislocation, the shoulder relies more on muscles because the ligaments are stretched. Strengthening:<br><br>* Improves rotator cuff control<br>* Helps keep the joint centered during movement<br>* Reduces that "slipping" sensation over time<br><br>---<br><br>### 2. Improved coordination (not just strength)<br><br>A big part of "confidence" is actually your nervous system:<br><br>* Early on, your brain is cautious and protective<br>* Rehab retrains how your muscles react quickly and automatically<br>* Movements start to feel smoother and more natural<br><br>---<br><br>### 3. Less fear response<br><br>At first, your body may "warn" you in certain positions. As stability improves:<br><br>* Those warning sensations decrease<br>* You stop anticipating the shoulder coming out<br>* You trust the arm more in daily activities<br><br>---	Yes, strengthening is the **only** way to truly get your confidence back. In the world of rehab, we often say that "strength is the best cure for fear."<br><br>At 20, your brain is currently in "high-alert" mode. It has experienced a trauma (the dislocation) and has rightfully decided that your shoulder is no longer a safe place. Strengthening exercises act as a series of successful "test runs" that prove to your brain that the joint is secure.<br><br>Here is how strengthening builds that mental and physical confidence:<br><br>### 1. Removing the "Vague" Feeling<br>Right now, your shoulder probably feels "hollow" or like it's just dangling. Strengthening the **rotator cuff** (the deep muscles) creates a "compression" effect. When those muscles are strong, they "hug" the bone into the socket. Once you feel that physical "tightness" and "centeredness," the vague, floaty feeling that causes anxiety starts to disappear.<br><br>### 2. Recalibrating Your "Danger Sensors"<br>When you do controlled strengthening (like pushing against a wall or using a light band), you are exposing your shoulder to small, safe amounts of stress.<br>* **The Result:** Your brain realizes, *"Hey, we did that movement and nothing popped out."* * Every successful, pain-free rep is a "vote" of confidence that overrides the memory of the injury.<br><br>### 3. Faster Reflexes<br>Confidence comes from knowing you can handle the unexpected. If you trip or someone bumps into you in a hallway, a weak shoulder will just let the force pull it out. A strong, trained shoulder has **"reactive stability"**—the muscles will reflexively snap tight to protect the joint before you even realize you've been bumped. Knowing your muscles "have your back" is where true confidence comes from.<br><br>---	Yes, absolutely. In fact, for a 20-year-old recovering from a first-time dislocation, building confidence is just as important a goal of these exercises as building muscle. The two are deeply connected.<br><br>What you are experiencing is very common. After a dislocation, the shoulder doesn't just feel physically loose; it feels **unpredictable**. Strengthening exercises are the most effective tool you have to replace that feeling of unpredictability with a feeling of **control**.<br><br>Here is the specific, plain-text explanation of how the exercises rebuild your confidence.<br><br>### How Strengthening Rewires Your Brain (Not Just Your Muscle)<br><br>The shaky, unsure feeling you have is called **Apprehension**. It is a protective reflex from your brain. The brain remembers the trauma of the bone popping out and, as a safety measure, it hesitates to let you move the arm fully.<br><br>Strengthening exercises combat this in three specific ways:<br><br>**1. Restoring Proprioception (The "GPS" of the Joint)**<br>- **What It Is:** Inside your ligaments and muscles are tiny sensors that tell your brain exactly where your arm is in space, even with your eyes closed. When you dislocated, you damaged these sensors.<br>- **How Exercises Help:** When you do **Isometric External Rotation** (pushing outward against the wall), you are not just making the muscle tired; you are recalibrating those sensors. Over time, your brain receives clearer, more accurate data about the position of the ball in the socket.<br>- **The Confidence Boost:** When the brain has good data, it stops being afraid of the unknown. It knows *exactly* where the edge of the socket is and trusts the muscles to stop before reaching it.

	## When you start to feel more confident<br><br>Most people notice:<br><br>* **2–4 weeks:** small improvements in control<br>* **6–8 weeks:** clearer stability in daily use<br>* **3+ months:** major improvement in confidence and function<br><br>(Full return to high confidence in sports usually takes longer.)<br><br>---<br><br>## Important point<br><br>Confidence improves **after consistency**, not after one or two sessions. Early on:<br><br>* Exercises may feel shaky or uncertain<br>* That does NOT mean they are not working<br>* That is part of retraining the system<br><br>---<br><br>## What builds confidence fastest<br><br>* Regular, gentle strengthening (not aggressive)<br>* Avoiding risky positions early on<br>* Gradual exposure to movement<br>* Not skipping rehab sessions<br>* Progressing step by step with control<br><br>---<br><br>## Bottom line<br><br>Yes—**strengthening exercises are essential for rebuilding confidence in your shoulder**, because they restore stability and retrain coordination. The feeling of confidence returns gradually as your shoulder becomes more controlled and reliable.<br><br>---<br><br>If you want, I can explain **which specific exercises are most important for building shoulder stability and confidence after dislocation**, so you know what to focus on.	### The "Confidence" Timeline<br>You'll notice your confidence returning in three distinct stages:<br><br>* **Stage 1 (Internal Confidence):** You can move your arm around your own body (brushing teeth, washing hair) without feeling like you have to move "in slow motion."<br>* **Stage 2 (Environmental Confidence):** You feel okay being in a crowded place or carrying a light bag because you trust your shoulder to stay in place if you move suddenly.<br>* **Stage 3 (Athletic Confidence):** You can reach overhead or "throw" a motion without hesitation. This usually happens around the **4-to-6-month mark** once your strength is back to 90% of your uninjured side.<br><br>---<br><br>### **A Pro Tip for 20-Year-Olds**<br>Don't just train for "size." If you want to feel **confident**, you need to train for **stability**.<br>* Instead of just doing a rep and dropping it, try **"Internal Holds."** * Hold the muscle contraction for 10 seconds. That "time under tension" is what teaches your brain that the joint is stable for long periods, which is a massive confidence booster.<br><br>> **Bottom Line:** You can't "think" your way into being confident with a bad shoulder. you have to **build** your way there. Every day you do your exercises, you are essentially buying a little more "insurance" for your joint.<br><br>**The Expert Guide Question:**<br>On a scale of 1 to 10, how much do you "trust" your shoulder right now when you're just sitting still?	**2. Building the "Dynamic Bumper"**<br>- **The Physical Reality:** The passive seatbelt (ligament) is loose and may always be a little loose. The exercises build a wall of muscle (specifically the back of the rotator cuff and scapula) that acts like a **dynamic bumper**.<br>- **The Confidence Boost:** As these muscles get stronger, you will physically feel the back of the shoulder get tighter when you move your arm forward. This sensation of "tightness in the back" is the direct opposite of the "looseness in the front." That physical tightness translates directly into mental security.<br><br>**3. Reducing Muscle Guarding**<br>- **What It Is:** Right now, the big neck and shoulder muscles are in a state of low-level spasm, trying to be a human cast to protect the joint. This creates stiffness and a feeling of "fragility."<br>- **How Exercises Help:** The specific exercises we discussed teach the small, deep muscles to do their job. When the small muscles start working, the big muscles can finally relax. The shoulder moves smoother and with less effort.<br>- **The Confidence Boost:** You stop feeling like you are made of glass. You start feeling like you have a normal, functioning arm again.<br><br>### The Timeline of Confidence vs. Strength<br><br>It is important to manage your expectations on *when* confidence returns. It **lags behind** strength.<br><br>- **Weeks 2-4:** **Low Confidence.** You have to think about every single movement. This is normal. The brain is in "high alert" mode.<br>- **Weeks 6-8:** **Cautious Trust.** The muscles are stronger, but you still hold your breath when reaching for a seatbelt. This is the critical phase where you must continue the exercises even though you *feel* okay.<br>- **Months 3-4:** **The Shift.** This is when most patients report "forgetting" about the shoulder for a few hours at a time. You will reach for a coffee cup or open a door without pre-planning the movement. **This is the moment the exercises pay off psychologically.**<br>- **Month 6+:** **Functional Confidence.** You trust the shoulder enough to use it in dynamic situations (sports, lifting, catching a falling object).<br><br>### A Crucial Distinction: Confidence vs. Recklessness

<table>
<tr>
<td></td>
<td></td>
<td></td>
<td>There is one warning here, specific to a 20-year-old mindset. The exercises will make you feel confident and strong **long before** the ligament is biologically healed.

- **Good Confidence:** "I can reach into the back seat of the car without panicking." (This is the goal).
- **False Confidence:** "I feel great, I'm going to go throw a football 50 yards." (This is how you end up back in the ER).

Summary
Yes, these exercises are the bridge back to trusting your body. Every Pendulum swing and every Wall Press is a message to your brain saying, *"We are in control. We are safe."* Stick with the program, and that shaky, unsure feeling will slowly be replaced by a quiet, stable assurance.</td>
</tr>
<tr>
<td>57</td>
<td>Returning to sports after a **first anterior shoulder dislocation** depends a lot on the sport and how stable your shoulder becomes during rehab. There isn’t one fixed date, but there are fairly standard ranges.

🕒 Typical return-to-sport timelines

Non-contact sports (running, cycling, gym legs, etc.)

* Often: **3–6 weeks**
* Only if:

  * Pain is minimal
  * Daily movements are comfortable
  * No feeling of instability

Light upper-body non-contact sports (light gym training, swimming drills)

* Usually: **8–12 weeks**
* Only with:

  * Good range of motion
  * Basic strength restored
  * No “slipping” sensation

Contact sports or high-risk sports</td>
<td>Since you're **20**, your "return to play" clock is a bit different than a 40-year-old’s. You have the advantage of faster healing, but you also likely play at a higher intensity, which means the risk of a "collision" re-dislocation is much higher. The standard timeline for returning to sports is usually **4 to 6 months**, but it depends entirely on the **type of sport** you play. --- ### The Sport-Specific Timeline | Sport Type	When You Can Return	Why?
Non-Contact (Running, Cycling)	**6–8 Weeks**	No overhead reaching or impact; the shoulder just needs to be stable enough to "carry" the weight of your arm.
Lower-Body Contact (Soccer)	**3–4 Months**	Risk of falling or being bumped is high. You need enough "reactive strength" to brace yourself during a fall.
Overhead/Racket (Tennis, Swimming, Volleyball)	**4–6 Months**	These put massive stress on the labrum. You need near-perfect rotator cuff endurance to avoid "fraying" the joint.
High-Impact/Collision (Football, Rugby, Wrestling, Basketball)	**6+ Months**	This is the "danger zone." You need 100% structural integrity and peak strength to survive a tackle or a reach for a rebound. | --- ### The "Three-Step" Clearance Process You don't just wake up one day and play a full game. You have to "graduate" through these steps:</td>
<td>Returning to sports after a first-time shoulder dislocation requires patience. For a 20-year-old treated without surgery, a full return to unrestricted sports usually takes at least **3 to 6 months**. While you may be able to perform some limited training sooner, achieving the strength and stability required to safely re-enter a competitive game takes longer than simply feeling "better" .

The Typical Return to Sport Timeline
Recovery is a step-by-step process. The timeline is dictated by biological healing and the achievement of specific functional milestones, not just the calendar.

- **Weeks 0-6: Maximum Protection**
   - **Goal**: Protect the healing ligaments. Avoid positions of risk (arm out to the side and rotated back).
   - **Sport Activity**: **None.** Focus entirely on gentle range of motion and posture exercises prescribed by your physiotherapist .
- **Weeks 6-12: Building a Foundation**
   - **Goal**: Restore full, pain-free movement and begin strengthening the rotator cuff and scapular muscles .
   - **Sport Activity**: You can begin **sports-specific drills** but with **zero contact** and no risk of falling. For example, a tennis player might practice shadow swings or gentle volleys, but no serves or running.
- **Months 3-6: Gradual Return to Training**
   - **Goal**: Regain **symmetrical strength** (your injured shoulder should be as strong as your healthy one) and joint awareness (proprioception) .
   - **Sport Activity**: This is when you can **phase back into training**. Start with non-contact drills, then progress to controlled contact (e.g., pushing a tackle bag), and finally full-contact practice **only after you've been cleared** . It is wise to</td>
</tr>
</table>

	(e.g. football/soccer contact, basketball, martial arts, wrestling, rugby)<br><br>* Usually: **3–6 months**<br>* Sometimes longer in young people with first-time dislocation<br>* Only when:<br><br>* Full strength is restored<br>* Shoulder feels stable in all directions<br>* You’ve completed structured rehab<br>* A doctor/physiotherapist clears you<br><br>---<br><br>### **Overhead/throwing sports**<br><br>(e.g. volleyball, tennis, handball)<br><br>* Often: **4–6+ months**<br>* These place high stress on the same position that caused dislocation<br><br>---<br><br>## ⚠️ Very important for your case (age 20)<br><br>At your age:<br><br>* Risk of re-dislocation is higher if you return too early<br>* Even if pain is gone, the shoulder may still be **structurally vulnerable**<br>* The goal is not just “feeling fine,” but **having true stability and control**<br><br>---<br><br>## ✔ Return-to-sport checklist (more important than time)<br><br>You should only return when you can:<br><br>* Move the arm fully without pain<br>* Do strengthening exercises without instability<br>* Perform sport-specific movements safely<br>* Have no “slipping” or apprehension feeling<br>* Pass clearance from a clinician/physiotherapist<br><br>---<br><br>## ⊘ What to avoid	1. **The "Gym" Phase:** You have full range of motion and your strength on the injured side is at least **90%** of your healthy side.<br>2. **The "Non-Contact" Phase:** You practice your sport solo. (e.g., shooting baskets by yourself, hitting a tennis ball against a wall, or light swimming laps). No one is allowed to touch you.<br>3. **The "Full Contact" Phase:** Once you have zero pain or "apprehension" (fear) during solo practice, you move to full-speed play.<br><br>---<br><br>### The "90% Strength" Rule<br>Most Physical Therapists will not clear a 20-year-old for contact sports until the injured shoulder is **90% as strong** as the uninjured one.<br>* **Why?** If you go back at 70% strength, your muscles will fatigue by the second half of the game. Once they fatigue, the joint becomes "loose," and one awkward bump will pop it right back out.<br><br>---<br><br>### **A Warning for Your Age Group**<br>The highest rate of repeat dislocations happens in **men under 25** who return to sports too early.<br>> If you rush back at 3 months because you "feel fine," but your labrum hasn't fully scarred down, you risk a second dislocation. A second dislocation often leads to **permanent bone loss** in the socket, which usually requires a much more invasive surgery (like a Latarjet procedure) to fix.<br><br>### **The "Expert Guide" Question**<br>What specific sport are you most anxious to get back to? (The rehab for a pitcher is very different from the rehab for a soccer player.)	ease into competition by playing partial games as a substitute before starting a full match .<br>- **6 Months and Beyond: Full Clearance**<br>- **Goal**: Psychological readiness. You should feel confident and have no apprehension (fear of instability) during dynamic movements .<br>- **Sport Activity**: Clearance for full competition. This is the standard timeframe for returning to high-risk sports like rugby, football, or competitive swimming .<br><br>### **Criteria for Returning Safely**<br>You should not return to full sports until you meet these conditions. Rushing back before achieving them is the primary cause of repeat dislocations in patients your age :<br>- **Full Range of Motion**: You can move your arm in all directions without pain, matching your uninjured side .<br>- **Strength Symmetry**: Your injured shoulder has at least 80-90% of the strength of your healthy shoulder .<br>- **No Apprehension**: You can place your arm in the vulnerable "throwing" position without a feeling of anxiety or panic .<br>- **Clearance from a Professional**: Your physiotherapist or surgeon has formally tested your shoulder and cleared you for contact or overhead activity .<br><br>### **Important Note on Recurrence Risk**<br>As a 20-year-old athlete, you are in the highest risk category for the shoulder dislocating again, with studies showing recurrence rates up to 50-80% with non-surgical management in contact sports . If you experience a feeling of **slipping, shifting, or a second full dislocation** during your rehab, report this to your doctor immediately. It may indicate that the structural damage is too significant for rehab alone, and an earlier discussion about surgical stabilization may be needed to safely return to your sport .

	* Returning just because pain is gone<br>* Playing through mild instability<br>* Contact or overhead sport too early<br><br>These significantly increase recurrence risk.<br><br>---<br><br>## 🧠 Bottom line<br><br>* Light activity: **~3–6 weeks**<br>* Most gym/controlled sports: **~2–3 months**<br>* Contact or high-risk sports: **~3–6 months (sometimes longer)**<br><br>---<br><br>If you want, tell me what sport you play—I can give you a **more specific and realistic return timeline and safe progression plan** for that exact activity.		
58	You'll know your shoulder is ready for sports again when it has recovered **not just pain-wise, but in strength, control, and stability under stress**. Feeling "fine" in daily life is not enough on its own—sports put much higher demands on the joint.<br><br>Here are the main signs doctors and physiotherapists look for:<br><br>---<br><br>## ✔ 1. Full, pain-free range of motion<br><br>You should be able to move your arm:<br><br>* All the way up (overhead)<br>* Out to the side<br>* Across your body<br>* Behind your back<br><br>…without pain, tightness, or hesitation.<br><br>---<br><br>## ✔ 2. Strength is close to the other side<br><br>Your injured shoulder should feel:<br><br>* Almost as strong as the healthy one<br>* Able to resist light pushing/pulling without pain	At **20 years old**, your brain will often tell you that you're ready long before your ligaments actually are. "Feeling good" is not the same as being "sports-ready." To safely return to play, you need to pass what physical therapists call **Objective Criteria.**<br><br>You'll know you're ready when you can check off these four specific milestones:<br><br>### 1. The "90% Strength" Test<br>This is the gold standard. A professional will use a dynamometer (a tool to measure force) to compare your two shoulders.<br>* **The Goal:** Your injured shoulder must be at least **90% as strong** as your healthy one in every direction—especially **external rotation** (turning the hand out).<br>* **Why:** If you go back with only 70% strength, your muscles will "give out" during the fourth quarter or a long set, leaving your joint unprotected.<br><br>### 2. Full "Functional" Range of Motion<br>You shouldn't just be able to lift your arm; you need to be able to move it at **high speeds** without hesitation.<br>* **The Test:** Can you reach into the "High-Five" position (arm up and back) quickly and without feeling a "catch," a "pinch," or that nauseating "apprehension" feeling?<br>* **The Goal:** Zero fear when the arm is in vulnerable positions.<br><br>### 3. Power and Plyometric Stability<br>Sports aren't slow. Your shoulder needs to handle **sudden** loads.	This is the most important question you will ask before returning to the field, court, or gym. As a 20-year-old with a first-time dislocation, you cannot rely on the calendar or simply "feeling okay" to determine readiness. You must rely on **specific, measurable criteria**.<br><br>Rushing back before meeting these benchmarks is the number one reason young athletes end up in the emergency room with a second dislocation.<br><br>Here is the clear, plain-text checklist you and your physical therapist will use to determine if your shoulder is truly ready for sports.<br><br>### The 4 Non-Negotiable Criteria for Return to Sport<br><br>You should be able to say "Yes" to all four of these categories before playing a game or practicing at full speed.<br><br>**1. Symmetrical Range of Motion (The Mirror Test)**<br>- **What It Means:** Your injured arm must be able to move exactly as far as your healthy arm in **all** directions without pain.<br>- **The Key Test:** **External Rotation at 90 Degrees.** This is the "throwing position"—arm out to the side, elbow bent 90 degrees, hand pointing toward the sky.<br>- **How to Know You're Ready:** You can hold this position **without holding your breath, without feeling a front pinch, and

* Able to hold positions without shaking excessively

A key idea: **no big difference between right and left shoulder strength**

✓ 3. No “instability” feelings

This is very important for your injury.

You should NOT feel:

* Like the shoulder might slip out
* Sudden fear or “warning sensation” in certain positions
* A sense of looseness when moving quickly

✓ 4. Good control during movement

You should be able to:

* Move your arm smoothly and confidently
* Do exercises without shaking or loss of control
* Perform slow and faster movements without hesitation

✓ 5. Ability to handle sport-like movements

Before returning to sport, you should be able to do:

* Light pushing and pulling
* Quick direction changes of the arm
* Gentle impact or resistance (depending on sport)
* Sport-specific drills (progressively)

✓ 6. No pain or swelling after activity

Even after exercise or practice:

* No increase in pain later that day or next day
* No feeling of “setback” after activity

---

* **The Test:** A common one is the **Closed Kinetic Chain Upper Extremity Stability Test (CKCUEST).** You get into a push-up position and quickly tap your hands back and forth for 15 seconds.
* **The Sign:** If you can do this without your shoulder "diving" inward or feeling shaky, your "reactive" stability is back.

4. The "Fatigue" Challenge
Many re-dislocations happen at the *end* of a game when the athlete is tired.
* **The Test:** Do a full shoulder workout until your muscles feel "spent," then try to perform a sport-specific move (like a light throw or a sport-specific reach).
* **The Goal:** Even when the muscles are tired, the joint should still feel "centered" and secure.

The Final "Confidence" Check
Ask yourself this question: *“If I had to dive for a ball or take a hard bump from an opponent right now, would I hesitate?”*
* If the answer is **"Yes,"** you are not ready. Hesitation causes you to move awkwardly, which actually **increases** your risk of a new injury.
* If the answer is **"I wouldn't even think about it,"** and you've passed the strength tests, you're likely cleared.

The "Safe Entry" Strategy
Even when you are "ready," don't go 100% on day one. Follow the **Rule of Halves**:
* **Week 1:** Practice at 50% speed/intensity. No contact.
* **Week 2:** Practice at 75% speed. Light "drills" with teammates.
* **Week 3:** Full speed practice.
* **Week 4:** Full game play.

A Quick Reality Check
In your 20s, the risk of re-dislocation is highest because the "anchors" (labrum) are still maturing. If you haven't seen a PT for a **discharge exam**, please do so before your first game. They can perform "Special Tests" (like the *Apprehension and Relocation test*) where they physically try to shift the bone to see if the ligaments hold.

The Expert Guide Question:
Which sport-specific movement are you most worried about—is it a heavy lift, a throw, a swim stroke, or a collision?

without your shoulder blade hiking up to your ear.** This specific motion puts maximum stress on the torn front ligaments. If you cannot do this comfortably with the same range as the other side, you are **not ready** for overhead sports or throwing.

2. Strength Symmetry (The 90% Rule)
- **What It Means:** The muscles that stabilize the socket (rotator cuff and shoulder blade) must be nearly as strong as the uninjured side.
- **The Key Test:** **Isometric External Rotation Strength.** A PT will have you push outward against their hand while your elbow is at your side.
- **How to Know You're Ready:** Your injured side should be at least **80-90% as strong** as the healthy side. If there is a significant deficit, the dynamic "bumper" of muscle is not yet strong enough to compensate for the loose ligaments during a hard tackle or fast swing.

3. The Absence of Apprehension (The Mental Test)
- **What It Means:** You can move your arm into vulnerable positions **without** a wave of anxiety or a subconscious muscle spasm.
- **The Key Test:** **The Surprise Reach.** Have a friend or PT stand behind you and gently, unexpectedly push your arm into the throwing position (arm out, hand back) while your eyes are closed.
- **How to Know You're Ready:** Your body stays relaxed. You do not flinch, pull back, or feel a flash of panic. If your brain still perceives the joint as a threat, it will subconsciously inhibit your performance and you will "play scared," which ironically increases injury risk.

4. Functional Sports-Specific Drill Tolerance
- **What It Means:** You have completed **at least 2-4 weeks** of full, unrestricted practice without pain, swelling, or "slipping" sensations.
- **The Key Test:** You can perform the most demanding action of your sport back-to-back without compensation. For example: A swimmer can do 10 sprint freestyle flip-turns; a football player can push off a blocking sled; a tennis player can hit 20 overhead serves.
- **How to Know You're Ready:** You wake up the next morning with normal muscle soreness (like any workout), **not** joint stiffness or sharp front-of-shoulder pain.

Red Flags: Signs You Are *NOT* Ready
Even if the calendar says "Month 6," you must delay return if you experience any of the following during training:

	## ✓ 7. Clearance from a professional<br><br>Even if you feel ready, ideally:<br><br>* A physiotherapist or doctor confirms stability<br>* Functional tests are passed (strength + control + sport drills)<br><br>---<br><br>## ⚠️ Important for your age (20)<br><br>Even if you feel good early:<br><br>* The shoulder may still be healing internally for months<br>* Returning too early is one of the biggest risk factors for recurrence<br>* Confidence should match **objective strength and control**, not just absence of pain<br><br>---<br><br>## 🧠 Simple self-check rule<br><br>You are *likely not ready yet* if:<br><br>* You still think about the shoulder during movement<br>* You avoid certain positions instinctively<br>* You feel even mild instability<br><br>You are *getting close* when:<br><br>* You forget about the shoulder in daily life<br>* Movement feels natural and controlled<br>* Exercises feel easy and stable<br><br>---<br><br>## 🟢 Bottom line<br><br>Your shoulder is ready for sports when it has:<br><br>* Full motion<br>* Near-normal strength<br>* No instability sensations<br>* Good control under speed and load<br>* Professional clearance<br><br>---		- **The "Clunk" Sensation:** During a push-up or a bench press, you feel the ball of the shoulder shift or thud in the front of the socket. (This is a subluxation).<br>- **Dead Arm Syndrome:** After reaching or throwing, the arm feels heavy, numb, or weak for a few seconds.<br>- **Night Pain:** You have trouble sleeping on that side or wake up with a deep ache after a hard practice.<br><br>### The Reality for a 20-Year-Old Patient<br>Because you are young and the tissues are stretchy, your shoulder may feel "strong enough" in the gym **months before** it is "stable enough" for sports. The force of a collision or a high-speed pitch is exponentially greater than the force of a resistance band.<br><br>**Final Clearance:** Do not self-clear. You need a physical therapist or sports medicine doctor to physically test your strength and apprehension with their hands. They can feel subtle joint shifting that you might have mentally learned to ignore. Once they confirm you meet the criteria above, you can return to the sport you love with confidence.

	If you want, I can help you with a **step-by-step “return to sports progression plan”** so you know exactly how to safely move from rehab → training → full play without risking another dislocation.		
59	Short answer: **no—you shouldn’t return to sports if your shoulder still doesn’t feel stable or “normal.”** Even if pain is gone, that lingering “not quite right” feeling is important feedback.<br><br>---<br><br>## Why “not feeling normal” matters<br><br>After a shoulder dislocation, pain often improves before true stability returns. If your shoulder still feels:<br><br>* Unstable<br>* Weak<br>* “Off” or uncertain<br>* Like it might slip in certain positions<br><br>…it usually means the **deep stabilizers (ligaments + rotator cuff control) are not fully ready yet**.<br><br>Sports stress those exact systems.<br><br>---<br><br>## What can happen if you return too early<br><br>If you go back while it still feels not normal:<br><br>* Higher risk of **re-dislocation**<br>* More micro-instability (small “near slips”)<br>* Slower overall recovery<br>* Possible need for surgery later if it keeps happening<br><br>This is especially relevant at your age (20), where recurrence risk is already higher.<br><br>---<br><br>## Pain vs stability (important difference)<br><br>* **No pain ≠ ready**<br>* **Feeling stable + strong + controlled = ready**<br><br>You need all three:<br><br>* No pain during movement	The short answer for a **20-year-old** is: **Almost certainly not.**<br><br>Going back to sports while your shoulder still feels "off" is like driving a car with a loose steering wheel at 100 mph. You might be able to go straight for a while, but the moment you have to make a sudden turn (or take a hit), the system will fail.<br><br>Here is why that "not normal" feeling is a massive warning sign:<br><br>### 1. "Not Normal" = No Stability<br>If your shoulder doesn't feel normal, it’s usually because your **proprioception** (your brain’s GPS for the joint) is still broken. In sports, everything happens in milliseconds. If your brain is even slightly unsure of where the ball of the joint is, it won't fire the protective muscles fast enough to stop a re-dislocation during a collision or a fall.<br><br>### 2. The "Instability Cycle"<br>At your age, your risk of a second dislocation is already statistically high. If you return while it feels "loose" or "weird":<br>* You will subconsciously **hesitate**.<br>* Hesitation leads to **poor form**.<br>* Poor form leads to **extra stress** on the joint.<br>* **Result:** A second dislocation, which often causes permanent bone loss (a "Hill-Sachs" or "Bankart" lesion) that usually requires surgery to fix.<br><br>### 3. The Fatigue Factor<br>You might feel "okay" for the first 10 minutes of a game, but a shoulder that isn't fully recovered will **fatigue 5x faster** than a healthy one. Once those stabilizer muscles get tired, they "let go," and the only thing holding your shoulder together is your already-damaged labrum.<br><br>---<br><br>### Exceptions: When can you go back "early"?<br>There are very few scenarios where you can play before feeling "100%," and they depend entirely on the sport:<br><br>* **Strictly Linear Sports:** If you are a long-distance runner or a track athlete, you can often return early because you aren't using your arms for power or contact.<br>* **The "Braced" Return:** Some athletes return to play wearing a **Sulley Brace** or a shoulder stabilizer. These are restrictive	That's an excellent and very important question. It is completely normal to feel like your shoulder isn't "right" even after the bone is back in the socket. The short and critical answer is: **No, you should not return to sports yet just because the shoulder is back in place.**<br><br>Here is the detailed explanation of why, and what you need to do first.<br><br>### Why You Should Not Play Yet (Even If It Feels "Okay")<br><br>When you dislocate your shoulder at age 20, the ball rips through the labrum (the cartilage bumper that keeps the joint stable). Even though the ER doctor fixed the position of the bone, **that torn cartilage does not heal itself back to the bone without proper rehabilitation.**<br><br>If you go back to sports while the shoulder feels "loose" or "not completely normal," you are at extremely high risk for a second dislocation. Statistics show that for a 20-year-old male athlete, the chance of dislocating it again within one year is between **70% and 90%** if you do not properly strengthen the muscles first. Every time it pops out again, it damages the bone socket more, which can turn a simple recovery into a surgery.<br><br>### The "Not Normal" Feeling<br>You mentioned it doesn't feel completely normal. That is **100% expected**. That feeling is likely:<br>- **Apprehension:** A mental fear that it will pop out again.<br>- **Proprioceptive Deficit:** The nerves that tell your brain where your arm is in space were stretched. Your brain has temporarily "forgotten" how to control the shoulder smoothly.<br>- **Rotator Cuff Weakness:** The small muscles that should be hugging the ball into the socket are currently shut down due to pain and swelling.<br><br>Playing sports with this feeling is like driving a car with a loose steering wheel. You might get away with it for a while, but eventually, you'll crash.<br><br>### The Correct Path Back to Sports<br><br>You can absolutely return to sports and perform at a high level, but you must follow this timeline.

	* Full confidence in the joint<br>* Strength close to the other side<br><br>---<br><br>## What “ready” usually feels like<br><br>You’re closer when:<br><br>* You forget about the shoulder in daily life<br>* Exercises feel controlled, not shaky<br>* No “warning sensation” in risky positions<br>* You can move quickly without hesitation<br><br>---<br><br>## Safer approach<br><br>Instead of full return:<br><br>* Progress gradually (rehab → training drills → partial practice → full sport)<br>* Test sport movements in controlled rehab first<br>* Only return fully when cleared and stable<br><br>---<br><br>## Bottom line<br><br>If your shoulder doesn’t feel completely normal yet, **it’s a sign you are not ready for full sports participation**. Waiting until strength, control, and confidence return significantly lowers your risk of re-injury.<br><br>---<br><br>If you want, tell me what sport you play—I can give you a **safe step-by-step return plan specific to that sport**, so you know exactly what “safe progression” looks like.	harnesses that physically prevent your arm from moving into the "danger zone."<br>* *Note: Even with a brace, your muscles still need to be strong enough to support the joint.*<br><br>### The "Risk vs. Reward" Math<br>Ask yourself: **"Is playing this one game worth potentially needing a 6-month surgical recovery?"**<br>* If you dislocate a second time, the tissue becomes "mushier" and harder for a surgeon to repair later.<br>* Taking an extra 3–4 weeks now to finish your strengthening can save you from a lifetime of "trick shoulder" issues where your arm pops out just from sneezing or reaching for a seatbelt.<br><br>---<br><br>### **The "Green Light" Indicator**<br>You are ready when the shoulder feels **"boring."** When you don't think about it, don't worry about it, and don't feel it "clicking" during your warm-ups, you are close. If you still have to "check in" with your shoulder every few minutes, you aren't ready for the chaos of a game.<br><br>**The Expert Guide Question:**<br>Is there a specific game or tournament coming up that is making you feel like you need to rush back?	**Phase 1: Immobilization (Weeks 1-3)**<br>- You should likely be in a sling. Do **not** lift weights or stretch overhead.<br>- **What you CAN do:** Remove sling for gentle pendulum swings and elbow/wrist motion only. This prevents the elbow from getting stiff.<br><br>**Phase 2: Physical Therapy (Weeks 4-12)**<br>- **This is non-negotiable.** You need a physical therapist (PT) to guide you.<br>- **Goal:** Retrain the Rotator Cuff and Scapular muscles. You will start with isometric exercises (pushing without moving) and very light resistance bands.<br>- **Crucial Rule:** You must avoid the **"High Five Position"** (arm out to the side, palm forward). That is the position of dislocation. Your PT will give you safe ranges of motion.<br><br>**Phase 3: Return to Sport Testing (Months 3-5)**<br>- This is when you can start thinking about *practice*. You do not just "show up" to practice; you must pass a test first.<br>- Your PT or Athletic Trainer will have you perform a push-up, a plank, and specific strength tests. **If your injured side is not at least 90% as strong as your healthy side, you are not cleared.**<br><br>### When Is It Actually Safe?<br>Most first-time dislocations in 20-year-olds require **4 to 6 months** before full-contact or overhead sports (basketball, volleyball, swimming, football) are safe. Some sports like soccer (goalkeeper) or baseball throwing may take **6+ months** to regain full confidence and power.<br><br>### A Note on the Future<br>Because you are 20, you are in the highest risk group for recurrence. Even with perfect rehab, there is a chance of re-injury. If it happens a second time, the conversation shifts strongly toward surgical repair (Bankart repair) to fix the torn cartilage anchor.<br><br>**Summary for today:** Rest in the sling as directed. The feeling of "not normal" is your body's warning light. Do not ignore it. Get a referral to a sports medicine doctor or physical therapist. They will tell you exactly when that warning light turns green.
60	Yes—**some sports carry a much higher risk of shoulder re-dislocation than others**, especially after a first anterior shoulder dislocation like yours.<br><br>The risk mainly depends on whether the sport involves:	Yes, absolutely. Because of the way the shoulder is designed—like a golf ball on a tee—certain sports put the joint in a "levered" position that makes a re-dislocation much more likely.	Yes, absolutely. The type of sport you play is the single biggest factor in whether your shoulder will dislocate again. Since you are 20 years old and have already had a first-time dislocation, understanding this difference is crucial for protecting your shoulder.

	* Contact or collisions<br>* Falling onto an outstretched arm<br>* Overhead throwing or reaching<br>* Sudden force on the shoulder in awkward positions<br><br>---<br><br>## 🟥 High-risk sports (higher chance of re-dislocation)<br><br>These put your shoulder in the *exact vulnerable position* or involve contact:<br><br>* **Rugby / American football**<br>* **Wrestling / grappling sports**<br>* **Judo / martial arts with throws**<br>* **Basketball**<br>* **Football (Soccer) (especially contact play or falls)**<br><br>Why risk is higher:<br><br>* Frequent shoulder contact or falls<br>* Arm often forced into outward rotation or extension<br>* High chance of sudden impact<br><br>---<br><br>## 🟧 Moderate-risk sports<br><br>These involve overhead movement or some impact but less direct contact:<br><br>* **Volleyball**<br>* **Tennis**<br>* **Handball**<br>* Gym training with heavy overhead lifts (if not controlled)<br><br>Why risk is moderate:<br><br>* Repetitive overhead stress (same vulnerable position)<br>* Sudden explosive arm movements<br><br>---<br><br>## 🟩 Lower-risk sports<br><br>These are generally safer once your shoulder has recovered:	Since you are **20**, you are likely moving at a higher speed and intensity, which increases the "shear" forces on the joint. Here is how the risk breaks down by sport:<br><br>### 1. The "High-Five" Sports (Highest Risk)<br>Any sport that requires **abduction and external rotation** (lifting your arm up and rotating it back) is high risk. This position naturally shifts the ball to the very edge of the socket.<br>* **Basketball:** Reaching back for a rebound or having your arm swiped while shooting.<br>* **Volleyball:** The "cocking" phase of a spike.<br>* **Tennis:** The overhead serve.<br>* **Water Polo/Handball:** Throwing while being jostled by an opponent.<br><br>### 2. The Collision & Fall Sports (High Risk)<br>In these sports, the injury usually happens because of an external force (an opponent) or an uncontrolled landing.<br>* **Football/Rugby:** Tackling with an outstretched arm or landing hard on the point of the shoulder.<br>* **Wrestling/BJJ:** Having your arm "posted" or trapped in a levered position.<br>* **Skateboarding/Snowboarding:** "FOOSH" injuries (**F**all **O**n **O**utstretched **H**and). This pushes the arm bone directly up and out of the socket.<br><br>### 3. The "Traction" Sports (Moderate/High Risk)<br>These sports don't involve hitting anyone, but they "pull" on the joint.<br>* **Gymnastics/Calisthenics:** Hanging from bars or doing rings. The weight of your body creates "traction," which can stretch a healing labrum.<br>* **Swimming:** Specifically the "catch" phase of the freestyle stroke or butterfly, which requires repetitive, high-force overhead rotation.<br><br>### 4. The "Safety" Sports (Lowest Risk)<br>These are sports where the arms stay mostly in front of the body or are not used for power.<br>* **Running/Cross Country.**<br>* **Cycling** (Assuming you don't crash).<br>* **Soccer** (Though headers and falling during tackles carry some risk).<br><br>---<br><br>### **The "Danger Zone" Visualized**<br>Imagine your shoulder is a clock.<br>* **Safe Zone:** 12 o'clock to 6 o'clock in *front* of your body.	The risk is not the same across all sports. It depends entirely on where your arm needs to be positioned during play. The most vulnerable position for your shoulder is **abduction and external rotation**. That means: arm raised to the side at shoulder height (or higher) with the palm facing forward or up—exactly like a baseball pitch or a volleyball spike.<br><br>Here is how sports break down by risk level for your specific injury:<br><br>### High-Risk Sports (Very Likely to Cause Re-Dislocation)<br>These sports require you to put your arm in that "high-five" position forcefully or suddenly. You need to be extremely cautious and fully cleared by a physical therapist before returning to these.<br><br>- **Overhead Throwing:** Baseball (especially pitching), Football Quarterback, Javelin.<br>- **Overhead Hitting:** Volleyball (spiking/serving), Tennis (serve), Badminton smash.<br>- **Swimming:** Specifically Freestyle, Backstroke, and Butterfly. The repetitive overhead pull puts constant stress on the front of the joint where you tore the cartilage.<br>- **Contact Sports Above the Waist:** Wrestling, Rugby, American Football (tackling), and Boxing. Even a slight shove to an outstretched arm can easily pop it out again.<br>- **Gymnastics and Rock Climbing:** These involve hanging and pulling with the arm fully extended overhead.<br><br>### Moderate-Risk Sports (Manageable with Bracing and Rehab)<br>These sports keep the arm lower or involve the body more than the arm, but they still carry a risk of falling on an outstretched hand.<br><br>- **Basketball:** Rebounding and reaching for a steal overhead are the danger moments.<br>- **Soccer:** This is generally low risk *unless you are a goalkeeper* diving with arms extended. For field players, the risk is falling incorrectly after a slide tackle.<br>- **Weightlifting:** Certain lifts are safe (bicep curls, rows). Others are dangerous: **Behind-the-neck press, Wide-grip bench press, and Dips.** These stretch the front capsule of the shoulder exactly where it is already loose.<br><br>### Lower-Risk Sports (Safer Options During Recovery)

	* Running / jogging<br>* Cycling<br>* Swimming (after rehab and clearance)<br>* Controlled gym work (no heavy overhead or contact early on)<br><br>Why risk is lower:<br><br>* Less contact and less extreme shoulder positions<br><br>---<br><br>## ⚠️ Important for your situation (age 20)<br><br>At your age:<br><br>* The shoulder is **more likely to re-dislocate in high-risk sports**<br>* Risk is highest if you return before full rehab completion<br>* Proper strengthening reduces risk but does not eliminate it<br><br>---<br><br>## 🧠 Key idea<br><br>It's not just the sport itself—it's:<br><br>* How much contact there is<br>* How often your arm is in the "danger position"<br>* Whether your shoulder is fully rehabilitated<br><br>---<br><br>## ✓ Bottom line<br><br>Yes—**contact and overhead sports significantly increase the chance of your shoulder coming out again**, especially if you return too early. Lower-risk sports are safer during recovery, and return to higher-risk sports should only happen after full strength, control, and clearance.<br><br>---<br><br>If you want, tell me your specific sport—I can give you a **personalized risk level and a safe return-to-play timeline** for that exact activity.	* **Danger Zone:** Anything reaching behind the plane of your body (like reaching into the backseat of a car) or reaching high and wide.<br><br>### **How to Lower the Risk in Your Sport**<br>If you play a high-risk sport, you don't necessarily have to quit, but you do need to adapt:<br>* **Bracing:** Wearing a "Shoulder Savers" or "Sulley" brace can physically block your arm from entering the "Danger Zone."<br>* **Technique Tweaks:** A swim coach or throwing coach can help you move your arm in a way that uses more of your back and less of the "edge" of the shoulder joint.<br>* **Over-Training the Rotator Cuff:** If you play a "High-Five" sport, your rotator cuff needs to be twice as strong as a normal person's to keep that ball centered.<br><br>> **The Reality Check:** At 20, if you return to a **collision sport** (like football or wrestling) without a full 4–6 months of rehab, the chance of it coming out again is estimated to be over **50–70%**.<br><br>**The Expert Guide Question:**<br>Based on that list, does the sport you play fall into the "High-Five" or "Collision" category?	These sports keep your elbow close to your side or involve the lower body primarily. These are actually encouraged during your rehab phase to maintain fitness.<br><br>- **Running / Cycling:** Very safe as long as you don't crash and land on the shoulder.<br>- **Soccer (Field Player):** Relatively safe once you are out of the sling and cleared for running.<br>- **Rowing (Machine):** Safe because the motion is in front of the body, not overhead.<br>- **Closed-Chain Exercises:** Push-ups on the knees (once cleared) are safer than bench press because your hand is fixed to the ground, which stabilizes the joint.<br><br>### A Critical Rule for Overhead Athletes<br>If you play a high-risk sport like baseball or volleyball, you cannot rely on just **feeling** ready. You need **objective clearance**. This usually involves a test called an **Isokinetic Strength Test** where a machine measures the power of your external rotators (the muscles that keep the ball in the socket). If those muscles are not at least 90% as strong as your healthy side, the ball will slide forward the moment you try to throw hard.<br><br>**Bottom Line:** If you are a swimmer, pitcher, or volleyball player, your return-to-play timeline is significantly longer (closer to 6 months) and should involve a conversation with a sports medicine doctor about whether bracing or even early surgical repair is a better long-term option for you. If you are a runner or cyclist, you can resume those activities much sooner.
61	Yes—**it's very normal to feel scared or hesitant about using your shoulder again in sports after a dislocation**. That reaction is actually common and expected, especially in the first weeks to months.<br><br>---	It is **100% normal**. In fact, almost every athlete who has suffered a dislocation—from pros in the NFL to 20-year-olds playing pickup games—deals with this. There is even a medical term for it: **Kinesiophobia** (the fear of movement).	Yes, it is absolutely normal. In fact, it is so common that sports medicine doctors and physical therapists have a specific name for it: **Kinesiophobia**—the fear of movement. You are not weak or mentally soft for feeling this way; your brain is actually doing its job.

	## Why you feel scared<br><br>### 1. Your brain is protecting you<br><br>After a dislocation, your nervous system learns that certain positions were “dangerous.” So it becomes:<br><br>* More alert in those positions<br>* Quick to create a “warning feeling”<br>* Protective even when healing is progressing<br><br>This is called a **protective or guarding response**.<br><br>---<br><br>### 2. Your confidence and strength don’t return at the same time<br><br>* Pain often improves first<br>* Strength and joint stability take longer<br>* Confidence usually comes **last**<br><br>So even if your shoulder is improving physically, your mind may still feel unsure.<br><br>---<br><br>### 3. You remember the injury<br><br>It’s normal for your brain to “replay” the moment it dislocated, especially when thinking about returning to:<br><br>* Contact sports<br>* Falling<br>* Overhead movement<br><br>This creates caution, which feels like fear.<br><br>---<br><br>## Is this fear a bad sign?<br><br>Not necessarily.<br><br>* Mild fear = **normal and often helpful early on**<br>* It helps you avoid risky movements while healing<br>* It usually decreases as strength and stability improve<br><br>---	At your age, your brain has a very fast "learning curve" for trauma. It remembers the pain and the "wrongness" of the joint coming out, and it is now trying to protect you by sending signals of fear.<br><br>Here is why that fear is actually a biological tool, and how to handle it:<br><br>### 1. Your Brain’s "Internal Governor"<br>Think of your fear like a "speed limiter" on a car. Your brain isn't sure if the "repairs" (your rehab) are finished yet, so it makes you feel scared to prevent you from doing something reckless.<br>* **The Good News:** This fear keeps you from rushing back too early.<br>* **The Process:** As you get physically stronger, your brain slowly "unlocks" more movement.<br><br>### 2. The "Apprehension" Reflex<br>When you move your arm toward the position where it originally popped out, your nervous system will physically tighten your muscles. This is a reflex. It can feel like a "ping" of anxiety or a sudden "brake" in your movement.<br>* **How to fix it:** You can't talk yourself out of this reflex; you have to **train** your way out of it. Thousands of successful, pain-free repetitions in rehab are the only way to "prove" to your nervous system that the danger has passed.<br><br>### 3. The "Trust" Gap<br>Confidence is the last thing to return—usually **months** after the pain has gone away.<br>* **Months 1–2:** You trust it for daily life.<br>* **Months 3–4:** You trust it for the gym.<br>* **Months 5–6+:** You trust it for the "chaos" of sports.<br><br>---<br><br>### How to Overcome the Fear<br>If you try to "just ignore it" and jump into a game, you are actually at a **higher risk** of injury. Fear makes you move stiffly, and stiff joints break more easily than fluid ones. Instead, try this:<br><br>* **Gradual Exposure:** Don't go from "rehab" to "full game." Start by just playing catch, then light practice, then non-contact drills. Each "win" builds a layer of confidence.<br>* **Visualization:** It sounds "woo-woo," but elite athletes use this. Spend 5 minutes a day picturing yourself making a move in your sport where your shoulder stays perfectly stable and strong.<br>* **Wear the Gear:** If wearing a light compression sleeve or a brace makes you feel "tucked in" and safe, do it! The psychological	Here is why that fear exists and, more importantly, how to get past it so it doesn't hold you back forever.<br><br>### Why You Are Scared (The Science of the "Pop")<br>When your shoulder dislocated, your body experienced a significant trauma. Your brain logged that event as a **"Red Alert"** emergency. The brain's primary job is survival, not sports performance. It has now created a protective reflex.<br><br>Even though the X-ray shows the bone is back in place, your nervous system remembers the exact position where the injury happened. Every time your arm drifts toward that **"high-five"** or overhead position, your brain instinctively slams on the brakes. That braking sensation feels like tightness, a catch, or pure fear.<br><br>This is not just in your head; it's neurological. The muscles that stabilize your shoulder (the rotator cuff) are still inhibited and weak from the injury. Your brain knows the muscles aren't strong enough to protect you yet, so it sends a **pain signal** even when there is no actual danger, just to make you stop moving.<br><br>### The Danger of Ignoring the Fear vs. Listening to It<br>There are two ways this can go:<br><br>1. **Ignoring It Completely (The Wrong Way):** If you try to be "tough" and power through the fear before the muscles are ready, you are relying entirely on the torn cartilage to hold the shoulder in. This is how **90% of re-dislocations happen** in 20-year-olds. You are right to be cautious.<br>2. **Being Paralyzed By It (The Unnecessary Way):** If you let the fear stop you from doing your physical therapy exercises, the shoulder stays weak forever. The longer you avoid moving it, the tighter and more frozen it feels.<br><br>### How to Turn the Fear Off (The Rehab Approach)<br>The only way to make the fear go away is to prove to your brain that the shoulder is safe. You cannot do this by just waiting for time to pass. You have to retrain the system.<br><br>Here is the process that works:<br><br>**Step 1: Build the "Brakes" Before You Use the "Gas"**<br>In rehab, you will focus heavily on **External Rotation** (pulling your hand away from your belly against a band). This motion activates the muscles that pull the ball *back* into the socket. When these muscles are strong, your brain gets a new

	## When it becomes a problem<br><br>It may need more attention if:<br><br>* Fear doesn’t improve over time even as strength returns<br>* You avoid all use of the arm, even in safe situations<br>* You feel panic or strong anxiety at the idea of movement<br>* You feel unable to progress in rehab despite physical recovery<br><br>---<br><br>## How confidence usually returns<br><br>Confidence builds gradually through:<br><br>* Safe, repeated movement<br>* Strengthening exercises<br>* Positive experiences without pain or instability<br>* Gradual return to sport-specific drills<br><br>Eventually, your brain “re-learns” that the shoulder is safe again.<br><br>---<br><br>## Important point<br><br>Feeling scared does **not mean your shoulder is damaged again**. It usually reflects:<br><br>* Normal psychological response<br>* Ongoing recovery of stability and control<br><br>---<br><br>## Bottom line<br><br>Yes—**it is completely normal to feel scared about returning to sports after a shoulder dislocation**, and this fear usually decreases as your shoulder becomes stronger, more stable, and more trusted over time.<br><br>---<br><br>If you want, I can help you with a **step-by-step plan to rebuild confidence in sports safely**, so you can return without feeling like you’re “risking it every time you move.”	"hug" of a brace can help lower your brain's alarm bells while you transition back.<br><br>### **The "Safe" Mindset**<br>Don't beat yourself up for being scared. It doesn't mean you're "weak" or "not an athlete." It means your brain is doing its job of keeping you safe. Trust the process: **Strength breeds stability, and stability breeds confidence.**<br><br>> **One final thought:** Most 20-year-olds find that once they take that first "hit" or make that first "big play" and realize the shoulder held up, the fear vanishes instantly. You just have to build the foundation to get to that moment safely.<br><br>**The Expert Guide Question:**<br>When you think about playing your sport again, is there one specific move (like a certain hit or a specific reach) that gives you that "stomach-drop" feeling?	signal: *"It's okay, the brakes are on, the shoulder won't slip."* Fear decreases as strength increases.<br><br>**Step 2: Proprioception Drills (Eye Closed Balance)**<br>This is critical for fear. You need to do exercises with your **eyes closed** while on your hands and knees (plank position). This forces the brain to trust the shoulder joint's position sensors without relying on vision. It re-teaches the brain that the arm is attached securely.<br><br>**Step 3: The "Safe" Return to Sport**<br>When you finally go back to practice, you should **not** go 100% on Day 1. You need to trick your brain into trusting the arm again with low-stakes drills:<br>- **Basketball:** Start with dribbling and chest passes. **Do not attempt a rebound** for several weeks.<br>- **Swimming:** Start with kicking with a board and a pull buoy. **Do not attempt a full freestyle stroke** until cleared.<br><br>### The "Second Injury" Phenomenon<br>Here is an encouraging fact: **Once you return to play and take your first hard fall or make your first hard throw *without* the shoulder coming out, the fear often vanishes instantly.** The brain logs a new memory: *"We survived that. We are safe now."*<br><br>But you **must** earn the right to take that first hit by doing the boring, repetitive band work first.<br><br>**Summary for you:** Feeling scared is a sign of a healthy, intelligent nervous system protecting you. Do not rush it. Use that fear as motivation to get your rotator cuff bulletproof. As the strength returns, the fear naturally fades into the background. If the fear persists even after you are cleared medically, that is also normal, and a sports psychologist can help with specific mental tools for a day or two.
62	Yes—**returning to sports too early is one of the biggest reasons the shoulder comes out again after a first dislocation.**	It is completely understandable that you want to get back to your sport as soon as possible, but the short answer is **yes**. Returning	Yes. Going back too early is not just a risk factor; it is the **direct cause** of repeat dislocations in patients your age. The

	After an anterior shoulder dislocation, the joint may *feel better before it is actually stable*, which is why timing matters so much.<br><br>---<br><br>## Why early return increases risk<br><br>### 1. Healing structures are still weak<br><br>Even if pain improves, the:<br><br>* labrum<br>* ligaments<br>* joint capsule<br><br>are still healing for **weeks to months**. Sports can stress them before they're ready.<br><br>---<br><br>### 2. Sports put the shoulder in "danger positions"<br><br>Many sports involve:<br><br>* Arm out to the side<br>* Arm rotated outward<br>* Sudden impact or falling<br>* Overhead force<br><br>These are exactly the positions that can trigger another dislocation.<br><br>---<br><br>### 3. Muscles are not fully retrained yet<br><br>After injury:<br><br>* Rotator cuff muscles are weak and slow to react<br>* Shoulder blade control is not fully coordinated<br>* Reaction timing is reduced<br><br>So the shoulder doesn't protect itself well yet during fast movements.<br><br>---<br><br>## What "too early" usually means<br><br>Risk is highest if you return when:	to sports before your shoulder has properly healed and strengthened significantly increases the risk of it coming out again.<br><br>Because you are 20 years old, your risk profile is different than it would be for an older adult. Here is a breakdown of why timing matters so much for your recovery:<br><br>## The Healing Process<br>Even though your shoulder is back in its socket, the "soft tissues" that hold it there—specifically the **labrum** (a rim of cartilage) and the **ligaments**—were stretched or torn during the dislocation.<br>* These tissues do not have a robust blood supply, so they heal slowly.<br>* Think of it like a door frame that has been warped; if you don't fix the frame before you start slamming the door again, the door won't stay shut.<br><br>## The Age Factor<br>Statistically, younger athletes (under age 25) have a much higher rate of **recurrent instability**—meaning the shoulder is more likely to dislocate a second or third time. This is often because younger tissues are more flexible, and the physical demands of your sports are typically higher. If you return too early, you may develop "chronic instability," which often eventually requires surgery to fix.<br><br>## Muscle Protection vs. Structural Weakness<br>Right now, your shoulder is relying heavily on your muscles for stability because the ligaments are compromised. If those muscles fatigue during a game or if you haven't trained them to react to sudden movements, the joint becomes vulnerable.<br>* **Early return:** Muscles are weak, ligaments are loose = High risk of re-injury.<br>* **Gradual return:** Muscles are "retrained" to hold the joint in place = Lower risk.<br><br>## What to Look For<br>Before you head back to the field or court, you should generally meet these criteria:<br>* **Full Range of Motion:** You can move your arm in all directions without pain.<br>* **Symmetry:** Your injured shoulder is nearly as strong as your uninjured one.<br>* **Functional Drills:** You can perform sports-specific movements (like throwing, bracing, or tackling) without the sensation that the shoulder is "slipping."<br><br>It is highly recommended that you work with a physical therapist who specializes in sports medicine. They can give you a specific	statistics in sports medicine are very clear on this specific scenario.<br><br>If you return to sports before the muscles are strong enough and before the swelling has fully settled, the answer is a definitive **yes—it will almost certainly come out again.**<br><br>Here is the detailed explanation of *why* going back early causes failure, and what happens mechanically inside your shoulder when you push the timeline.<br><br>### The "Loose Ligament" Reality<br>When you dislocated your shoulder, the ball of the humerus tore through the **labrum** (the cartilage bumper) and stretched the **capsule** (the ligaments wrapping the joint).<br><br>Right now, those tissues are like a stretched-out rubber band. **They will never shrink back to their original tightness on their own.** Nature does not tighten loose ligaments. Therefore, the only thing holding your shoulder in the socket right now is **muscle contraction.**<br><br>If you go back to sports *before* you can fire those muscles fast enough to catch the ball when it slips, the joint will pop out with much less force than it took the first time. A simple high-five, a swim stroke, or reaching for a rebound becomes enough force to dislocate it again.<br><br>### What Happens If It Comes Out Again?<br>This is the most important part for a 20-year-old to understand. You might think, *"If it pops out, I'll just go to the ER and pop it back in like last time."*<br><br>**That is a dangerous assumption.** Here is the cascade of damage that occurs with a second dislocation:<br><br>1. **Hill-Sachs Lesion (The Bite Mark):** When the shoulder dislocates, the soft ball of the humerus slams against the hard rim of the socket. This leaves a dent or "bite mark" on the ball. Every time it dislocates, **that dent gets bigger.**<br>2. **Bone Loss:** If that dent gets too big (more than 20% of the ball's surface), the shoulder becomes **unstable even with perfect muscles.** At that point, you can no longer rehab your way out of it. You would require a complex bone graft surgery (called a Latarjet procedure) instead of a simple soft tissue repair.<br>3. **Arthritis:** Each dislocation causes cartilage damage. A shoulder that dislocates 3-5 times will develop arthritis 10-15 years earlier than a shoulder that was protected.

	* You still feel instability or “slipping”<br>* Strength is clearly weaker than the other side<br>* You haven’t completed rehab exercises consistently<br>* You haven’t tested sport-specific movements safely<br><br>---<br><br>## What can happen if you return too early<br><br>* Re-dislocation (most important risk)<br>* Repeated “near slips” (subluxations)<br>* Slower healing and longer rehab overall<br>* In some cases, increased likelihood of needing surgery later<br><br>This is especially relevant at your age (20), where recurrence risk is naturally higher.<br><br>---<br><br>## When return becomes safer<br><br>Sports are usually safer when:<br><br>* Full range of motion is restored<br>* Strength is close to the other side<br>* No instability sensations remain<br>* You can do sport-like movements in rehab without pain or fear<br>* A doctor or physiotherapist clears you<br><br>---<br><br>## Bottom line<br><br>Yes—**going back to sports too early can significantly increase the chance of your shoulder coming out again**, because the joint is not fully stabilized yet even if it feels better.<br><br>---<br><br>If you want, I can tell you a **simple “readiness checklist” for your specific sport**, so you can clearly know when it’s actually safe to return without guessing.	timeline based on the physical demands of your particular sport. Dealing with a few weeks of rehab now is much better than dealing with a lifetime of shoulder issues.	 ### The Timeline of Failure<br>When doctors see a **"Recurrent Dislocator"** in the office, the story almost always sounds like this:<br>- *Month 1-2:* Shoulder feels "fine" in daily life. Sling is annoying and comes off early.<br>- *Month 3:* Patient starts shooting hoops or swimming easy laps. Shoulder feels a little "loose" or "catches," but they play through it because they are 20 and want to get back.<br>- *Month 4:* **The Re-Dislocation.** It happens on a routine motion (throwing a ball from the outfield, reaching for a high shelf, or even just rolling over in bed). It pops out again.<br><br>### The Hard Truth About Returning Too Early<br>If you return to sports **before 4 months** for a contact/overhead sport:<br>- The capsule is still inflamed and stretchy.<br>- The rotator cuff reflexes are slow.<br>- The result: The ball slides forward (subluxation) and either pops out fully or, worse, stretches the capsule *even more* making future surgery less successful.<br><br>**Summary:** Yes, returning too early causes re-dislocation because your muscles are the only thing holding the shoulder in place, and those muscles are currently **inhibited and weak** from the trauma. Waiting the full 4-6 months (and passing a strength test) is not about being overly cautious; it is about protecting the bone structure so you don't need a much bigger surgery when you are 25.
63	Yes—**you do need specific exercises before returning to sports**, not just general daily use. After a shoulder dislocation, sports require **strength, control, and reaction stability**, not just absence of pain.<br><br>---<br><br>## Why specific exercises matter	Yes, performing specific exercises is arguably the most important part of your recovery. Because your ligaments were stretched during the dislocation, your shoulder is now "looser" than it used to be. You have to train your muscles to act as a dynamic brace to compensate for that looseness.	Yes, absolutely. This is not optional. You cannot simply wait for time to pass and then show up to practice. You must complete a specific, targeted exercise program to re-train the muscles that are currently "offline."

	Your shoulder doesn’t just need to heal—it needs to:<br><br>* Regain **rotator cuff strength** (keeps the joint centered)<br>* Restore **shoulder blade control** (scapular stability)<br>* Retrain **fast protective reactions**<br>* Build tolerance to sport-like positions<br><br>Without this, the shoulder may feel “fine” in daily life but still be **unstable in sport situations**.<br><br>---<br><br>## Types of exercises you usually need before returning to sport<br><br>### 1. Early activation (foundation stage)<br><br>* Pendulum exercises<br>* Gentle range-of-motion work<br>* Shoulder blade setting (scapular control)<br><br>Goal: restore safe movement and basic control<br><br>---<br><br>### 2. Strengthening (core stability phase)<br><br>* External rotation with resistance band (rotator cuff)<br>* Internal rotation with resistance band<br>* Light rows or pulling movements<br>* Isometric shoulder exercises<br><br>Goal: rebuild stability muscles<br><br>---<br><br>### 3. Functional control (sport preparation)<br><br>* Closed-chain exercises (hands supported, like wall pushes)<br>* Controlled overhead movements<br>* Balance + stability drills for shoulder<br><br>Goal: teach the shoulder to stay stable under load<br><br>---<br><br>### 4. Sport-specific drills<br><br>* Throwing motions (for overhead sports)	If you jump straight back into sports without targeted training, you are relying on luck to keep the joint in place. Here is how a typical exercise progression looks for a first-time dislocation:<br><br>## 1. Rotator Cuff Strengthening<br>The rotator cuff is a group of four small muscles that surround your shoulder joint. Their primary job is to pull the head of your arm bone tightly into the socket.<br>* **The Goal:** Build endurance in these muscles so they don't tire out during a game.<br>* **The Exercises:** Usually involve "internal and external rotation" using light resistance bands or small weights.<br><br>## 2. Scapular (Shoulder Blade) Stability<br>Your shoulder blade is the foundation of your shoulder joint. If the shoulder blade doesn't move correctly, the socket won't be in the right position to support your arm.<br>* **The Goal:** Ensure your shoulder blade "tracks" properly when you reach or throw.<br>* **The Exercises:** Often include movements like "rows," "scapular squeezes," or "serratus punches."<br><br>## 3. Proprioception Training<br>This is a fancy word for "joint position sense." After a dislocation, your brain loses some of its ability to sense exactly where your shoulder is in space.<br>* **The Goal:** Retrain your nerves to react instantly to sudden movements.<br>* **The Exercises:** Might include balancing your hand on a physical therapy ball against a wall or doing controlled "plank" variations.<br><br>## 4. Sport-Specific Drills<br>Once you have a baseline of strength, you have to bridge the gap between rehab and the game.<br>* **The Goal:** To ensure the shoulder stays stable during high-velocity or contact movements.<br>* **The Exercises:** If you play a throwing sport, this involves a gradual throwing program. If you play a contact sport, it involves controlled "impact" drills or weight-bearing exercises like push-ups.<br><br>## Why "General Lifting" Isn't Enough<br>Many athletes think that just doing standard bench presses or overhead presses at the gym is enough. However, these "big" exercises often use the large muscle groups (like your pecs and deltoids) which can actually pull the shoulder out of the socket if the smaller stabilizer muscles aren't working correctly.	Going back to sports without doing these specific exercises is like trying to run a race on a leg that has been in a cast for two months with no physical therapy. You will fall.<br><br>Here are the specific types of exercises you need to master, in the exact order you should do them. Please note: **Do not start the strengthening phase until your doctor or physical therapist clears you to remove the sling and begin active motion.**<br><br>### Phase 1: Activating the "Brakes" (Isometrics & Rotator Cuff)<br>*Goal: Wake up the muscles that were stretched and shut down during the dislocation.*<br><br>**The Critical Muscle: The Subscapularis and Infraspinatus.** These muscles act like the reins of a horse, pulling the ball of the shoulder back into the socket. In an anterior dislocation, the *front* of the shoulder stretched out. Therefore, you must strengthen the **back** of the shoulder to compensate.<br><br>**Specific Exercise 1: External Rotation (The Most Important One)**<br>- **How:** Lie on your good side. Place a rolled-up towel between your injured elbow and your ribs. Hold a very light weight (1-2 lbs) or a can of soup. Keeping your elbow glued to your side, rotate your forearm up toward the ceiling like a gate swinging open.<br>- **Why:** This is the exact opposite motion of the dislocation. It tightens the back of the capsule and prevents the ball from slipping forward.<br>- **When to start:** Usually around Week 4-6, *only* if pain-free.<br><br>**Specific Exercise 2: Scapular Retraction (Rows)**<br>- **How:** Using a resistance band anchored in front of you, pull your elbows back, squeezing your shoulder blades together. Do **not** let your shoulders shrug up to your ears.<br>- **Why:** The shoulder blade is the foundation of the shoulder joint. If the foundation is slouched and weak, the ball is already halfway out of the socket before you even move your arm. You need a stable platform for the arm to move on.<br><br>### Phase 2: The "Closed Chain" Safety Zone<br>*Goal: Build confidence and strength without risking the arm drifting into the danger zone.*<br><br>Before you are allowed to lift your arm overhead or throw, you should be able to do these safely.

	* Contact preparation drills (for contact sports)<br>* Quick directional arm movements<br><br>Goal: prepare for real game situations<br><br>---<br><br>## Important point<br><br>You should NOT return to sport based on:<br><br>* “It feels okay”<br>* “Pain is gone”<br>* “I can move it normally”<br><br>You return when:<br><br>* Strength is close to the other side<br>* Control is good even under speed<br>* No instability feelings remain<br>* You can do sport-specific drills safely<br>* A clinician clears you<br><br>---<br><br>## What happens if you skip this stage<br><br>Returning without proper rehab increases risk of:<br><br>* Re-dislocation<br>* “Near-slip” episodes<br>* Longer recovery time overall<br><br>---<br><br>## Bottom line<br><br>Yes—**you need specific, progressive shoulder rehabilitation exercises before returning to sports**, especially strengthening and control work. This is what actually rebuilds stability and protects you from another dislocation.<br><br>---<br><br>If you want, I can build you a **simple step-by-step return-to-sport exercise progression based on your sport**, so you know exactly what to do each phase.	**A physical therapist is your best resource here.** They will test your strength levels and give you a "return-to-play" protocol that ensures your stabilizers are firing correctly before you take your first hit or make your first fast play.<br><br>What specific sport are you hoping to get back to?	**Specific Exercise 3: Push-Up Plus (on Knees)**<br>- **How:** Get on hands and knees. Do a partial push-up. At the **top** of the push-up, push the floor away even further, rounding your upper back slightly.<br>- **Why:** This activates the **Serratus Anterior**. This is a secret weapon muscle that prevents the shoulder blade from winging out and causing impingement or instability when you reach overhead. If this muscle is weak, swimming or throwing will be painful and unstable.<br><br>**Specific Exercise 4: Plank with Shoulder Taps**<br>- **How:** Hold a push-up position on your knees (or toes if strong). Slowly lift your good hand to tap your injured shoulder. **The goal is to keep your hips and the injured shoulder completely still.** No wobbling.<br>- **Why:** This trains your brain and muscles to stabilize the joint under a shifting load—exactly what happens when you reach for a ball or make a tackle.<br><br>### Phase 3: Advanced Sport-Specific Prep (Months 3-4+)<br>*Goal: Simulate the demands of your sport in a controlled environment.*<br><br>**If you are a Thrower / Overhead Athlete:**<br>- **Exercise:** **Prone Y's and T's** (lying on stomach, lifting arms in the shape of a Y and T).<br>- **Specific Note:** You must avoid **Empty Can** exercises (thumbs down lift). This position pinches the rotator cuff and mimics the dislocation position. You want **Full Can** (thumbs up) lifts.<br><br>**If you are a Swimmer:**<br>- **Exercise:** **Standing Row with External Rotation.** Pull the band toward your chest, then rotate your forearm out. This mimics the pull-through phase of freestyle without the dangerous recovery phase overhead.<br><br>### The "Return to Sport" Test<br>You should not play a game or match until you can pass this simple test on the injured side compared to the healthy side:<br><br>1. **Strength Symmetry:** Can you do the External Rotation exercise (Phase 1, Exercise 1) with the exact same weight and repetitions on the injured arm as the healthy arm?<br>2. **Endurance:** Can you hold a side plank on the injured elbow for 60 seconds without the shoulder burning or feeling like it's "slipping"?<br>3. **The Wall Slide Test:** Can you stand facing a wall and slide your hands up the wall overhead until your arms are

			straight, **without** shrugging your shoulder up to your ear? If you cannot do this smoothly, you are not ready for overhead sports.<br><br>**Summary:** You need to do specific rotator cuff and shoulder blade exercises **every other day** for at least 12-16 weeks. Doing random gym lifts like bench press or bicep curls will **not** stabilize your shoulder; they often make the imbalance worse. You must prioritize the small, boring, stabilizing muscles first.
64	In most cases, **a brace or taping can help a bit, but it is not enough on its own to make a shoulder safe for sports after a dislocation**.<br><br>Think of it as **extra support**, not protection that replaces proper healing and rehabilitation.<br><br>---<br><br>## 🟢 Does taping or a brace help?<br><br>Yes, but only modestly:<br><br>* Can give **slight sense of stability**<br>* May improve **confidence**<br>* Can remind you to avoid risky positions<br><br>Common options include:<br><br>* Shoulder braces that limit extreme outward rotation<br>* Athletic taping by a trained physiotherapist<br><br>---<br><br>## 🟡 What it does NOT do<br><br>This is important:<br><br>* It **does not fully prevent dislocation**<br>* It cannot replace muscle strength and control<br>* It does not protect you from strong contact or awkward falls<br><br>So even with tape or a brace, the shoulder can still come out if it's not ready.<br><br>---<br><br>## 🟥 When taping is NOT enough	Whether you should wear a brace or use tape depends on your specific sport and the level of stability you've regained through rehab. While these tools can provide support, they are generally considered "extra insurance" rather than a replacement for strengthening exercises.<br><br>Here is a breakdown of the common options for protecting a shoulder during sports:<br><br>## 1. Kinesiology Tape (K-Tape)<br>You have likely seen athletes with colorful strips of tape on their shoulders.<br>* **How it works:** It doesn't physically "hold" the shoulder in place. Instead, it provides **proprioceptive feedback**. The pull of the tape on your skin sends signals to your brain, making you more aware of your shoulder's position.<br>* **Best for:** Non-contact sports or as a psychological "reminder" to maintain good posture and form.<br><br>## 2. Athletic Taping (The "Sling" Wrap)<br>A trainer or physical therapist can use rigid, non-stretching tape to physically limit how far your arm can move.<br>* **How it works:** It creates a mechanical barrier to prevent your arm from moving into the "at-risk" position (usually high and rotated back, like a throwing motion).<br>* **Best for:** High-intensity games where you need a physical limit on your range of motion. The downside is that tape often loosens once you start sweating.<br><br>## 3. Neoprene Sleeves<br>These are simple, slip-on sleeves that provide compression.<br>* **How it works:** Like K-tape, they provide warmth and a sense of "tightness" that helps with joint awareness, but they offer very little mechanical protection against a re-dislocation.<br>* **Best for:** Low-impact activities or the later stages of rehab.<br><br>## 4. Functional Shoulder Braces (The "Sulley" Brace)<br>In contact sports like football, rugby, or wrestling, doctors often recommend a more heavy-duty brace.	Yes, for a first-time dislocation in a 20-year-old athlete, using a brace or tape when you first return to sports is **strongly recommended** by sports medicine specialists. It is not a sign of weakness and it is not a permanent crutch. Think of it like a seatbelt in a car: you do not plan to crash, but you wear it just in case you take an unexpected hit.<br><br>Here is exactly what you need to know about braces and tape, how they work, and when to use them.<br><br>### How Braces and Tape Actually Help<br>They do **not** physically stop the bone from coming out if you put your arm in a dangerous position with full force. The forces in sports are too high for a piece of fabric to stop a dislocation.<br><br>Instead, they serve two critical purposes that are especially important for someone your age returning to play:<br><br>1. **Proprioceptive Reminder (The "Warning Track"):** The brace or tape pulls on your skin when your arm gets close to the **"High-Five / Throwing" position** (abduction and external rotation). It gives you a split-second signal that says, *"Stop. Do not let your arm go further back."* This helps you avoid the position entirely.<br>2. **Compression and Confidence:** After a dislocation, the joint feels "loose" or "empty." The pressure of a brace or tape gives the brain a sense of security and stability, which helps you **play looser and faster**. If you are scared and tense, you are actually more likely to get hurt.<br><br>### Best Options for Anterior Shoulder Dislocation<br><br>**Option 1: The Shoulder Stabilizer Brace (Recommended for Contact Sports)**<br>- **What it looks like:** A sleeve that goes over the bicep and has a strap that wraps around your chest/back, limiting how far your arm can rotate backward.

You should NOT rely on taping if:

* You still feel instability in daily life
* Strength is clearly weaker than the other side
* You haven't completed rehab
* You're returning too early to contact or overhead sports

In those cases, the risk remains high even with support.

🧠 What actually protects your shoulder in sports

The main protection comes from:

* Rotator cuff strength
* Shoulder blade control
* Good coordination and reaction speed
* Proper rehab progression
* Gradual return to sport

These matter far more than external support.

🟦 When taping or a brace can be useful

It may be helpful:

* During late rehab phases
* When returning to sport *after clearance*
* For added confidence in early return phases
* In non-contact or controlled practice settings

⚠️ Important for your situation (age 20)

At your age:

* Risk of re-dislocation is naturally higher
* Bracing alone will not make early return safe
* Rehab completion is much more important than external support

---

✔ Bottom line

* **How it works:** These braces use thick straps to physically tether your arm to your torso, preventing it from reaching the overhead or outward positions where dislocations usually happen.
* **Best for:** High-risk contact sports. While very effective, they can be bulky and may slightly limit your athletic performance or speed.

The Important Catch: Dependency
The biggest risk with using a brace or tape is becoming **psychologically or physically dependent** on it. If you rely on a brace to hold your shoulder together, your muscles might not work as hard to stabilize the joint themselves.

The goal is to use these tools to help you transition back into the game, rather than using them to "bypass" the hard work of physical therapy. Most specialists suggest that if you feel your shoulder is so unstable that it *requires* a brace just to stay in the socket, you might not be ready for sports quite yet.

Are you playing a contact sport (like football or basketball) or a non-contact sport (like tennis or track)?

- **Common Brands:** DonJoy Sully Brace, Shock Doctor Shoulder Support.
- **Best For:** Football, Rugby, Wrestling, Basketball (rebounding), and the first 3-6 months of returning to volleyball or baseball throwing.
- **Important Note:** You must practice in this brace *before* playing in a game. It slightly restricts your range of motion, and you need to get used to catching and throwing with it on.

Option 2: Kinesiology Tape (KT Tape) - (Recommended for Swimming/Non-Contact)
- **How it's applied:** Two specific strips applied from the front of the shoulder (near the armpit) pulling back toward the shoulder blade.
- **Best For:** Swimmers, Tennis players (non-contact), and athletes who find the strap brace too bulky.
- **Limitation:** It does **not** provide a hard stop. It is purely for sensory feedback and slight muscle facilitation. It will not save you if you dive for a ball and land on an outstretched arm.

A Critical Warning About "Bracing"
You cannot use a brace as a substitute for the exercises we discussed earlier. **A brace on a weak shoulder is useless.** The brace is the cherry on top of a fully rehabbed muscle foundation. If you put on a Sully brace but you haven't done your rotator cuff external rotations for 4 months, the shoulder will still slip underneath the brace.

Taping Technique (If You Choose Tape)
If you or your athletic trainer uses rigid athletic tape, it is typically applied in an **"X" pattern** across the front of the shoulder joint. The goal is to create a mechanical block that prevents the arm from going into full "cocking" position (arm back, palm up).

The Long-Term Plan
Most 20-year-olds do not need to wear a brace forever for everyday life. However, for sports participation:
- **Year 1 Post-Dislocation:** Wear the brace for **all** practices and games. This is the highest risk window for re-injury.
- **Year 2 and Beyond:** Many athletes stop wearing the brace but continue to **tape** the shoulder for games as a mental reminder.

Summary: Yes, get fitted for a shoulder stabilizer brace (like the DonJoy Sully or similar) and wear it for sports for at least the first full season back. It is a smart, mature decision that

	* Taping or a brace can give **extra support and confidence**<br>* But it **cannot prevent re-dislocation on its own**<br>* Safe return to sports depends mainly on **rehabilitation, strength, and stability—not equipment**<br><br>---<br><br>If you want, I can tell you **when athletes actually start using braces safely and which sports benefit most from them**, based on real return-to-play guidelines.		significantly lowers your risk of a second, more damaging dislocation. Combine that with the specific rotator cuff exercises, and you give yourself the best possible chance to play without fear.
65	If you experience any of the following signs while playing sports, **you should stop immediately** and get your shoulder checked again by a healthcare professional, as they could indicate that your shoulder isn't fully healed or may be at risk of re-dislocation or other issues.<br><br>---<br><br>## 🚨 Warning signs to stop and get your shoulder checked:<br><br>### 1. **Instability or “slipping” sensation**<br><br>* If you feel like your shoulder **is slipping or about to slip out** during movement, stop immediately.<br>* Any sensation that the shoulder is not **secure or centered** during sports is a red flag.<br><br>---<br><br>### 2. **Sharp or intense pain**<br><br>* A sudden, sharp pain (not just muscle soreness) or a **pinching feeling** inside the shoulder can be a sign of injury or stress to the healing structures.<br>* Pain that doesn’t ease with rest or causes you to change your playing position is a clear sign to stop.<br><br>---<br><br>### 3. **Swelling or increased discomfort**<br><br>* If you notice swelling in the shoulder or surrounding areas after playing sports, it might indicate that the joint is **irritated or injured again**.<br>* **Increased discomfort** (not just mild soreness) that persists after activity should be checked out.<br><br>---	Even after you’ve been cleared to play, you need to be "tuned in" to your shoulder’s signals. Since you’ve already had one dislocation, your body's early warning system is your best defense against a more serious injury.<br><br>You should stop playing and see a doctor or physical therapist if you experience any of the following:<br><br>## 1. The Feeling of "Subluxation" (Slipping)<br>If you feel like the shoulder is "sliding," "shifting," or "popping" in and out, even if it goes right back in on its own, this is a major red flag. This is called a subluxation. It means the ligaments are not holding the joint tight enough, and a full dislocation could be next.<br><br>## 2. "Dead Arm" Syndrome<br>If you take a hit or make a sudden movement and your arm suddenly feels weak, numb, or "dead" for a few seconds or minutes, you need to stop. This sensation often indicates that the joint shifted enough to temporarily irritate the nerves or blood vessels.<br><br>## 3. Sharp, Stabbing Pain<br>Muscle soreness after a game is normal, but sharp, localized pain—especially deep inside the joint or at the front of the shoulder—is not. If the pain is sharp enough to make you "wince" or change the way you move (like changing your throwing motion to avoid pain), something is wrong.<br><br>## 4. New or Increased Instability<br>If you start feeling "guarded" or anxious about your shoulder during routine movements, your brain is likely picking up on instability that you haven't consciously noticed yet. Trust that "apprehension" feeling; it’s usually your body’s way of telling you the joint is vulnerable.<br><br>## 5. Night Pain or Persistent Swelling<br>* **Night Pain:** Pain that wakes you up at night or makes it impossible to sleep on that side often indicates significant inflammation or a tear that isn't healing.	This is one of the most important questions for long-term shoulder health. Knowing when to stop is the difference between a minor setback and a major surgical problem. Since you are 20 and have a first-time dislocation, you need to be very clear on the **"Stop Signs"** versus the **"Normal Rehab Pains."**<br><br>Here are the specific symptoms that mean you should stop playing immediately and see your sports medicine doctor or physical therapist again.<br><br>### 1. The "Dead Arm" Sensation (Stop Immediately)<br>This is not just pain. This is a specific neurological event.<br>- **What it feels like:** You go to throw a ball, swing a racquet, or reach for a rebound, and suddenly your entire arm feels **numb, heavy, and weak for a few seconds to minutes.** You may feel like you can't grip anything.<br>- **Why you must stop:** This is a **Subluxation** (partial dislocation). The ball of the shoulder slid forward onto the rim of the socket, stretched the nerves, and then slid back. You did not need to go to the ER to pop it back in, but **damage was done.** Every time this happens, the bone socket gets more dented (Hill-Sachs lesion). If you feel Dead Arm, your season is over for the day, and you need a re-evaluation.<br><br>### 2. A Feeling of "Slipping" or "Clunking" During a Specific Motion<br>- **What it feels like:** When you do a push-up, or when you reach into the back seat of the car, you feel a **clunk** or a **shift** inside the joint that you can sometimes even hear.<br>- **Normal vs. Not Normal:** *Normal* is popping/clicking with no pain (this is often just tight muscles snapping over bone). *Not Normal* is a deep **thud** or **shift** that causes a brief moment of panic or nausea.<br>- **Why you must stop:** This indicates the labrum (cartilage bumper) has not healed in the correct position. Playing through this will only tear it more.<br><br>### 3. Pain in the **Back** of the Shoulder (Posterior Pain)

4. **Weakness or loss of function**

* If your shoulder feels **weak** or you lose **strength or control** during sport-specific movements, it's a sign that the muscles and stabilizers aren't ready for the stress of sport yet.
* Difficulty in using your arm for basic actions (like throwing, catching, or pushing) during sport means the joint isn't stable.

---

5. **Unusual clicking or popping**

* If you hear or feel **clicking, popping, or grinding** in the shoulder joint during movement, especially if it's associated with discomfort, it could indicate that the labrum or other structures haven't healed properly.

---

6. **Pain or discomfort in the neck or upper arm**

* Pain radiating from the shoulder down the arm or into the neck could suggest that the **nerve structures** are being irritated.
* This can be a sign that the shoulder joint is not yet moving correctly or that there is too much strain on the surrounding tissues.

---

7. **Significant fatigue or muscle strain**

* If your shoulder muscles feel **fatigued or strained far quicker than usual**, it may indicate that they are still not strong enough or aren't supporting the joint adequately.
* This fatigue, especially during sport, should be taken seriously as it could compromise stability.

---

8. **Apprehension or fear of movement**

* If you find yourself avoiding certain **movements** because you feel **apprehensive or scared** of re-injury, or if the thought of certain sports or movements makes you nervous, your shoulder may not be fully ready for the demands of sport.

🧠 What should happen instead

* **Swelling:** Visible swelling or a "full" feeling in the joint capsule after activity means the shoulder is being overstressed.

6. Catching or Locking
If your shoulder feels like it's "catching" on something or physically locking up when you try to rotate it, this could mean a piece of the labrum (cartilage) is torn and getting caught in the joint.

The Golden Rule: If you have to "check" your shoulder or "pop" it back into place to feel comfortable, you are risking permanent damage to the bone and cartilage. It is much easier to fix a minor instability with a few weeks of focused rehab than it is to recover from a second full dislocation.

Does your shoulder currently feel stable during your daily activities, or do you find yourself being extra cautious with it?

- **What it feels like:** You would expect the front of the shoulder to hurt (where it came out). But if you start feeling a deep, sharp ache **in the back of the shoulder joint** (near the armpit from behind), stop.
- **Why you must stop:** When the front of the shoulder is loose, the ball drifts backward during certain exercises (like bench press or dips) and **pinches the rotator cuff against the bone.** This is called Internal Impingement. Continuing to play will fray and tear the rotator cuff tendon. A torn cuff is a much bigger surgery than a dislocation repair.

4. Night Pain That Wakes You Up
- **What it feels like:** You can play through the game, but 3 hours later you are lying in bed and you cannot find a comfortable position for the arm. You wake up multiple times per night needing to move it.
- **Why you must stop:** This is a classic sign of **Synovitis** (joint inflammation) or a developing **Frozen Shoulder** phase. If you are losing sleep because of shoulder ache, the joint is not ready for the impact of sports. Rest and anti-inflammatory management (guided by a doctor) are required.

5. Loss of Motion You Used to Have
- **What it looks like:** Last week you could wash your hair with both hands. This week, you can only get your hand up to your forehead before it "sticks" or hurts sharply.
- **Why you must stop:** If your range of motion is **decreasing** instead of increasing, you may be developing **Adhesive Capsulitis** (Frozen Shoulder) or you have a loose piece of cartilage floating in the joint. Playing sports will make this stiffness permanent.

What About Just Being "Sore"?
It is completely normal for the muscles around your shoulder blade (rhomboids, traps) and the **front** of the shoulder (pec minor) to be sore after activity. That is just muscle fatigue from working hard to stabilize the loose joint.
- **Rule of Thumb:** Muscle soreness is fine. Joint "looseness" or nerve "zingers" are not fine.

The Final Check: The Apprehension Test (Self-Check)
You can do this gently at home **after** activity to see if you should go back to the doctor:
1. Lie on your back on the floor.
2. Have a friend or parent lift your injured arm to **90 degrees out to the side** (like half of a snow angel).
3. Bend your elbow to 90 degrees so your hand points to the ceiling.

	When your shoulder is truly ready for sports:<br><br>* You should be able to play without fear of it **slipping out**.<br>* It should feel **strong and stable**, even in high-stress positions (like overhead or contact).<br>* **No pain** or excessive discomfort during or after sports.<br>* **Confidence** in your shoulder should increase with each practice or session.<br><br>---<br><br>## 🟢 Bottom line:<br><br>If you experience **instability, pain, weakness, or swelling** while playing, stop immediately and get your shoulder checked. Early signs of reinjury or instability can lead to longer recovery times or even the need for surgical intervention if ignored.<br><br>---<br><br>Would you like me to break down **specific tests or motions** to be cautious of during your sport that may indicate it's time to stop and seek advice?		4. Have them gently try to rotate your hand toward the floor behind your head (External Rotation).<br><br>**If you feel like you have to grab their arm to stop them or you feel panic/fear in your stomach before you feel pain:** The shoulder is still unstable and you should **not** be playing contact or overhead sports. You need more rehab or a surgical consult.<br><br>**Summary:** Stop and get checked if you feel **numbness, a clunk/shift, or if you start waking up at night in pain.** Do not try to be a hero and play through these specific symptoms.
