# Supplementary material for: Large Language Models in Shoulder Dislocation: Performance Across Medical and Rehabilitation Domains
Source: Healthcare (Basel). 2026 Nov 3;14(21):3726. doi: 10.3390/healthcare14213726 (PMC13763467; doi:10.3390/healthcare14213726)
Supplement: Supplementary file 1 [file healthcare-14-03726-s001.zip › Supplementary_Table_S1_Clinical_Themes.pdf]

Table S1. Questions used to evaluate large language model responses and their primary clinical themes.

No.	Question	Clinical Theme
**Medical and Safety Domain (n = 28)**		
1	My shoulder came out. Do I need to go to the emergency room right away?	Acute management and safety
2	Can I try to put my shoulder back in place by myself?	Acute management and safety
3	Is it okay to move my arm at all?	Acute management and safety
4	If I move my shoulder too soon, can it cause problems?	Acute management and safety
5	What should worry me after my shoulder comes out?	Acute management and safety
6	Is it normal to feel numbness or tingling in my arm after my shoulder comes out?	Acute management and safety
7	Is it okay to wait at home and see if the pain gets better?	Acute management and safety
8	Are there things I should avoid doing now?	Acute management and safety
9	Do I really need to wear a sling after my shoulder dislocation?	Immobilization and post-reduction symptoms
10	How long do I need to keep my arm in a sling?	Immobilization and post-reduction symptoms
11	Can taking the sling off too early cause problems?	Immobilization and post-reduction symptoms
12	Is it okay to take my arm out of the sling sometimes?	Immobilization and post-reduction symptoms
13	Should I keep my shoulder completely still while wearing the sling?	Immobilization and post-reduction symptoms
14	Is pain still normal after my shoulder has been put back in place?	Immobilization and post-reduction symptoms
15	My shoulder was put back in place, but it's swollen and stiff. Is this normal?	Immobilization and post-reduction symptoms
16	Is my shoulder likely to come out again?	Recurrence and instability
17	What happens if my shoulder comes out again?	Recurrence and instability
18	Does my age affect the chances of my shoulder coming out again?	Recurrence and instability
19	How can I tell if my shoulder feels unstable or might come out again?	Recurrence and instability
20	When should I see a doctor again, even if my shoulder feels better?	Follow-up and surgical management
21	If my shoulder comes out again, should I go to the hospital right away?	Recurrence and instability
22	Will I need surgery later on?	Follow-up and surgical management
23	Is it better to have surgery early, or can my shoulder heal well without surgery?	Follow-up and surgical management
24	In what situations would surgery be recommended?	Follow-up and surgical management
25	Could my shoulder be permanently damaged because of this?	Recurrence and instability
26	Can I take painkillers? Which ones are safe?	Pain medication
27	How often can I take pain medication?	Pain medication
28	Do I need an MRI or any other imaging?	Imaging
**Rehabilitation Domain (n = 37)**		
29	Can I sleep normally after my shoulder dislocation, or do I need a special position?	Daily activities
30	Is it okay to sleep on my injured shoulder?	Daily activities
31	Can I shower or take a bath with my shoulder injury?	Daily activities
32	Can I use my arm for simple daily things like eating, brushing my teeth, or using my phone?	Daily activities
33	Is it okay to go back to school?	Daily activities
34	Can I carry a backpack or school bag with my injured shoulder?	Daily activities
35	Can I carry things like a bag, books, or shopping bags with my injured arm?	Daily activities
36	Are there any daily movements at home or school that I should avoid?	Daily activities
37	Is it normal to feel tired or uncomfortable when using my arm during daily activities?	Daily activities
38	Can I sit at a desk, write, or use a computer normally?	Daily activities
39	Do I need to be very careful with my arm during daily activities, or can I use it normally?	Daily activities
40	When can I start exercising my shoulder?	Exercise initiation and progression
41	Can I do shoulder exercises at home, or do I need someone to show me how?	Exercise initiation and progression
42	Can starting exercises too early cause my shoulder to come out again?	Exercise initiation and progression
43	What kind of exercises should I start with?	Exercise initiation and progression
44	Should I stop exercising if I feel pain while doing the exercises?	Exercise initiation and progression
45	Is it normal for exercises to feel uncomfortable at first?	Exercise initiation and progression
46	How often should I do my shoulder exercises?	Exercise initiation and progression
47	How do I know if I'm doing the exercises correctly?	Exercise initiation and progression
48	If I skip my exercises, can it slow my recovery or cause problems?	Exercise initiation and progression
49	Will strengthening my shoulder help stop it from coming out again?	Strengthening and shoulder stability
50	When can I start strengthening my shoulder?	Strengthening and shoulder

No.	Question	Clinical Theme
		stability
51	How can I tell if my shoulder is getting stronger and feels more secure?	Strengthening and shoulder stability
52	Is it normal to feel shaky or unsure when using my shoulder during exercises?	Strengthening and shoulder stability
53	Why does my shoulder still feel like it could come out even though I'm doing my exercises?	Strengthening and shoulder stability
54	How long does it usually take for my shoulder to feel strong and stable again?	Strengthening and shoulder stability
55	Can doing the wrong exercises make my shoulder feel worse or come out again?	Strengthening and shoulder stability
56	Will strengthening exercises help me feel more confident using my shoulder?	Strengthening and shoulder stability
57	When can I go back to sports?	Return to sport
58	How will I know if my shoulder is ready for sports again?	Return to sport
59	Can I go back to sports even if my shoulder doesn't feel completely normal yet?	Return to sport
60	Are some sports more likely to make my shoulder come out again?	Return to sport
61	Is it normal to feel scared about using my shoulder again in sports?	Return to sport
62	Can going back to sports too early cause my shoulder to come out again?	Return to sport
63	Do I need to do specific exercises before I can play sports again?	Return to sport
64	Do I need to wear something on my shoulder or tape it when I play sports?	Return to sport
65	What should make me stop playing sports and get my shoulder checked again?	Return to sport
